# Regioselective C2-Sulfonylation of Indoles and Pyrroles via SO_2_ Insertions

**DOI:** 10.1021/acs.orglett.6c00146

**Published:** 2026-02-04

**Authors:** Rekha Bai, Wan-Lin Cheng, Chun-Yu Peng, Yu-Hao Chen, Pin-Han Wang, Chin-Fa Lee

**Affiliations:** † Department of Chemistry, 34916 National Chung Hsing University, Taichung City 40227, Taiwan (R.O.C.); ‡ i-Center for Advanced Science and Technology (iCAST), National Chung Hsing University, Taichung City 40227, Taiwan (R.O.C.); § Innovation and Development Center of Sustainable Agriculture (IDCSA), National Chung Hsing University, Taichung City 40227, Taiwan (R.O.C.)

## Abstract

A molecular iodine-catalyzed three-component cascade reaction of indoles/pyrroles, anilines, and DABSO has been developed, providing C2-sulfonylated indoles/pyrroles in good to excellent yields. The transformation proceeds via the *in situ* generation of the arylsulfonyl radical from the reaction of anilines, ^*t*^BuONO, and DABSO, followed by controlled formation of a carbon-centered radical intermediate. In this radical-mediated cascade reaction, DABSO acts as the sulfone (SO_2_) source while ^*t*^BuONO facilitates the generation of reactive species. Moreover, this one-pot transformation proceeds under mild conditions, exhibits a broad substrate scope, and offers an efficient and sustainable strategy for the construction of C2-sulfonated indoles and pyrroles.

## Graphical abstract

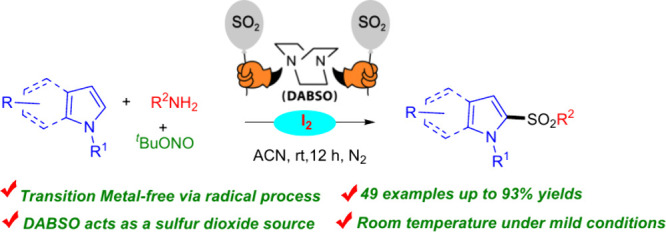

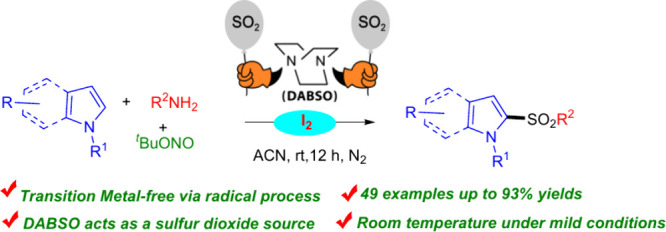

Multicomponent reactions (MCRs) involve the combination of three or more starting materials in one pot, efficiently incorporating most atoms from the reactants into the final products.^1^ Recently, sulfur dioxide insertion-based multicomponent reactions have attracted a considerable amount of attention as efficient one-pot strategies for the synthesis of organosulfones.^2^ These transformations are highly appealing due to their straightforward, single-step construction of sulfonyl-containing compounds.^3^ Traditionally, gaseous SO_2_ was commonly used as the sulfur dioxide source; however, its toxic and corrosive nature limits its practical use in academic laboratories.^4^ To overcome this disadvantage, the development of stable and user-friendly sulfur dioxide surrogates has received much attention in recent years. Various SO_2_ surrogates such as DABSO, Na_2_S_2_O_5_, K_2_S_2_O_5_, rongalite, thiourea, and SOgen have been reported over the past decade.^2b,5^ Among them, the 1,4-diazabicyclo[2.2.2]­octane bis­(sulfur dioxide) adduct (DABSO), which is bench stable, solid, and easy to handle, has emerged as a safer and more convenient alternative, serving as an efficient surrogate for SO_2_.^1b^ Owing to these advantageous properties, it is widely employed for the synthesis of sulfur-containing compounds under mild reaction conditions, including sulfones,^6^ sulfonyl halides,^4b,7^ sulfonohydrazides,^8^ sulfonic esters,^9^ and sulfonothioesters, among others.^5a^

Organosulfones represent a valuable class of organic compounds, widely used both as synthetic intermediates and as final bioactive products in pharmaceuticals and agrochemicals.^10^ They were also employed as protecting and activating groups in various synthetic transformations. In particular, heteroaryl (indoles or pyrroles) sulfones have attracted a significant amount of attention because of their broad spectrum of biological activities, including anti-HIV-1, antibacterial, antifungal, and antitumor properties (Figure 1).^11^

**Figure 1:**
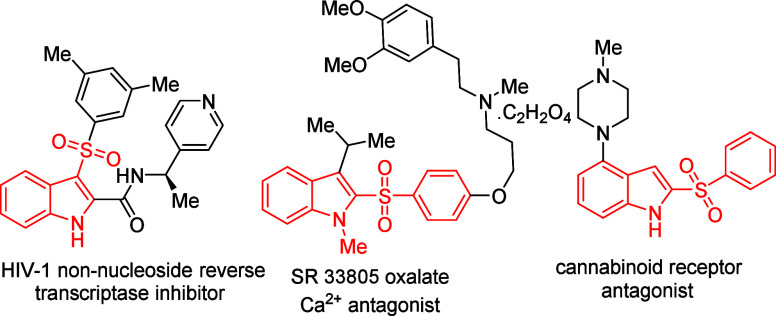
Selected representative examples of biologically active sulfonylated indoles.

Indoles have long been a central focus in synthetic organic chemistry due to their widespread occurrence in natural products and pharmaceuticals, along with their notable synthetic versatility.^12^ However, introducing sulfonyl groups, especially at the C2 position of the indole ring, remains challenging, as the C3 position is typically more reactive and thus easily functionalized. While many strategies for sulfonylation at the C3 position exist,^13^ C2-sulfonylation remains less explored and is synthetically demanding. Conventionally, C2-sulfonylindoles have been prepared either by oxidation of indolyl aryl sulfides using Oxone or mCPBA under anhydrous conditions (Scheme 1A) or through C–H functionalization strategies involving hydrazines, sulfonyl halides, or aryl sulfonic acids as coupling partners (Scheme 1B).^14,15^ The groups of Deng,^15b^ Kuhakarn,^15d^ Zhang,^15c^ and Yan^15f^ have independently reported the synthesis of C2-sulfonylated indoles using indoles and sodium sulfinates as coupling partners, employing I_2_/TBHP in AcOH, I_2_/MeOH, NH_4_I/TBHP in AcOH, and KI/Oxone as catalyst systems under heating or room-temperature conditions. Additionally, Liu and co-workers^15e^ have achieved the synthesis of C2-sulfonylated indoles via the reaction of indoles with hydrazides using an iodophor/H_2_O_2_ system (Scheme 1B).

**Scheme 1:**
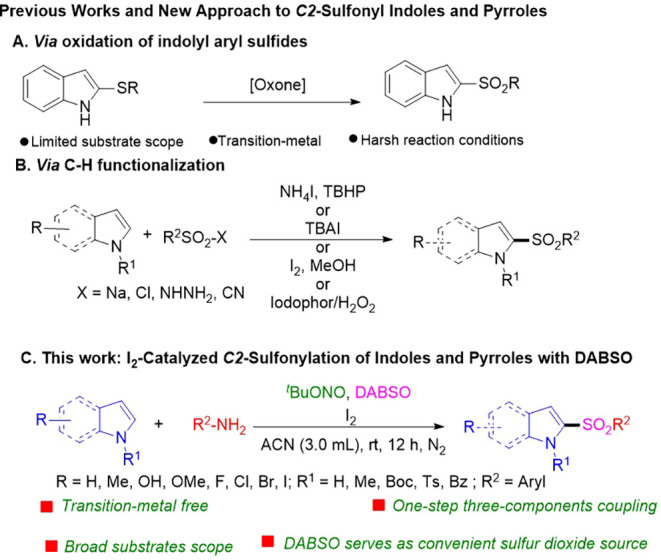
Previous Works and a New Approach to C2-Sulfonyl Indoles and Pyrroles

The use of molecular iodine and its salt as catalysts in organic transformations has attracted a significant amount of attention due to the growing demand for greener and more sustainable chemical processes. Owing to its ease of handling, commercial availability, mild reactivity, and specifically metal-like behavior, iodine has emerged as a valuable and efficient catalyst for promoting the formation of C–C and C–heteroatom bonds.^16^ Recently, we reported a radical process synthesis of sulfonamides through a reaction of anilines, a nitrite source, and DABSO.^8b^ In this transformation, the *in situ*-generated aryl sulfonyl radical was identified as the key intermediate. Based on these insights, we intended that the indoles and pyrroles might also be utilized as the substrates in the insertion reaction of sulfur dioxide. Herein, we report a novel molecular iodine-catalyzed strategy for the direct C2-sulfonylation of indoles and pyrroles through a three-component C–S coupling reaction involving indoles/pyrroles, anilines, DABSO, and TBN (^*t*^BuONO). In this transformation, the aryl sulfonyl group is introduced through a radical sulfonylation pathway mediated by DABSO (Scheme 1C). This approach offers several advantages. It is metal-free, employs a stable SO_2_ surrogate, and proceeds in a one-pot manner, making it operationally simple and broadly applicable.

After a series of optimization experiments were performed, the optimal reaction conditions for this multicomponent sulfur dioxide insertion reaction were established as follows: indole (**1a**, 0.5 mmol), aniline (**2a**, 0.75 mmol), ^*t*^BuONO (1.0 mmol), DABSO (0.6 mmol), and I_2_ (0.6 mmol) in acetonitrile (3 mL) at room temperature under a nitrogen atmosphere for 12 h. Under these conditions, desired product 2-(phenylsulfonyl)-1*H*-indole (**3aa**) was isolated in 83% yield (Table 1, entry 1). Amendment of the reaction parameters provided the following results. No desired product **3aa** was detected in the absence of I_2_ or ^*t*^BuONO (Table 1, entry 2 or 3, respectively). When KI or NH_4_I was used instead of I_2_, the reaction furnished **3aa** in 21% or 58% yield, respectively, (Table 1, entry 4 or 5, respectively). A trace amount of product **3aa** was detected when TBAI was employed instead of iodine (Table 1, entry 6). The use of inorganic SO_2_ surrogates such as K_2_S_2_O_5_ and Na_2_S_2_O_5_ resulted in no product formation (Table 1, entries 7 and 8, respectively). Performing the reaction in 1,4-dioxane or DMSO afforded desired product **3aa** in only trace or 30% yield, respectively, while DMF and toluene provided **3aa** in 63% or 25%, respectively (Table 1, entries 9–12). Next, other nitrite sources such as isomylONO, ^*n*^BuONO, and NaNO_2_ were examined. IsomylONO and ^*n*^BuONO afforded desired product **3aa** in 53% and 49% yields, respectively, whereas NaNO_2_ failed to produce the desired product (Table 1, entries 13–15). Increasing the amount of iodine to 1.0 mmol or decreasing the reaction time to 8 h did not significantly influence the yield of product **3aa** (Table 1, entry 16 or 17, respectively). Subsequently, when the reaction was conducted using water as the solvent, only a trace amount of product **3aa** was detected (Table 1, entry 18). Furthermore, performing the reaction under an oxygen atmosphere led to the formation of **3aa** in only 15% yield (Table 1, entry 19).

**Table 1:**
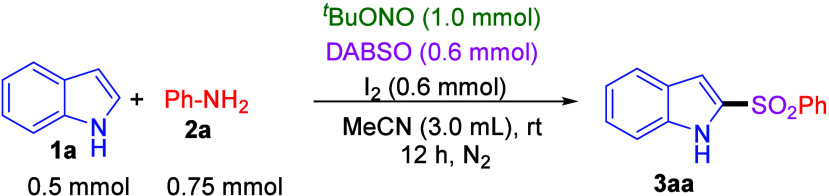
Optimization Conditions for the Reaction of *NH*-Indole **1a**, DABSO, Aniline **2a**, and BuONO^a^

entry	deviation from the standard conditions	yield of **3aa** (%)^b^
1	no change	83
2	without I_2_	ND^a^
3	without TBN	ND^a^
4	KI instead of I_2_	21
5	NH_4_I instead of I_2_	58
6	TBAI instead of I_2_	trace
7	K_2_S_2_O_5_ instead of DABSO	ND^a^
8	Na_2_S_2_O_5_ instead of DABSO	ND^a^
9	1,4-dioxane instead of MeCN	trace
10	DMSO instead of MeCN	30
11	DMF instead of MeCN	63
12	toluene instead of MeCN	25
13	isoamyl ONO instead of ^*t*^BuONO	53
14	^*n*^BuONO instead of ^*t*^BuONO	49
15	NaNO_2_ instead ^*t*^BuONO	NR
16	1.0 mmol of I_2_	85
17	8 h instead of 12 h	56
18	H_2_O instead of MeCN	trace
19	under an O_2_ atmosphere	15

a For the reactions, **1a** (0.5 mmol, 1.0 equiv), **2a** (0.75 mmol, 1.5 equiv), TBN (1.0 mmol, 2.0 equiv), DABSO (0.6 mmol, 1.2 equiv), and I_2_ (0.6 mmol, 1.2 equiv) were reacted in MeCN (3 mL) under a nitrogen atmosphere at room temperature for 12 h.

b Isolated yield.

c Desired product not detected.

With the optimized reaction conditions established, we next explored the substrate scope of this multicomponent transformation (Table 2). Initially, the reactivity of various indole derivatives was examined by using aniline **2a**, ^*t*^BuONO, and DABSO as coupling partners. Indoles bearing electron-donating substituents at position C5 such as 5-methylindole (**1b**), 5-hydroxyindole (**1c**), and 5-methoxyindole (**1d**) smoothly underwent the transformation with **2a**, ^*t*^BuONO, and DABSO, delivering 2-sulfonylindoles **3aa–3da** in 83–87% yields. Likewise, indoles, containing electron-withdrawing substituents at positions C5 and C6, i.e., 5-fluoroindole (**1e**), 5-chloroindole (**1f**), 5-bromoindole (**1g**), 5-iodoindole (**1h**), and 6-chloroindole (**1i**), were also efficiently reacted with **2a**, ^*t*^BuONO, and DABSO under the optimized reaction conditions, affording desired C2-sulfonylated indoles **3ea–3ia**, respectively, in 67–78% yields. Additionally, *N*-methylindole (**1j**), *N*-Boc indole (**1k**), *N*-tosyl indole (**1l**), and *N*-benzyl indole (**1m**) were also compatible with aniline **2a**, providing products **3ja–3ma**, respectively, in 75–88% yields. These results clearly demonstrate that the electronic nature and substitution pattern on the indole ring have a minimal influence on the reaction efficiency. Subsequently, the effect of substituents on the aniline moiety was systematically investigated. 4-Methyl aniline **2b** smoothly participated in the reaction with various indole derivatives possessing electron-withdrawing and electron-donating groups at position C5 or C6 (i.e., 5-Me, 5-OH, 5-OMe, 5-Cl, 5-Br, 5-F, and 6-Cl). In all of these cases, C2-sulfonylindoles **3ab–3gb** and **3ib** were obtained in 60–92% isolated yields, highlighting the notable functional group tolerance of this strategy.

**Table 2:**
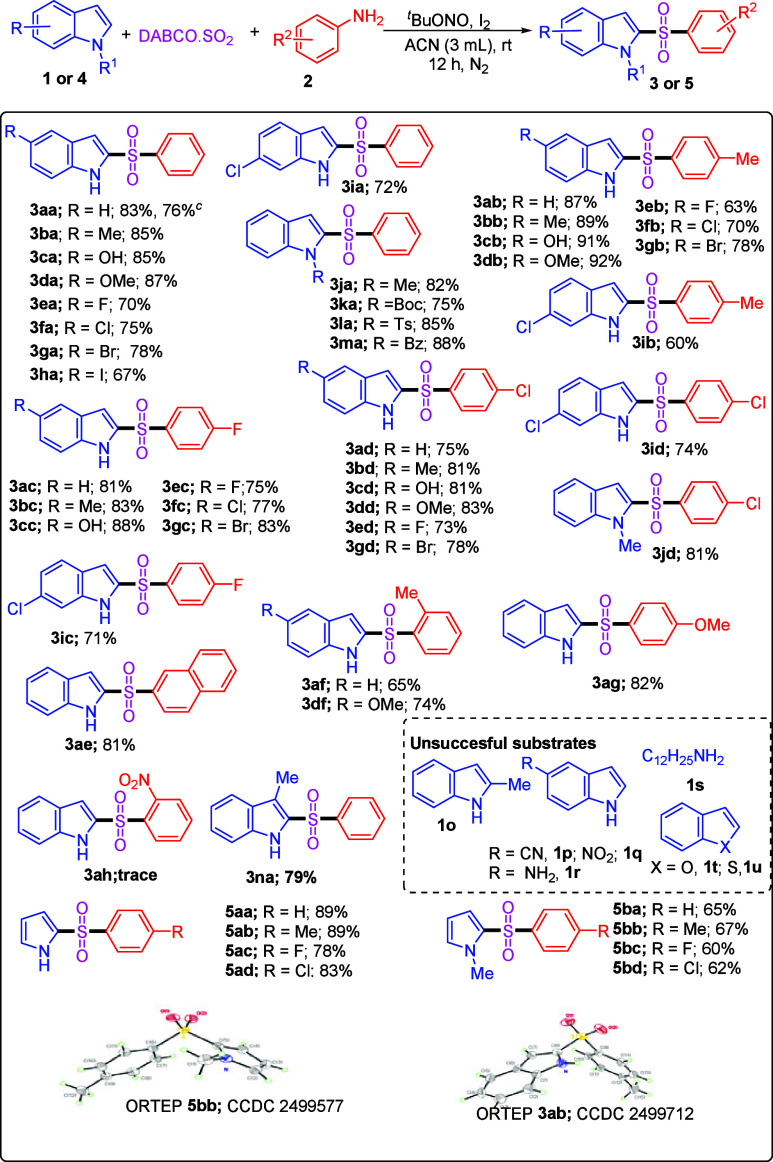
Substrate Investigation for the Arylsulfonation Reaction of Indoles/Pyrroles and Anilines^a^,^b^

a For the reactions, **1** or **4** (0.5 mmol, 1.0 equiv), **2** (0.75 mmol, 1.5 equiv), *^t^* BuONO (1.0 mmol, 2.0 equiv), DABSO (0.6 mmol, 1.2 equiv), and I_2_ (0.6 mmol, 1.2 equiv) were reacted in MeCN (3 mL) under a nitrogen atmosphere at room temperature for 12 h.

b Isolated yield.

c Reaction on a 5.0 mmol scale.

Next, we turned our attention toward the electron-deficient anilines like 4-fluoroaniline (**2c**) and 4-chloroanilines (**2d**) for this multicomponent reaction. First, 4-fluoroaniline **2c** smoothly reacted with electron-rich *NH*-indole (**1a**), 5-methylindole (**1b**), and 5-hydroxyindole (**1c**) to deliver C2-sulfonylindoles **3ac–3cc**, respectively, in 81–88% isolated yields. Indoles bearing electron-withdrawing groups at position C5 or C6 were also smoothly reacted with **2c**, ^*t*^BuONO, and DABSO under the standard reaction conditions, delivering **3ec–3gc** and **3ic** in 71–83% yields. Similarly, 4-chloroaniline (**2d**) was also compatible with electron-rich and electron-deficient indoles, ^*t*^BuONO, and DABSO, affording desired corresponding C2-sulfonylindoles **3ad–3gd** and **3id** in 73–83% yields. *N*-Methylindole (**1j**) also smoothly reacted with 4-chloroaniline (**2d**), ^*t*^BuONO, and DABSO, providing 2-((4-chlorophenyl)­sulfonyl)-1-methyl-1*H*-indole (**3jd**) in 81% yield. Furthermore, we carried out the reaction using *NH*-indole (**1a**) and naphthalen-2-amine (**2e**) under the optimized reaction conditions and successfully afforded desired 2-(naphthalen-2-ylsulfonyl)-1*H*-indole (**3ae**) in 81% yield. Similarly, 2-methylaniline (**2f**) proved to be an effective coupling partner and reacted smoothly with *NH*-indole (**1a**) and 5-methoxyindole (**1d**) in the presence of DABSO and ^*t*^BuONO under the standard reaction conditions, affording products **3af** and **3df** in 65% and 74% yields, respectively. Subsequently, we tested other aniline derivatives, such as 4-methoxyaniline (**2g**) and 2-nitroaniline (**2h**), under the standard reaction conditions. While **3ag** was isolated in 82% yield, **3ah** was formed only in trace amounts. To further examine the selectivity, reactions of 3-methylindole (**1n**) and 2-methylindole (**1o**) with **1a**, ^*t*^BuONO, DABSO, and iodine under the standard conditions were carried out. The reaction proceeded smoothly with 3-methylindole, affording desired product **3na** in 79% yield, whereas no desired product was obtained from 2-methylindole. Unfortunately, **1o**–**1u** proved to be unsuccessful substrates under these reaction conditions.

Encouraged by this observation, we then extended the scope to pyrrole derivatives. *NH*-Pyrrole (**4a**) was efficiently reacted with electron-rich and electron-deficient aniline (**2a**), *p*-toluidine (**2b**), 4-fluoroaniline (**2c**), and 4-chloroaniline (**2d**), ^*t*^BuONO, and DABSO, affording products **5aa–5ad**, respectively, in 78–89% yields. Similarly, *N*-methyl pyrrole was also undergoing smooth coupling with aniline (**2a**), *p*-toluidine (**2b**), 4-fluoroaniline (**2c**), and 4-chloroaniline (**2d**), efficiently converted into 2-sulfonylated pyrroles **5ba–5bd**, respectively, in 60–67% yields under the optimized reaction conditions. Furthermore, to evaluate the efficiency of the reaction on a gram scale, we performed the reaction on a 5.0 mmol scale, which afforded compound **3aa** in 76% isolated yield (details in the Supporting Information). The structures of products **3** and **5** were established by ^1^H NMR, ^13^C NMR, and HRMS analysis, while the structures of products **3ab** and **5bb** ^17c^ were further confirmed through single-crystal X-ray diffraction (XRD) analysis (see the Supporting Information).

To gain insight into the reaction mechanism, a series of control experiments were performed using indole (**1a**, 0.5 mmol), DABSO (0.6 mmol), aniline (**2a**, 0.75 mmol), and ^*t*^BuONO (1.0 mmol) as model substrates under the optimized reaction conditions in the presence of various radical scavengers (Scheme 2a). The addition of TEMPO (1.5 mmol) to the reaction mixture completely suppressed the formation of desired product **3aa**, and instead, TEMPO-trapped radical adducts **6** and **7** were detected by FTMS+ESI, confirming the involvement of radical intermediates. Similarly, when DMPO (1.5 mmol) was added under identical reaction conditions, target product **3aa** was not detected; rather, DMPO-trapped adducts **8** and **9** were identified in the FTMS+ESI analysis. In addition, EPR experiments were performed under these conditions, which revealed mixed EPR signals (details in the Supporting Information). Furthermore, a control reaction was conducted to examine the possible interactions between aniline (**2a**) and ^*t*^BuONO in acetonitrile. FTMS+ESI analysis of this reaction revealed the formation of *N*-hydroxybenzenediazene (Ph–N=N–OH) (**10**), suggesting the generation of a diazinyl-type radical species (Scheme 2b). Collectively, these control experiments strongly support that the present multicomponent transformation proceeds via a radical-mediated reaction pathway involving arylsulfonyl radical intermediates.

**Scheme 2:**
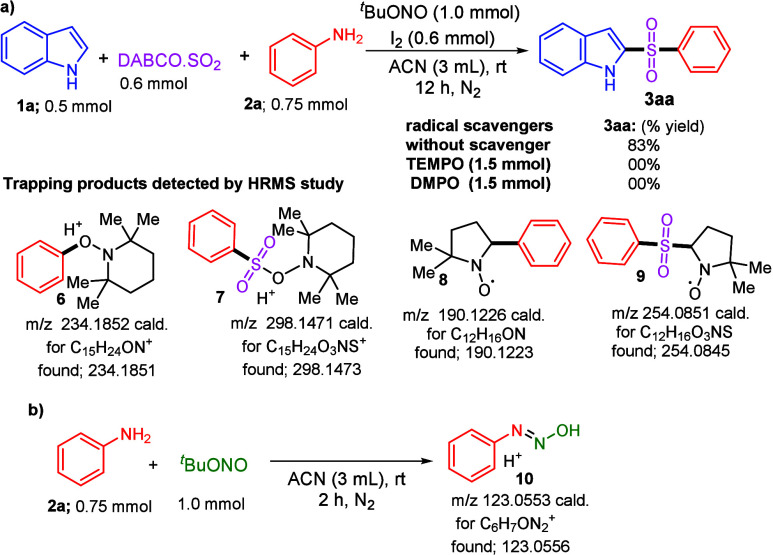
Control Experiments

Based on control experiments and previous literature reports,^2–5,15,17^ a possible reaction pathway for this multicomponent SO_2_ insertion reaction is illustrated in Scheme 3. Initially, ^*t*^BuONO reacts with aniline to form *N*-hydroxybenzenediazonium **A** (Ph–N=N–OH) and ^*t*^BuOH. This intermediate undergoes homolytic cleavage with release of nitrogen gas and water molecule to generate aryl radical **B**. The aryl radical then reacts with sulfur dioxide (SO_2_) released from DABSO to give aryl sulfonyl radical **C**. Instantly, this aryl sulfonyl reacts with indole to produce indolyl radical **D**. This indolyl radical reacts with iodine and furnishes intermediate **E**. Subsequent elimination of HI delivers final 2-sulfonylated product **3** or **5**.

**Scheme 3:**
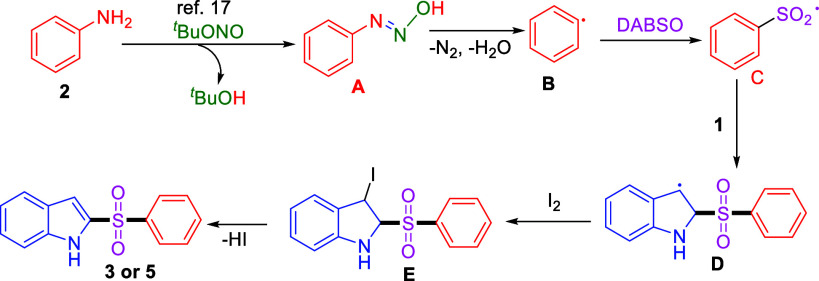
Plausible Mechanism

In summary, we have developed an efficient transition-metal-free, molecular iodine-catalyzed strategy for the regioselective synthesis of C2-sulfonylated indoles and pyrroles via a one-pot three-component reaction of indoles/pyrroles, anilines, ^*t*^BuONO, and DABSO. In this transformation, DABSO serves as the sulfur dioxide source, enabling the *in situ* formation of the arylsulfonyl radical intermediate through the combination of aryl radical (generated from anilines and ^*t*^BuONO) and sulfur dioxide. Molecular iodine served as an efficient iodide catalyst, while acetonitrile was identified as the optimal solvent. This operationally simple and sustainable protocol features a readily available substrate, mild conditions, a broad substrate scope, and excellent functional group tolerance, affording desired C2-sulfonylated indoles and pyrroles in 60–93% yields. Furthermore, its gram-scale applicability and mechanistic clarity highlight its synthetic utility for constructing biologically relevant sulfonylated heterocycles.

**Safety:** *Caution! All reactions involve the explosive nature of diazo-type intermediates. In our protocol, these intermediates are generated in situ and consumed immediately, preventing their accumulation. All reactions were conducted under dilute conditions behind a safety shield, following standard precautions for handling reactive intermediates*.

## Supplementary Material



## Data Availability

The data underlying this study are available in the published article and its Supporting Information.

## Notes

General experimental procedure for the synthesis of compounds **3** and **5**, large-scale synthesis details, EPR studies, and complete characterization data for all compounds, including XRD (X-ray crystallographic) details, copies of all NMR spectra, and copies of HRMS spectra (PDF)
