# Supplementary material for: Regioselective C2-Sulfonylation of Indoles and Pyrroles via SO2 Insertions
Source: Org Lett. 2026 Feb 4;28(7):2482–7. doi: 10.1021/acs.orglett.6c00146 (PMC12930489; doi:10.1021/acs.orglett.6c00146)
Supplement: Supplementary file 1 [file ol6c00146_si_001.pdf]

# Regioselective *C2*-Sulfonylation of Indoles and Pyrroles *via* $SO_2$ Insertions

Rekha Bai,[a] Wan-Lin Cheng,[a] Chun-Yu Peng,[a] Yu-Hao Chen,[a] Pin-Han Wang[a] and Chin-Fa Lee*[a,b,c]

[a] Department of Chemistry, National Chung Hsing University, Taichung City 40227, Taiwan (R.O.C.)
[b] *i*-Center for Advanced Science and Technology (*i*CAST), National Chung Hsing University, Taichung City 40227, Taiwan (R.O.C.)
[c] Innovation and Development Center of Sustainable Agriculture (IDCSA), National Chung Hsing University, Taichung City 40227, Taiwan (R.O.C.)

*Corresponding author. E-mail: cfalee@dragon.nchu.edu.tw


| **Entry** | **Contents** | **Pg. No** |
|---|---|---|
| 1 | General informations | S2 |
| 2 | General procedure for the synthesis of compounds **3** and **5** | S2 |
| 3 | Large scale synthesis | S3 |
| 4 | Characterization data for all compounds and XRD details | S10-S16<br>S30-S36 |
| 5 | EPR experiments | S38-S39 |
| 5 | Reference | S41 |
| 6 | Copies of NMR Spectra | S42-151 |
| 7 | Copies of HRMS Spectra | S152-154 |

**General information:**

All the solvents and starting materials were parched commercial suppliers and used without further purification. Reactions were performed under an atmosphere of open air, $O_2$, or $N_2$ with magnetic stirring. Reactions were monitored by thin layer chromatography (TLC). TLC was performed using E. Merck precoated silica plates (60F-254) with 0.25 mm thickness and visualized using short-wave UV light. Purifications were performed using flash column chromatography with 60−120-mesh silica gel as the stationary phase and a gradient of ethyl acetate in hexanes as the mobile phase. NMR spectra were recorded in $CDCl_3$ and DMSO-$d_6$ with tetramethyl silane as internal standard on a Jeol-400 MHz or Agilent-400 MHz NMR instruments. $^1$H NMR chemical shifts are reported in ppm ($\delta$) relative to the internal standard TMS ($\delta$ 0.00 ppm) and 2.50 for DMSO-$d_6$. $^{13}$C{H} NMR chemical shifts are reported in ppm with respect to solvent resonance as the internal standard ($CDCl_3$ at 77.10 ppm and DMSO-$d_6$ at 39.52). $^1$H NMR data are reported as given here: chemical shift multiplicity such as [singlet (s), doublet (d), triplet (t), quartet (q), pentet (p), multiplet (m), and broad singlet (br s)], coupling constant [Hz] and integration. GC-MS analyses were carried out on an Agilent Technologies 5977A GC equipped with Agilent 7890B MS. High-resolution mass spectra (HRMS) were analyzed on a Jeol JMS-HX 110 ESI spectrometer by the services provided at the National Chung Hsing University. Melting points were measured on X4 melting point apparatus and uncorrected. The X-ray diffraction measurements were carried out using Bruker D8 VENTURE XRD instrument. Known compounds were characterized by comparing their $^1$H NMR and $^{13}$C NMR spectra to the previously reported data. New compounds were characterized by $^1$H NMR, $^{13}$C NMR, and HRMS. The copies of $^1$H and $^{13}$C NMR spectra have been included at the end of the Supporting Information.

**General procedure for the synthesis of compounds 3 and 5:**

In a reaction tube, anilines (0.75 mmol) and $^t$BuONO (1.0 mmol) were added to 2.0 mL of acetonitrile under a nitrogen atmosphere and stirred for 10 minutes. In a separate tube, indole/pyrroles (0.5 mmol), molecular iodine (0.6 mmol), and DABSO (0.6 mmol) were dissolved in 1.0 mL of acetonitrile. The resulting solution was added dropwise to the reaction mixture, and the reaction was stirred at room temperature for 12 hours. The progress of the reaction was monitored by TLC. After completion, the solvent was evaporated under reduced pressure, and the crude product was purified by flash column chromatography using 10-15% ethyl acetate in hexane as the eluent. All synthesized compounds were characterized by detailed spectroscopic analyses, including $^1$H NMR and $^{13}$C NMR for known compounds, and $^1$H NMR, $^{13}$C NMR, and HRMS for

newly synthesized compounds. Additionally, the structures of representative compounds **3ab** and **5bb** was further confirmed by single-crystal X-ray diffraction analysis.

**Large Scale Synthesis**

To evaluate the scalability of our developed protocol, we carried out the reaction on a 5.0 mmol scale. Accordingly, *NH*-indole (5.0 mmol, 0.585 mg, 1.0 equiv.) was reacted with aniline (7.5 mmol, 0.697 mg, 1.5 equiv.), DABSO (6.0 mmol, 1.44g, 1.2 equiv.), $^t$BuONO (10.0 mmol, 1.03g, 2.0 equiv.), and $I_2$ (6.0 mmol, 1.52g, 1.2 equiv.) in 30 mL acetonitrile under the optimized reaction conditions. Upon completion, the desired product **3aa** was isolated in 76% yield.

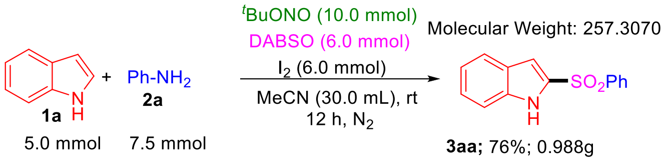


**2-(Phenylsulfonyl)-*1H*-indole (3aa):**[S1]

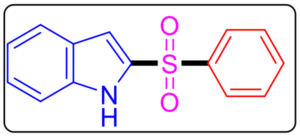

The titled compound was prepared following the general procedure, using indole (**1a**, 0.5 mmol, 59.0 mg, 1.0 equiv.), aniline (**2a**, 0.75 mmol, 70.0 mg, 1.5 equiv.), $^t$BuONO (1.0 mmol, 103.0 mg, 2.0 equiv.), DABSO (0.6 mmol, 144.0 mg, 1.2 equiv.), and $I_2$ (0.6 mmol, 152.0 mg, 1.2 equiv.) in MeCN (3.0 mL). The crude product purified by flash column chromatography ($SiO_2$, 8-10% ethyl acetate in hexanes) to provide **3aa** as white solid, M. P.: 130-131 °C; yield: 104 mg, 83%. $^1$H NMR (400 MHz, DMSO-$d_6$): $\delta$ 12.45 (s, 1H), 8.04 (dd, $J$ = 7.6 & 2.8 Hz, 2H), 7.69-7.59 (m, 4H), 7.46 (dd, $J$ = 8.8 & 2.8 Hz, 1H), 7.32-7.25 (m, 2H), 7.13-7.09 (m, 1H); $^{13}$C{H} NMR (100 MHz, DMSO-$d_6$): $\delta$ 141.3, 137.9, 134.3, 133.7, 120.7, 127.0, 126.0, 125.4, 122.4, 120.9, 112.8, 108.1.

**5-Methyl-2-(phenylsulfonyl)-*1H*-indole (3ba):**[S1]

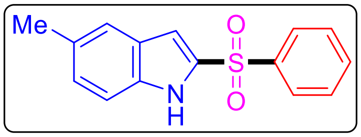

The titled compound was prepared following the general procedure, using 5-methyl-*1H*-indole (**1b**, 0.5 mmol, 66.0 mg, 1.0 equiv.), aniline (**2a**, 0.75 mmol, 70.0 mg, 1.5 equiv.), $^t$BuONO (1.0 mmol, 103.0 mg, 2.0 equiv.), DABSO (0.6 mmol, 144.0 mg, 1.2 equiv.), and $I_2$ (0.6 mmol, 152.0 mg, 1.2 equiv.) in MeCN (3.0 mL). The crude product purified by flash column chromatography ($SiO_2$, 8-

10% ethyl acetate in hexanes) to provide **3ba** as white solid, M. P.: 121-123 °C; yield: 115 mg, 85%. $^{1}$H NMR (400 MHz, $CDCl_3$): $\delta$ 9.70 (s, 1H), 8.01 (dd, $J$ = 8.4 & 2.0 Hz, 2H), 7.48-7.38 (m, 4H), 7.30 (d, $J$ = 8.8 Hz, 1H), 7.15 (t, $J$ = 2.4 Hz, 1H), 7.09 (dd, $J$ = 8.8 & 2.4 Hz, 1H), 2.38 (s, 3H); $^{13}$C{H} NMR (100 MHz, $CDCl_3$): $\delta$ 141.5, 135.9, 133.6, 133.4, 130.9, 129.3, 128.0, 127.2, 127.1, 121.7, 112.2, 108.8, 21.4.

**2-(Phenylsulfonyl)-*1H*-indol-5-ol (3ca):**

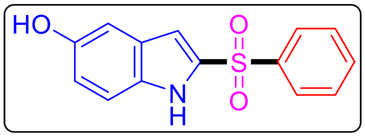


The titled compound was prepared according the general procedure, using *1H*-indol-5-ol (**1c**, 0.5 mmol, 67.0 mg, 1.0 equiv.), aniline (**2a**, 0.75 mmol, 70.0 mg, 1.5 equiv.), $^{t}$BuONO (1.0 mmol, 103.0 mg, 2.0 equiv.), DABSO (0.6 mmol, 144.0 mg, 1.2 equiv.), and $I_2$ (0.6 mmol, 152.0 mg, 1.2 equiv.) in MeCN (3.0 mL). The crude product purified by flash column chromatography ($SiO_2$, 10-15% ethyl acetate in hexanes) to provide **3ca** as white solid, M. P.: 175-176 °C; yield: 116 mg, 85%. $^{1}$H NMR (400 MHz, DMSO-$d_6$): $\delta$ 12.13 (s, 1H), 9.09 (s, 1H), 7.99 (dd, $J$ = 8.4 & 2.0 Hz, 2H), 7.68-7.59 (m, 3H), 7.28 (d, $J$ = 9.2 Hz, 1H), 7.06 (s, 1H), 6.97 (d, $J$ = 2.0 Hz, 1H), 6.88 (dd, $J$ = 9.2 & 2.4 Hz, 1H); $^{13}$C{H} NMR (100 MHz, DMSO-$d_6$): $\delta$ 151.9, 141.6, 134.0, 133.6, 132.6, 129.7, 126.9, 117.0, 113.5, 107.1, 104.7; HRMS (ESI, TOF) m/z, calcd for $C_{14}H_{11}SNO_3$ [M]+ 273.0460, found 273.0462.

**5-Methoxy-2-(phenylsulfonyl)-*1H*-indole (3da):**[S1]

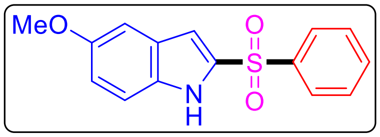


The titled compound was prepared following the general procedure, using 5-methoxy-*1H*-indole (**1d**, 0.5 mmol, 74.0 mg, 1.0 equiv.), aniline (**2a**, 0.75 mmol, 70.0 mg, 1.5 equiv.), $^{t}$BuONO (1.0 mmol, 103.0 mg, 2.0 equiv.), DABSO (0.6 mmol, 144.0 mg, 1.2 equiv.), and $I_2$ (0.6 mmol, 152.0 mg, 1.2 equiv.) in MeCN (3.0 mL). The crude product purified by flash column chromatography ($SiO_2$, 10-15% ethyl acetate in hexanes) to provide **3da** as pale purple solid, M. P.: 121-122 °C; yield: 124 mg, 87%. $^{1}$H NMR (400 MHz, $CDCl_3$): $\delta$ 9.73 (s, 1H), 8.00 (d, $J$ = 7.6 Hz, 2H), 7.51-7.40 (m, 3H), 7.30 (d, $J$ = 8.8 Hz, 1H), 7.15 (t, $J$ = 1.6 Hz, 1H), 7.20 (d, $J$ = 2.4 Hz, 1H), 6.95 (dd,

$J$ = 9.2 & 2.6 Hz, 1H), 3.79 (s, 3H); $^{13}$C{H} NMR (100 MHz, $CDCl_3$): $\delta$ 155.1, 141.5, 135.9, 133.4, 132.8, 129.4, 127.4, 127.2, 117.8, 113.6, 108.8, 102.4, 55.7.

**5-Fluoro-2-(phenylsulfonyl)-*1H*-indole (3ea):**[S1]

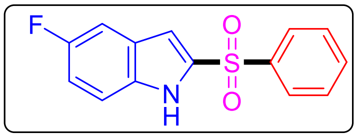


The titled compound was prepared following the general procedure, using 5-fluoro-*1H*-indole (**1e**, 0.5 mmol, 68.0 mg; 1.0 equiv.), aniline (**2a**, 0.75 mmol, 70.0 mg; 1.5 equiv.), $^t$BuONO (1.0 mmol, 103.0 mg, 2.0 equiv.), DABSO (0.6 mmol, 144.0 mg, 1.2 equiv.), and $I_2$ (0.6 mmol, 152.0 mg, 1.2 equiv.) in MeCN (3.0 mL). The crude product purified by flash column chromatography ($SiO_2$, 5-10% ethyl acetate in hexanes) to provide **3ea** as white solid, M. P.: 133-134 °C; yield: 96 mg, 70%. $^1$H NMR (400 MHz, DMSO-$d_6$): $\delta$ 12.62 (s, 1H), 8.04 (dd, $J$ = 8.4 & 1.6 Hz, 2H), 7.65-7.56 (m, 3H), 7.51-7.49 (m, 2H), 7.25 (s, 1H), 7.19 (td, $J$ = 9.2 & 2.4 Hz, 1H); $^{13}$C{H} NMR (100 MHz, DMSO-$d_6$): $\delta$ 157.6 (d, $J$ = 234.0 Hz), 141.1, 136.1, 134.6, 133.8, 129.7, 127.1, 126.2 (d, $J$ = 11.0 Hz), 114.7, 114.4 (d, $J$ = 10.0 Hz), 108.0 (d, $J$ = 5.0 Hz), 106.5 (d, $J$ = 23.0 Hz); $^{19}$F NMR (376 MHz, DMSO-d$_6$) $\delta$ –(122.95-123.03) (m, 1F).

**5-Chloro-2-(phenylsulfonyl)-*1H*-indole (3fa):**[S2]

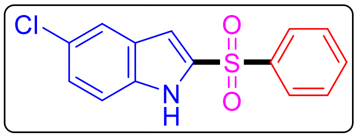


The titled compound was prepared following the general procedure, using 5-chloro-*1H*-indole (**1f**, 0.5 mmol, 76.0 mg, 1.0 equiv.), aniline (**2a**, 0.75 mmol, 70.0 mg, 1.5 equiv.), $^t$BuONO (1.0 mmol, 103.0 mg, 2.0 equiv.), DABSO (0.6 mmol, 144.0 mg, 1.2 equiv.), and $I_2$ (0.6 mmol, 152.0 mg, 1.2 equiv.) in MeCN (3.0 mL). The crude product purified by flash column chromatography ($SiO_2$, 5-10% ethyl acetate in hexanes) to provide **3fa** as white solid, M. P.: 153-155 °C; yield: 109 mg, 75%. $^1$H NMR (400 MHz, $CDCl_3$): $\delta$ 9.93 (s, 1H), 8.02 (dd, 7.6 & 2.0 Hz, 2H), 7.59 -7.56 (m, 2H), 7.47 (t, $J$ = 7.6 Hz, 2H), 7.33 (d, $J$ = 8.8 Hz, 1H), 7.21 (dd, $J$ = 8.8 & 2.0 Hz, 1H), 7.13 (s, 1H); $^{13}$C{H} NMR (100 MHz, $CDCl_3$): $\delta$ 140.9, 135.7, 135.2, 133.8, 129.5, 127.8, 127.3, 127.2, 126.6, 121.7, 113.8, 108.5.

**5-Bromo-2-(phenylsulfonyl)-*1H*-indole (3ga):**[S1]

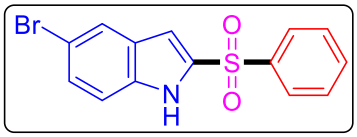


The titled compound was prepared following the general procedure, using 5-bromo-*1H*-indole (**1g**, 0.5 mmol, 98.0 mg, 1.0 equiv.), aniline (**2a**, 0.75 mmol, 70.0 mg, 1.5 equiv.), $^{t}$BuONO (1.0 mmol, 103.0 mg, 2.0 equiv.), DABSO (0.6 mmol, 144.0 mg, 1.2 equiv.), and $I_2$ (0.6 mmol, 152.0 mg, 1.2 equiv.) in MeCN (3.0 mL). The crude product purified by flash column chromatography ($SiO_2$, 5-10% ethyl acetate in hexanes) to provide **3ga** as white solid, M. P.: 153-155 °C; yield: 131 mg, 78%. $^{1}$H NMR (400 MHz, $CDCl_3$): $\delta$ 9.65 (s, 1H), 8.01 (d, $J$ = 8.0 Hz, 2H), 7.78 (d, $J$ = 1.6 Hz, 1H), 7.58 -7.47 (m, 3H), 7.38 (dt, $J$ = 8.8 & 2.0 Hz, 1H), 7.29 (d, $J$ = 8.8 Hz, 1H), 7.12 (dd, $J$ = 2.0 & 1.2 Hz, 1H); $^{13}$C{H} NMR (100 MHz, $CDCl_3$): $\delta$ 141.0, 135.8, 135.2, 133.8, 129.6, 129.2, 128.6, 127.3, 125.0, 114.8, 114.0, 108.3.

**5-Iodo-2-(phenylsulfonyl)-*1H*-indole (3ha)**[S10]

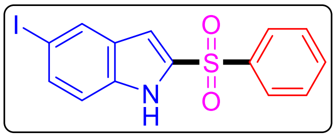


The titled compound was prepared following the general procedure, using 5-iodo-*1H*-indole (**1h**, 0.5 mmol, 122.0 mg, 1.0 equiv.), aniline (**2a**, 0.75 mmol, 70.0 mg, 1.5 equiv.), $^{t}$BuONO (1.0 mmol, 103.0 mg, 2.0 equiv.), DABSO (0.6 mmol, 144.0 mg, 1.2 equiv.), and $I_2$ (0.6 mmol, 152.0 mg, 1.2 equiv.) in MeCN (3.0 mL). The crude product purified by flash column chromatography ($SiO_2$, 5-10% ethyl acetate in hexanes) to provide **3ha** as white solid, M. P.: 133-134 °C; yield: 129 mg, 67%. $^{1}$H NMR (400 MHz, $CDCl_3$): $\delta$ 9.45 (s, 1H), 9.93 (dd, $J$ = 7.6 & 2.0 Hz, 3H), 7.53-7.41 (m, 4H), 7.12 (d, J = 8.8 Hz, 1H), 7.03 (d, $J$ = 1.6 Hz, 1H); $^{13}$C{H} NMR (100 MHz, $CDCl_3$): $\delta$ 141.0, 136.2, 134.7, 134.5, 133.8, 131.4, 129.6, 129.4, 127.3, 114.4, 108.0, 85.0.

**6-Chloro-2-(phenylsulfonyl)-*1H*-indole (3ia):**[S2]

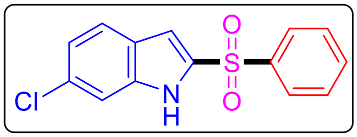


The titled compound was prepared following the general procedure, using 6-chloro-*1H*-indole (**1i**, 0.5 mmol, 76.0 mg, 1.0 equiv.), aniline (**2a**, 0.75 mmol, 70.0 mg, 1.5 equiv.), $^{t}$BuONO (1.0 mmol, 103.0 mg, 2.0 equiv.), DABSO (0.6 mmol, 144.0 mg, 1.2 equiv.), and $I_2$ (0.6 mmol, 152.0 mg, 1.2

equiv.) in MeCN (3.0 mL). The crude product purified by flash column chromatography ($SiO_2$, 5-10% ethyl acetate in hexanes) to provide **3ia** as pale pink solid, M. P.: 165-167 °C; yield: 105 mg, 72%. $^1$H NMR (400 MHz, DMSO-$d_6$): $\delta$ 12.63 (s, 1H), 8.05-8.02 (m, 2H), 7.69-7.59 (m, 4H), 7.49-7.48 (m, 1H), 7.27 (d, $J$ = 1.2 Hz, 1H), 7.12 (dd, $J$ = 8.8 & 2.0 Hz, 1H); $^{13}$C{H} NMR (100 MHz, DMSO-$d_6$): $\delta$ 141.0, 138.1, 135.4, 133.9, 130.1, 129.7, 127.1, 124.8, 123.9, 121.6, 112.3, 108.2.

**1-Methyl-2-(phenylsulfonyl)-*1H*-indole (3ja)[S2]:**

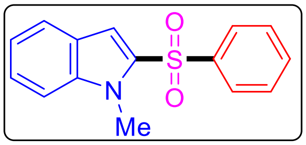


The titled compound was prepared following the general procedure, using 1-methyl-*1H*-indole (**1j**, 0.5 mmol, 66.0 mg, 1.0 equiv.), aniline (**2a**, 0.75 mmol, 70.0 mg, 1.5 equiv.), $^t$BuONO (1.0 mmol, 103.0 mg, 2.0 equiv.), DABSO (0.6 mmol, 144.0 mg, 1.2 equiv.), and $I_2$ (0.6 mmol, 152.0 mg, 1.2 equiv.) in MeCN (3.0 mL). The crude product purified by flash column chromatography ($SiO_2$, 5-10% ethyl acetate in hexanes) to provide **3ja** as white solid, M. P.: 118-120 °C; yield: 112 mg, 82%. $^1$H NMR (400 MHz, DMSO-$d_6$): $\delta$ 8.00-7.98 (m, 2H), 7.75-7.69 (m, 2H), 7.66-7.62 (m, 2H), 7.53 (dd, $J$ = 8.8 & 1.2 Hz, 1H), 7.42 (d, $J$ = 0.8 Hz, 1H), 7.39-7.35 (m, 1H), 7.19-7.15 (m, 1H); $^{13}$C{H} NMR (100 MHz, DMSO-$d_6$): $\delta$ 140.6, 139.2, 134.5, 133.9, 129.9, 127.3, 125.7, 124.6, 122.6, 121.1, 111.1, 110.2, 30.9.

***Tert*-butyl 2-(phenylsulfonyl)-*1H*-indole-1-carboxylate (3ka)[S9]**

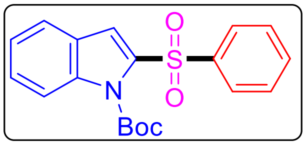


The titled compound was prepared following the general procedure, using *tert*-butyl *1H*-indole-1-carboxylate (**1k**, 0.5 mmol, 109.0 mg, 1.0 equiv.), aniline (**2a**, 0.75 mmol, 70.0 mg, 1.5 equiv.), $^t$BuONO (1.0 mmol, 103.0 mg, 2.0 equiv.), DABSO (0.6 mmol, 144.0 mg, 1.2 equiv.), and $I_2$ (0.6 mmol, 152.0 mg, 1.2 equiv.) in MeCN (3.0 mL). The crude product purified by flash column chromatography ($SiO_2$, 2-5% ethyl acetate in hexanes) to provide **3ka** as purple liquid, yield: 135 mg, 75%. $^1$H NMR (400 MHz, $CDCl_3$): $\delta$ 7.95 (d, $J$ = 8.4 Hz, 1H), 7.91 (d, $J$ = 7.2 Hz, 2H), 7.57-7.52 (m, 2H), 7.45 (t, $J$ = 8.0 Hz, 2H), 7.38 (t, $J$ = 9.2 Hz, 1H), 7.24-7.18 (m, 2H), 1.52 (s, 9H);

$^{13}C\{H\}$ NMR (100 MHz, $CDCl_3$): $\delta$ 148.1, 141.3, 137.8, 136.8, 133.2, 128.7, 128.2, 128.0, 126.2, 123.8, 122.8, 119.7, 115.9, 86.2., 27.9.

**2-(Phenylsulfonyl)-1-tosyl-*1H*-indole (3la)**

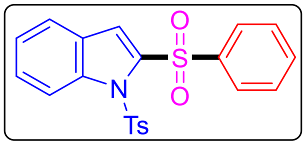


The titled compound was prepared following the general procedure, using 1-tosyl-*1H*-indole (**1l**, 0.5 mmol, 135.0 mg, 1.0 equiv.), aniline (**2a**, 0.75 mmol, 70.0 mg, 1.5 equiv.), $^t$BuONO (1.0 mmol, 103.0 mg, 2.0 equiv.), DABSO (0.6 mmol, 144.0 mg, 1.2 equiv.), and $I_2$ (0.6 mmol, 152.0 mg, 1.2 equiv.) in MeCN (3.0 mL). The crude product purified by flash column chromatography ($SiO_2$, 10-15% ethyl acetate in hexanes) to provide **3la** as color less liquid, yield: 175 mg, 85%. $^1$H NMR (400 MHz, DMSO-$d_6$): $\delta$ 8.12 (d, $J$ = 8.8 Hz, 1H), 7.99 (dd, $J$ = 8.8 & 1.6 Hz, 2H), 7.95 (s, 1H), 7.83-7.75 (m, 2H), 7.73-7.66 (m, 4H), 7.60-7.56 (m, 1H), 7.39 (t, $J$ = 7.6 Hz, 1H), 7.31 (d, $J$ = 8.4 Hz, 2H), 2.27 (s, 3H); $^{13}C\{H\}$ NMR (100 MHz, DMSO-$d_6$): $\delta$ 146.2, 140.1, 138.1, 136.9, 134.1, 133.5, 130.1, 129.3, 129.2, 128.1, 127.2, 126.4, 124.9, 123.9, 123.8, 114.9, 21.1. HRMS (ESI, TOF) m/z, calcd for $C_{21}H_{17}O_4NS_2$ $[M+H]^+$ 412.0672, found 412.0668.

**Phenyl(2-(phenylsulfonyl)-*1H*-indol-1-yl)methanone (3ma)[S1]**

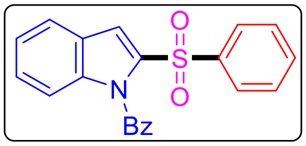


The titled compound was prepared following the general procedure, using (*1H*-indol-1-yl)(phenyl)methanone (**1m**, 0.5 mmol, 110.0 mg, 1.0 equiv.), aniline (**2a**, 0.75 mmol, 70.0 mg, 1.5 equiv.), $^t$BuONO (1.0 mmol, 103.0 mg, 2.0 equiv.), DABSO (0.6 mmol, 144.0 mg, 1.2 equiv.), and $I_2$ (0.6 mmol, 152.0 mg, 1.2 equiv.) in MeCN (3.0 mL). The crude product purified by flash column chromatography ($SiO_2$, 10-15% ethyl acetate in hexanes) to provide **3ma** as white solid, yield: 160 mg, 88%. $^1$H NMR (400 MHz, DMSO-$d_6$): $\delta$ 7.86 (d, $J$ = 7.2 Hz, 1H), 7.79 (dd, $J$ = 8.0 & 1.2 Hz, 2H), 7.59-7.55 (m, 2H), 7.47-7.44 (m, 2H), 7.35-7.26 (m, 2H), 7.19-7.15 (m, 1H), 7.12-7.08 (m, 3H), 6.76-6.73 (m, 2H), 5.66 (s, 2H); $^{13}C\{H\}$ NMR (100 MHz, DMSO-$d_6$): $\delta$ 140.3, 138.9, 136.9, 134.1, 133.8, 129.6, 128.3, 127.3, 127.1, 126.1, 125.8, 124.9, 122.9, 121.5, 111.9, 111.3, 47.2.

**2-Tosyl-*1H*-indole (3ab):**[S1]

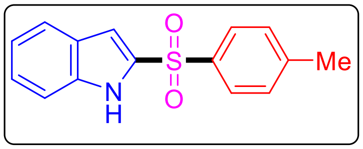


The titled compound was prepared following the general procedure, using indole (**1a**, 0.5 mmol, 59.0 mg, 1.0 equiv.), *p*-toluidine (**2b**, 0.75 mmol, 80.0 mg, 1.5 equiv.), $^t$BuONO (1.0 mmol, 103.0 mg, 2.0 equiv.), DABSO (0.6 mmol, 144.0 mg, 1.2 equiv.), and $I_2$ (0.6 mmol, 152.0 mg, 1.2 equiv.) in MeCN (3.0 mL). The crude product purified by flash column chromatography ($SiO_2$, 8-10% ethyl acetate in hexanes) to provide **3ab** as pale pink solid, M. P.: 140-141 $^oC$; yield: 118 mg, 87%. $^1H$ NMR (400 MHz, $CDCl_3$): $\delta$ 9.33 (s, 1H), 7.90 (dd, $J$ = 8.4 & 2.0 Hz, 2H), 7.65 (d, $J$ = 8.4 Hz, 1H), 7.41 (d, $J$ = 8.4 Hz, 1H), 7.33-7.25 (m, 3H), 7.19-7.13 (m, 2H), 2.37 (s, 3H); $^{13}C\{H\}$ NMR (100 MHz, $CDCl_3$): $\delta$ 144.6, 138.6, 137.2, 134.5, 130.1, 127.4, 127.1, 125.9, 122.7, 121.6, 112.4, 108.9, 21.7. **Crystal data** for **3ab (CCDC 2499712)**: After flash column chromatographic separation, the compound 3ab was dissolved in 20% EA/Hexanes and kept at room temperature for overnight which provided the crystal to perform the single-crystal data analysis.

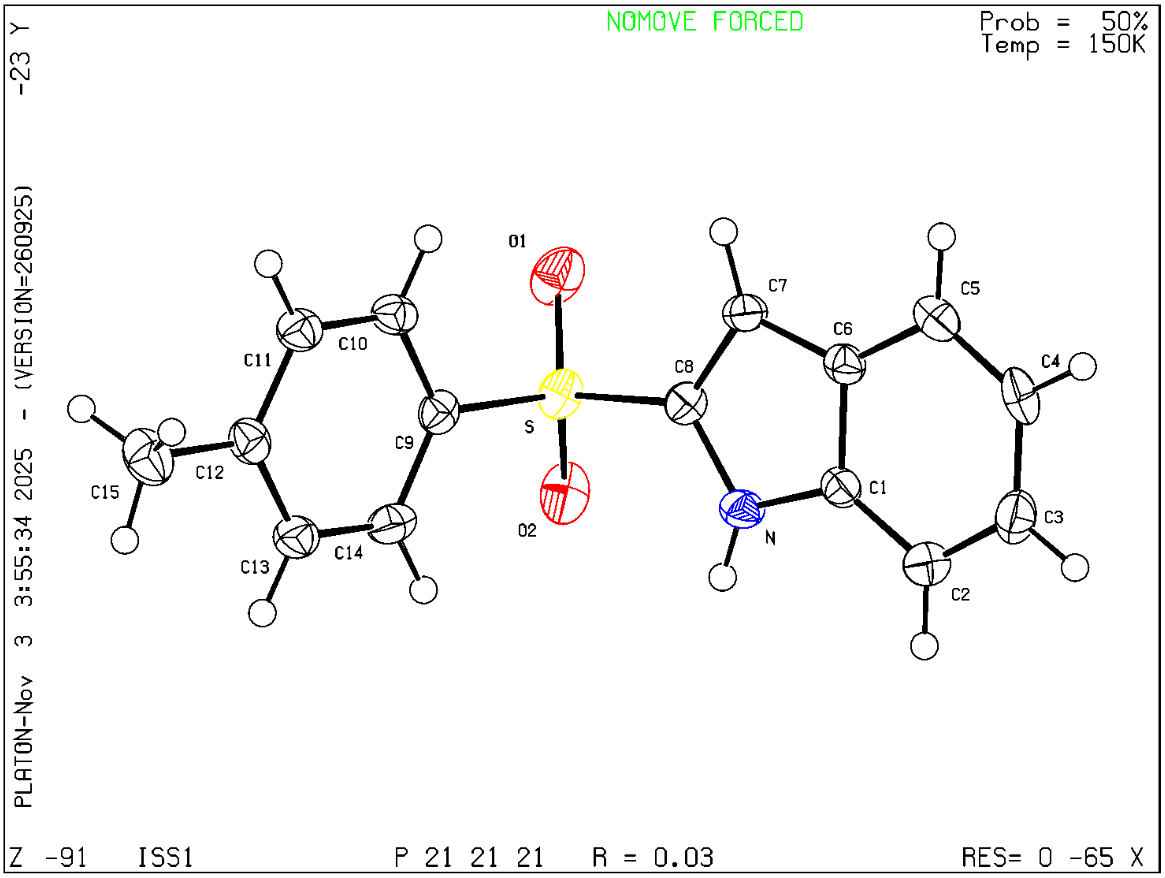


**Table 1. Crystal data and structure refinement for 3ab**

| | | |
|---|---|---|
| Identification code | **3ab** | |
| Empirical formula | $C_{15}H_{13}NO_2S$ | |
| Formula weight | 271.32 | |
| Temperature | 150(2) K | |
| Wavelength | 0.71073 Å | |
| Crystal system | Orthorhombic | |
| Space group | $P2_12_12_1$ | |
| Unit cell dimensions | a = 5.7596(2) Å | α= 90°. |
| | b = 7.9852(4) Å | β= 90°. |
| | c = 27.9349(12) Å | γ = 90°. |
| Volume | 1284.77(10) $Å^3$ | |
| Z | 4 | |
| Density (calculated) | 1.403 $Mg/m^3$ | |
| Absorption coefficient | 0.248 $mm^{-1}$ | |

| | |
|---|---|
| F(000) | 568 |
| Crystal size | 0.380 x 0.190 x 0.120 $mm^3$ |
| Theta range for data collection | 3.361 to 27.523°. |
| Index ranges | -7<=h<=7, -10<=k<=10, -36<=l<=36 |
| Reflections collected | 31321 |
| Independent reflections | 2948 [R(int) = 0.0447] |
| Completeness to theta = 25.242° | 98.6 % |
| Absorption correction | Semi-empirical from equivalents |
| Max. and min. transmission | 0.7456 and 0.6427 |
| Refinement method | Full-matrix least-squares on $F^2$ |
| Data / restraints / parameters | 2948 / 0 / 176 |
| Goodness-of-fit on $F^2$ | 1.135 |
| Final R indices [I>2sigma(I)] | R1 = 0.0349, wR2 = 0.0946 |
| R indices (all data) | R1 = 0.0354, wR2 = 0.0950 |
| Absolute structure parameter | 0.038(15) |
| Extinction coefficient | n/a |
| Largest diff. peak and hole | 0.771 and -0.303 e.$Å^{-3}$ |

**Table 2. Atomic coordinates ( x $10^4$) and equivalent isotropic displacement parameters ($Å^2$x $10^3$) For 3ab. U(eq) is defined as one third of the trace of the orthogonalized $U^{ij}$ tensor.**

| | x | y | z | U(eq) |
|---|---|---|---|---|
| S | 9406(1) | 4886(1) | 1205(1) | 27(1) |
| O(1) | 11906(3) | 4935(3) | 1204(1) | 40(1) |
| O(2) | 8212(4) | 3431(2) | 1028(1) | 36(1) |
| N | 6461(4) | 4508(3) | 1961(1) | 25(1) |
| C(1) | 6323(4) | 4819(3) | 2444(1) | 22(1) |
| C(2) | 4594(4) | 4420(3) | 2772(1) | 30(1) |
| C(3) | 4907(5) | 4936(4) | 3239(1) | 33(1) |
| C(4) | 6903(5) | 5810(4) | 3382(1) | 33(1) |
| C(5) | 8653(5) | 6176(3) | 3060(1) | 29(1) |
| C(6) | 8377(4) | 5683(3) | 2581(1) | 23(1) |
| C(7) | 9756(4) | 5872(3) | 2163(1) | 24(1) |
| C(8) | 8537(4) | 5142(3) | 1798(1) | 24(1) |
| C(9) | 8377(4) | 6650(3) | 893(1) | 23(1) |

| | | | | |
|---|---|---|---|---|
| C(10) | 9699(4) | 8104(3) | 890(1) | 25(1) |
| C(11) | 8854(4) | 9503(3) | 649(1) | 26(1) |
| C(12) | 6711(4) | 9465(3) | 416(1) | 26(1) |
| C(13) | 5432(5) | 7984(3) | 424(1) | 28(1) |
| C(14) | 6242(4) | 6570(3) | 666(1) | 28(1) |
| C(15) | 5809(6) | 10981(3) | 160(1) | 38(1) |

**Table 3. Bond lengths [Å] and angles [°] for 3ab.**

| | |
|---|---|
| S-O(1) | 1.4407(19) |
| S-O(2) | 1.437(2) |
| S-C(8) | 1.742(2) |
| S-C(9) | 1.760(2) |
| N-C(8) | 1.376(3) |
| N-C(1) | 1.376(3) |
| N-H(0A) | 0.86(4) |
| C(1)-C(2) | 1.389(3) |
| C(1)-C(6) | 1.422(3) |
| C(2)-C(3) | 1.381(4) |
| C(2)-H(2B) | 0.9500 |
| C(3)-C(4) | 1.402(4) |
| C(3)-H(3A) | 0.9500 |
| C(4)-C(5) | 1.382(4) |
| C(4)-H(4A) | 0.9500 |
| C(5)-C(6) | 1.403(3) |
| C(5)-H(5A) | 0.9500 |
| C(6)-C(7) | 1.421(3) |
| C(7)-C(8) | 1.368(3) |
| C(7)-H(7A) | 0.9500 |
| C(9)-C(14) | 1.386(3) |
| C(9)-C(10) | 1.388(3) |
| C(10)-C(11) | 1.392(3) |
| C(10)-H(10A) | 0.9500 |
| C(11)-C(12) | 1.397(3) |
| C(11)-H(11A) | 0.9500 |
| C(12)-C(13) | 1.393(3) |

| | |
|---|---|
| C(12)-C(15) | 1.498(4) |
| C(13)-C(14) | 1.395(4) |
| C(13)-H(13A) | 0.9500 |
| C(14)-H(14A) | 0.9500 |
| C(15)-H(15A) | 0.9800 |
| C(15)-H(15B) | 0.9800 |
| C(15)-H(15C) | 0.9800 |

| | |
|---|---|
| O(1)-S-O(2) | 119.98(13) |
| O(1)-S-C(8) | 106.66(12) |
| O(2)-S-C(8) | 106.51(11) |
| O(1)-S-C(9) | 108.24(12) |
| O(2)-S-C(9) | 108.40(12) |
| C(8)-S-C(9) | 106.27(11) |
| C(8)-N-C(1) | 107.96(19) |
| C(8)-N-H(0A) | 128(2) |
| C(1)-N-H(0A) | 124(2) |
| N-C(1)-C(2) | 130.3(2) |
| N-C(1)-C(6) | 107.6(2) |
| C(2)-C(1)-C(6) | 122.1(2) |
| C(3)-C(2)-C(1) | 117.4(2) |
| C(3)-C(2)-H(2B) | 121.3 |
| C(1)-C(2)-H(2B) | 121.3 |
| C(2)-C(3)-C(4) | 121.5(2) |
| C(2)-C(3)-H(3A) | 119.2 |
| C(4)-C(3)-H(3A) | 119.2 |
| C(5)-C(4)-C(3) | 121.3(2) |
| C(5)-C(4)-H(4A) | 119.4 |
| C(3)-C(4)-H(4A) | 119.4 |
| C(4)-C(5)-C(6) | 118.6(2) |
| C(4)-C(5)-H(5A) | 120.7 |
| C(6)-C(5)-H(5A) | 120.7 |
| C(5)-C(6)-C(7) | 133.7(2) |
| C(5)-C(6)-C(1) | 119.1(2) |
| C(7)-C(6)-C(1) | 107.2(2) |
| C(8)-C(7)-C(6) | 106.3(2) |

| | |
|---|---|
| C(8)-C(7)-H(7A) | 126.9 |
| C(6)-C(7)-H(7A) | 126.9 |
| N-C(8)-C(7) | 110.9(2) |
| N-C(8)-S | 121.37(17) |
| C(7)-C(8)-S | 127.65(19) |
| C(14)-C(9)-C(10) | 121.5(2) |
| C(14)-C(9)-S | 119.27(19) |
| C(10)-C(9)-S | 119.20(19) |
| C(9)-C(10)-C(11) | 118.9(2) |
| C(9)-C(10)-H(10A) | 120.6 |
| C(11)-C(10)-H(10A) | 120.6 |
| C(12)-C(11)-C(10) | 121.1(2) |
| C(12)-C(11)-H(11A) | 119.4 |
| C(10)-C(11)-H(11A) | 119.4 |
| C(11)-C(12)-C(13) | 118.6(2) |
| C(11)-C(12)-C(15) | 120.7(2) |
| C(13)-C(12)-C(15) | 120.7(2) |
| C(14)-C(13)-C(12) | 121.2(2) |
| C(14)-C(13)-H(13A) | 119.4 |
| C(12)-C(13)-H(13A) | 119.4 |
| C(9)-C(14)-C(13) | 118.7(2) |
| C(9)-C(14)-H(14A) | 120.6 |
| C(13)-C(14)-H(14A) | 120.6 |
| C(12)-C(15)-H(15A) | 109.5 |
| C(12)-C(15)-H(15B) | 109.5 |
| H(15A)-C(15)-H(15B) | 109.5 |
| C(12)-C(15)-H(15C) | 109.5 |
| H(15A)-C(15)-H(15C) | 109.5 |
| H(15B)-C(15)-H(15C) | 109.5 |

Symmetry transformations used to generate equivalent atoms:

**Table 4. Anisotropic displacement parameters ($Å^2$x $10^3$) for 3ab. The anisotropic displacement factor exponent takes the form: $-2\pi^2[ h^2 a^{*2}U^{11} + ... + 2 h k a^* b^* U^{12}]$**

| | $U^{11}$ | $U^{22}$ | $U^{33}$ | $U^{23}$ | $U^{13}$ | $U^{12}$ |
|---|---|---|---|---|---|---|

| | | | | | | |
|---|---|---|---|---|---|---|
| S | 31(1) | 24(1) | 26(1) | -1(1) | 6(1) | 3(1) |
| O(1) | 31(1) | 42(1) | 47(1) | 9(1) | 13(1) | 11(1) |
| O(2) | 57(1) | 24(1) | 29(1) | -6(1) | 5(1) | -2(1) |
| N | 24(1) | 28(1) | 23(1) | -4(1) | -2(1) | -6(1) |
| C(1) | 23(1) | 22(1) | 22(1) | 1(1) | -4(1) | 0(1) |
| C(2) | 26(1) | 33(1) | 32(1) | 2(1) | -1(1) | -3(1) |
| C(3) | 33(1) | 39(1) | 27(1) | 9(1) | 6(1) | 4(1) |
| C(4) | 42(1) | 39(1) | 17(1) | 2(1) | -5(1) | 7(1) |
| C(5) | 30(1) | 30(1) | 27(1) | -1(1) | -8(1) | 1(1) |
| C(6) | 22(1) | 20(1) | 26(1) | 2(1) | -4(1) | 2(1) |
| C(7) | 20(1) | 24(1) | 29(1) | 1(1) | -2(1) | -3(1) |
| C(8) | 24(1) | 23(1) | 24(1) | -1(1) | 2(1) | 0(1) |
| C(9) | 26(1) | 24(1) | 20(1) | -3(1) | 4(1) | -1(1) |
| C(10) | 23(1) | 28(1) | 24(1) | -6(1) | 0(1) | -2(1) |
| C(11) | 28(1) | 27(1) | 25(1) | -5(1) | 0(1) | -3(1) |
| C(12) | 29(1) | 29(1) | 20(1) | -3(1) | -1(1) | 1(1) |
| C(13) | 26(1) | 35(1) | 24(1) | -3(1) | 1(1) | -4(1) |
| C(14) | 25(1) | 30(1) | 29(1) | -5(1) | 4(1) | -7(1) |
| C(15) | 47(2) | 32(1) | 36(1) | 2(1) | -10(1) | 3(1) |

**Table 5. Hydrogen coordinates ( x $10^4$) and isotropic displacement parameters (Å$^2$x 10 $^3$) 3ab**

| | x | y | z | U(eq) |
|---|---|---|---|---|
| H(0A) | 5430(70) | 3980(50) | 1801(13) | 47(10) |
| H(2B) | 3248 | 3817 | 2678 | 37 |
| H(3A) | 3743 | 4693 | 3470 | 40 |
| H(4A) | 7056 | 6156 | 3705 | 39 |
| H(5A) | 10013 | 6749 | 3160 | 35 |
| H(7A) | 11230 | 6400 | 2141 | 29 |
| H(10A) | 11155 | 8142 | 1049 | 30 |
| H(11A) | 9751 | 10502 | 644 | 32 |
| H(13A) | 3981 | 7937 | 263 | 34 |
| H(14A) | 5347 | 5571 | 674 | 34 |

| | | | | |
|---|---|---|---|---|
| H(15A) | 4294 | 10726 | 19 | 58 |
| H(15B) | 5646 | 11907 | 388 | 58 |
| H(15C) | 6899 | 11301 | -93 | 58 |

**5-Methyl-2-tosyl-*1H*-indole (3bb):**[S2]

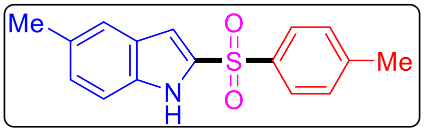


The titled compound was prepared following the general procedure, using 5-methyl-*1H*-indole (**1b**, 0.5 mmol, 66.0 mg, 1.0 equiv.), *p*-toluidine (**2b**, 0.75 mmol, 80.0 mg, 1.5 equiv.), $^t$BuONO (1.0 mmol, 103.0 mg, 2.0 equiv.), DABSO (0.6 mmol, 144.0 mg, 1.2 equiv.), and $I_2$ (0.6 mmol, 152.0 mg, 1.2 equiv.) in MeCN (3.0 mL). The crude product purified by flash column chromatography ($SiO_2$, 8-10% ethyl acetate in hexanes) to provide **3bb** as pale pink solid, M. P.: 149-150 $^o$C; yield: 127 mg, 89%. $^1$H NMR (400 MHz, $CDCl_3$): $\delta$ 9.74 (s, 1H), 7.89 (d, *J* = 8.0 Hz, 2H), 7.39 (s, 1H), 7.29 (d, *J* = 8.4 Hz, 1H), 7.19 (d, *J* = 8.0 Hz, 2H), 7.12-7.07 (m, 2H), 2.37 (s, 3H), 2.30 (s, 3H); $^{13}$C{H} NMR (100 MHz, $CDCl_3$): $\delta$ 144.4, 138.6, 135.9, 134.0, 130.8, 129.9, 127.8, 127.2, 127.1, 121.7, 112.2, 108.4, 21.5, 21.4.

**2-Tosyl-*1H*-indol-5-ol (3cb):**[S3]

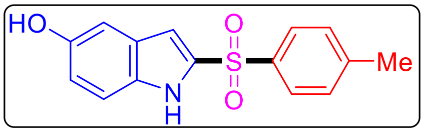


The titled compound was prepared following the general procedure, using *1H*-indol-5-ol (**1c**, 0.5 mmol, 67.0 mg, 1.0 equiv.), *p*-toluidine (**2b**, 0.75 mmol, 80.0 mg, 1.5 equiv.), $^t$BuONO (1.0 mmol, 103.0 mg, 2.0 equiv.), DABSO (0.6 mmol, 144.0 mg, 1.2 equiv.), and $I_2$ (0.6 mmol, 152.0 mg, 1.2 equiv.) in MeCN (3.0 mL). The crude product purified by flash column chromatography ($SiO_2$, 8-10% ethyl acetate in hexanes) to provide **3cb** as off white solid, M. P.: 180-181 $^o$C; yield: 131 mg, 91%. $^1$H NMR (400 MHz, DMSO-$d_6$): $\delta$ 12.06 (s, 1H), 9.08 (d, *J* = 5.7 Hz, 1H), 7.89-7.85 (m, 2H), 7.40 (t, *J* = 7.2 Hz, 2H), 7.27 (t, *J* = 8.4 Hz, 1H), 6.99 (d, *J* = 8.0 Hz, 1H), 6.95-6.84 (m, 2H), 2.32 (s, 3H); $^{13}$C{H} NMR (100 MHz, DMSO-$d_6$): $\delta$ 151.9, 144.2, 138.7, 134.5, 132.5, 130.1, 127.0, 126.9, 116.9, 113.4, 106.7, 104.7, 21.0.

**5-Methoxy-2-tosyl-1H-indole (3db):**[S1]

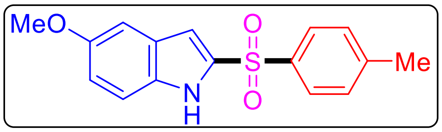


The titled compound was prepared following the general procedure, using 5-methoxy-*1H*-indole (**1d**, 0.5 mmol, 74.0 mg, 1.0 equiv.), *p*-toluidine (**2b**, 0.75 mmol, 80.0 mg, 1.5 equiv.), $^t$BuONO (1.0 mmol, 103.0 mg, 2.0 equiv.), DABSO (0.6 mmol, 144.0 mg, 1.2 equiv.), and $I_2$ (0.6 mmol, 152.0 mg, 1.2 equiv.) in MeCN (3.0 mL). The crude product purified by flash column chromatography ($SiO_2$, 8-10% ethyl acetate in hexanes) to provide **3db** as white solid, M. P.: 165-166 $^o$C; yield: 138 mg, 92%. $^1$H NMR (400 MHz, $CDCl_3$): $\delta$ 9.53 (s, 1H), 7.88 (d, *J* = 7.6 Hz, 2H), 7.29 (d, *J* = 9.2 Hz, 1H), 7.24 (d, *J* = 8.0 Hz, 2H), 7.10 (s, 1H), 7.02 (d, *J* = 2.4 Hz, 1H), 6.96 (dd, *J* = 9.2 & 2.4 Hz, 1H), 3.80 (s, 3H), 2.35 (s, 3H); $^{13}$C{H} NMR (100 MHz, $CDCl_3$): $\delta$ 155.1, 144.5, 138.6, 134.4, 132.6, 130.0, 127.5, 127.3, 117.6, 113.5, 108.4, 102.4, 55.7, 21.6.

**5-Fluoro-2-tosyl-*1H*-indole (3eb):**[S1]

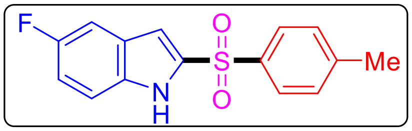


The titled compound was prepared following the general procedure, using 5-fluoro-*1H*-indole (**1e**, 0.5 mmol, 68.0 mg, 1.0 equiv.), *p*-toluidine (**2b**, 0.75 mmol, 80.0 mg, 1.5 equiv.), $^t$BuONO (1.0 mmol, 103.0 mg, 2.0 equiv.), DABSO (0.6 mmol, 144.0 mg, 1.2 equiv.), and $I_2$ (0.6 mmol, 152.0 mg, 1.2 equiv.) in MeCN (3.0 mL). The crude product purified by flash column chromatography ($SiO_2$, 8-10% ethyl acetate in hexanes) to provide **3eb** as pink solid, M. P.: 144 $^o$C; yield: 91 mg, 63%. $^1$H NMR (400 MHz, DMSO-$d_6$): $\delta$ 12.51 (s, 1H), 7.89 (dt, *J* = 6.4 & 1.6 Hz, 2H), 7.47-7.40 (m, 4H), 7.19-7.14 (m, 2H), 2.33 (s, 3H); $^{13}$C{H} NMR (100 MHz, DMSO-$d_6$): $\delta$ 157.4 (d, *J* = 233.0 Hz), 144.6, 138.2, 136.4, 134.5, 130.2, 127.2, 126.2 (d, *J* = 10.0 Hz), 114.5, 114.2 (d, *J* = 8.0 Hz), 107.5, 106.4 (d, *J* = 24.0 Hz), 21.0; $^{19}$F NMR (376 MHz, DMSO-$d_6$) $\delta$ –(123.03-123.09) (m, 1F).

**5-Chloro-2-tosyl-*1H*-indole (3fb):**[S2]

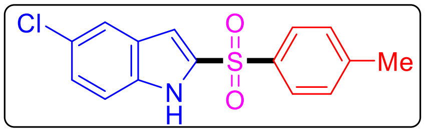


The titled compound was prepared following the general procedure, using 5-chloro-*1H*-indole (**1f**, 0.5 mmol, 76.0 mg, 1.0 equiv.), *p*-toluidine (**2b**, 0.75 mmol, 80.0 mg, 1.5 equiv.), $^t$BuONO (1.0

mmol, 103.0 mg, 2.0 equiv.), DABSO (0.6 mmol, 144.0 mg, 1.2 equiv.), and $I_2$ (0.6 mmol, 152.0 mg, 1.2 equiv.) in MeCN (3.0 mL). The crude product purified by flash column chromatography ($SiO_2$, 8-10% ethyl acetate in hexanes) to provide **3fb** as pink solid, M. P.: 178-180 $^oC$; yield: 107 mg, 70%. $^1H$ NMR (400 MHz, $CDCl_3$): $\delta$ 9.58 (s, 1H), 7.89 (d, $J$ = 8.0 Hz, 2H), 7.61 (d, $J$ = 2.0 Hz, 1H), 7.35-7.23 (m, 4H), 7.09 (d, $J$ = 2.0 Hz, 1H), 2.38 (s, 3H); $^{13}C\{H\}$ NMR (100 MHz, $CDCl_3$): $\delta$ 144.9, 138.1, 135.8, 135.5, 130.2, 127.9, 127.4, 127.2, 126.5, 121.8, 113.7, 108.1, 21.7.

**5-Bromo-2-tosyl-*1H*-indole (3gb):**[S1]

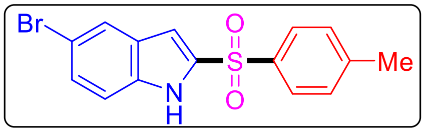


The titled compound was prepared following the general procedure, using 5-bromo-*1H*-indole (**1g**, 0.5 mmol, 98.0 mg, 1.0 equiv.), *p*-toluidine (**2b**, 0.75 mmol, 80.0 mg, 1.5 equiv.), $^t$BuONO (1.0 mmol, 103.0 mg, 2.0 equiv.), DABSO (0.6 mmol, 144.0 mg, 1.2 equiv.), and $I_2$ (0.6 mmol, 152.0 mg, 1.2 equiv.) in MeCN (3.0 mL). The crude product purified by flash column chromatography ($SiO_2$, 8-10% ethyl acetate in hexanes) to provide **3gb** as off white solid, M. P.: 202-204 $^oC$; yield: 137 mg, 78%. $^1H$ NMR (400 MHz, DMSO-$d_6$): $\delta$ 12.58 (s, 1H), 7.98-7.87 (m, 3H), 7.44 (dd, $J$ = 8.4 & 0.8 Hz, 2H), 7.40 (t, $J$ = 1.2 Hz, 2H), 7.16 (s, 1H), 2.36 (s, 3H); $^{13}C\{H\}$ NMR (100 MHz, DMSO-$d_6$): $\delta$ 144.6, 138.0, 136.3, 136.1, 130.1, 127.9, 127.7, 127.2, 124.5, 114.8, 113.2, 107.1, 21.0.

**6-Chloro-2-tosyl-*1H*-indole (3ib):**[S2]

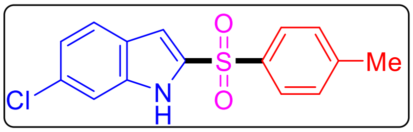


The titled compound was prepared following the general procedure, using 6-chloro-*1H*-indole (**1i**, 0.5 mmol, 76.0 mg, 1.0 equiv.), *p*-toluidine (**2b**, 0.75 mmol, 80.0 mg, 1.5 equiv.), $^t$BuONO (1.0 mmol, 103.0 mg, 2.0 equiv.), DABSO (0.6 mmol, 144.0 mg, 1.2 equiv.), and $I_2$ (0.6 mmol, 152.0 mg, 1.2 equiv.) in MeCN (3.0 mL). The crude product purified by flash column chromatography ($SiO_2$, 8-10% ethyl acetate in hexanes) to provide **3ib** as pink solid, M. P.: 191-192 $^oC$; yield: 94 mg, 60%. $^1H$ NMR (400 MHz, DMSO-$d_6$): $\delta$ 12.55 (s, 1H), 7.89 (dd, $J$ = 6.4 & 2.0, 2H), 7.69 (d, $J$ = 8.8 & 0.7 Hz, 1H), 7.45-7.41 (m, 3H), 7.22 (d, $J$ = 0.8 Hz, 1H), 7.13 (dd, $J$ = 8.4 & 2.0 Hz, 1H), 2.34 (s, 3H); $^{13}C\{H\}$ NMR (100 MHz, DMSO-$d_6$): $\delta$ 144.6, 138.1, 137.9, 135.8, 130.2, 129.8, 127.2, 124.8, 123.9, 121.5, 112.2, 107.8, 21.1.

**2-((4-Fluorophenyl)sulfonyl)-*1H*-indole (3ac):**[S1]

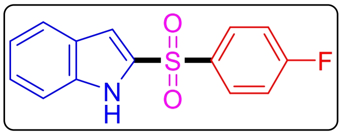

The titled compound was prepared following the general procedure, using indole (**1a**, 0.5 mmol, 59.0 mg, 1.0 equiv.), 4-fluoroaniline (**2c**, 0.75 mmol, 84.0 mg, 1.5 equiv.), $^{t}$BuONO (1.0 mmol, 103.0 mg, 2.0 equiv.), DABSO (0.6 mmol, 144.0 mg, 1.2 equiv.), and $I_2$ (0.6 mmol, 152.0 mg, 1.2 equiv.) in MeCN (3.0 mL). The crude product purified by flash column chromatography ($SiO_2$, 8-10% ethyl acetate in hexanes) to provide **3ac** as light blue solid, M. P.: 168 $^{o}$C; yield: 111.0 mg, 81%. $^{1}$H NMR (400 MHz, DMSO-$d_6$): $\delta$ 12.42 (s, 1H), 8.11-8.06 (m, 2H), 7.68 (dt, $J$ = 8.4 & 1.2 Hz, 1H), 7.51-7.43 (m, 3H), 7.33-7.29 (m, 1H), 7.24 (d, $J$ = 1.2 Hz, 1H), 7.14-7.10 m, 1H); $^{13}$C{H} NMR (100 MHz, DMSO-$d_6$): $\delta$ 165.6 (d, $J$ = 254.0 Hz), 137.5, 133.6, 130.1 (d, $J$ = 10.0 Hz), 126.9, 126.2, 122.7, 121.7, 116.8, 116.6, 112.6, 109.4. $^{19}$F NMR (376 MHz, DMSO-$d_6$): $\delta$ –(105.52-105.56) (m, 1F).

**2-((4-Fluorophenyl)sulfonyl)-5-methyl-*1H*-indole (3bc):**[S4]

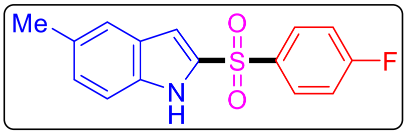

The titled compound was prepared following the general procedure, using 5-methyl-*1H*-indole (**1b**, 0.5 mmol, 66.0 mg, 1.0 equiv.), 4-fluoroaniline (**2c**, 0.75 mmol, 84.0 mg, 1.5 equiv.), $^{t}$BuONO (1.0 mmol, 103.0 mg, 2.0 equiv.), DABSO (0.6 mmol, 144.0 mg, 1.2 equiv.), and $I_2$ (0.6 mmol, 152.0 mg, 1.2 equiv.) in MeCN (3.0 mL). The crude product purified by flash column chromatography ($SiO_2$, 8-10% ethyl acetate in hexanes) to provide **3bc** as white solid, M. P.: 134-136 $^{o}$C; yield: 120.0 mg, 83%. $^{1}$H NMR (400 MHz, DMSO-$d_6$): $\delta$ 12.31 (s, 1H), 8.09-8.04 (m, 2H), 7.49-7.43 (m, 3H), 7.34 (dd, $J$ = 8.8 & 1.6 Hz, 1H), 7.14-7.12 (m, 2H), 2.34 (s, 2H); $^{13}$C{H} NMR (100 MHz, DMSO-$d_6$): $\delta$ 164.9 (d, $J$ = 252.0), 137.8, 136.4, 133.9, 130.2 (d, $J$ = 10.0 Hz), 129.8, 127.2, 126.3, 121.4, 116.9 (d, $J$ = 23.0 Hz), 112.5, 107.7. $^{19}$F NMR (376 MHz, DMSO-$d_6$): $\delta$ –(105.57-105.65) (m, 1F).

**2-((4-Fluorophenyl)sulfonyl)-*1H*-indol-5-ol (3cc)**

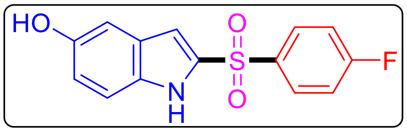

The titled compound was prepared following the general procedure, using *1H*-indol-5-ol (**1c**, 0.5 mmol, 67.0 mg, 1.0 equiv.), 4-fluoroaniline (**2c**, 0.75 mmol, 84.0 mg, 1.5 equiv.), $^t$BuONO (1.0 mmol, 103.0 mg, 2.0 equiv.), DABSO (0.6 mmol, 144.0 mg, 1.2 equiv.), and $I_2$ (0.6 mmol, 152.0 mg, 1.2 equiv.) in MeCN (3.0 mL). The crude product purified by flash column chromatography ($SiO_2$, 8-10% ethyl acetate in hexanes) to provide **3cc** as white solid, M. P.: 191 $^oC$; yield: 128.0 mg, 88%. $^1H$ NMR (400 MHz, DMSO-$d_6$): $\delta$ 12.21 (d, $J$ = 2.4 Hz, 1H), 9.19 (s, 1H), 8.09 (dd, $J$ = 8.8 & 4.8 Hz, 2H), 7.42-7.35 (m, 3H), 7.09 (dd, $J$ = 18.0 & 2.4 Hz, 2H), 6.96 (dd, $J$ = 9.2 & 2.8 Hz, 1H); $^{13}C\{H\}$ NMR (100 MHz, DMSO-$d_6$): $\delta$ 164.9 (d, $J$ = 252.0 Hz), 152.2, 138.1, 134.2, 132.9, 130.3 (d, $J$ = 10.0 Hz), 127.2, 117.4, 116.9 (d, $J$ = 23.0 Hz), 113.7, 107.4, 105.0. $^{19}F$ NMR (376 MHz, DMSO-$d_6$): $\delta$ –(105.71-105.78) (m, 1F); HRMS (ESI, TOF) m/z, calcd for $C_{14}H_{10}FSNO_3$ [M]+ 291.0365, found 291.0374.

**5-Fuoro-2-((4-fluorophenyl)sulfonyl)-*1H*-indole (3ec):**

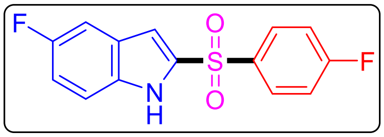

The titled compound was prepared following the general procedure, using 5-fluoro-*1H*-indole (**1e**, 0.5 mmol, 68.0 mg, 1.0 equiv.), 4-fluoroaniline (**2c**, 0.75 mmol, 84.0 mg, 1.5 equiv.), $^t$BuONO (1.0 mmol, 103.0 mg, 2.0 equiv.), DABSO (0.6 mmol, 144.0 mg, 1.2 equiv.), and $I_2$ (0.6 mmol, 152.0 mg, 1.2 equiv.) in MeCN (3.0 mL). The crude product purified by flash column chromatography ($SiO_2$, 8-10% ethyl acetate in hexanes) to provide **3ec** as white solid, M. P.: 127-128 $^oC$; yield: 109.0 mg, 75%. $^1H$ NMR (400 MHz, DMSO-$d_6$): $\delta$ 12.58 (s, 1H), 8.11-8.06 (m, 2H), 7.51-7.44 (m, 4H), 7.22-7.16 (m, 2H); $^{13}C\{H\}$ NMR (100 MHz, DMSO-$d_6$): $\delta$ 165.0 (d, $J$ = 252.0 Hz), 157.5 (d, $J$ = 241.0 Hz), 137.4, 135.8, 134.6, 130.4 (d, $J$ = 10.0 Hz), 126.2 (d, $J$ = 11.0 Hz), 117.1 (d, $J$ = 23.0 Hz), 114.6 (d, $J$ = 27.0 Hz), 114.4 (d, $J$ = 9.0 Hz), 107.9, 106.6 (d, $J$ = 23.0 Hz); $^{19}F$ NMR (376 MHz, DMSO-$d_6$): $\delta$ –(105.16-105.26) (m, 1F), -(122.84-122.93) (m, 1F); HRMS (EI), calcd for $C_{14}H_9F_2SNO_2$ [M]+ 293.0322, found 293.0317.

**5-Chloro-2-((4-fluorophenyl)sulfonyl)-*1H*-indole (3fc):**

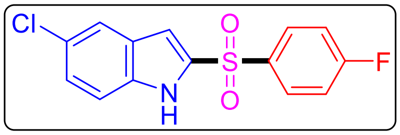

The titled compound was prepared following the general procedure, using 5-chloro-*1H*-indole (**1f**, 0.5 mmol, 76.0 mg, 1.0 equiv.), 4-fluoroaniline (**2c**, 0.75 mmol, 84.0 mg, 1.5 equiv.), $^t$BuONO (1.0 mmol, 103.0 mg, 2.0 equiv.), DABSO (0.6 mmol, 144.0 mg, 1.2 equiv.), and $I_2$ (0.6 mmol, 152.0 mg, 1.2 equiv.) in MeCN (3.0 mL). The crude product purified by flash column chromatography ($SiO_2$, 8-10% ethyl acetate in hexanes) to provide **3fc** as white solid, M. P.: 163-164 °C; yield: 109.0 mg, 77%. $^1$H NMR (400 MHz, DMSO-$d_6$): $\delta$ 12.68 (s, 1H), 8.11-8.07 (m, 2H), 7.74 (dd, $J$ = 4.0 & 2.4 Hz, 1H), 7.50-7.44 (m, 3H), 7.31-7.28 (m, 1H), 7.22 (s, 1H); $^{13}$C{H} NMR (100 MHz, DMSO-$d_6$): $\delta$ 165.1 (d, $J$ = 252.0 Hz), 137.3, 136.3, 135.8, 130.5 (d, $J$ = 10.0 Hz), 127.1, 125.8, 125.5, 121.5, 117.1 (d, $J$ = 22.0 Hz), 114.6, 107.7. $^{19}$F NMR (376 MHz, DMSO-$d_6$): $\delta$ –(105.07-105.15) (m, 1F); HRMS (EI), calcd for $C_{14}H_9ClSFNO_2$ [M]+ 309.0027, found 309.0033.

**5-Bromo-2-((4-fluorophenyl)sulfonyl)-*1H*-indole (3gc):**[S1]

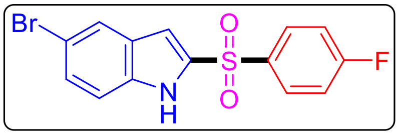

The titled compound was prepared following the general procedure, using 5-bromo-*1H*-indole (**1g**, 0.5 mmol, 98.0 mg, 1.0 equiv.), 4-fluoroaniline (**2c**, 0.75 mmol, 84.0 mg, 1.5 equiv.), $^t$BuONO (1.0 mmol, 103.0 mg, 2.0 equiv.), DABSO (0.6 mmol, 144.0 mg, 1.2 equiv.), and $I_2$ (0.6 mmol, 152.0 mg, 1.2 equiv.) in MeCN (3.0 mL). The crude purified by flash column chromatography ($SiO_2$, 8-10% ethyl acetate in hexanes) to provide **3gc** as yellow solid, M. P.: 163-164 °C; yield: 148.0 mg, 83%. $^1$H NMR (400 MHz, DMSO-$d_6$): $\delta$ 12.71 (s, 1H), 8.12-8.08 (m, 2H), 7.86 (t, $J$ = 2.0 Hz, 1H), 7.47-7.36 (m, 4H), 7.26 (s, 1H); $^{13}$C{H} NMR (100 MHz, DMSO-$d_6$): $\delta$ 165.1 (d, $J$ = 253.0 Hz), 137.3, 136.5, 135.6, 130.5, (d, $J$ = 9.0 Hz), 128.2, 127.7, 124.6, 117.0 (d, $J$ = 23.0 Hz), 114.9, 113.4, 107.6. $^{19}$F NMR (376 MHz, DMSO-$d_6$): $\delta$ –(105.07-105.16) (m, 1F).

**6-Chloro-2-((4-fluorophenyl)sulfonyl)-*1H*-indole (3ic):**

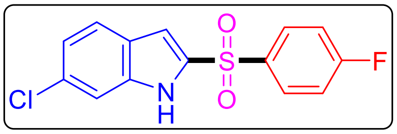

The titled compound was prepared following the general procedure, using 6-chloro-*1H*-indole (**1i**, 0.5 mmol, 76.0 mg, 1.0 equiv.), 4-fluoroaniline (**2c**, 0.75 mmol, 84.0 mg, 1.5 equiv.), $^t$BuONO (1.0 mmol, 103.0 mg, 2.0 equiv.), DABSO (0.6 mmol, 144.0 mg, 1.2 equiv.), and $I_2$ (0.6 mmol, 152.0 mg, 1.2 equiv.) in MeCN (3.0 mL). The crude purified by flash column chromatography

($SiO_2$, 8-10% ethyl acetate in hexanes) to provide **3ic** as white solid, M. P.: 186-187 °C; yield: 110.0 mg, 71%. $^1$H NMR (400 MHz, DMSO-$d_6$): $\delta$ 12.62 (s, 1H), 8.11-8.06 (m, 2H), 7.69 (dt, $J$ = 8.4 & 1.2 Hz, 1H), 7.51-7.46 (m, 3H), 7.27 (d, $J$ = 1.2 Hz, 1H), 7.15-7.12 (m, 1H); $^{13}$C{H} NMR (100 MHz, DMSO-$d_6$): $\delta$ 165.0 (d, $J$ = 252.0 Hz), 138.1, 137.3, 135.2, 130.4 (d, $J$ = 10.0 Hz), 130.1, 124.8, 124.1, 121.6, 117.1 (d, $J$ = 22.0 Hz), 112.2, 108.3; $^{19}$F NMR (376 MHz, DMSO-$d_6$): $\delta$ –(105.15-105.24) (m, 1F); HRMS (ESI, TOF) m/z, calcd for $C_{14}H_9ClSFNO_2$ [M]+ 309.0027, found 309.0023.

**2-((4-Chlorophenyl)sulfonyl)-*1H*-indole (3ad):**[S1]

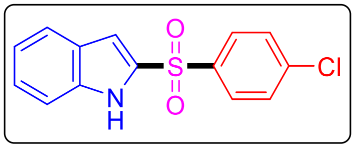


The titled compound was prepared following the general procedure, using indole (**1a**, 0.5 mmol, 59.0 mg, 1.0 equiv.), 4-chloroaniline (**2d**, 0.75 mmol, 96.0 mg, 1.5 equiv.), $^t$BuONO (1.0 mmol, 103.0 mg, 2.0 equiv.), DABSO (0.6 mmol, 144.0 mg, 1.2 equiv.), and $I_2$ (0.6 mmol, 152.0 mg, 1.2 equiv.) in MeCN (3.0 mL). The crude product purified by flash column chromatography ($SiO_2$, 8-10% ethyl acetate in hexanes) to provide **3ad** as white solid, M. P.: 164 °C; yield: 110.0 mg, 75%. $^1$H NMR (400 MHz, $CDCl_3$): $\delta$ 9.35 (s, 1H), 7.97-7.92 (m, 2H), 7.66 (d, $J$ = 7.6 Hz, 1H), 7.47-7.41 (m, 3H), 7.34 (t, $J$ = 7.2 Hz, 1H), 7.23-7.16 (m, 2H); $^{13}$C{H} NMR (100 MHz, $CDCl_3$): $\delta$ 140.0, 139.6, 137.4, 133.5, 129.8, 128.8, 127.1, 126.4, 122.8, 121.8, 112.4, 109.7.

**2-((4-Chlorophenyl)sulfonyl)-5-methyl-*1H*-indole (3bd):**[S1]

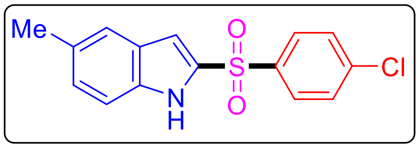


The titled compound was prepared following the general procedure, using 5-methyl-*1H*-indole (**1b**, 0.5 mmol, 66.0 mg, 1.0 equiv.), 4-chloroaniline (**2d**, 0.75 mmol, 96.0 mg, 1.5 equiv.), $^t$BuONO (1.0 mmol, 103.0 mg, 2.0 equiv.), DABSO (0.6 mmol, 144.0 mg, 1.2 equiv.), and $I_2$ (0.6 mmol, 152.0 mg, 1.2 equiv.) in MeCN (3.0 mL). The crude product purified by flash column chromatography ($SiO_2$, 8-10% ethyl acetate in hexanes) to provide **3bd** as brown solid, M. P.: 144-145 °C; yield: 124.0 mg, 81%. $^1$H NMR (400 MHz, $CDCl_3$): $\delta$ 12.34 (s, 1H), 8.02-7.98 (m, 2H), 7.67 (dd, $J$ = 7.2 & 2.0 Hz, 2H), 7.42 (s, 1H), 7.35 (d, $J$ = 8.8 Hz, 1H), 7.16 (s, 1H), 7.12 (dd, $J$ =

8.4 & 1.6 Hz, 1H), 2.32 (s, 3H); $^{13}C\{H\}$ NMR (100 MHz, $CDCl_3$): $\delta$ 140.2, 138.7, 136.4, 133.5, 129.8, 128.9, 127.5, 126.3, 121.4, 112.5, 107.9, 21.05.

**2-((4-Chlorophenyl)sulfonyl)-*1H*-indol-5-ol (3cd):**

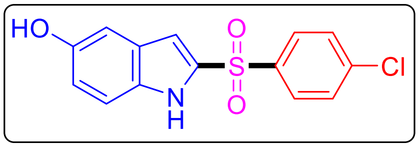


The titled compound was prepared following the general procedure, using *1H*-indol-5-ol (**1c**, 0.5 mmol, 67.0 mg, 1.0 equiv.), 4-chloroaniline (**2d**, 0.75 mmol, 96.0 mg, 1.5 equiv.), $^t$BuONO (1.0 mmol, 103.0 mg, 2.0 equiv.), DABSO (0.6 mmol, 144.0 mg, 1.2 equiv.), and $I_2$ (0.6 mmol, 152.0 mg, 1.2 equiv.) in MeCN (3.0 mL). The crude product purified by flash column chromatography ($SiO_2$, 8-10% ethyl acetate in hexanes) to provide **3cd** as white solid, M. P.: 206-208 °C; yield: 125.0 mg, 81%. $^1H$ NMR (400 MHz, DMSO-$d_6$): $\delta$ 12.18 (s, 1H), 9.13 (s, 1H), 8.00-7.97 (m, 2H), 7.68-7.64 (m, 2H), 7.30 (d, $J$ = 9.2 Hz, 1H), 7.09 (t, $J$ = 1.2 Hz, 1H), 6.99 (d, $J$ = 2.4 Hz, 1H), 6.90 (dd, $J$ = 8.8 & 2.4 Hz, 1H); $^{13}C\{H\}$ NMR (100 MHz, DMSO-$d_6$): $\delta$ 152.0, 140.4, 138.7, 135.5, 132.8, 129.8, 128.9, 126.9, 117.3, 113.5, 107.5, 104.8; HRMS (ESI, TOF) m/z, calcd for $C_{14}H_{10}ClSNO_3$ [M]+ 307.0070, found 307.0077.

**2-((4-chlorophenyl)sulfonyl)-5-methoxy-*1H*-indole (3dd):**[S1]

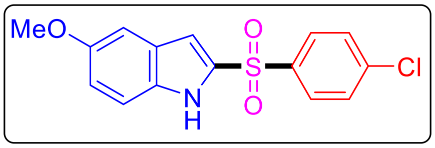


The titled compound was prepared following the general procedure, using 5-methoxy-*1H*-indole (**1d**, 0.5 mmol, 74.0 mg, 1.0 equiv.), 4-chloroaniline (**2d**, 0.75 mmol, 96.0 mg, 1.5 equiv.), $^t$BuONO (1.0 mmol, 103.0 mg, 2.0 equiv.), DABSO (0.6 mmol, 144.0 mg, 1.2 equiv.), and $I_2$ (0.6 mmol, 152.0 mg, 1.2 equiv.) in MeCN (3.0 mL). The crude product purified by flash column chromatography ($SiO_2$, 8-10% ethyl acetate in hexanes) to provide **3dd** as white solid, M. P.: 120-121 °C; yield: 134.0 mg, 83%. $^1H$ NMR (400 MHz, DMSO-$d_6$): $\delta$ 12.34 (s, 1H), 8.00-7.97 (m, 2H), 7.69-7.66 (m, 2H), 7.36 (d, $J$ = 9.2 Hz, 1H), 7.16 (s, 1H), 7.12 (d, $J$ = 2.4 Hz, 1H), 6.98 (dd, $J$ = 9.2 & 2.4 Hz, 1H), 3.73 (s, 3H); $^{13}C\{H\}$ NMR (100 MHz, DMSO-$d_6$): $\delta$ 154.4, 140.3, 138.7, 133.6, 133.3, 129.8, 128.9, 126.5, 117.3, 113.8, 108.0, 102.2, 55.3.

**2-((4-chlorophenyl)sulfonyl)-5-fluoro-*1H*-indole (3ed):**[S5]

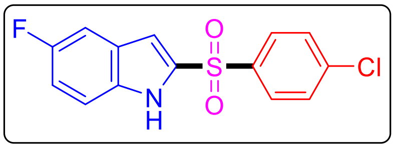


The titled compound was prepared following the general procedure, using 5-fluoro-*1H*-indole (**1e**, 0.5 mmol, 68.0 mg, 1.0 equiv.), 4-chloroaniline (**2d**, 0.75 mmol, 96.0 mg, 1.5 equiv.), $^t$BuONO (1.0 mmol, 103.0 mg, 2.0 equiv.), DABSO (0.6 mmol, 144.0 mg, 1.2 equiv.), and $I_2$ (0.6 mmol, 152.0 mg, 1.2 equiv.) in MeCN (3.0 mL). The crude product purified by flash column chromatography ($SiO_2$, 8-10% ethyl acetate in hexanes) to provide **3ed** as white solid, M. P.: 153 $^o$C; yield: 113.0 mg, 73%. $^1$H NMR (400 MHz, DMSO-$d_6$): $\delta$ 12.64 (s, 1H), 8.03-7.99 (m, 2H), 7.66-7.62 (m, 2H), 7.49-7.43 (m, 2H), 7.24 (d, $J$ = 1.6 Hz, 1H), 7.20-7.15 (m, 1H); $^{13}$C{H} NMR (100 MHz, DMSO-$d_6$): $\delta$ 157.6 (d, $J$ = 234.0), 139.8, 139.0, 135.5, 134.7, 129.9, 129.1, 126.2 (d, $J$ = 11.0 Hz), 114.7 (d, $J$ = 27.0 Hz), 114.4 (d, $J$ = 9.0 Hz), 108.3, 106.6 (d, $J$ = 24.0 Hz); $^{19}$F NMR (376 MHz, DMSO-$d_6$): $\delta$ –(122.81-122.88) (m, 1F).

**5-Bromo-2-((4-chlorophenyl)sulfonyl)-*1H*-indole (3gd):**[S1]

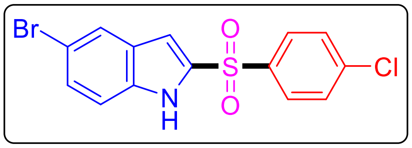


The titled compound was prepared following the general procedure, using 5-bromo-*1H*-indole (**1g**, 0.5 mmol, 98.0 mg, 1.0 equiv.), 4-chloroaniline (**2d**, 0.75 mmol, 96.0 mg, 1.5 equiv.), $^t$BuONO (1.0 mmol, 103.0 mg, 2.0 equiv.), DABSO (0.6 mmol, 144.0 mg, 1.2 equiv.), and $I_2$ (0.6 mmol, 152.0 mg, 1.2 equiv.) in MeCN (3.0 mL). The crude product purified by flash column chromatography ($SiO_2$, 8-10% ethyl acetate in hexanes) to provide **3gd** as white solid, M. P.: 198 $^o$C; yield: 144.0 mg, 78%. $^1$H NMR (400 MHz, DMSO-$d_6$): $\delta$ 12.71 (s, 1H), 8.02-7.99 (m, 2H), 7.88 (d, $J$ = 2.0 Hz, 1H), 7.68-7.65 (m, 2H), 7.44-7.37 (m, 2H), 7.24 (d, $J$ = 2.0 Hz, 1H); $^{13}$C{H} NMR (100 MHz, DMSO-$d_6$): $\delta$ 139.7, 139.1, 136.5, 135.2, 129.9, 129.1, 128.2, 127.7, 124.6, 114.9, 113.4, 107.8.

**6-Chloro-2-((4-chlorophenyl)sulfonyl)-*1H*-indole (3id):**[S5]

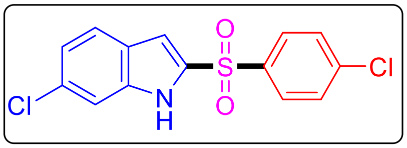

The titled compound was prepared following the general procedure, using 6-chloro-*1H*-indole (**1i**, 0.5 mmol, 76.0 mg, 1.0 equiv.), 4-chloroaniline (**2d**, 0.75 mmol, 96.0 mg, 1.5 equiv.), $^t$BuONO (1.0 mmol, 103.0 mg, 2.0 equiv.), DABSO (0.6 mmol, 144.0 mg, 1.2 equiv.), and $I_2$ (0.6 mmol, 152.0 mg, 1.2 equiv.) in MeCN (3.0 mL). The crude product purified by flash column chromatography ($SiO_2$, 8-10% ethyl acetate in hexanes) to provide **3id** as white solid, M. P.: 183-184 $^o$C; yield: 114.0 mg, 74%. $^1$H NMR (400 MHz, DMSO-*d*$_6$): *δ* 12.65 (s, 1H), 7.02-7.99 (m, 2H), 7.70-7.67 (m, 3H), 7.47 (t, *J* = 3.6 Hz, 1H), 7.29 (d, *J* = 2.8 Hz, 1H), 7.14-7.10 (m, 1H); $^{13}$C{H} NMR (100 MHz, DMSO-*d*$_6$): *δ* 139.7, 139.0, 138.1, 134.8, 130.2, 129.9, 124.8, 124.1, 121.7, 112.3, 108.6.

**2-((4-Chlorophenyl)sulfonyl)-1-methyl-*1H*-indole (3jd)[S1]:**

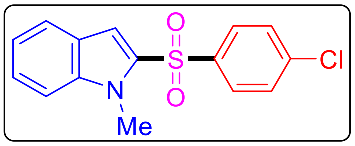


The titled compound was prepared following the general procedure, using 1-methyl-*1H*-indole (**1j**, 0.5 mmol, 66.0 mg, 1.0 equiv.), 4-chloroaniline (**2d**, 0.75 mmol, 96.0 mg, 1.5 equiv.), $^t$BuONO (1.0 mmol, 103.0 mg, 2.0 equiv.), DABSO (0.6 mmol, 144.0 mg, 1.2 equiv.), and $I_2$ (0.6 mmol, 152.0 mg, 1.2 equiv.) in MeCN (3.0 mL). The crude product purified by flash column chromatography ($SiO_2$, 8-10% ethyl acetate in hexanes) to provide **3jd** as white solid, M. P.: 152-153 $^o$C; yield: 124.0 mg, 81%. $^1$H NMR (400 MHz, DMSO-*d*$_6$): *δ* 8.00-7.97 (m, 2H), 7.75-7.68 (m, 3H), 7.56-7.54 (m, 1H), 7.43 (t, *J* =0.8 Hz, 1H), 7.39-7.36 (m, 1H), 7.19-7.16 (m, 1H), 3.83 (s. 3H); $^{13}$C{H} NMR (100 MHz, DMSO-*d*$_6$): *δ* 139.4, 139.3, 139.0, 133.9, 130.1, 129.3, 125.9, 124.6, 122.7, 121.2, 111.2, 110.6, 31.0.

**2-(Naphthalen-2-ylsulfonyl)-*1H*-indole (3ae)[S2]:**

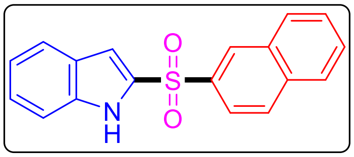


The titled compound was prepared following the general procedure, using indole (**1a**, 0.5 mmol, 59.0 mg, 1.0 equiv.), naphthalen-2-amine (**2e**, 0.75 mmol, 107.0 mg, 1.5 equiv.), $^t$BuONO (1.0 mmol, 103.0 mg, 2.0 equiv.), DABSO (0.6 mmol, 144.0 mg, 1.2 equiv.), and $I_2$ (0.6 mmol, 152.0 mg, 1.2 equiv.) in MeCN (3.0 mL). The crude product purified by flash column chromatography ($SiO_2$, 8-10% ethyl acetate in hexanes) to provide **3ae** as white solid, M. P.: 132-133 $^o$C; yield:

125.0 mg, 81%. $^{1}$H NMR (400 MHz, DMSO-$d_6$): $\delta$ 12.45 (s, 1H), 8.72 (d, $J$ = 1.6 Hz, 1H), 8.19 (d, $J$ = 8.8 Hz, 1H), 8.13 (d, $J$ = 8.8 Hz, 1H), 8.02-7.97 (m, 2H), 7.72-7.65 (m, 3H), 7.44 (d, $J$ =8.4 Hz, 1H), 7.30-7.27 (m, 2H), 7.10 (t, $J$ = 7.6 Hz, 1H); $^{13}$C{H} NMR (100 MHz, DMSO-$d_6$): $\delta$ 138.2, 137.9, 134.6, 134.3, 131.7, 129.9, 129.5, 128.4, 127.9, 126.0, 125.4, 122.4, 122.2, 120.9, 112.8, 108.2.

**2-(*o*-Tolylsulfonyl)-*1H*-indole (3af)**

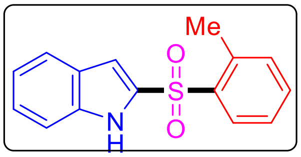


The titled compound was prepared following the general procedure, using indole (**1a**, 0.5 mmol, 59.0 mg, 1.0 equiv.), *o*-toluidine (**2f**, 0.75 mmol, 80.0 mg, 1.5 equiv.), $^{t}$BuONO (1.0 mmol, 103.0 mg, 2.0 equiv.), DABSO (0.6 mmol, 144.0 mg, 1.2 equiv.), and $I_2$ (0.6 mmol, 152.0 mg, 1.2 equiv.) in MeCN (3.0 mL). The crude product purified by flash column chromatography ($SiO_2$, 8-10% ethyl acetate in hexanes) to provide **3af** as colorless oil; yield: 89.0 mg, 65%. $^{1}$H NMR (400 MHz, DMSO-$d_6$): $\delta$ 12.34 (s, 1H), 7.89 (d, $J$ = 8.0 Hz, 2H), 7.66 (d, $J$ = 8.0 Hz, 1H), 7.43 (d, $J$ = 8.4 Hz, 3H), 7.31-7.27 (m, 1H), 7.18 (d, $J$ = 0.8 Hz, 1H), 7.13-7.09 (m, 1H), 2.35 (s, 3H); $^{13}$C{H} NMR (100 MHz, DMSO-$d_6$): $\delta$ 144.4, 138.4, 137.8, 134.7, 130.1, 127.1, 125.9, 125.3, 122.3, 120.8, 122.7, 107.7, 21.0. HRMS (FTMS-p ESI, TOF) m/z, calcd for $C_{15}H_{13}NO_2S$ $[M–H]^-$ 270.0583, found 270.0593.

**5-Methoxy-2-(*o*-tolylsulfonyl)-*1H*-indole (3df)**

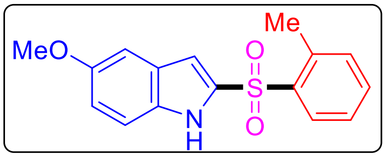


The titled compound was prepared following the general procedure, using 5-methoxy-*1H*-indole (**1d**, 0.5 mmol, 74.0 mg, 1.0 equiv.), *o*-toluidine (**2f**, 0.75 mmol, 80.0 mg, 1.5 equiv.), $^{t}$BuONO (1.0 mmol, 103.0 mg, 2.0 equiv.), DABSO (0.6 mmol, 144.0 mg, 1.2 equiv.), and $I_2$ (0.6 mmol, 152.0 mg, 1.2 equiv.) in MeCN (3.0 mL). The crude product purified by flash column chromatography ($SiO_2$, 8-10% ethyl acetate in hexanes) to provide **3df** as white solid, M.P.: 109-111 $^{o}$C; yield: 112.0 mg, 74%. $^{1}$H NMR (400 MHz, DMSO-$d_6$): $\delta$ 12.16 (s, 1H), 8.06 (dd, $J$ = 8.0 & 1.6 Hz, 1H), 7.58 (td, $J$ = 7.6 & 1.6 Hz, 1H), 7.51-7.49 (m, 1H), 7.38-7.32 (m, 2H), 7.15-7.14 (m, 2H), 6.96 (dd, $J$ = 9.2 & 2.4 Hz, 1H), 3.75 (s, 3H), 2.75 (s, 3H); $^{13}$C{H} NMR (100 MHz,

DMSO-$d_6$): $\delta$ 154.3, 139.1, 137.3, 133.9, 133.0, 132.8, 128.7, 126.9, 126.3, 117.0, 113.7, 107.9, 102.2, 155.3, 19.5. HRMS (FTMS-p ESI, TOF) m/z, calcd for $C_{16}H_{15}NO_3S$ $[M-H]^-$ 300.0689, found 300.0699.

**2-((4-Methoxyphenyl)sulfonyl)-*1H*-indole (3ag)[S7]:**

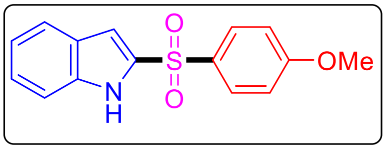


The titled compound was prepared following the general procedure, using indole (**1a**, 0.5 mmol, 59.0 mg, 1.0 equiv.), 4-methoxyaniline (**2g**, 0.75 mmol, 92.0 mg, 1.5 equiv.), $^t$BuONO (1.0 mmol, 103.0 mg, 2.0 equiv.), DABSO (0.6 mmol, 144.0 mg, 1.2 equiv.), and $I_2$ (0.6 mmol, 152.0 mg, 1.2 equiv.) in MeCN (3.0 mL). The crude product purified by flash column chromatography ($SiO_2$, 10-15% ethyl acetate in hexanes) to provide **3ag** as white solid, yield: 118 mg, 82%. $^1$H NMR (400 MHz, DMSO-$d_6$): $\delta$ 12.36 (s, 1H), 7.95 (d, $J$ = 8.8 Hz, 2H), 7.66 (dd, $J$ = 8.4 & 1.2 Hz, 1H), 7.44 (dd, $J$ = 8.0 & 1.2 Hz, 1H), 7.30-7.26 (m, 1H), 7.16-7.08 (m, 4H), 3.80 (s, 3H); $^{13}$C{H} NMR (100 MHz, DMSO-$d_6$): $\delta$ 163.2, 137.7, 135.3, 132.8, 129.5, 126.1, 125.2, 122.3, 120.8, 114.9, 112.7, 107.3, 55.8.

**3-Methyl-2-(phenylsulfonyl)-*1H*-indole (3na)[S1]:**

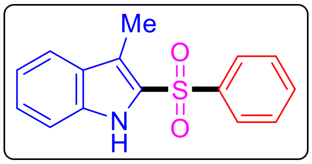


The titled compound was prepared following the general procedure, using 3-methyl-*1H*-indole (**1n**, 0.5 mmol, 59.0 mg, 1.0 equiv.), aniline (**2a**, 0.75 mmol, 70.0 mg, 1.5 equiv.), $^t$BuONO (1.0 mmol, 103.0 mg, 2.0 equiv.), DABSO (0.6 mmol, 144.0 mg, 1.2 equiv.), and $I_2$ (0.6 mmol, 152.0 mg, 1.2 equiv.) in MeCN (3.0 mL). The crude product purified by flash column chromatography ($SiO_2$, 10-15% ethyl acetate in hexanes) to provide **3na** as white solid, yield: 108 mg, 79%. $^1$H NMR (400 MHz, $CDCl_3$): $\delta$ 9.22 (s, 1H), 7.98 (dd, $J$ = 7.6 & 1.2 Hz, 2H), 7.59 (d, $J$ = 8.0 Hz, 1H), 7.56-7.52 (m, 1H), 7.50-7.45 (m, 2H), 7.41-7.39 (m, 1H), 7.34-7.29 (m, 1H), 7.17-7.23 (m, 1H), 2.52 (s, 3H); $^{13}$C{H} NMR (100 MHz, $CDCl_3$): $\delta$ 141.9, 136.1, 133.3, 129.4, 129.1, 128.3, 126.9, 126.3, 120.8, 118.9, 112.3, 8.9.

**2-(Phenylsulfonyl)-*1H*-pyrrole (5aa):[S6]**

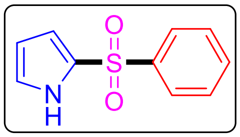


The titled compound was prepared following the general procedure, using *1H*-pyrrole (**4a**, 0.5 mmol, 34.0 mg, 1.0 equiv.), aniline (**2a**, 0.75 mmol, 70.0 mg, 1.5 equiv.), $^t$BuONO (1.0 mmol, 103.0 mg, 2.0 equiv.), DABSO (0.6 mmol, 144.0 mg, 1.2 equiv.), and $I_2$ (0.6 mmol, 152.0 mg, 1.2 equiv.) in MeCN (3.0 mL). The crude product purified by flash column chromatography ($SiO_2$, 5-10% ethyl acetate in hexanes) to provide **5aa** as white solid, M. P.: 100-101 $^o$C; yield: 91 mg, 89%.$^1$H NMR (400 MHz, DMSO-$d_6$): $\delta$ 12.38 (s, 1H), 7.91-7.89 (m, 2H), 7.65-7.57 (m, 3H), 7.12-7.11 (m, 1H), 6.84-6.82 (m, 1H), 6.22-6.20 (m, 1H); $^{13}$C{H} NMR (100 MHz, DMSO-$d_6$): $\delta$ 142.6, 133.0, 129.5, 127.6, 126.4, 125.4, 115.5, 109.8.

**2-Tosyl-*1H*-pyrrole (5ab):**[S6]

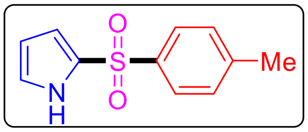


The titled compound was prepared following the general procedure, using *1H*-pyrrole (**4a**, 0.5 mmol, 34.0 mg, 1.0 equiv.), *p*-toluidine (**2b**, 0.75 mmol, 80.0 mg, 1.5 equiv.), $^t$BuONO (1.0 mmol, 103.0 mg, 2.0 equiv.), DABSO (0.6 mmol, 144.0 mg, 1.2 equiv.), and $I_2$ (0.6 mmol, 152.0 mg, 1.2 equiv.) in MeCN (3.0 mL). The crude product purified by flash column chromatography ($SiO_2$, 8-10% ethyl acetate in hexanes) to provide **5ab** as color less oil; yield: 98 mg, 89%. $^1$H NMR (400 MHz, DMSO-$d_6$): $\delta$ 12.33 (s, 1H), 7.79 (dd, $J$ = 6.4 & 2.0, 2H), 7.10-7.08 (m, 1H), 6.80-6.78 (m, 3H), 6.20-6.18 (m, 1H), 2.33 (s, 3H); $^{13}$C{H} NMR (100 MHz, DMSO-$d_6$): $\delta$ 143.5, 139.8, 129.9, 128.1, 126.5, 125.1, 115.1, 109.7, 20.9.

**2-((4-Fluorophenyl)sulfonyl)-1H-pyrrole (5ac):**

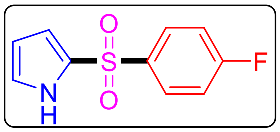


The titled compound was prepared following the general procedure, using *1H*-pyrrole (**4a**, 0.5 mmol, 34.0 mg, 1.0 equiv.), 4-fluoroaniline (**2c**, 0.75 mmol, 80.0 mg, 1.5 equiv.), $^t$BuONO (1.0 mmol, 103.0 mg, 2.0 equiv.), DABSO (0.6 mmol, 144.0 mg, 1.2 equiv.), and $I_2$ (0.6 mmol, 152.0 mg, 1.2 equiv.) in MeCN (3.0 mL). The crude product purified by flash column chromatography

($SiO_2$, 8-10% ethyl acetate in hexanes) to provide **5ac** as yellow solid, M. P.: 175 °C; yield: 88 mg, 78%. $^1$H NMR (400 MHz, DMSO-$d_6$): $\delta$ 12.40 (s, 1H), 7.99-7.95 (m, 2H), 7.47-7.41 (m, 2H), 7.13 (t, $J$ = 2.0 Hz, 1H), 6.84 (dd, $J$ = 4.0 & 1.6 Hz, 1H), 6.21 (dd, $J$ = 4.0 & 2.4 Hz, 1H); $^{13}$C{H} NMR (100 MHz, DMSO-$d_6$): $\delta$ 164.4 (d, $J$ = 251.0 Hz), 138.9, 129.6 (d, $J$ = 9.0 Hz), 127.4, 125.5, 116.7 (d, $J$ = 23.0 Hz), 115.4, 109.8; $^{19}$F NMR (376 MHz, DMSO-$d_6$) $\delta$ -105.66. HRMS (ESI, TOF) m/z, calcd for $C_{10}H_8FSNO_2$ $[M]^+$ 225.0260, found 225.0261.

**2-((4-Chlorophenyl)sulfonyl)-*1H*-pyrrole (5ad):**[S6]

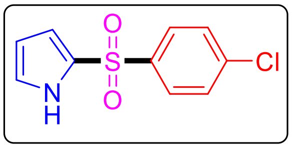


The titled compound was prepared following the general procedure, using *1H*-pyrrole (**4a**, 0.5 mmol, 34.0 mg, 1.0 equiv.), 4-chloroaniline (**2d**, 0.75 mmol, 96.0 mg, 1.5 equiv.), $^t$BuONO (1.0 mmol, 103.0 mg; 2.0 equiv.), DABSO (0.6 mmol, 144.0 mg; 1.2 equiv.), and $I_2$ (0.6 mmol, 152.0 mg, 1.2 equiv.) in MeCN (3.0 mL). The crude product purified by flash column chromatography ($SiO_2$, 8-10% ethyl acetate in hexanes) to provide **5ad** as white solid, M. P.: 170-171 °C; yield: 100.0 mg, 83%. $^1$H NMR (400 MHz, DMSO-$d_6$): $\delta$ 12.43 (s, 1H), 7.91-7.88 (m, 2H), 7.67-7.64 (m, 2H), 7.16-7.14 (m, 1H), 6.87-6.85 (m, 1H), 6.23-6.21 (m, 1H); $^{13}$C{H} NMR (100 MHz, DMSO-$d_6$): $\delta$ δ 141.4, 138.0, 129.6, 128.4, 127.1, 125.7, 115.8, 109.9.

**1-Methyl-2-(phenylsulfonyl)-*1H*-pyrrole (5ba):**[S7]

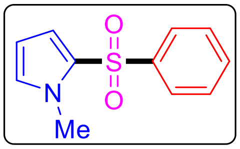


The titled compound was prepared following the general procedure, using 1-methyl-*1H*-pyrrole (**4b**, 0.5 mmol, 41.0 mg, 1.0 equiv.), aniline (**2a**, 0.75 mmol, 70.0 mg, 1.5 equiv.), $^t$BuONO (1.0 mmol, 103.0 mg, 2.0 equiv.), DABSO (0.6 mmol, 144.0 mg, 1.2 equiv.), and $I_2$ (0.6 mmol, 152.0 mg, 1.2 equiv.) in MeCN (3.0 mL). The crude product purified by flash column chromatography ($SiO_2$, 5-10% ethyl acetate in hexanes) to provide **5ba** as white solid, M. P.: 75-76 °C; yield: 72 mg, 65%. $^1$H NMR (400 MHz, DMSO-$d_6$): $\delta$ 7.88-7.85 (m, 2H), 7.68 -7.59 (m, 3H), 7.14 (t, $J$ = 2.4 Hz, 1H), 6.96 (dd, $J$ = 4.4 & 2.0 Hz, 1H), 6.19 (dd, $J$ = 4.0 & 2.4 Hz, 1H), 3.66 (s, 3H); $^{13}$C{H} NMR (100 MHz, DMSO-$d_6$): $\delta$ 141.8, 133.4, 130.9, 129.7, 127.3, 126.8, 118.3, 108.2, 35.2.

**1-Methyl-2-tosyl-*1H*-pyrrole (5bb):**[S7]

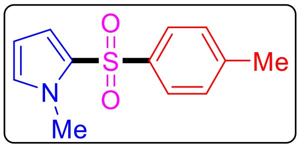


The titled compound was prepared following the general procedure, using 1-methyl-*1H*-pyrrole (**5b**, 0.5 mmol, 41.0 mg, 1.0 equiv.), *p*-toluidine (**2b**, 0.75 mmol, 80.0 mg, 1.5 equiv.), $^t$BuONO (1.0 mmol, 103.0 mg, 2.0 equiv.), DABSO (0.6 mmol, 144.0 mg, 1.2 equiv.), and $I_2$ (0.6 mmol, 152.0 mg, 1.2 equiv.) in MeCN (3.0 mL). The crude product purified by flash column chromatography ($SiO_2$, 8-10% ethyl acetate in hexanes) to provide **5bb** as white solid, M. P.: 100 $^o$C; yield: 79 mg, 67%. $^1$H NMR (400 MHz, DMSO-$d_6$): $\delta$ 7.75 -7.73 (m, 2H), 7.42-7.40 (m, 2H), 7.12 (t, $J$ = 2.6 Hz, 1H), 6.92 (dd, $J$ = 4.0 & 2.0 Hz, 1H), 6.17 (dd, $J$ = 4.0 & 2.8 Hz, 1H), 3.64 (s, 3H), 2.37 (s, 3H); $^{13}$C{H} NMR (100 MHz, DMSO-$d_6$): $\delta$ 143.9, 138.9, 130.6, 130.1, 127.7, 126.8, 117.9, 108.0, 35.1, 21.0. **Crystal data** for **5bb (CCDC 2499577)**: After flash column chromatographic separation, the compound 5aa was dissolved in 20% EA/Hexanes and kept at room temperature for overnight which provided the crystal to perform the single-crystal data analysis. Empirical formula $C_{12}H_{13}NO_2S$; Formula weight: 235.29; Temperature: 150(2) K; Wavelength: 0.71073 Å; Crystal system: Orthorhombic; Space group: Fdd2; Unit cell dimensions: a = 21.6600(11) Å, α= 90°, b = 26.793(2) Å, β= 90°, c = 7.7795(3) Å, γ = 90°; Volume: 4514.8(5) Å$^3$; Z: 16; Density (calculated): 1.385 Mg/m$^3$; Absorption coefficient: 0.270 mm$^{-1}$; F(000): 1984; Crystal size: 0.380 x 0.350 x 0.210 mm$^3$; Theta range for data collection: 3.041 to 27.897°; Index ranges:-28<=h<=28, -35<=k<=35, -10<=l<=10; Reflections collected: 27991; Independent reflections: 2668 [R(int) = 0.0407]; Completeness to theta = 25.242°: 99.8 %; Absorption correction: Semi-empirical from equivalents; Max. and min. transmission: 0.7456 and 0.6908; Refinement method: Full-matrix least-squares on F$^2$; Data / restraints / parameters: 2668 / 1 / 148; Goodness-of-fit on F$^2$:1.189; Final R indices [I>2sigma(I)]: R1 = 0.0305, wR2 = 0.0888; R indices (all data): R1 = 0.0335, wR2 = 0.0899; Absolute structure parameter: 0.013(17); Extinction coefficient: n/a; Largest diff. peak and hole: 0.404 and -0.287 e.Å$^{-3}$ Detailed X-ray crystallographic data is available from the Cambridge Crystallographic Data Centre, 12 Union Road, Cambridge CB2 1EZ, UK (for compound 3aa CCDC # 2174912).

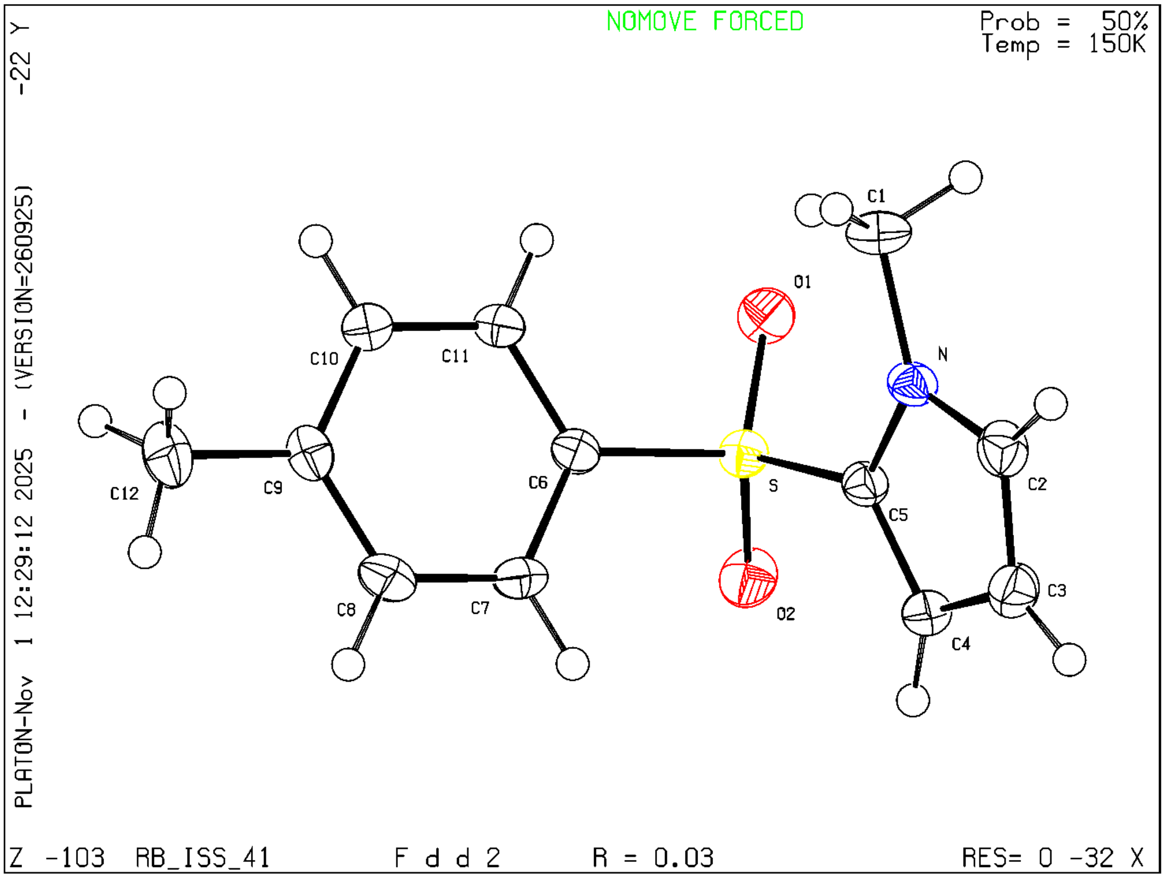


## Table 1. Crystal data and structure refinement for 5bb.

| | | |
|---|---|---|
| Identification code | **5bb** | |
| Empirical formula | $C_{12}H_{13}NO_2S$ | |
| Formula weight | 235.29 | |
| Temperature | 150(2) K | |
| Wavelength | 0.71073 Å | |
| Crystal system | Orthorhombic | |
| Space group | Fdd2 | |
| Unit cell dimensions | a = 21.6600(11) Å | $\alpha$= 90°. |
| | b = 26.793(2) Å | $\beta$= 90°. |
| | c = 7.7795(3) Å | $\gamma$ = 90°. |
| Volume | 4514.8(5) Å$^3$ | |
| Z | 16 | |
| Density (calculated) | 1.385 Mg/m$^3$ | |
| Absorption coefficient | 0.270 mm$^{-1}$ | |

| | |
|---|---|
| F(000) | 1984 |
| Crystal size | 0.380 x 0.350 x 0.210 $mm^3$ |
| Theta range for data collection | 3.041 to 27.897°. |
| Index ranges | -28<=h<=28, -35<=k<=35, -10<=l<=10 |
| Reflections collected | 27991 |
| Independent reflections | 2668 [R(int) = 0.0407] |
| Completeness to theta = 25.242° | 99.8 % |
| Absorption correction | Semi-empirical from equivalents |
| Max. and min. transmission | 0.7456 and 0.6908 |
| Refinement method | Full-matrix least-squares on $F^2$ |
| Data / restraints / parameters | 2668 / 1 / 148 |
| Goodness-of-fit on $F^2$ | 1.189 |
| Final R indices [I>2sigma(I)] | R1 = 0.0305, wR2 = 0.0888 |
| R indices (all data) | R1 = 0.0335, wR2 = 0.0899 |
| Absolute structure parameter | 0.013(17) |
| Extinction coefficient | n/a |
| Largest diff. peak and hole | 0.404 and -0.287 e.$Å^{-3}$ |

**Table 2. Atomic coordinates ( x $10^4$) and equivalent isotropic displacement parameters ($Å^2$x $10^3$) for 5bb. U(eq) is defined as one third of the trace of the orthogonalized $U^{ij}$ tensor.**

| | x | y | z | U(eq) |
|---|---|---|---|---|
| S | 343(1) | 831(1) | 2985(1) | 20(1) |
| O(1) | 966(1) | 663(1) | 3252(3) | 28(1) |
| O(2) | 0(1) | 653(1) | 1516(3) | 30(1) |
| N | 88(1) | 719(1) | 6451(3) | 22(1) |
| C(1) | 704(1) | 838(1) | 7092(4) | 31(1) |
| C(2) | -397(1) | 598(1) | 7470(3) | 27(1) |
| C(3) | -904(1) | 496(1) | 6459(4) | 28(1) |
| C(4) | -719(1) | 553(1) | 4737(3) | 24(1) |
| C(5) | -103(1) | 691(1) | 4756(3) | 20(1) |
| C(6) | 362(1) | 1489(1) | 2858(3) | 20(1) |
| C(7) | -172(1) | 1741(1) | 2375(4) | 26(1) |

| | | | | |
|---|---|---|---|---|
| C(8) | -158(1) | 2257(1) | 2239(4) | 28(1) |
| C(9) | 375(1) | 2525(1) | 2605(3) | 24(1) |
| C(10) | 906(1) | 2266(1) | 3083(4) | 25(1) |
| C(11) | 905(1) | 1748(1) | 3192(3) | 22(1) |
| C(12) | 374(1) | 3087(1) | 2488(4) | 34(1) |

**Table 3. Bond lengths [Å] and angles [°] for 5bb.**

| | |
|---|---|
| S-O(1) | 1.4373(18) |
| S-O(2) | 1.444(2) |
| S-C(5) | 1.725(3) |
| S-C(6) | 1.766(2) |
| N-C(2) | 1.356(3) |
| N-C(5) | 1.384(3) |
| N-C(1) | 1.460(3) |
| C(1)-H(1B) | 0.9800 |
| C(1)-H(1C) | 0.9800 |
| C(1)-H(1D) | 0.9800 |
| C(2)-C(3) | 1.378(4) |
| C(2)-H(2B) | 0.9500 |
| C(3)-C(4) | 1.406(4) |
| C(3)-H(3A) | 0.9500 |
| C(4)-C(5) | 1.385(3) |
| C(4)-H(4A) | 0.9500 |
| C(6)-C(11) | 1.390(3) |
| C(6)-C(7) | 1.391(3) |
| C(7)-C(8) | 1.387(4) |
| C(7)-H(7A) | 0.9500 |
| C(8)-C(9) | 1.389(4) |
| C(8)-H(8A) | 0.9500 |
| C(9)-C(10) | 1.396(3) |
| C(9)-C(12) | 1.507(3) |
| C(10)-C(11) | 1.390(3) |
| C(10)-H(10A) | 0.9500 |
| C(11)-H(11A) | 0.9500 |
| C(12)-H(12A) | 0.9800 |

| | |
|---|---|
| C(12)-H(12B) | 0.9800 |
| C(12)-H(12C) | 0.9800 |
| O(1)-S-O(2) | 119.58(12) |
| O(1)-S-C(5) | 110.02(12) |
| O(2)-S-C(5) | 105.80(11) |
| O(1)-S-C(6) | 107.38(11) |
| O(2)-S-C(6) | 107.27(12) |
| C(5)-S-C(6) | 106.01(12) |
| C(2)-N-C(5) | 108.2(2) |
| C(2)-N-C(1) | 124.1(2) |
| C(5)-N-C(1) | 127.7(2) |
| N-C(1)-H(1B) | 109.5 |
| N-C(1)-H(1C) | 109.5 |
| H(1B)-C(1)-H(1C) | 109.5 |
| N-C(1)-H(1D) | 109.5 |
| H(1B)-C(1)-H(1D) | 109.5 |
| H(1C)-C(1)-H(1D) | 109.5 |
| N-C(2)-C(3) | 109.3(2) |
| N-C(2)-H(2B) | 125.3 |
| C(3)-C(2)-H(2B) | 125.3 |
| C(2)-C(3)-C(4) | 107.2(2) |
| C(2)-C(3)-H(3A) | 126.4 |
| C(4)-C(3)-H(3A) | 126.4 |
| C(5)-C(4)-C(3) | 107.0(2) |
| C(5)-C(4)-H(4A) | 126.5 |
| C(3)-C(4)-H(4A) | 126.5 |
| C(4)-C(5)-N | 108.3(2) |
| C(4)-C(5)-S | 126.1(2) |
| N-C(5)-S | 125.54(18) |
| C(11)-C(6)-C(7) | 120.8(2) |
| C(11)-C(6)-S | 120.48(18) |
| C(7)-C(6)-S | 118.73(18) |
| C(8)-C(7)-C(6) | 119.1(2) |
| C(8)-C(7)-H(7A) | 120.4 |
| C(6)-C(7)-H(7A) | 120.4 |
| C(7)-C(8)-C(9) | 121.2(2) |

| | |
|---|---|
| C(7)-C(8)-H(8A) | 119.4 |
| C(9)-C(8)-H(8A) | 119.4 |
| C(10)-C(9)-C(8) | 118.9(2) |
| C(10)-C(9)-C(12) | 120.9(2) |
| C(8)-C(9)-C(12) | 120.2(2) |
| C(9)-C(10)-C(11) | 120.7(2) |
| C(9)-C(10)-H(10A) | 119.6 |
| C(11)-C(10)-H(10A) | 119.6 |
| C(6)-C(11)-C(10) | 119.3(2) |
| C(6)-C(11)-H(11A) | 120.4 |
| C(10)-C(11)-H(11A) | 120.4 |
| C(9)-C(12)-H(12A) | 109.5 |
| C(9)-C(12)-H(12B) | 109.5 |
| H(12A)-C(12)-H(12B) | 109.5 |
| C(9)-C(12)-H(12C) | 109.5 |
| H(12A)-C(12)-H(12C) | 109.5 |
| H(12B)-C(12)-H(12C) | 109.5 |

Symmetry transformations used to generate equivalent atoms:

**Table 4. Anisotropic displacement parameters ($Å^2$x $10^3$) for 5bb. The anisotropic displacement factor exponent takes the form: $-2p^2$[ $h^2$ $a^{*2}U^{11}$ + ... + 2 h k a* b* $U^{12}$]**

| | $U^{11}$ | $U^{22}$ | $U^{33}$ | $U^{23}$ | $U^{13}$ | $U^{12}$ |
|---|---|---|---|---|---|---|
| S | 22(1) | 20(1) | 19(1) | -4(1) | -1(1) | 0(1) |
| O(1) | 24(1) | 24(1) | 37(1) | -3(1) | 4(1) | 5(1) |
| O(2) | 39(1) | 31(1) | 21(1) | -6(1) | -3(1) | -7(1) |
| N | 23(1) | 22(1) | 19(1) | -2(1) | -5(1) | 0(1) |
| C(1) | 23(1) | 38(1) | 30(1) | -4(1) | -9(1) | -2(1) |
| C(2) | 32(1) | 27(1) | 21(1) | 2(1) | 1(1) | -2(1) |
| C(3) | 28(1) | 28(1) | 27(1) | 3(1) | 1(1) | -4(1) |
| C(4) | 23(1) | 24(1) | 26(1) | 1(1) | -5(1) | -2(1) |
| C(5) | 22(1) | 19(1) | 19(1) | -1(1) | -3(1) | 0(1) |
| C(6) | 20(1) | 22(1) | 17(1) | -1(1) | -1(1) | 1(1) |
| C(7) | 18(1) | 28(1) | 31(1) | 1(1) | -5(1) | -1(1) |
| C(8) | 22(1) | 30(1) | 31(1) | 2(1) | -4(1) | 7(1) |

| | | | | | | |
|---|---|---|---|---|---|---|
| C(9) | 30(1) | 22(1) | 21(1) | 2(1) | 0(1) | 2(1) |
| C(10) | 23(1) | 25(1) | 26(1) | 1(1) | -3(1) | -2(1) |
| C(11) | 19(1) | 24(1) | 23(1) | 0(1) | -3(1) | 1(1) |
| C(12) | 42(2) | 23(1) | 38(2) | 5(1) | -1(1) | 3(1) |

**Table 5. Hydrogen coordinates ( x $10^4$) and isotropic displacement parameters ($Å^2$x $10^3$) for 5bb.**

| | x | y | z | U(eq) |
|---|---|---|---|---|
| H(1B) | 977 | 912 | 6122 | 250(50) |
| H(1C) | 681 | 1128 | 7853 | 140(30) |
| H(1D) | 868 | 552 | 7732 | 150(30) |
| H(2B) | -390 | 587 | 8691 | 32 |
| H(3A) | -1304 | 404 | 6851 | 33 |
| H(4A) | -970 | 505 | 3748 | 29 |
| H(7A) | -541 | 1562 | 2141 | 31 |
| H(8A) | -520 | 2430 | 1889 | 33 |
| H(10A) | 1274 | 2445 | 3337 | 30 |
| H(11A) | 1270 | 1573 | 3492 | 26 |
| H(12A) | -37 | 3202 | 2137 | 51 |
| H(12B) | 477 | 3229 | 3613 | 51 |
| H(12C) | 680 | 3194 | 1639 | 51 |

**2-((4-Fluorophenyl)sulfonyl)-1-methyl-*1H*-pyrrole (5bc):**[S7]

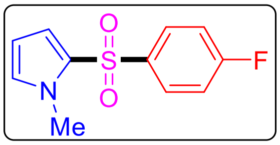


The titled compound was prepared following the general procedure, using 1-methyl-*1H*-pyrrole (**4b**, 0.5 mmol, 34.0 mg, 1.0 equiv.), 4-fluoroaniline (**2c**, 0.75 mmol, 80.0 mg; 1.5 equiv.), $^t$BuONO (1.0 mmol, 103.0 mg, 2.0 equiv.), DABSO (0.6 mmol, 144.0 mg, 1.2 equiv.), and $I_2$ (0.6 mmol, 152.0 mg; 1.2 equiv.) in MeCN (3.0 mL). The crude product purified by flash column chromatography ($SiO_2$, 8-10% ethyl acetate in hexanes) to provide **5bc** as brown solid, M. P.: 60 °C; yield: 72 mg, 60%. $^1$H NMR (400 MHz, DMSO-$d_6$): $\delta$ 7.96-7.92 (m, 2H), 7.48-7.43 (m, 2H),

7.16 (t, *J* = 2.4 Hz, 1H), 6.96 (dd, *J* = 4.0 & 2.0 Hz, 1H), 6.20 (dd, *J* = 4.0 & 2.8 Hz, 1H), 3.66 (s, 3H); $^{13}$C{H} NMR (100 MHz, DMSO-$d_6$): $\delta$ 164.6 (d, *J* = 251.0 Hz), 138.1, 131.0, 130.0 (d, *J* = 10.0 Hz), 127.1, 118.3, 116.9 (d, *J* = 23.0 Hz), 108.2, 35.2; $^{19}$F NMR (376 MHz, DMSO-$d_6$) $\delta$ -105.17.

**2-((4-Chlorophenyl)sulfonyl)-1-methyl-*1H*-pyrrole (5bd):**[S7]

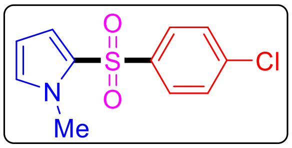


The titled compound was prepared following the general procedure, using 1-methyl-*1H*-pyrrole (**4b**, 0.5 mmol, 41.0 mg, 1.0 equiv.), 4-chloroaniline (**2d**, 0.75 mmol, 96.0 mg, 1.5 equiv.), $^t$BuONO (1.0 mmol, 103.0 mg, 2.0 equiv.), DABSO (0.6 mmol, 144.0 mg, 1.2 equiv.), and $I_2$ (0.6 mmol, 152.0 mg, 1.2 equiv.) in MeCN (3.0 mL). The crude product purified by flash column chromatography ($SiO_2$, 8-10% ethyl acetate in hexanes) to provide **5bd** as white solid, M. P.: 71-72 $^o$C; yield: 79.0 mg, 62%. $^1$H NMR (400 MHz, DMSO-$d_6$): $\delta$ 7.89-7.85 (m, 2H), 7.69-7.66 (m, 2H), 7.17 (t, *J* = 2.4 Hz, 1H), 6.98 (dd, *J* = 4.0 & 2.0 Hz, 1H), 6.20 (dd, *J* = 4.0 & 2.8 Hz, 1H), 3.67 (s, 3H); $^{13}$C{H} NMR (100 MHz, DMSO-$d_6$): $\delta$ 140.6, 138.3, 131.3, 129.8, 128.7, 126.7, 118.6, 108.3, 35.2.

**EPR studies**

A flame-dried 10 mL Schlenk tube was charged with **1a** (0.5 mmol), DABSO (0.6 mmol), aniline **2a** (0.75 mmol), $^t$BuONO (1.0 mmol), $I_2$ (0.6 mmol), 5,5-dimethyl-1-pyrroline N-oxide (DMPO, 1.5 mmol), and acetonitrile (3 mL) under a nitrogen atmosphere. The reaction mixture was stirred at room temperature for 30 min to 12 h. Aliquots were withdrawn after 30 min and 2 h and analyzed by EPR spectroscopy at 298 K under a nitrogen atmosphere. The EPR spectra revealed a mixture of two radical species with g = 2.00701, $\alpha$N = 14.2347 G and $\alpha$H = 21.4055 G, and g = 2.00714, $\alpha$N = 13.2924 G and $\alpha$H = 12.2326 G. These signals were attributed to the DMPO–Ph and DMPO–$SO_2$ radical adducts, respectively (Figure S1-S4). Furthermore, FTMS+ESI analysis corroborated the formation of DMPO–Ph and DMPO–$SO_2$Ph radical adducts in the reaction mixture (Figure S5-S6).

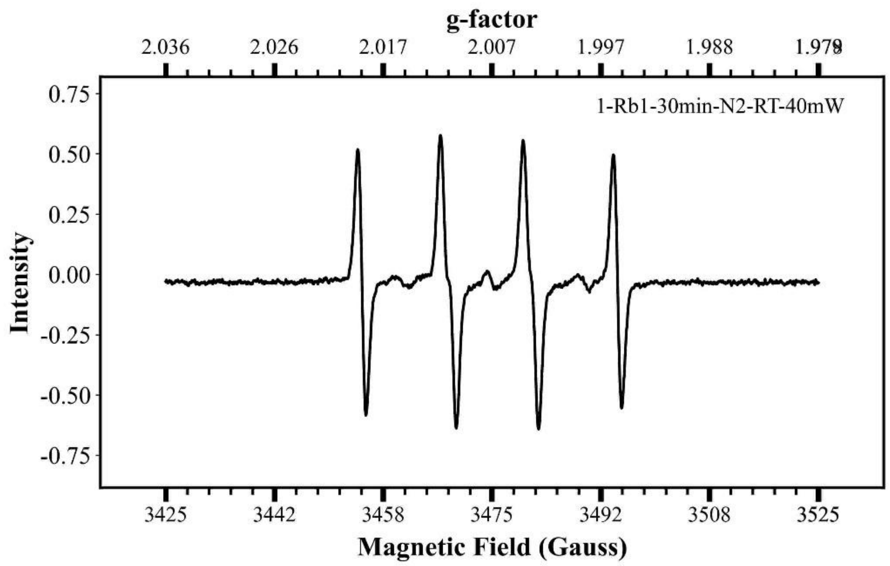


**Figure S1**. EPR spectrum of the reaction mixture recorded after 30 min under a nitrogen atmosphere, showing signals attributable to the DMPO–$SO_2^{\bullet}$ adduct, consistent with the proposed sulfonyl radical intermediate.

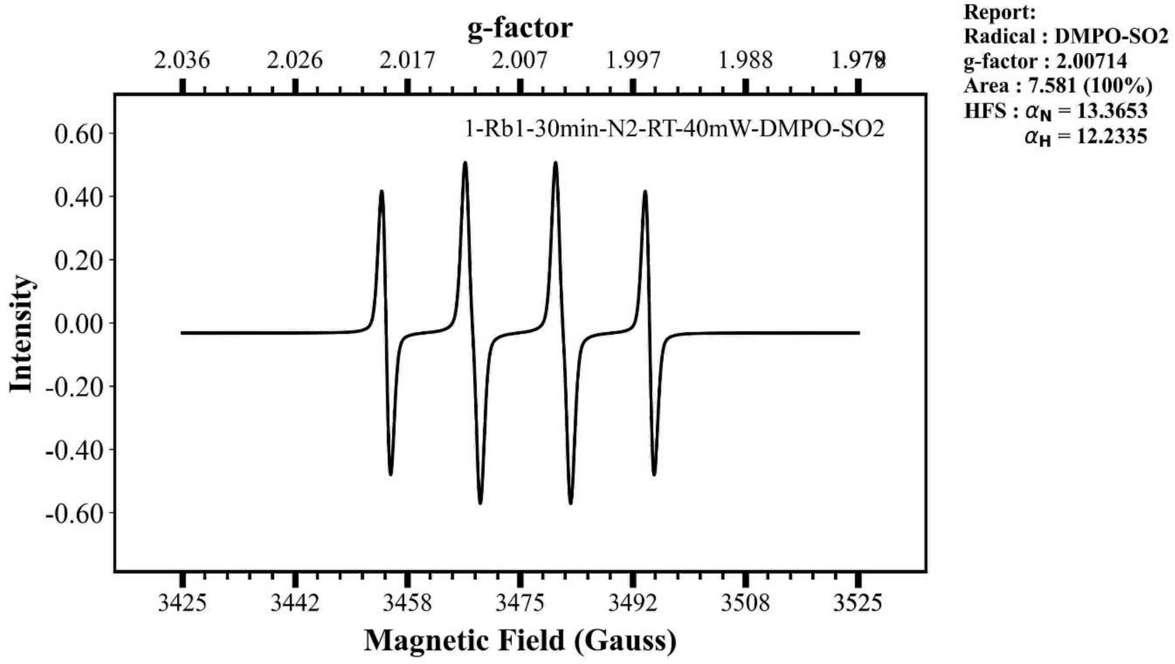


**Figure S2.** Figure S2. Simulated EPR spectrum of the DMPO–$SO_2$• adduct, corresponding to the experimental spectrum shown in Figure S1.

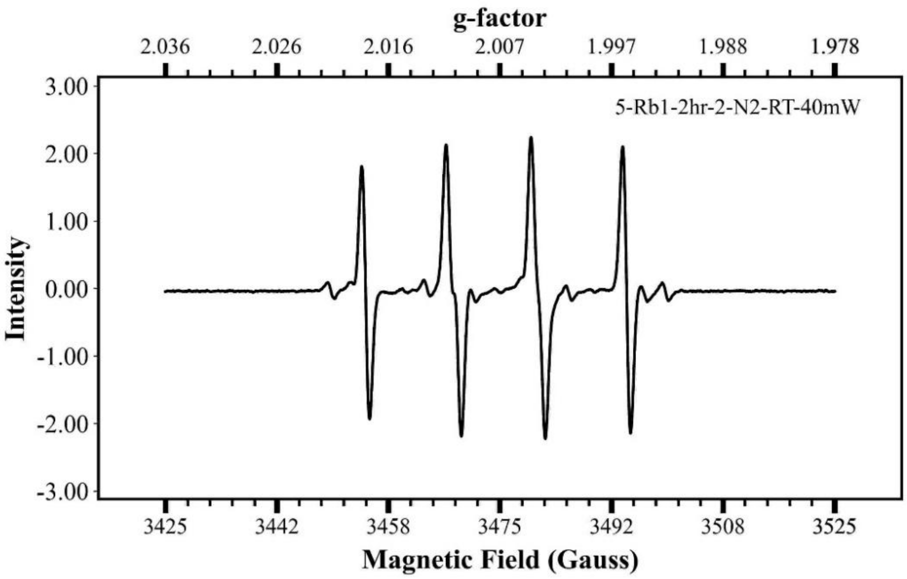


**Figure S3.** Figure S3. EPR spectrum of the reaction mixture recorded after 2 h under a nitrogen atmosphere, showing signals corresponding to both the DMPO–$SO_2$• and DMPO–Ph• adducts.

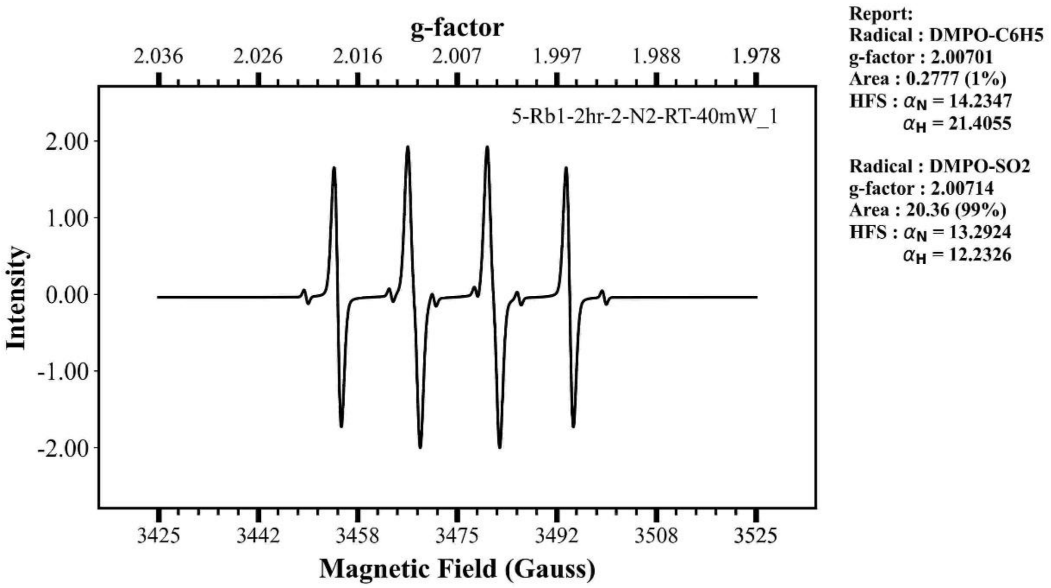


**Figure S4** Simulated EPR spectrum corresponding to the experimental spectrum shown in Figure S3, attributed to the DMPO–$SO_2$•$^-$ and DMPO–Ph• adducts.

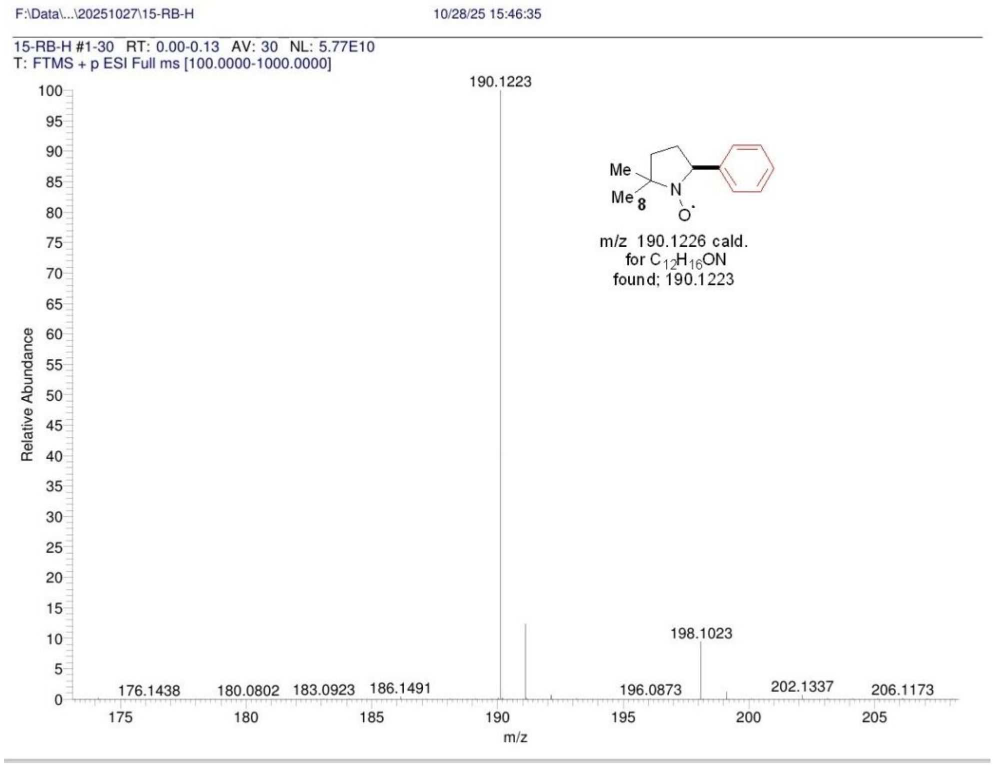


**Figure 5:** High-resolution FTMS+ ESI full mass spectrum of DMPO-Ph adduct.

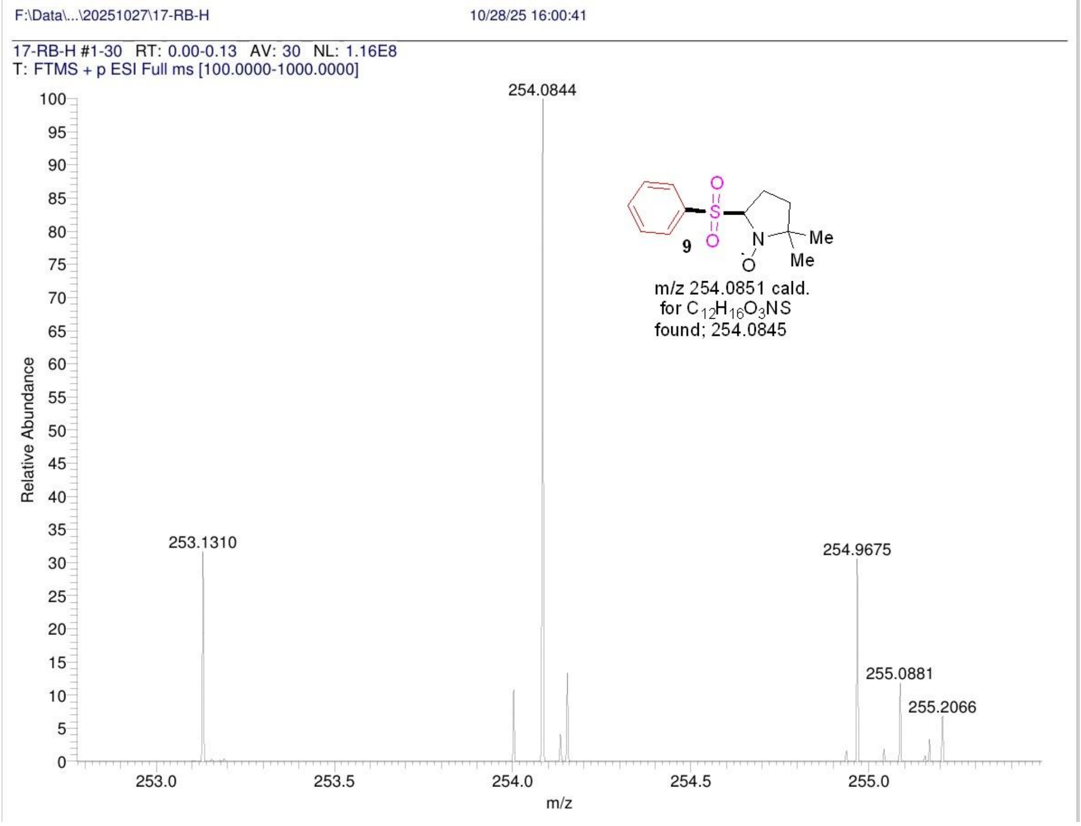


**Figure 6:** High-resolution FTMS+ ESI full mass spectrum of DMPO-$SO_2$Ph adduct.

**References:**

S1. Katrun, P.; Mueangkaew, C.; Pohmakotr, M.; Reutrakul, V.; Jaipetch, T.; Soorukram, D.; Kuhakarn, C. Regioselective C 2 Sulfonylation of Indoles Mediated by Molecular Iodine. *J. Org. Chem.* **2014**, *79*, 1778-1785.

S2. Xiao, F.; Chen, H.; Xie, H.; Chen, S.; Yang, L.; Deng, G.-J. Iodine-catalyzed regioselective 2-sulfonylation of indoles with sodium sulfinates. *Org. Lett.* **2014**, *16*, 50-53.

S3. Yang, Y.; Li, W.; Xia, C.; Ying, B.; Shen, C.; Zhang, P. Catalyst-Controlled Selectivity in C−S Bond Formation: Highly Efficient Synthesis of C2-and C3-Sulfonylindoles. *ChemCatChem* **2016**, *8*, 304–307.

S4. Park, H.; Bae, J.; Son, S.; Jang, H.-Y. Multifunctionalization of Indoles: Synthesis of 3-Iodo-2-sulfonyl Indoles. *Bull. Korean Chem. Soc.* **2019**, *40*, 1128–1133.

S5. Li, H.; Wang, X.; Yan, J. Convenient KI-catalyzed regioselective synthesis of 2-sulfonylindoles using water as solvent. *New J. Chem.* **2017**, *41* (11), 4277–4280.

S6. Yadav, J. S.; Reddy, B. V. S.; Kondaji, G.; Srinivasa Rao, R.; Praveen Kumar, S. Zinc-mediated acylation and sulfonation of pyrrole and its derivatives. *Tetrahedron Lett.* **2002**, *43*, 8133–8135.

S7. Zhang, J.; Wang, Z.; Chen, L.; Liu, Y.; Liu, P.; Dai, B. The fast and efficient KI/H 2 O 2 mediated 2-sulfonylation of indoles and *N*-methylpyrrole in water. *RSC Adv.* **2018**, *8*, 41651–41656.

S8. Pagire, S. K.; Hossain, A.; Reiser, O. Temperature controlled selective C–S or C–C bond formation: photocatalytic sulfonylation versus arylation of unactivated heterocycles utilizing aryl sulfonyl chlorides. *Org. Lett.*, **2018**, 20, 648-651.

S9. Xiong, Y.; Zhang, Q.; Zhang, J.; Wu, X. Visible-light-driven deoxygenative heteroarylation of alcohols with heteroaryl sulfones. *J. Org. Chem.*, **2024**, *89*, 3629-3634.

S10. Wu, Y. X.; Liu, Q.; Zhang, Q.; Ye, Z.; He, Y. Asymmetric allylic substitution-isomerization for accessing axially chiral vinylindoles by intramolecular π-π stacking interactions. *Cell Reports Physical Science*, **2022**, *3*, 101005.

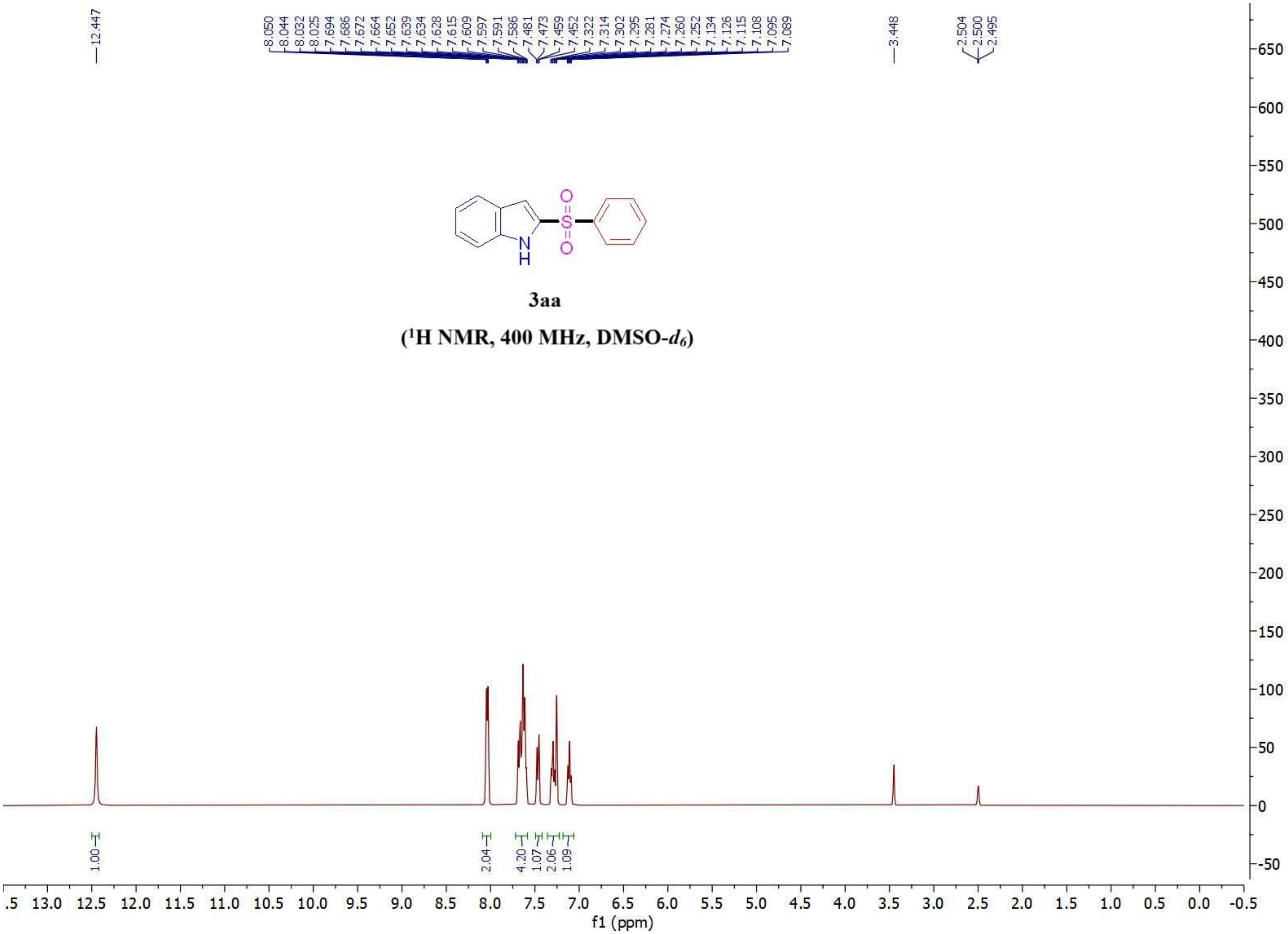
12.447
8.050
8.044
8.032
8.025
7.694
7.686
7.672
7.664
7.652
7.639
7.634
7.628
7.615
7.609
7.597
7.591
7.586
7.481
7.473
7.459
7.452
7.322
7.314
7.302
7.295
7.281
7.274
7.260
7.252
7.134
7.126
7.115
7.108
7.095
7.089
3.448
2.504
2.500
2.495
3aa
(¹H NMR, 400 MHz, DMSO-d6)
1.00
2.04
4.20
1.07
2.06
1.09
f1 (ppm)

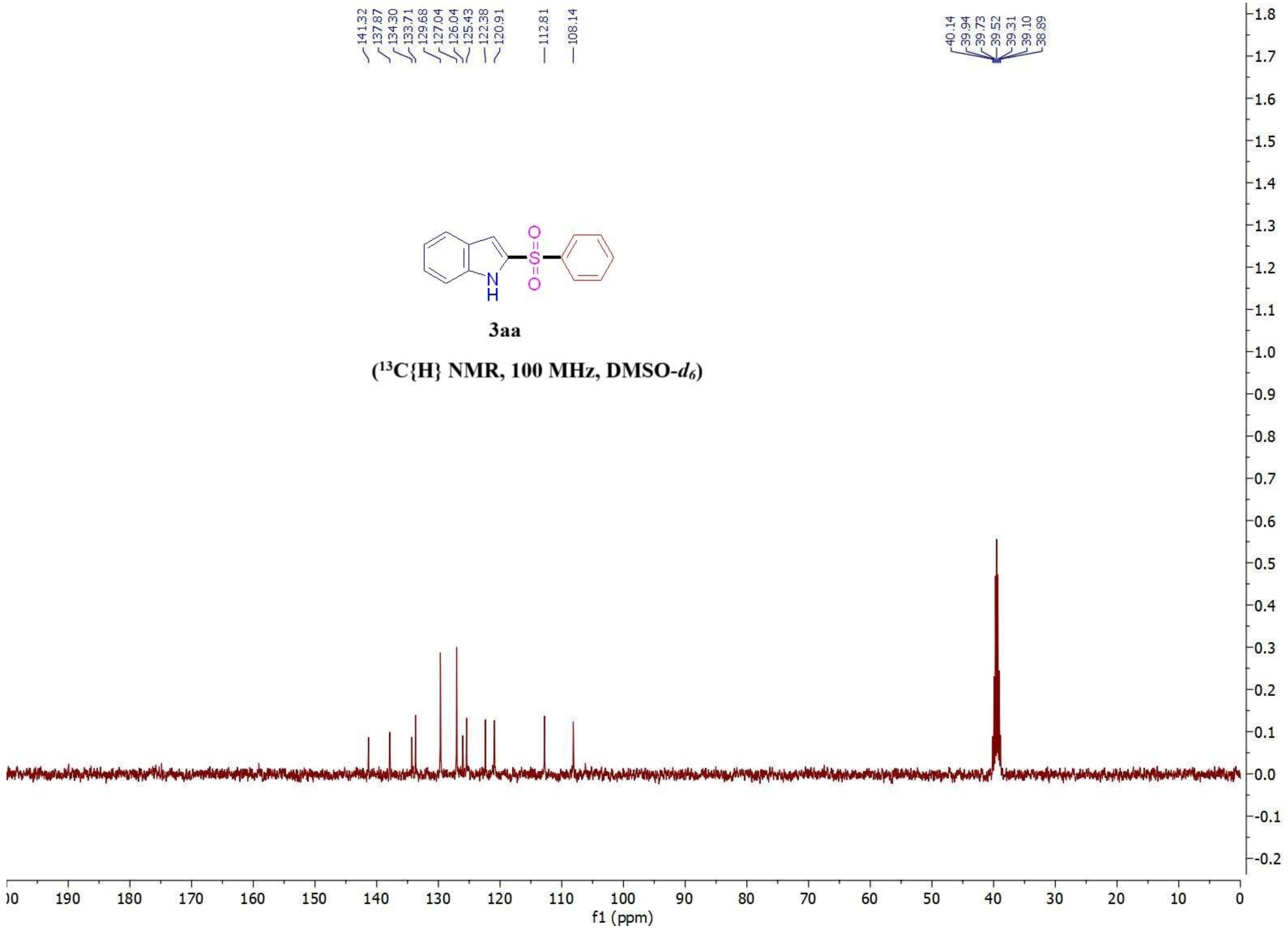


**3aa**

**($^{13}$C{H} NMR, 100 MHz, DMSO-$d_6$)**

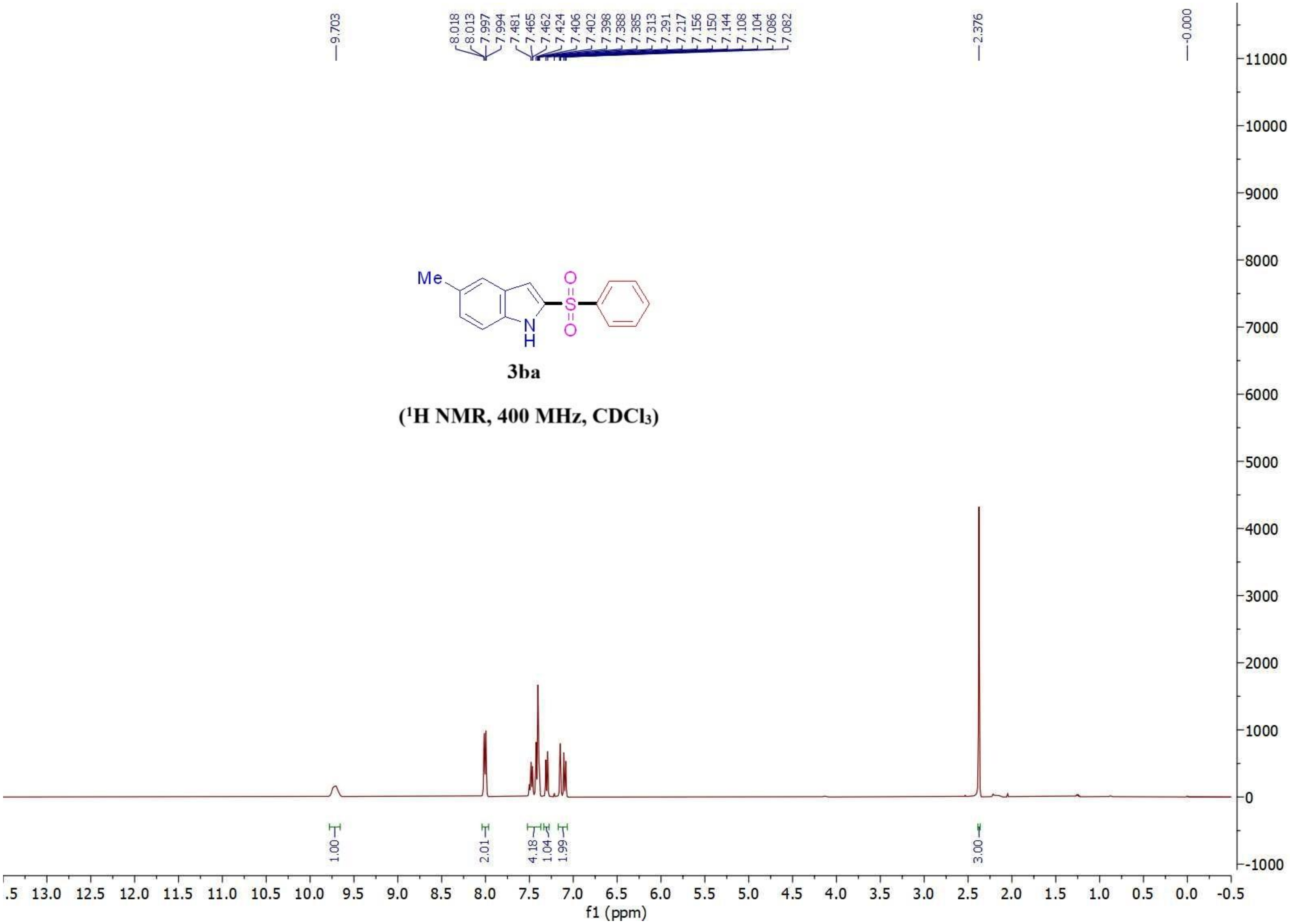
9.703
8.018
8.013
7.997
7.994
7.481
7.465
7.462
7.424
7.406
7.402
7.398
7.388
7.385
7.313
7.291
7.217
7.156
7.150
7.144
7.108
7.104
7.086
7.082
2.376
-0.000
Me
O
S
O
N
H
3ba
($^1$H NMR, 400 MHz, CDCl$_3$)
1.00
2.01
4.18
1.04
1.99
3.00
f1 (ppm)

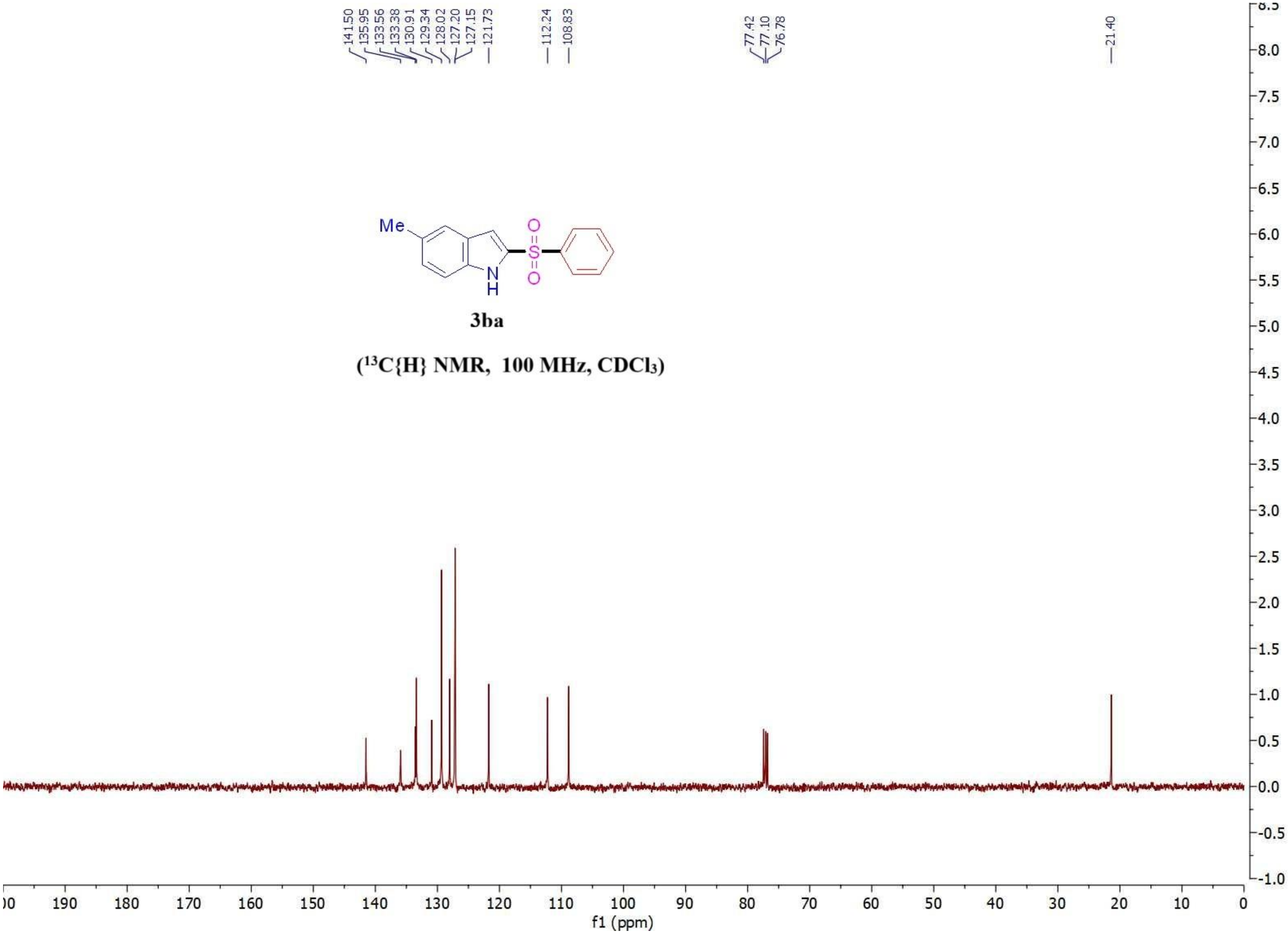


3ba

($^{13}C\{H\}$ NMR, 100 MHz, $CDCl_3$)

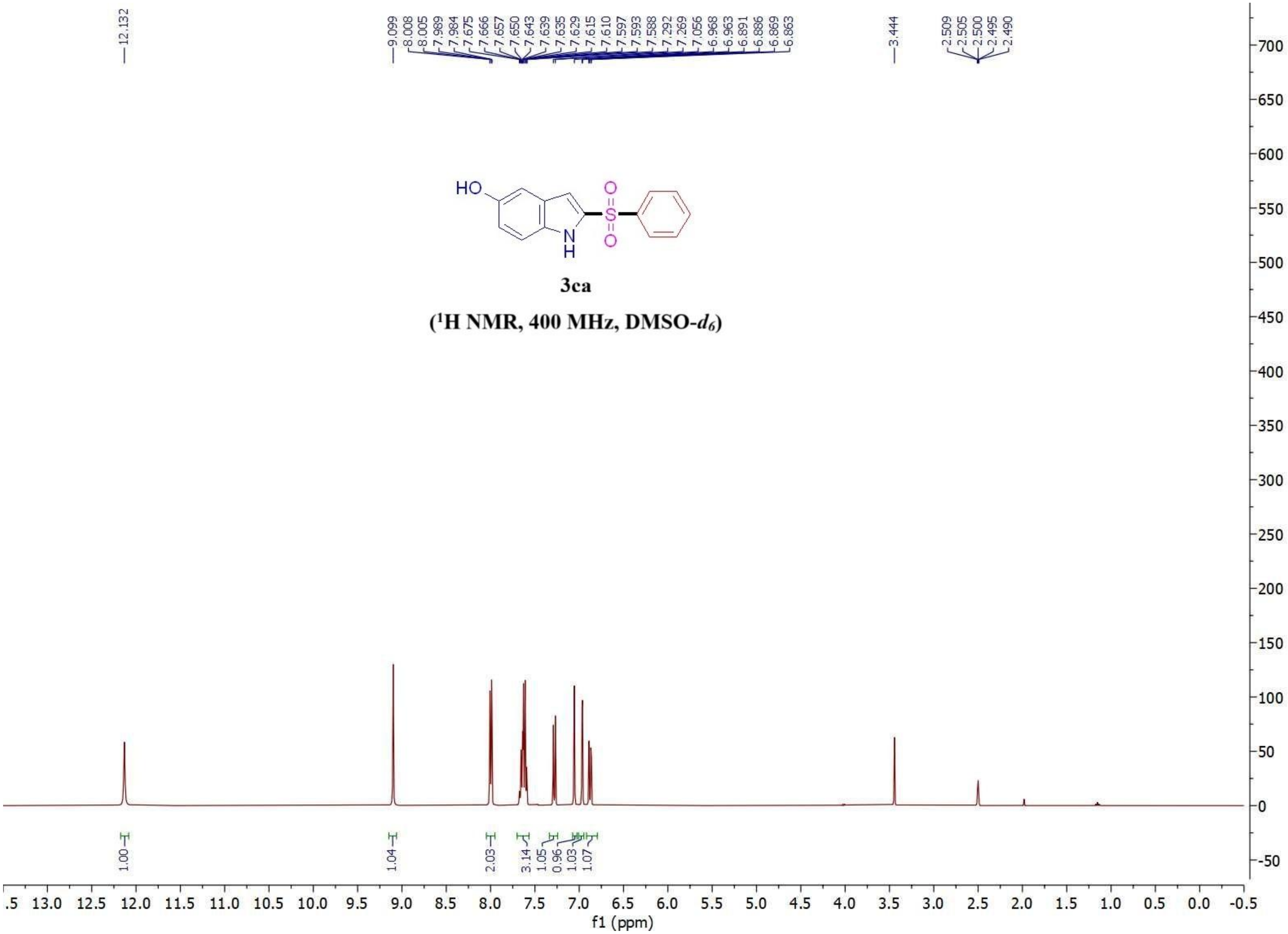

3ca

($^1$H NMR, 400 MHz, DMSO-$d_6$)

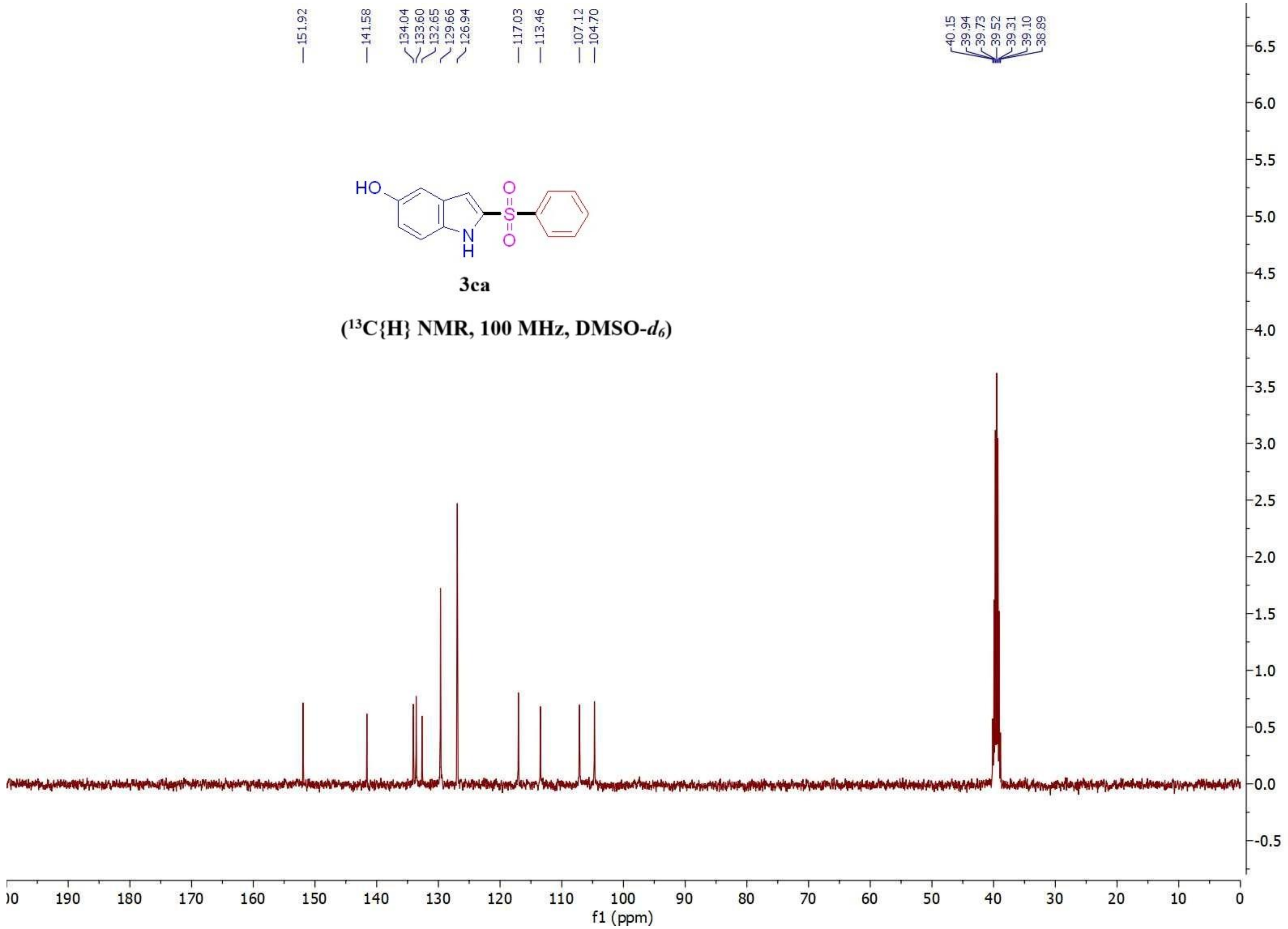


**3ca**

**($^{13}$C{H} NMR, 100 MHz, DMSO-$d_6$)**

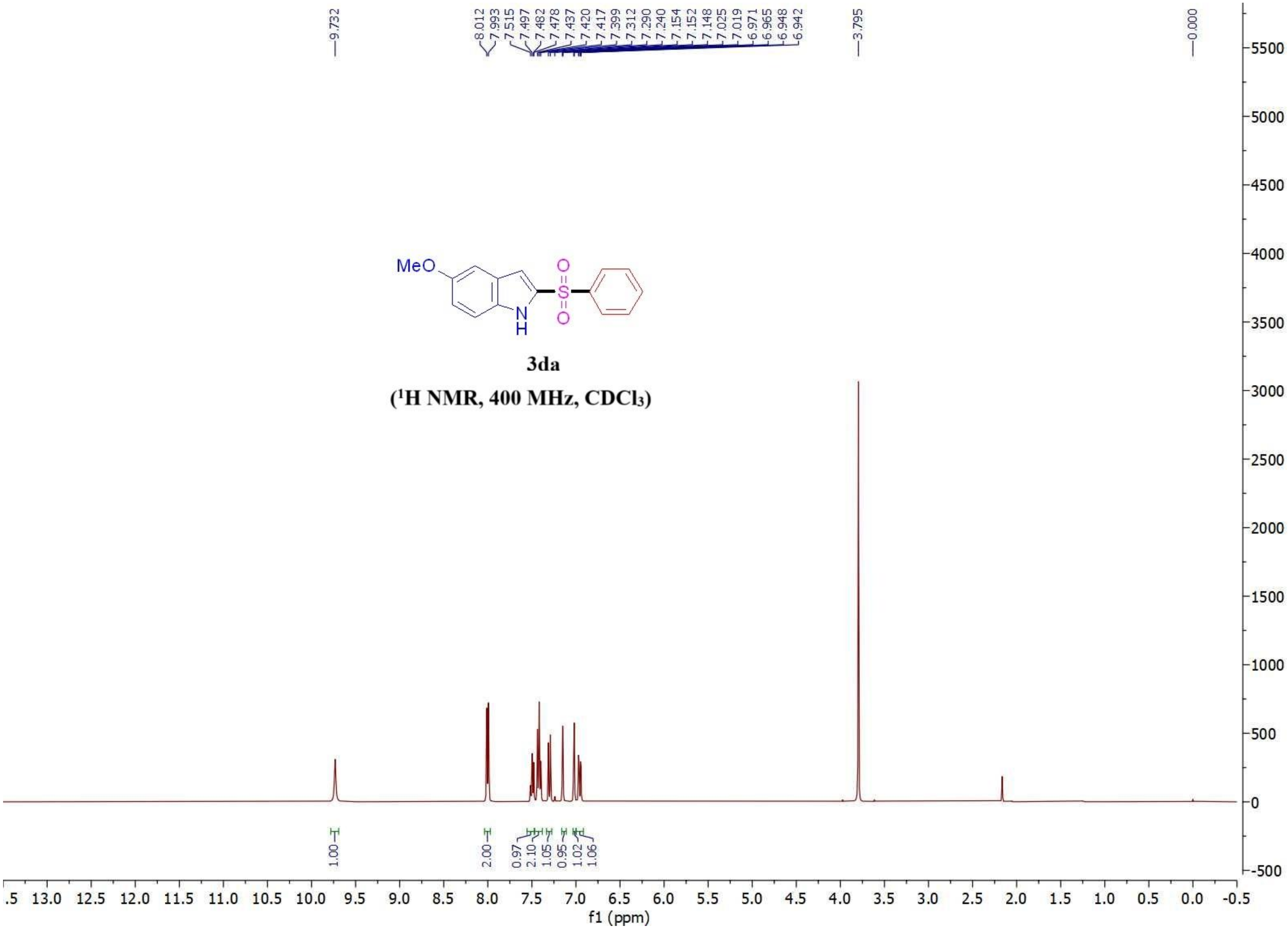


**3da**

**($^1$H NMR, 400 MHz, CDCl$_3$)**

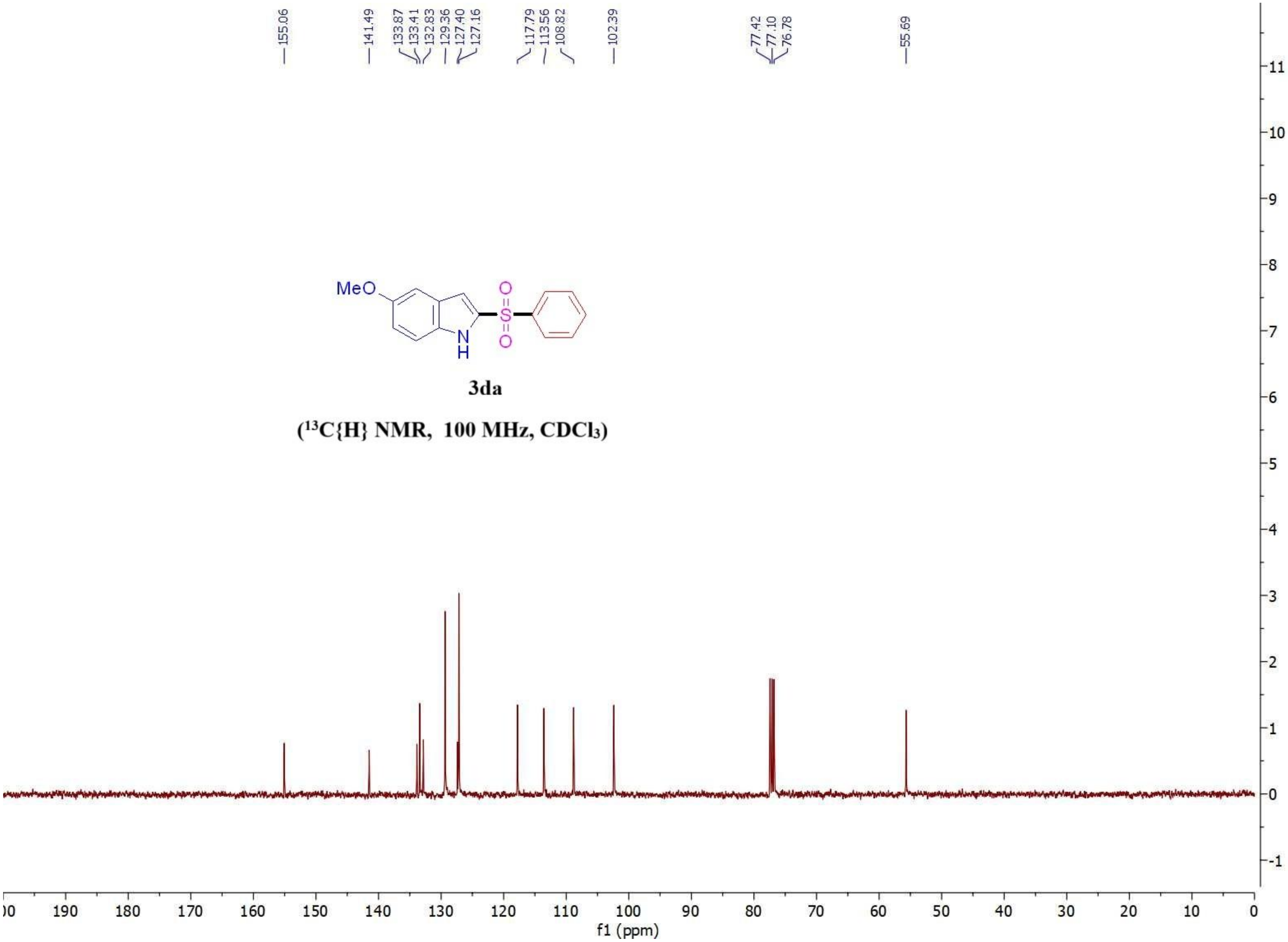


**3da**

**($^{13}$C{H} NMR, 100 MHz, $CDCl_3$)**

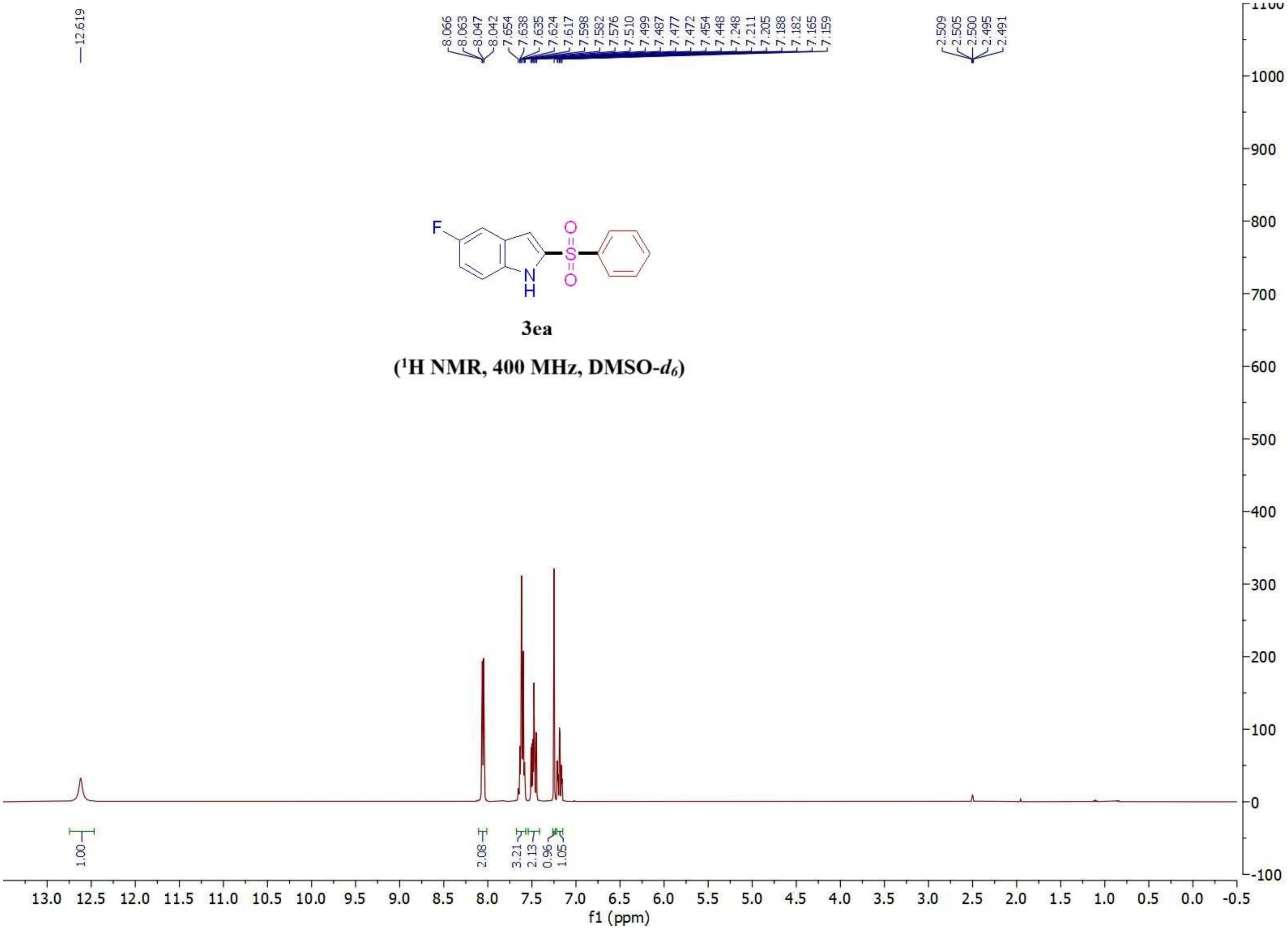


**3ea**

**($^1$H NMR, 400 MHz, DMSO-$d_6$)**

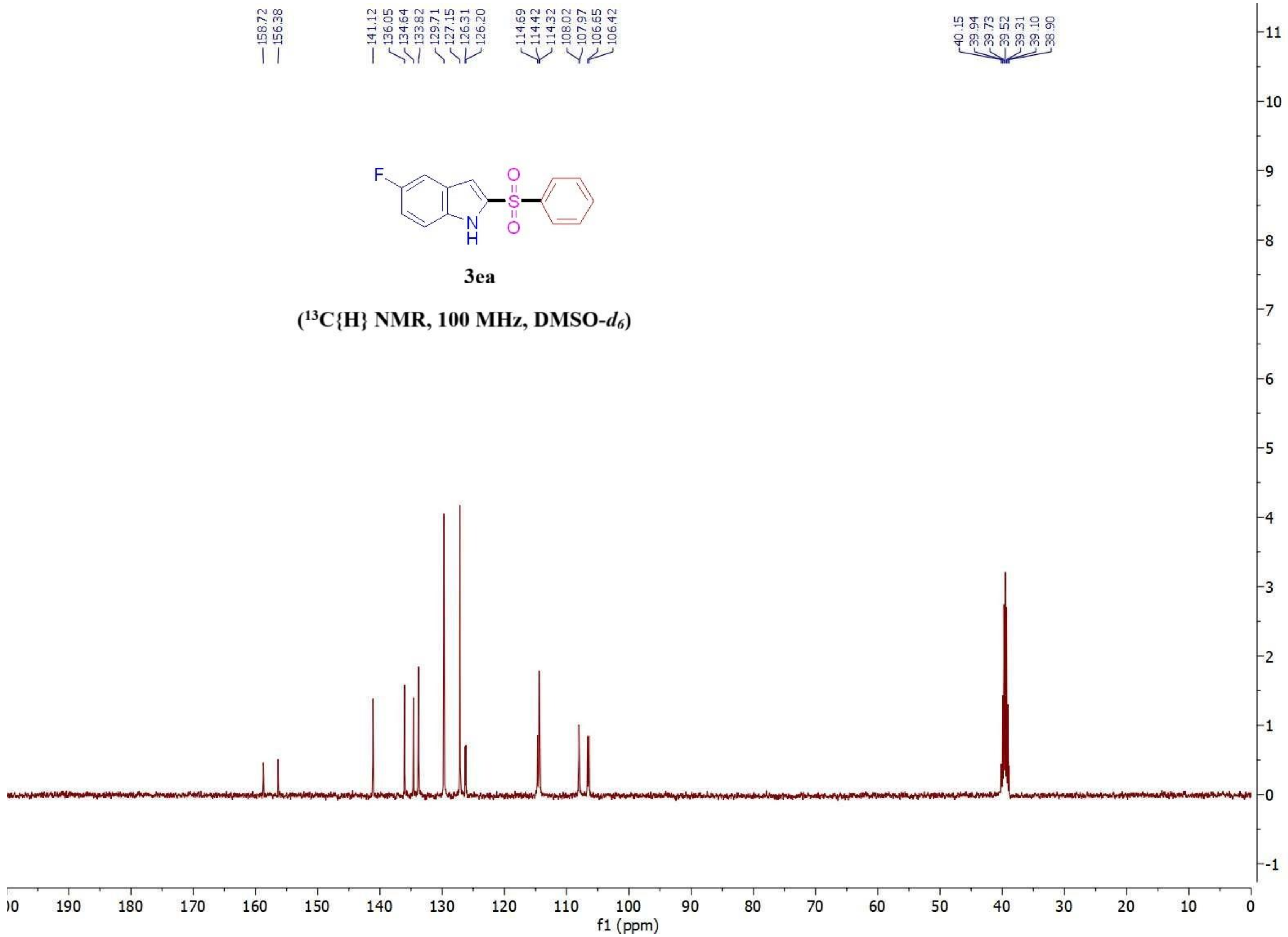
158.72
156.38
141.12
136.05
134.64
133.82
129.71
127.15
126.31
126.20
114.69
114.42
114.32
108.02
107.97
106.65
106.42
40.15
39.94
39.73
39.52
39.31
39.10
38.90
F
N
H
O
S
O
3ea
($^{13}$C{H} NMR, 100 MHz, DMSO-$d_6$)
f1 (ppm)

**3ea**

**($^{19}$F NMR, 376 MHz, DMSO-$d_6$)**

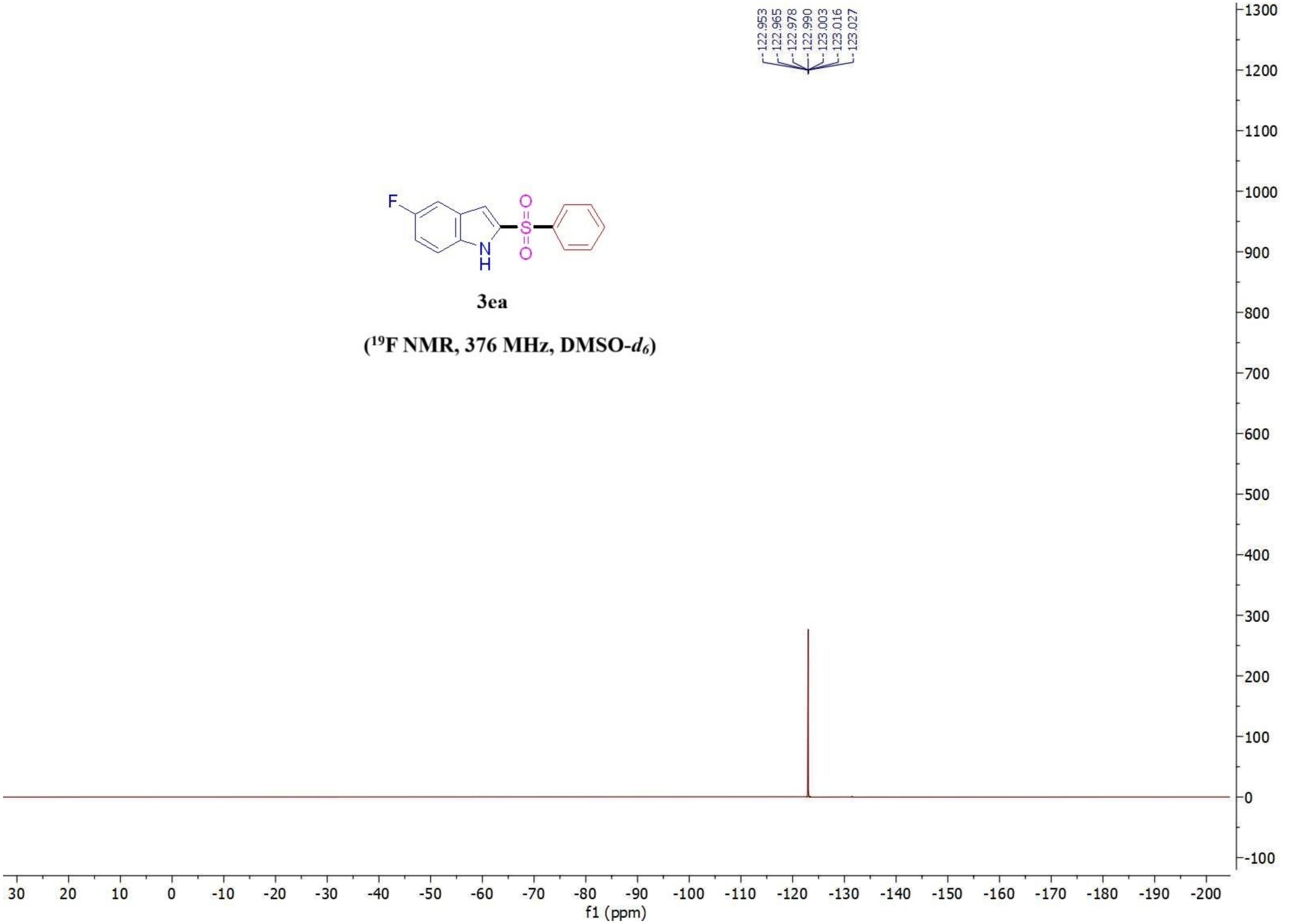

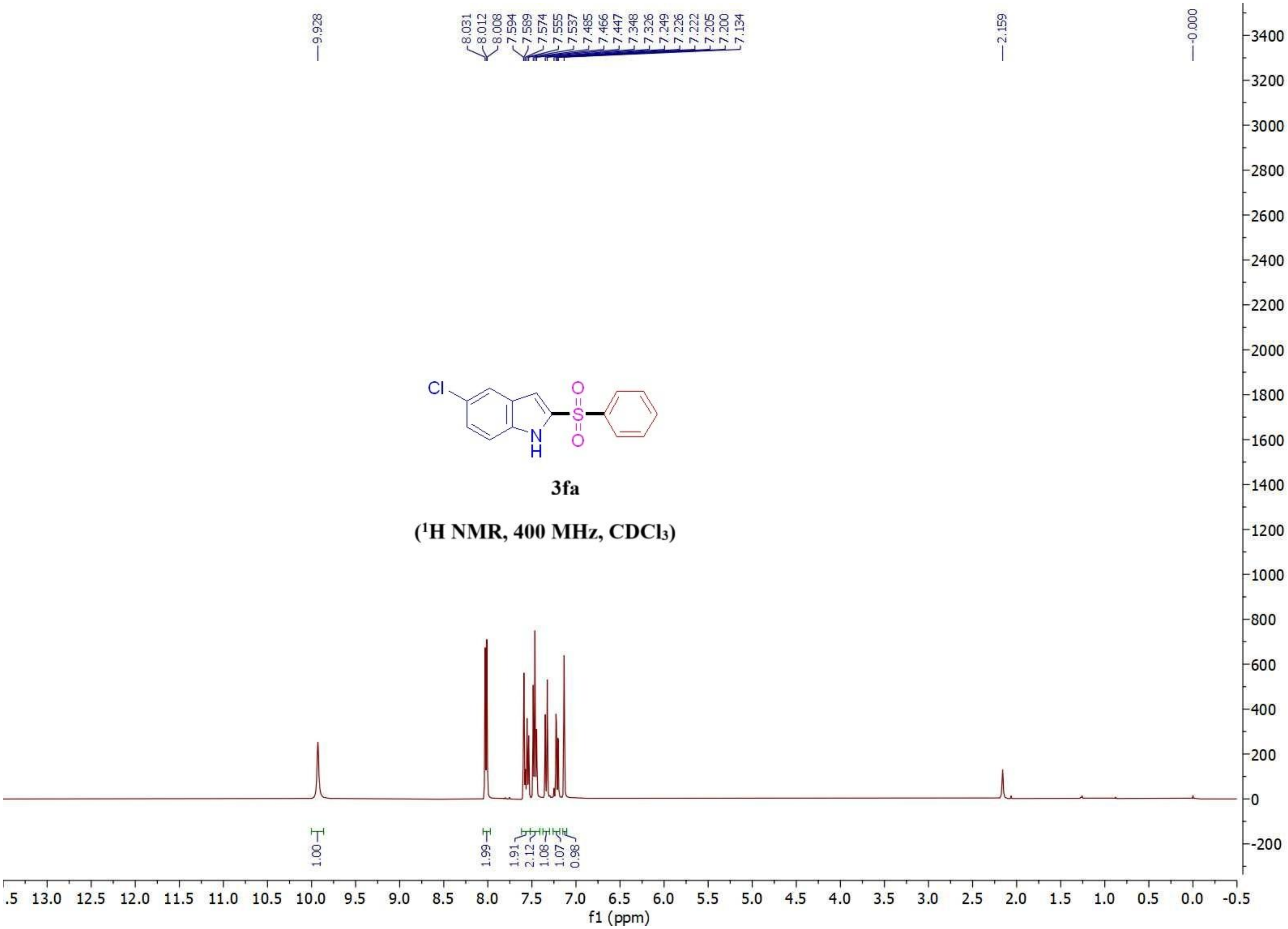


**3fa**

**($^1$H NMR, 400 MHz, $CDCl_3$)**

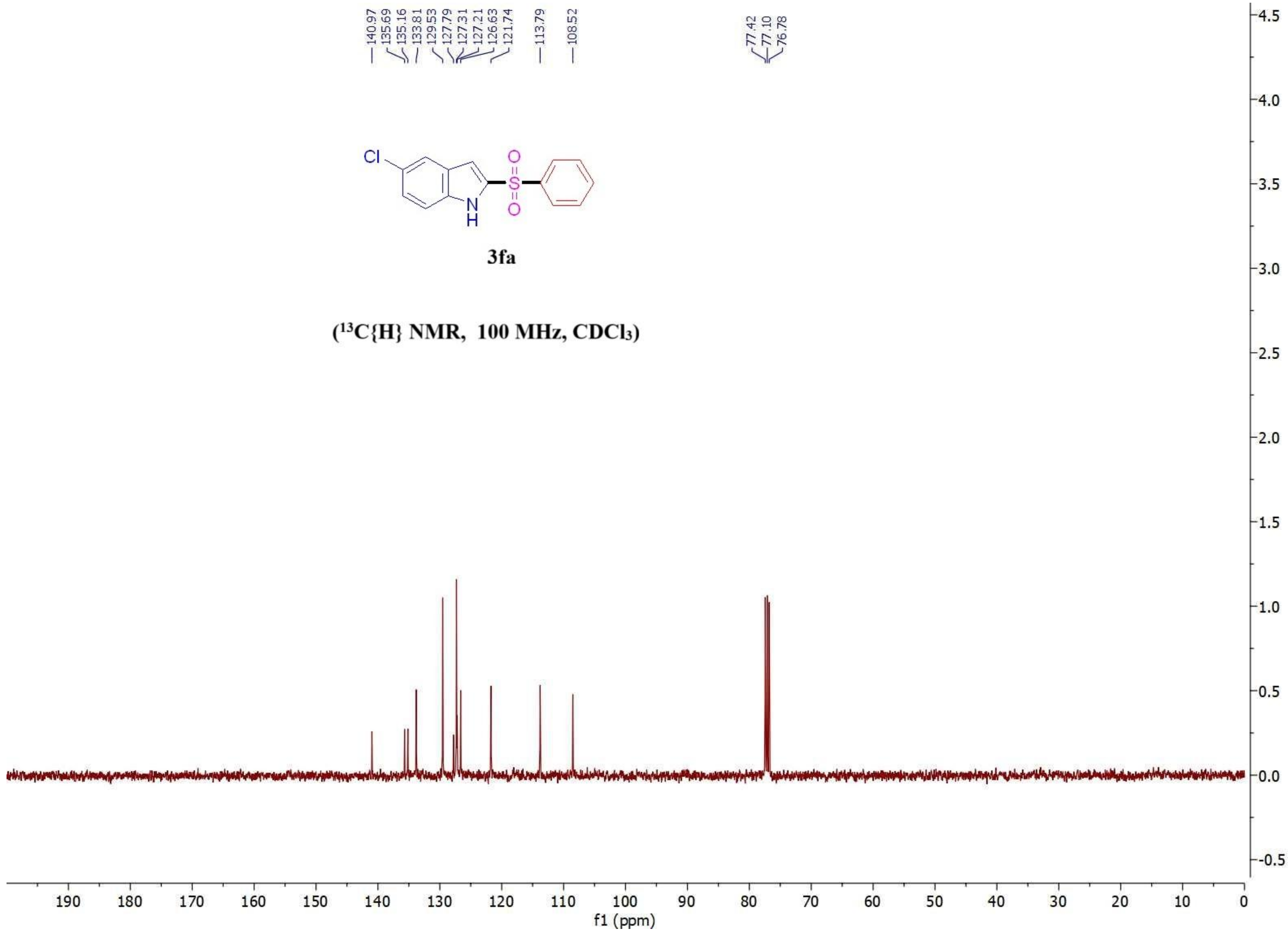
140.97
135.69
135.16
133.81
129.53
127.79
127.31
127.21
126.63
121.74
113.79
108.52
77.42
77.10
76.78
Cl
O
S
O
N
H
3fa
($^{13}$C{H} NMR, 100 MHz, CDCl$_3$)
f1 (ppm)

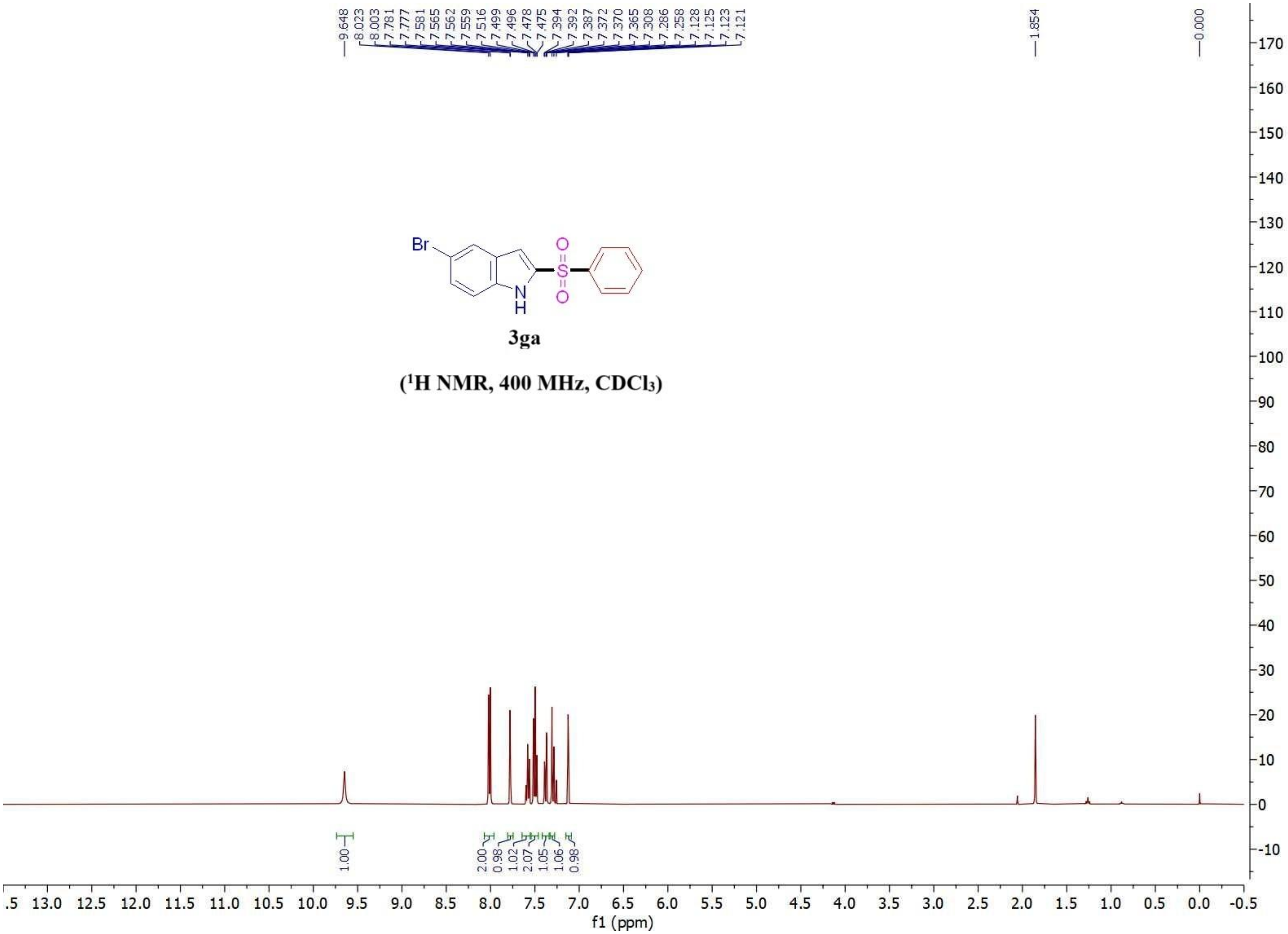


**3ga**

**($^1$H NMR, 400 MHz, $CDCl_3$)**

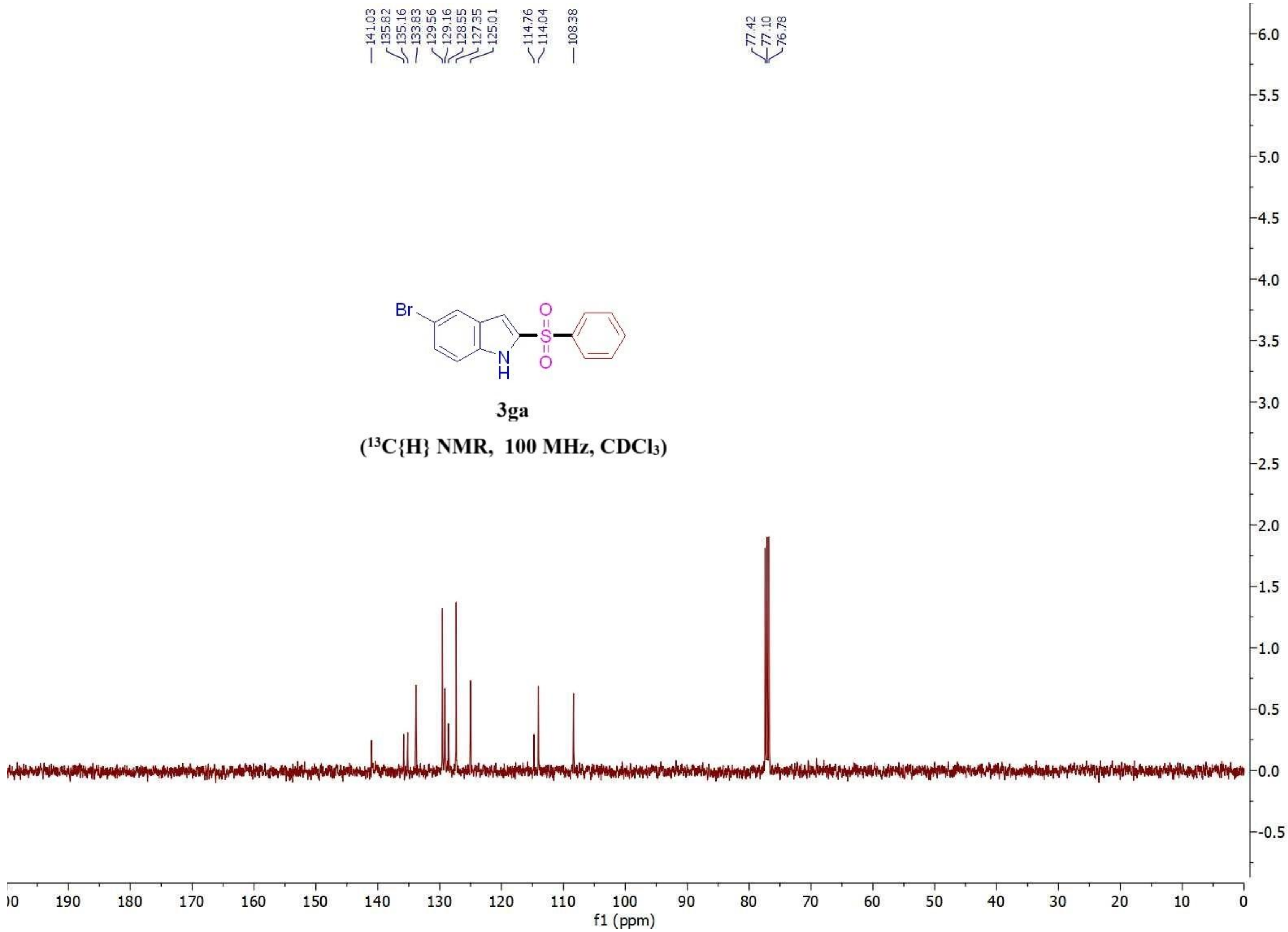


**3ga**

**($^{13}$C{H} NMR, 100 MHz, $CDCl_3$)**

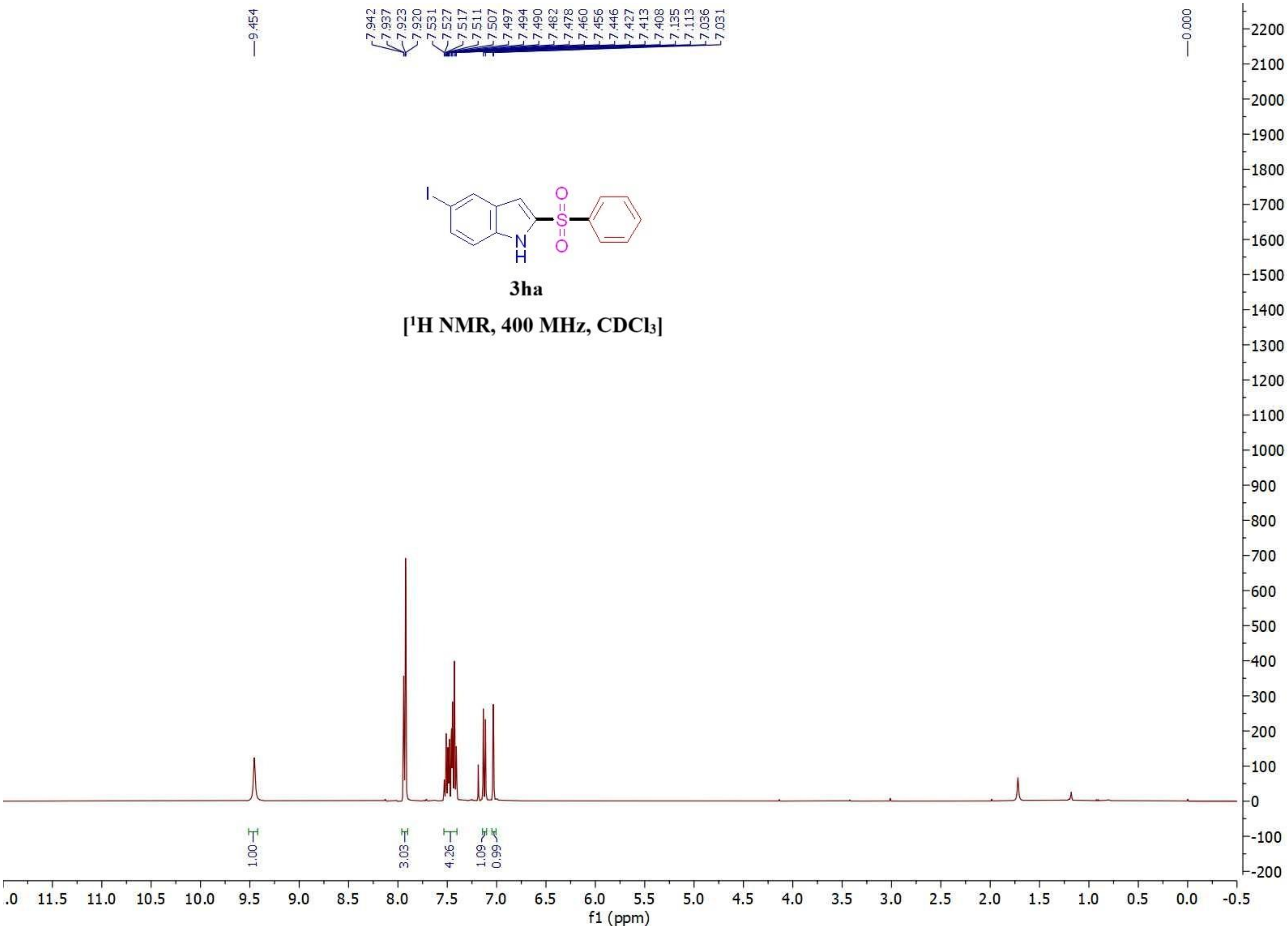

9.454
7.942 7.937 7.923 7.920 7.531 7.527 7.517 7.511 7.507 7.497 7.494 7.490 7.482 7.478 7.460 7.456 7.446 7.427 7.413 7.408 7.135 7.113 7.036 7.031
0.000
3ha
[1H NMR, 400 MHz, CDCl3]
1.00
3.03
4.26
1.09
0.99
f1 (ppm)

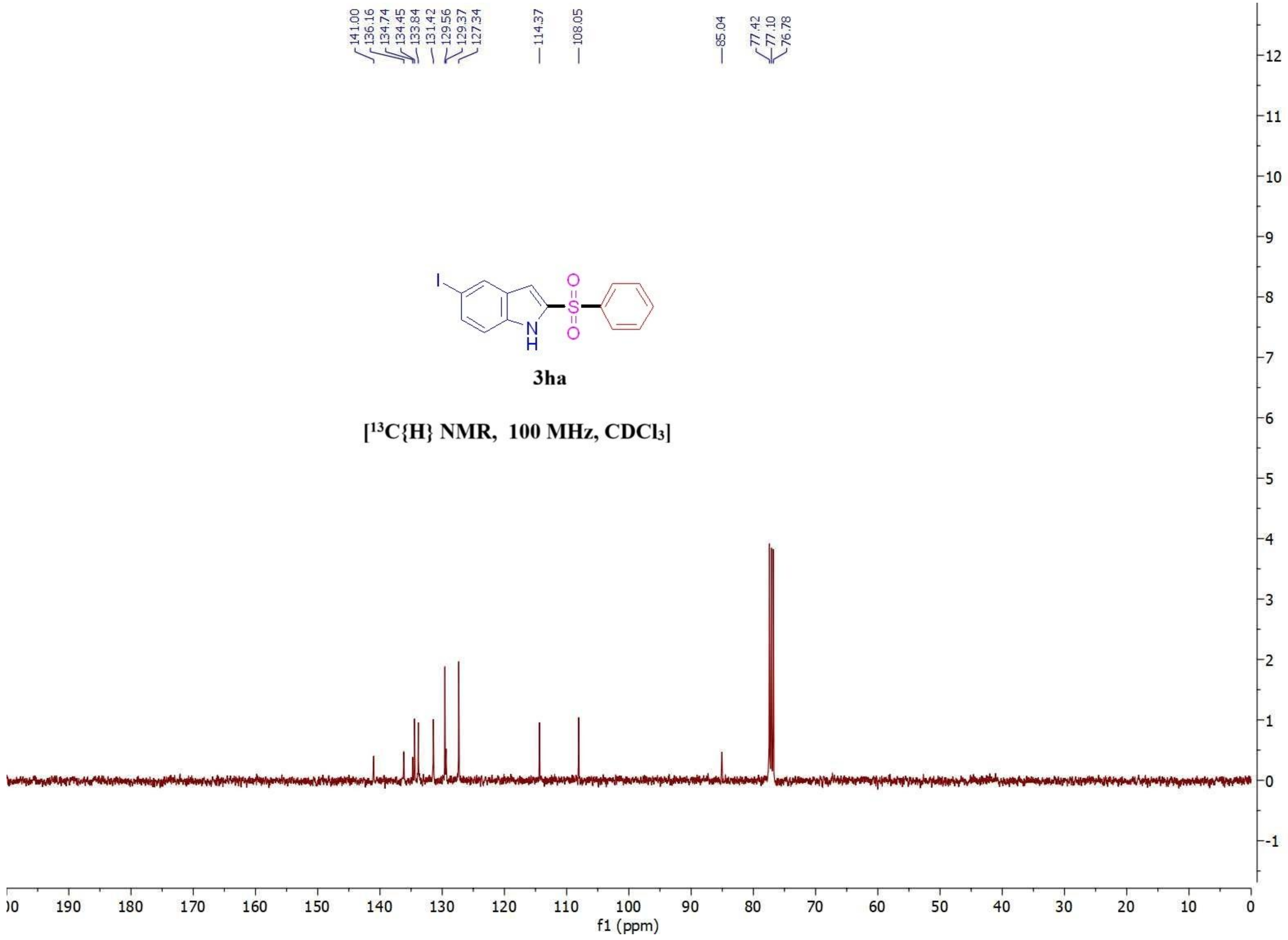
141.00
136.16
134.74
134.45
133.84
131.42
129.56
129.37
127.34
114.37
108.05
85.04
77.42
77.10
76.78
3ha
[13C{H} NMR, 100 MHz, CDCl3]
f1 (ppm)

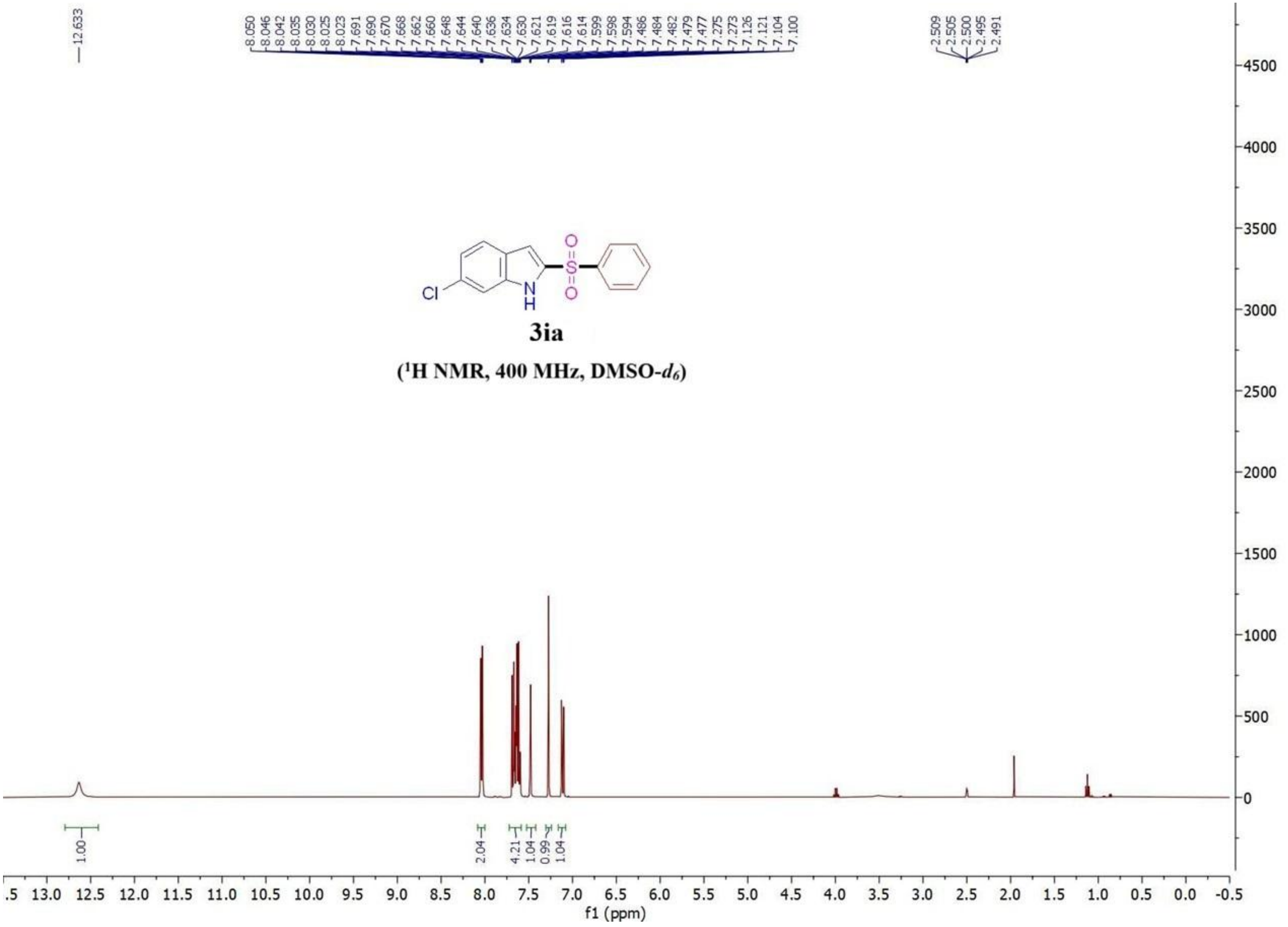
12.633
8.050
8.046
8.042
8.035
8.030
8.025
8.023
7.691
7.690
7.670
7.668
7.662
7.660
7.648
7.644
7.640
7.636
7.634
7.630
7.621
7.619
7.616
7.614
7.599
7.598
7.594
7.486
7.484
7.482
7.479
7.477
7.275
7.273
7.126
7.121
7.104
7.100
2.509
2.505
2.500
2.495
2.491
Cl
N
H
O
S
O
3ia
(1H NMR, 400 MHz, DMSO-d6)
1.00
2.04
4.21
1.04
0.99
1.04
f1 (ppm)

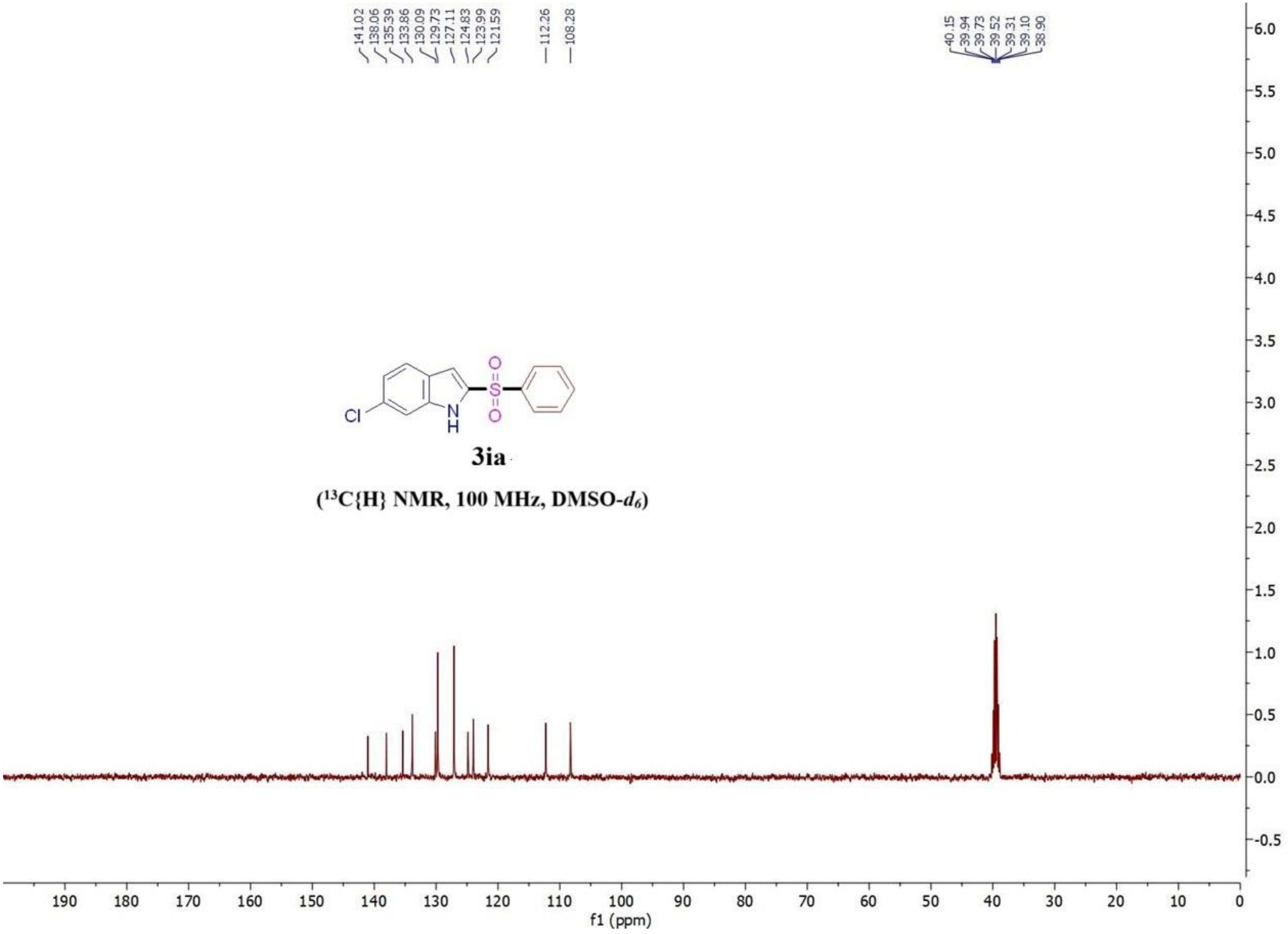


**3ia**

($^{13}$C{H} NMR, 100 MHz, DMSO-$d_6$)

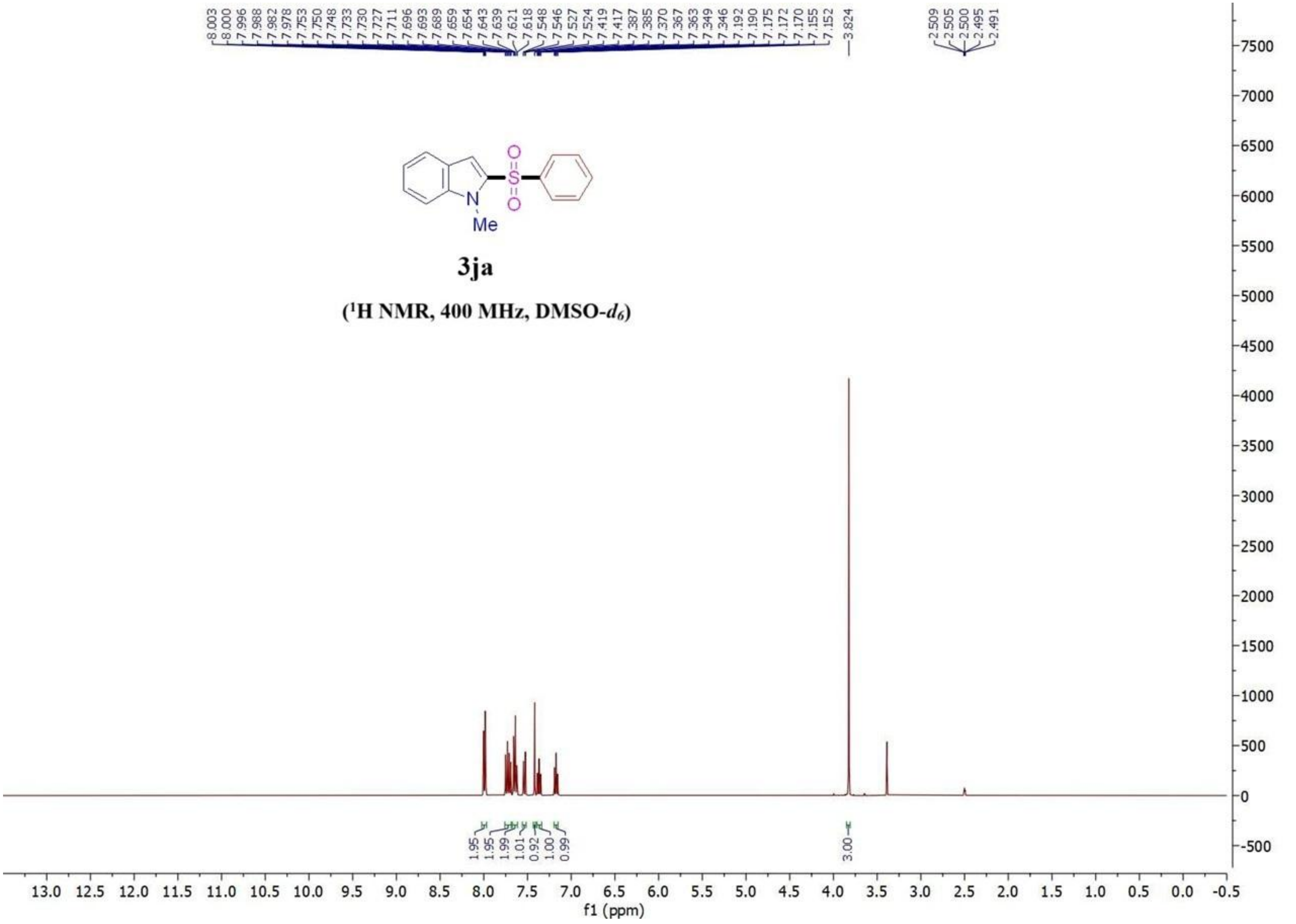
8.003
8.000
7.996
7.988
7.982
7.978
7.753
7.750
7.748
7.733
7.730
7.727
7.711
7.696
7.693
7.689
7.659
7.654
7.643
7.639
7.621
7.618
7.548
7.546
7.527
7.524
7.419
7.417
7.387
7.385
7.370
7.367
7.363
7.349
7.346
7.192
7.190
7.175
7.172
7.170
7.155
7.152
3.824
2.509
2.505
2.500
2.495
2.491
O
S
O
N
Me
3ja
(¹H NMR, 400 MHz, DMSO-d6)
1.95
1.95
1.99
1.01
0.92
1.00
0.99
3.00
f1 (ppm)

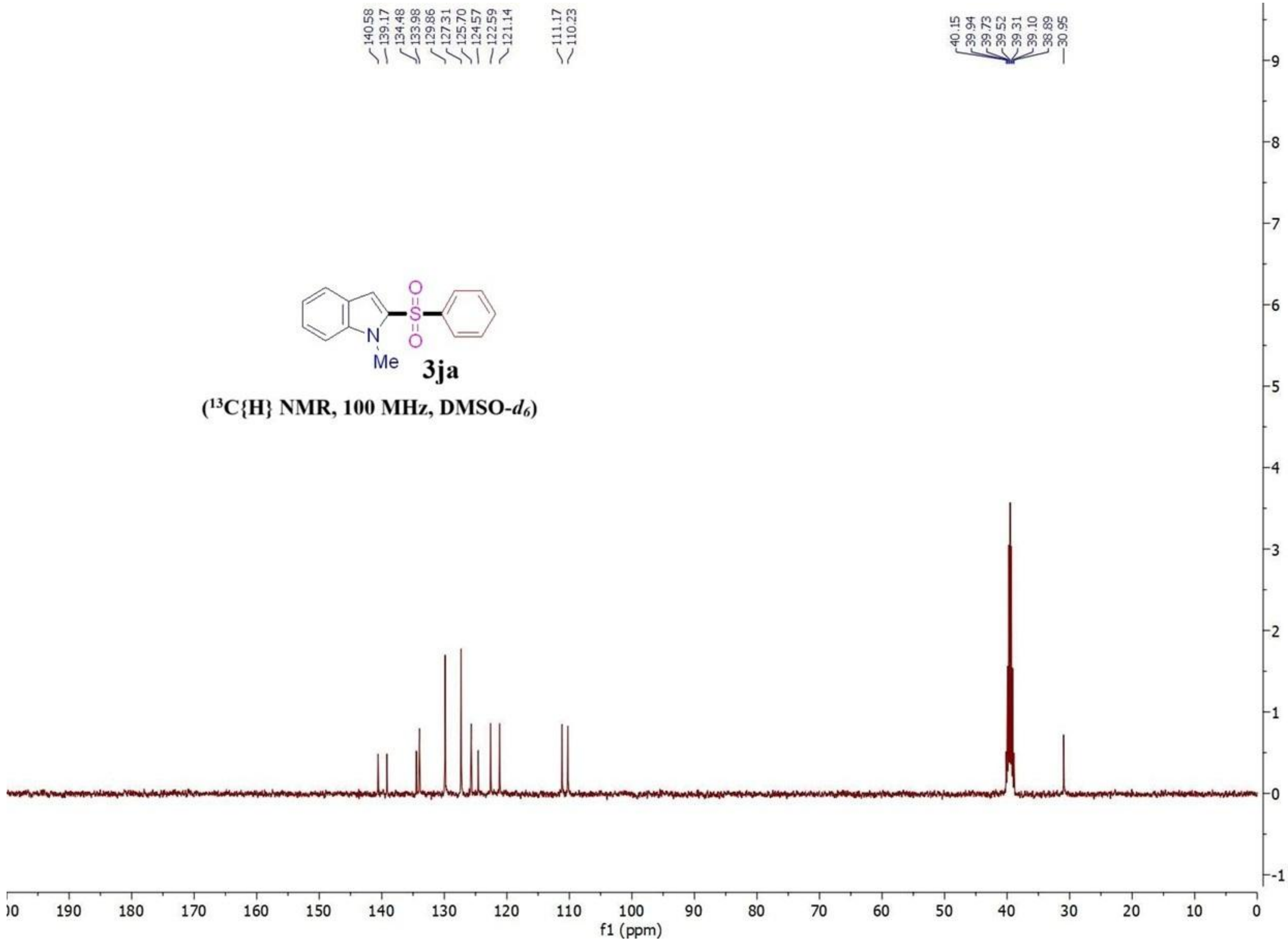
140.58
139.17
134.48
133.98
129.86
127.31
125.70
124.57
122.59
121.14
111.17
110.23
40.15
39.94
39.73
39.52
39.31
39.10
38.89
30.95
N
Me
S
O
O
3ja
(13C{H} NMR, 100 MHz, DMSO-d6)
f1 (ppm)

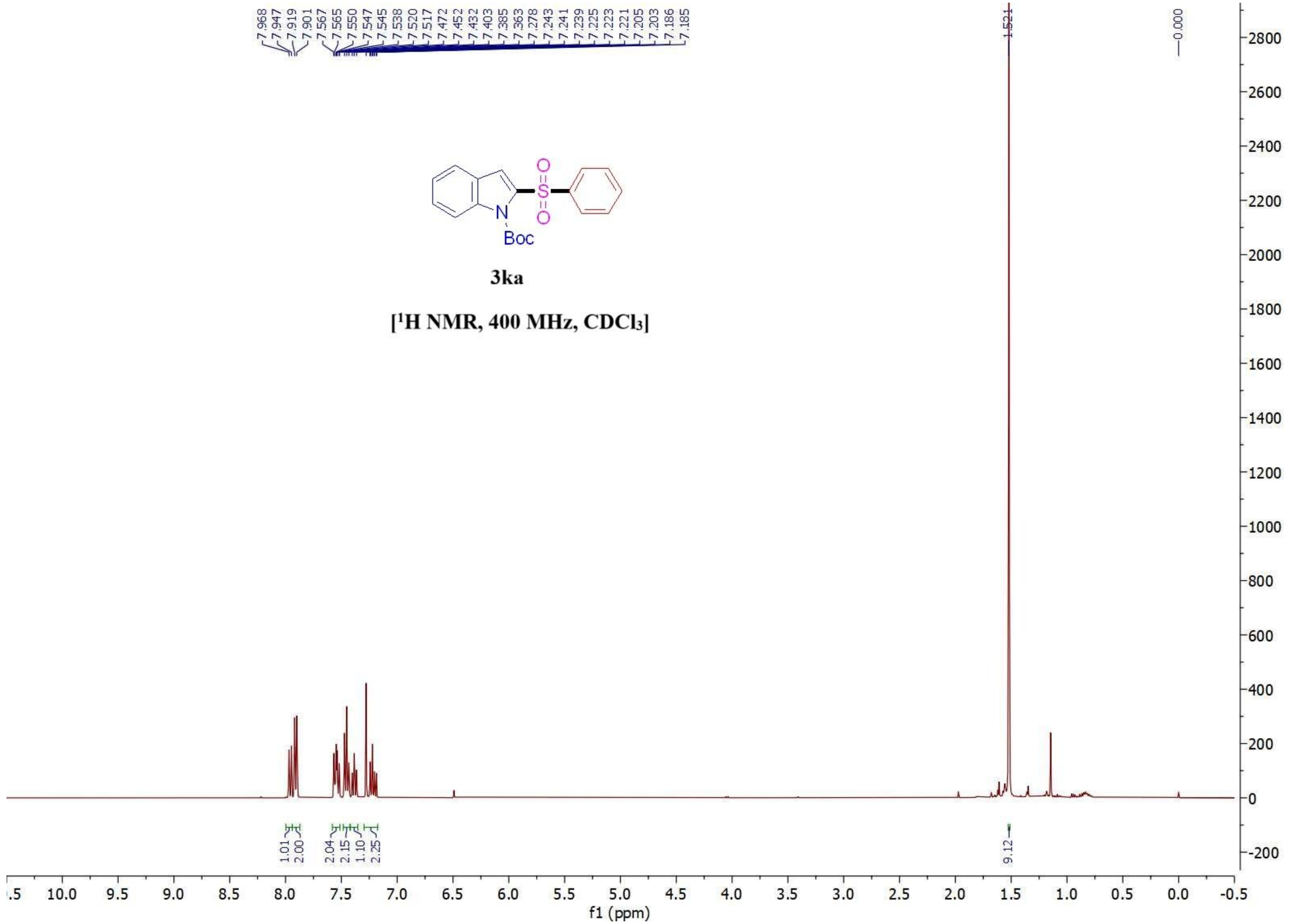

3ka

[$^{1}$H NMR, 400 MHz, CDCl3]

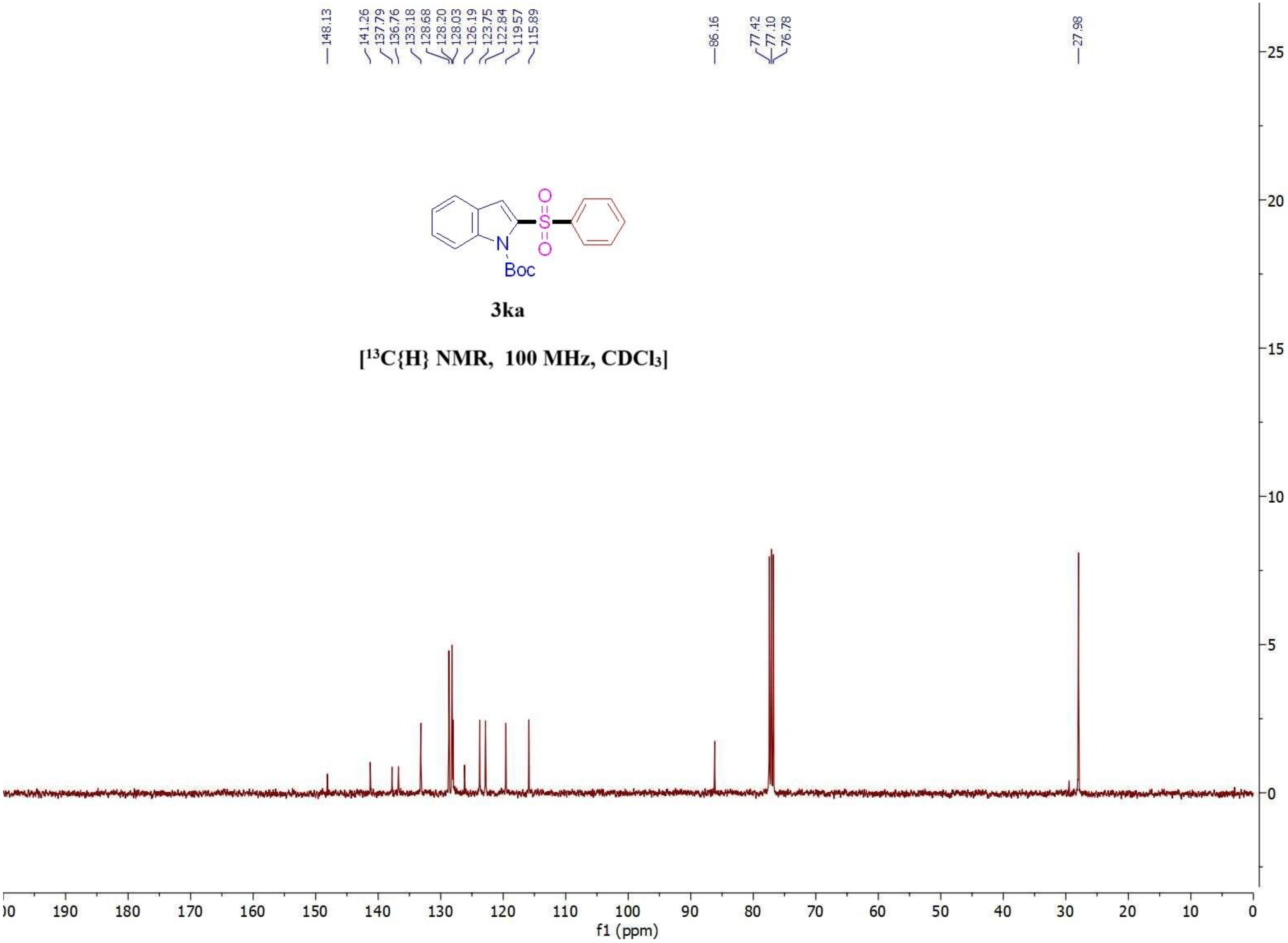
148.13
141.26
137.79
136.76
133.18
128.68
128.20
128.03
126.19
123.75
122.84
119.57
115.89
86.16
77.42
77.10
76.78
27.98
N
Boc
S
O
O
3ka
[13C{H} NMR, 100 MHz, CDCl3]
f1 (ppm)

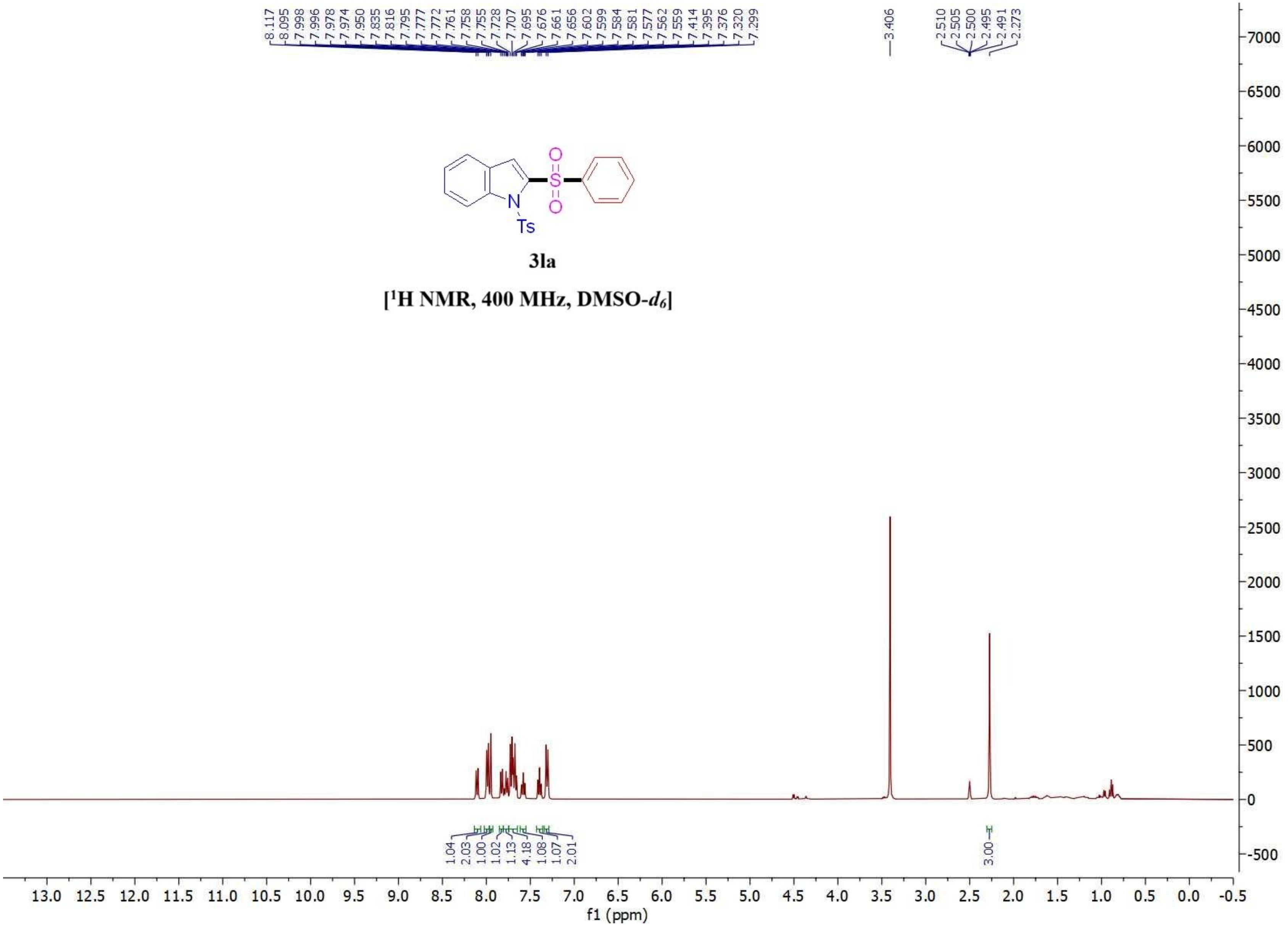


**3la**

**[$^1$H NMR, 400 MHz, DMSO-$d_6$]**

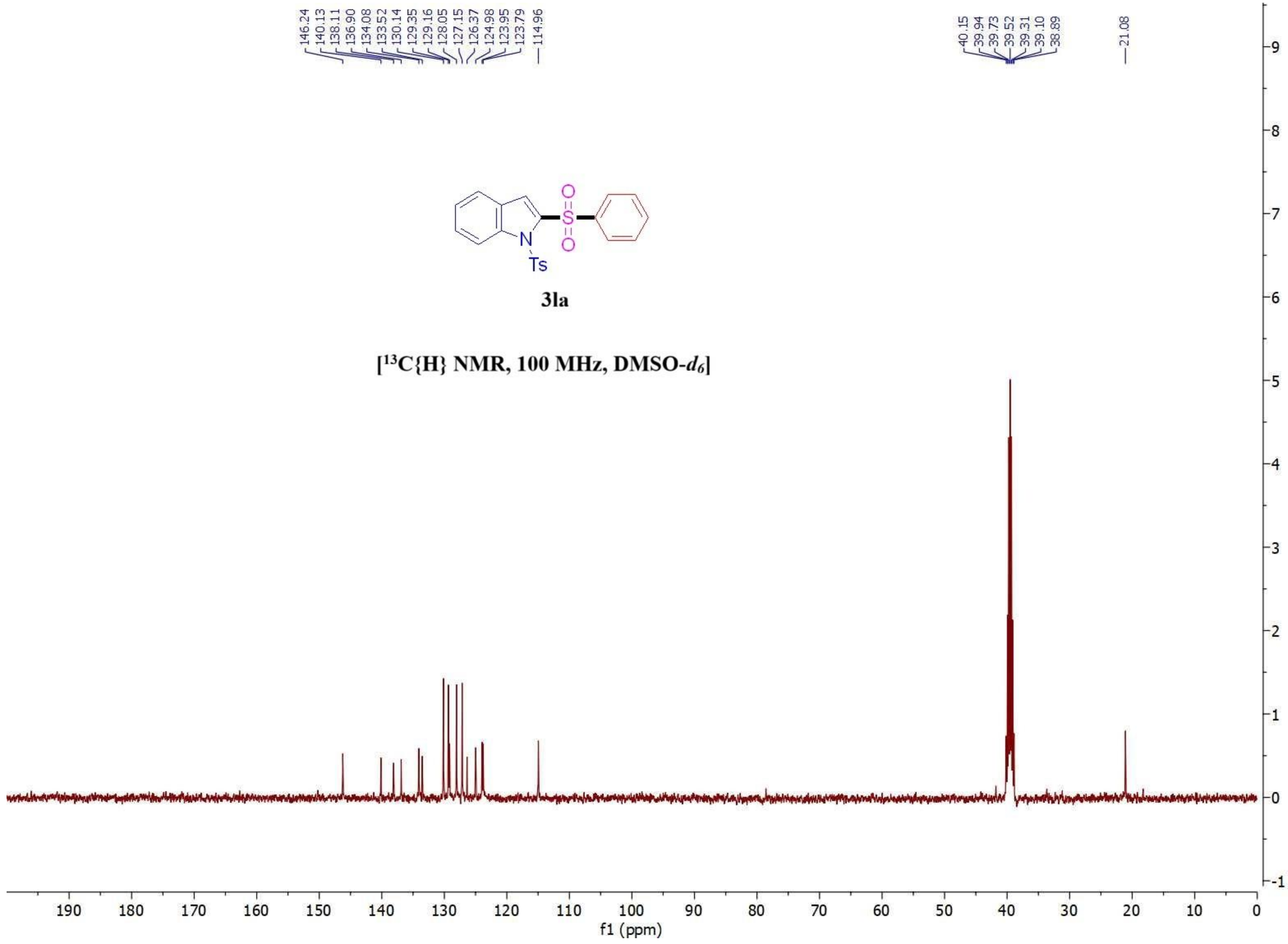


**3la**

[$^{13}C\{H\}$ NMR, 100 MHz, DMSO-$d_6$]

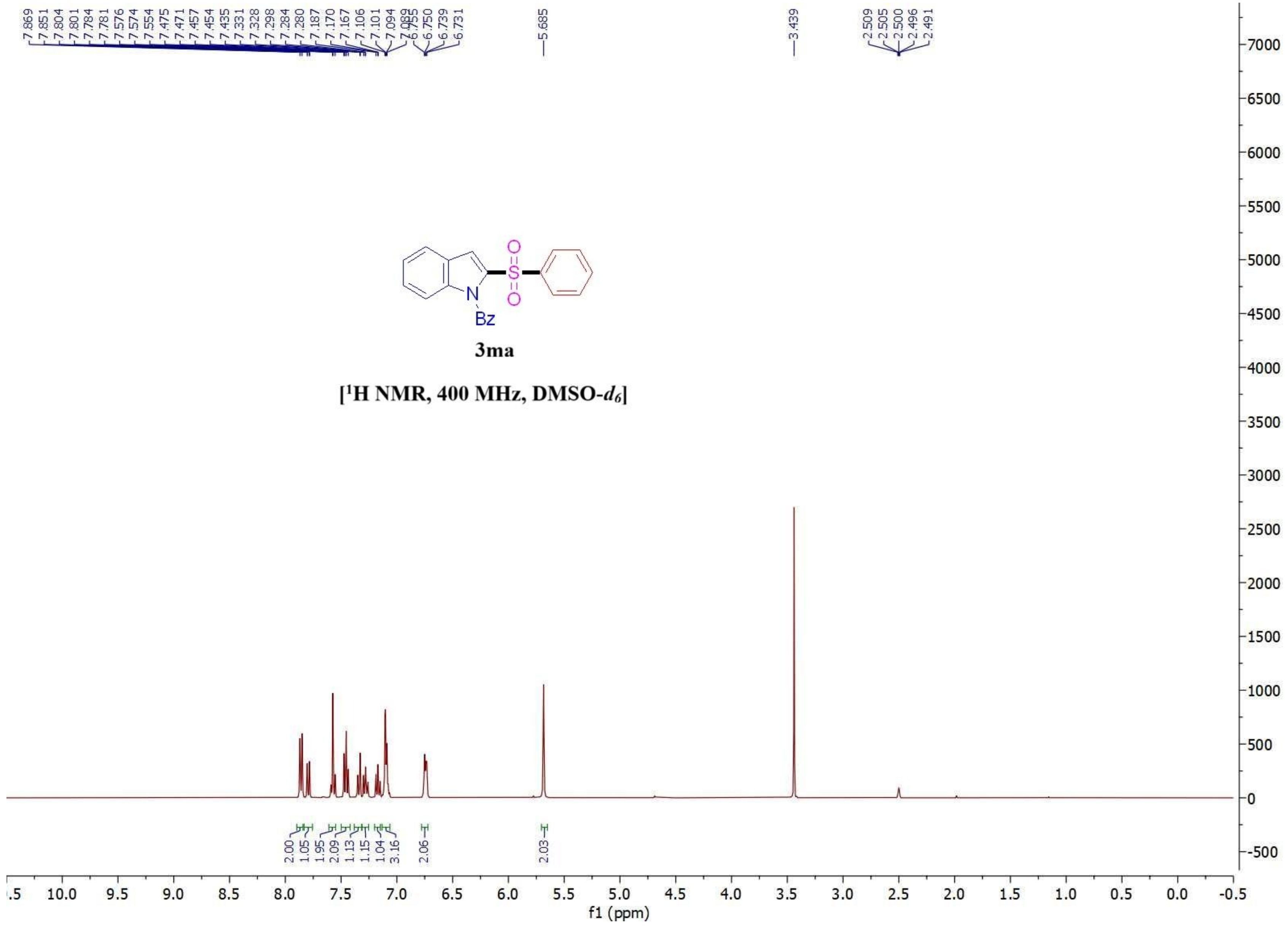

3ma
[$^1$H NMR, 400 MHz, DMSO-$d_6$]
f1 (ppm)

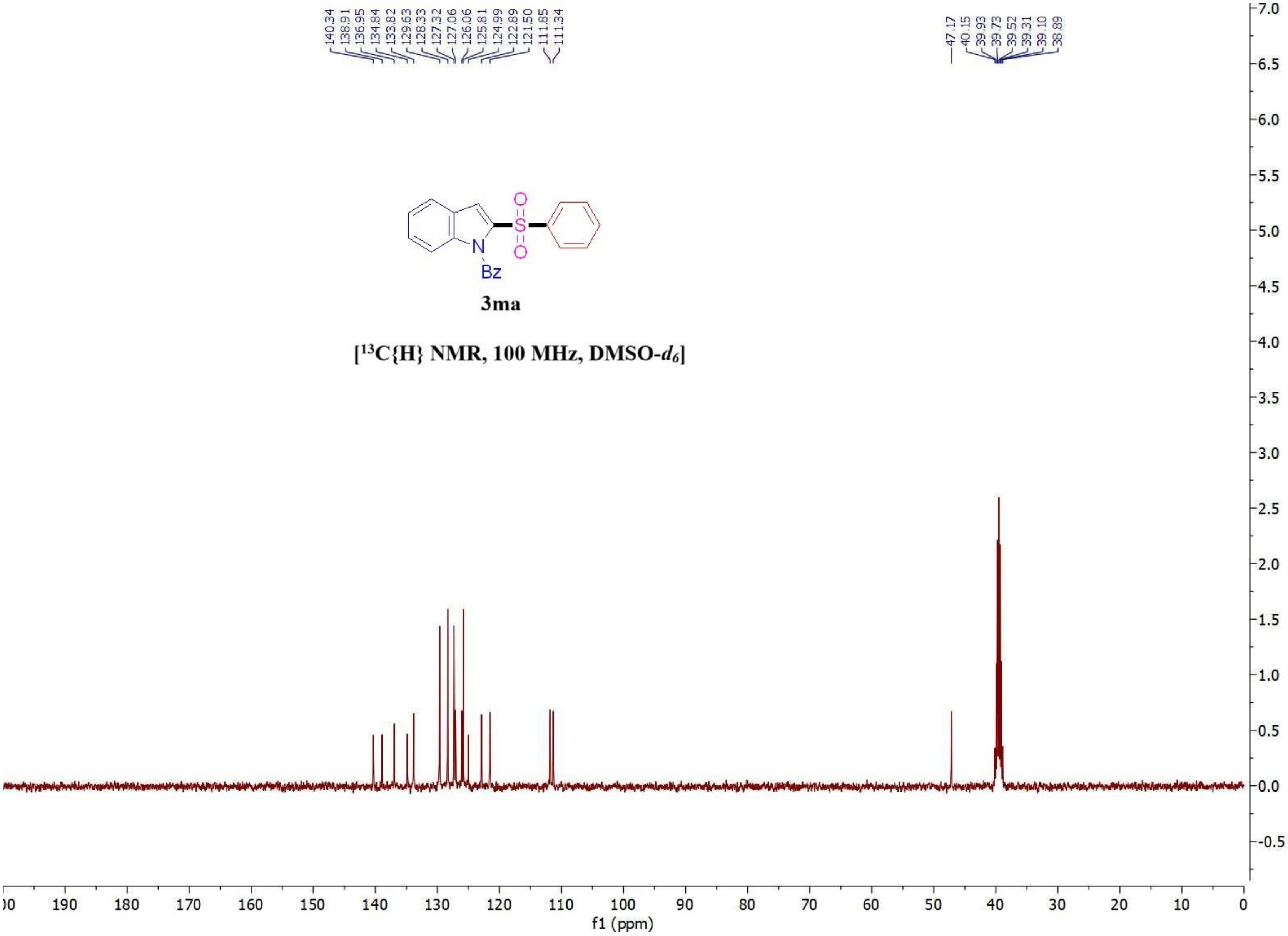


3ma

[$^{13}$C{H} NMR, 100 MHz, DMSO-$d_6$]

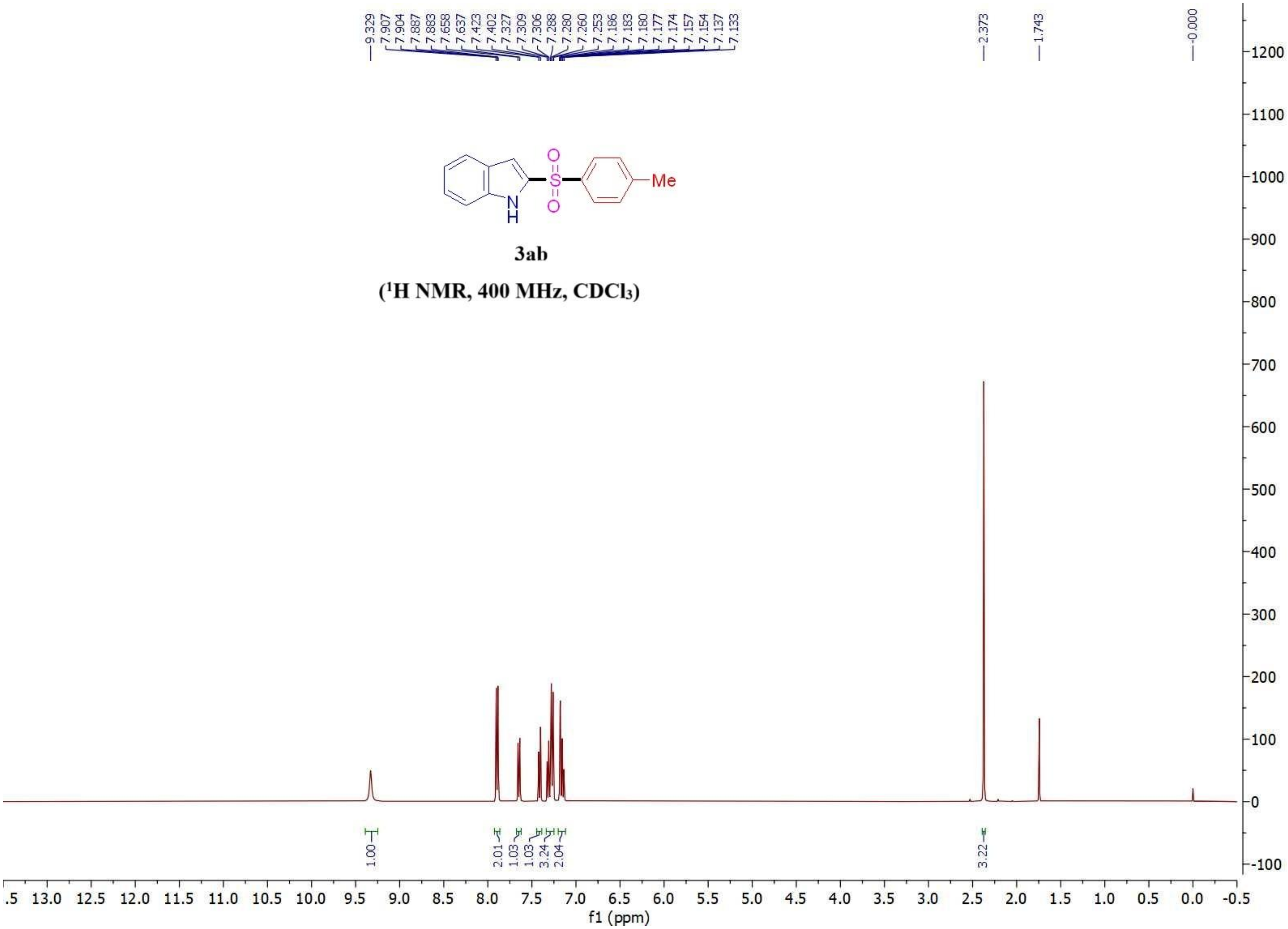
9.329
7.907
7.904
7.887
7.883
7.658
7.637
7.423
7.402
7.327
7.309
7.306
7.288
7.280
7.260
7.253
7.186
7.183
7.180
7.177
7.174
7.157
7.154
7.137
7.133
2.373
1.743
0.000
O
S
O
N
H
Me
3ab
(¹H NMR, 400 MHz, CDCl₃)
1.00
2.01
1.03
1.03
3.24
2.04
3.22
f1 (ppm)

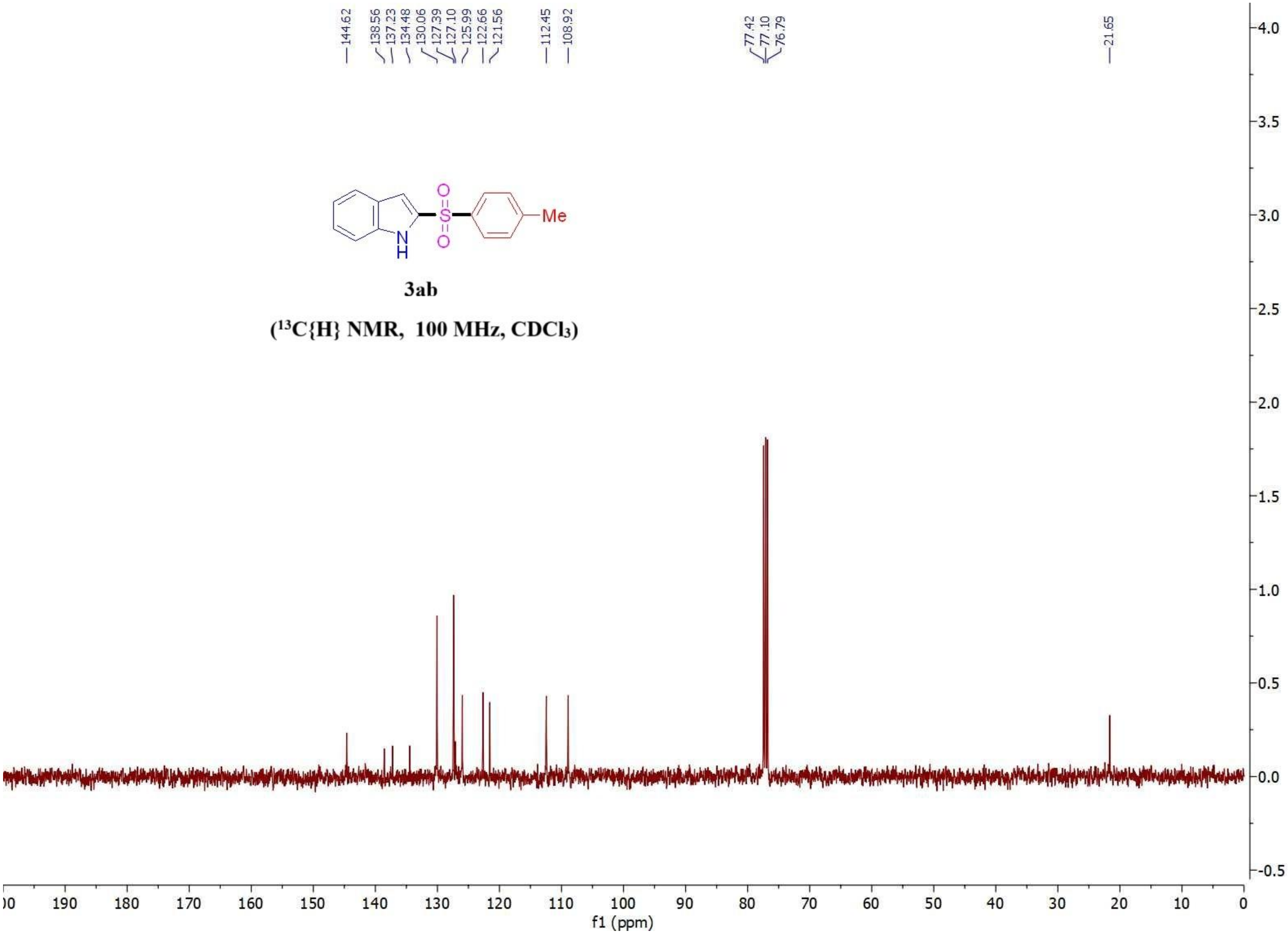


**3ab**

**($^{13}$C{H} NMR, 100 MHz, CDCl$_3$)**

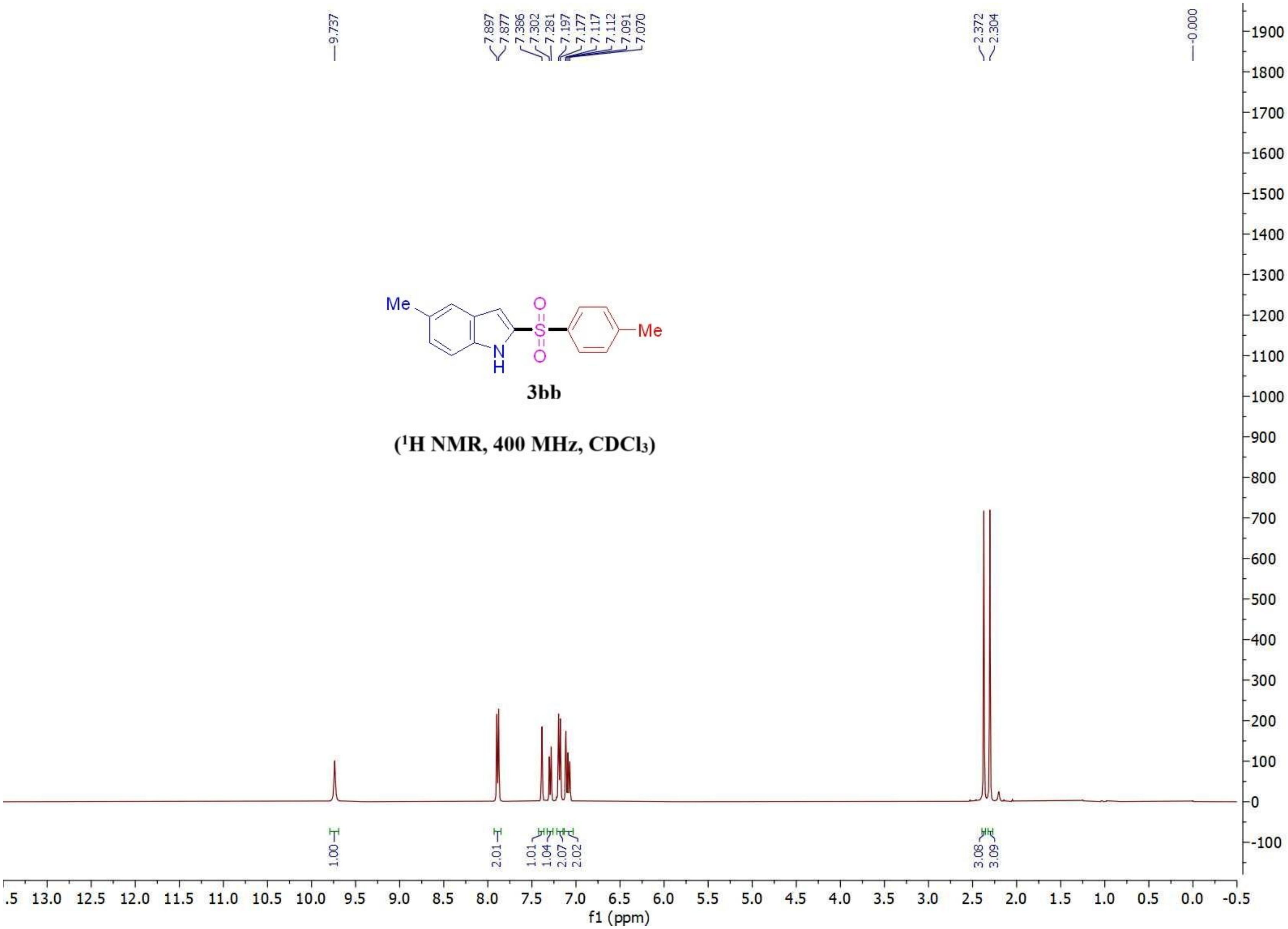

Me
Me
N
H
O
S
O
3bb
(¹H NMR, 400 MHz, CDCl3)
9.737
7.897
7.877
7.386
7.302
7.281
7.197
7.177
7.117
7.112
7.091
7.070
2.372
2.304
0.000
1.00
2.01
1.01
1.04
2.07
2.02
3.08
3.09
f1 (ppm)

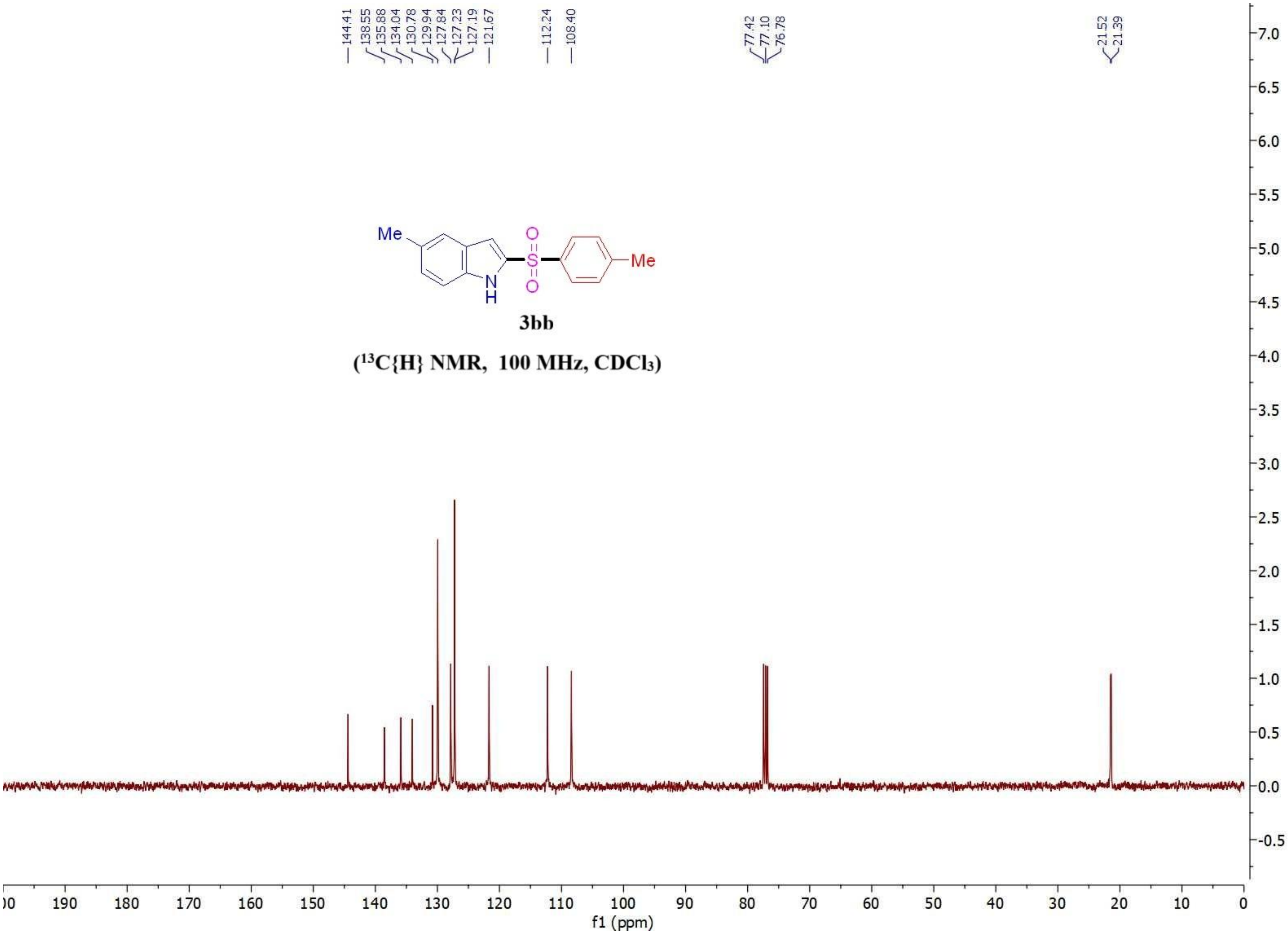


3bb

($^{13}C\{H\}$ NMR, 100 MHz, $CDCl_3$)

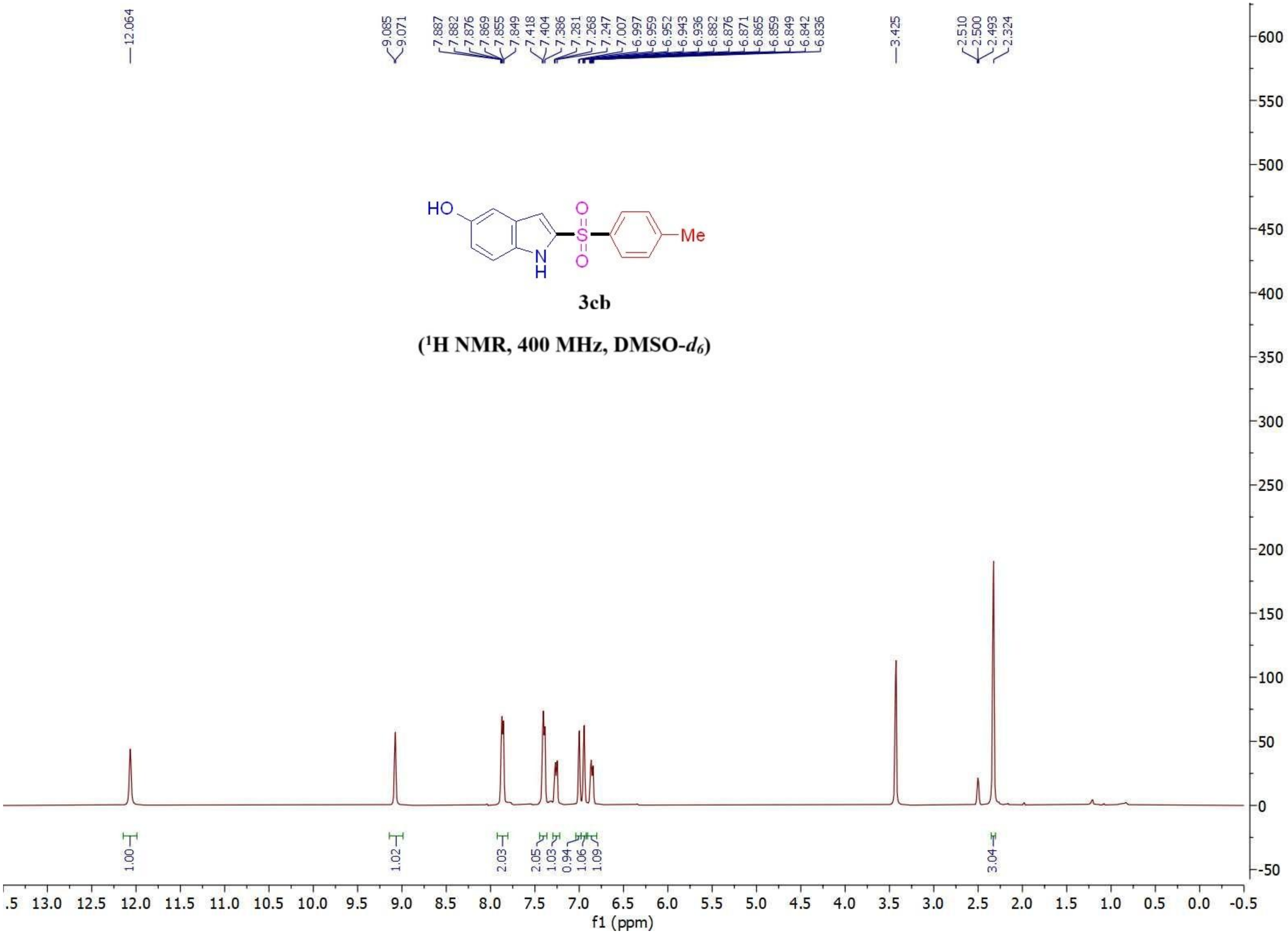
12.064
9.085
9.071
7.887
7.882
7.876
7.869
7.855
7.849
7.418
7.404
7.386
7.281
7.268
7.247
7.007
6.997
6.959
6.952
6.943
6.936
6.882
6.876
6.871
6.865
6.859
6.849
6.842
6.836
3.425
2.510
2.500
2.493
2.324
HO
O
S
O
N
H
Me
3cb
($^1$H NMR, 400 MHz, DMSO-$d_6$)
1.00
1.02
2.03
2.05
1.03
0.94
1.06
1.09
3.04
f1 (ppm)

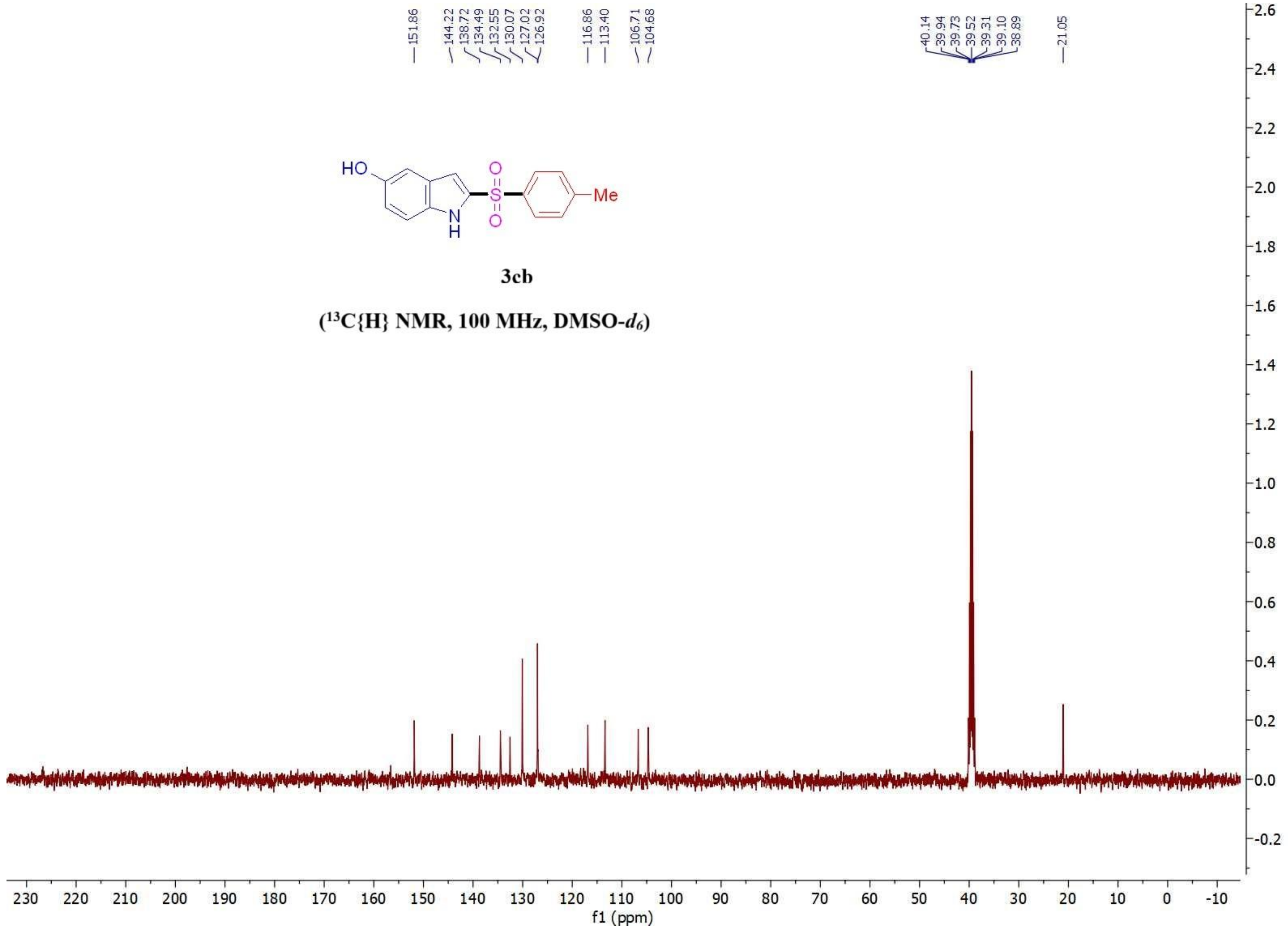


**3cb**

**($^{13}$C{H} NMR, 100 MHz, DMSO-*d*$_6$)**

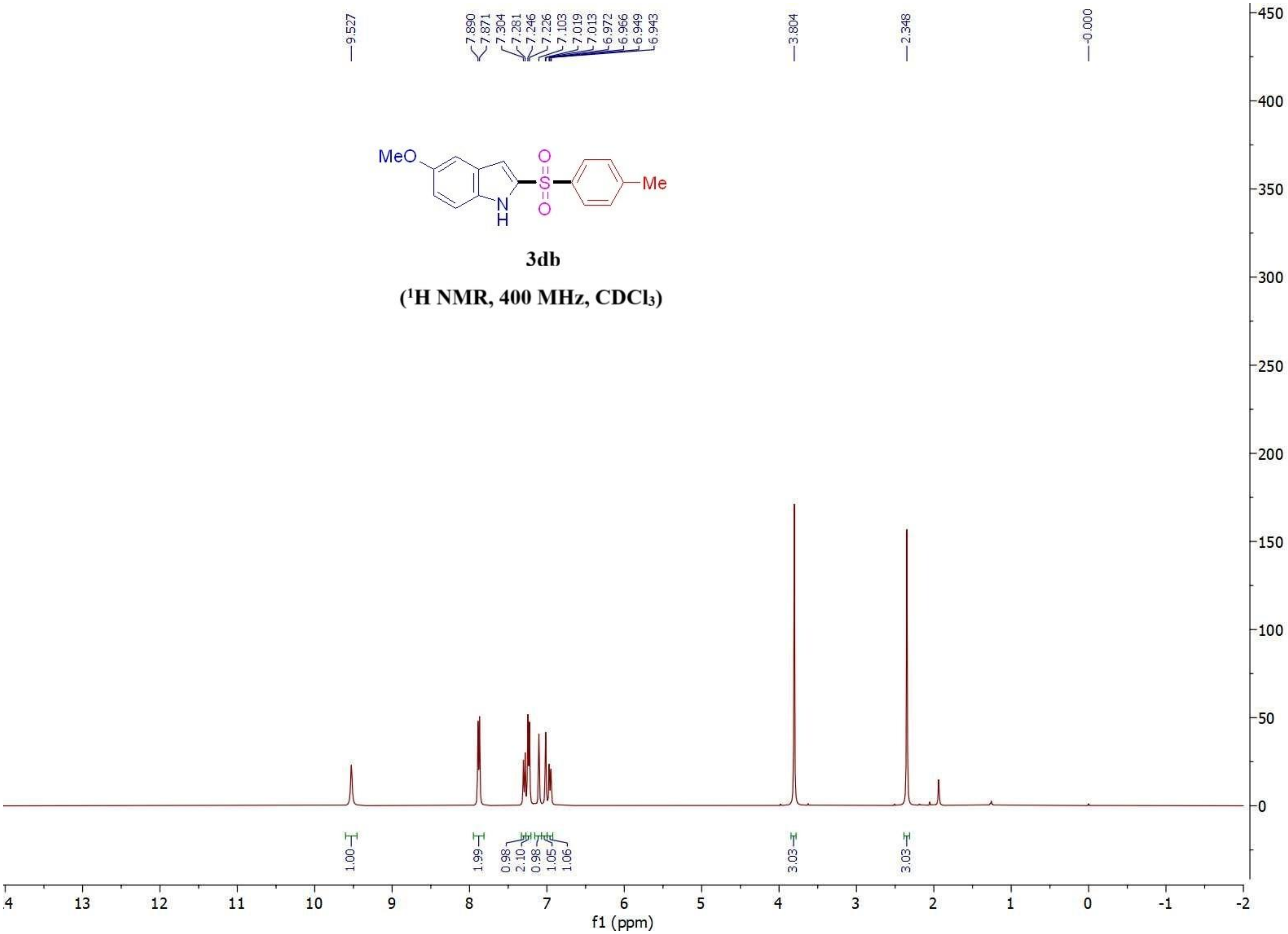
MeO
Me
3db
(1H NMR, 400 MHz, CDCl3)
9.527
7.890
7.871
7.304
7.281
7.246
7.226
7.103
7.019
7.013
6.972
6.966
6.949
6.943
3.804
2.348
0.000
1.00
1.99
0.98
2.10
0.98
1.05
1.06
3.03
3.03
f1 (ppm)

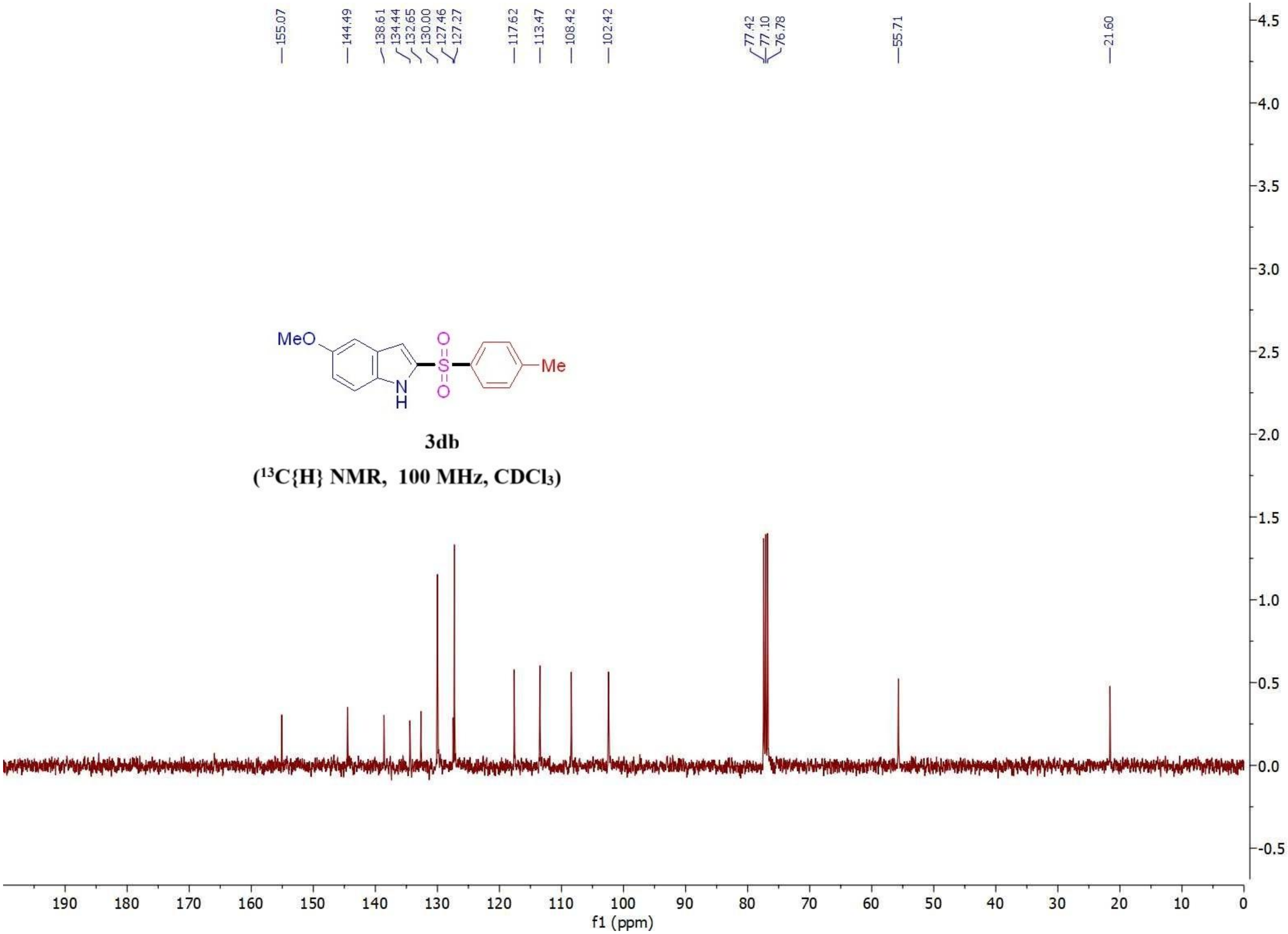


**3db**

**($^{13}$C{H} NMR, 100 MHz, $CDCl_3$)**

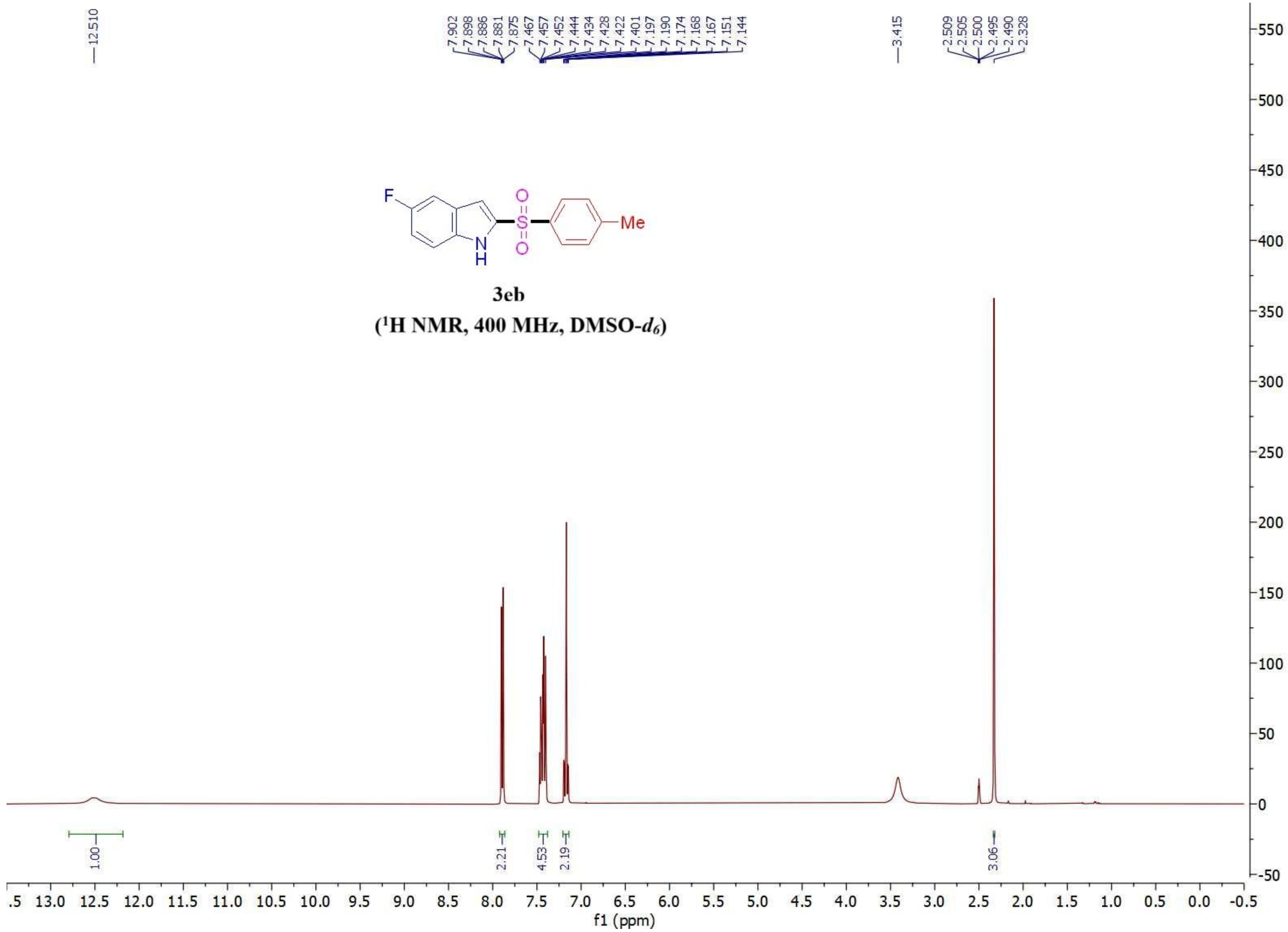

F
NH
Me
3eb
(¹H NMR, 400 MHz, DMSO-$d_6$)

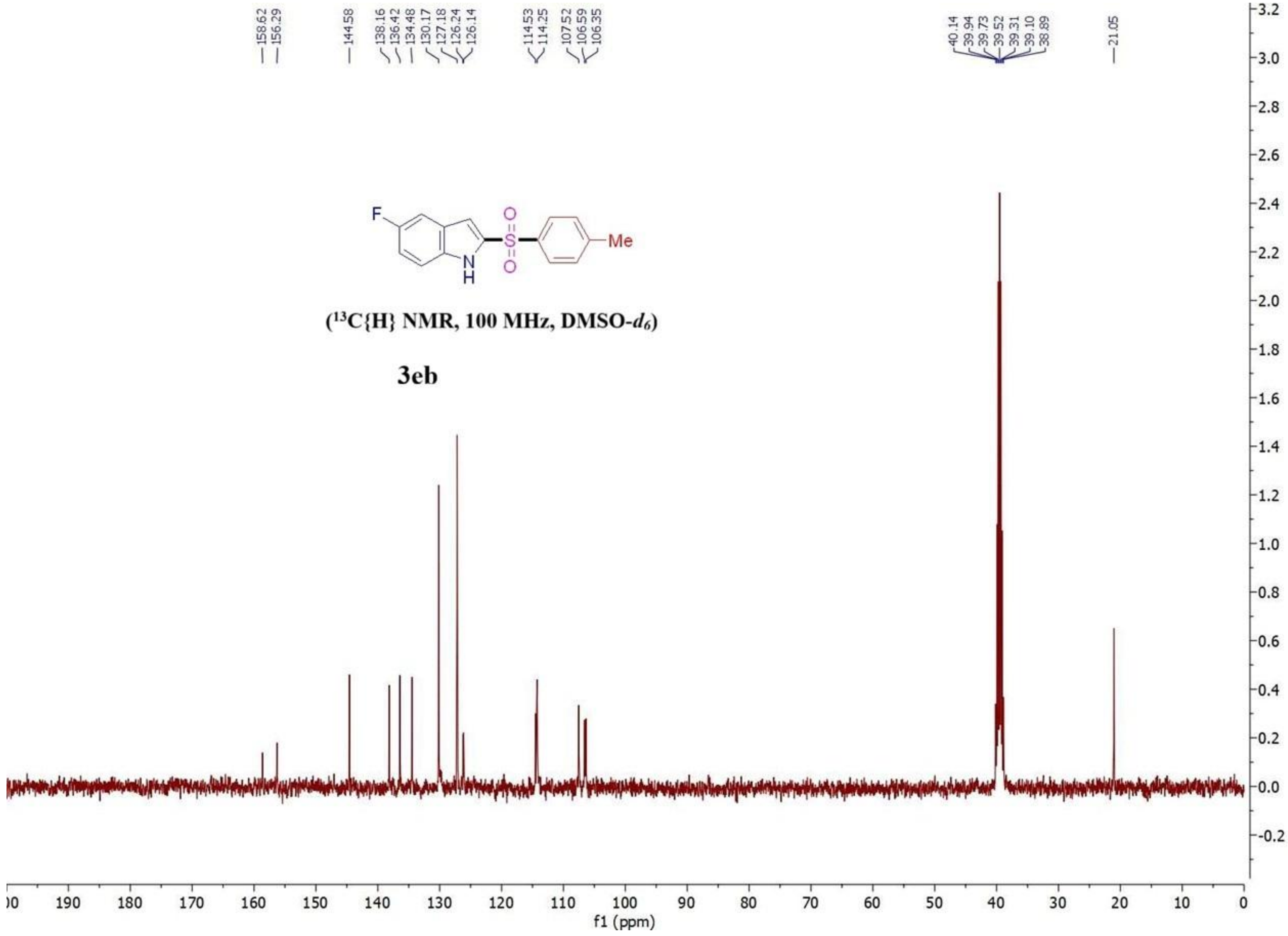
158.62
156.29
144.58
138.16
136.42
134.48
130.17
127.18
126.24
126.14
114.53
114.25
107.52
106.59
106.35
40.14
39.94
39.73
39.52
39.31
39.10
38.89
21.05
F
O
S
O
N
H
Me
($^{13}$C{H} NMR, 100 MHz, DMSO-$d_6$)
3eb
f1 (ppm)

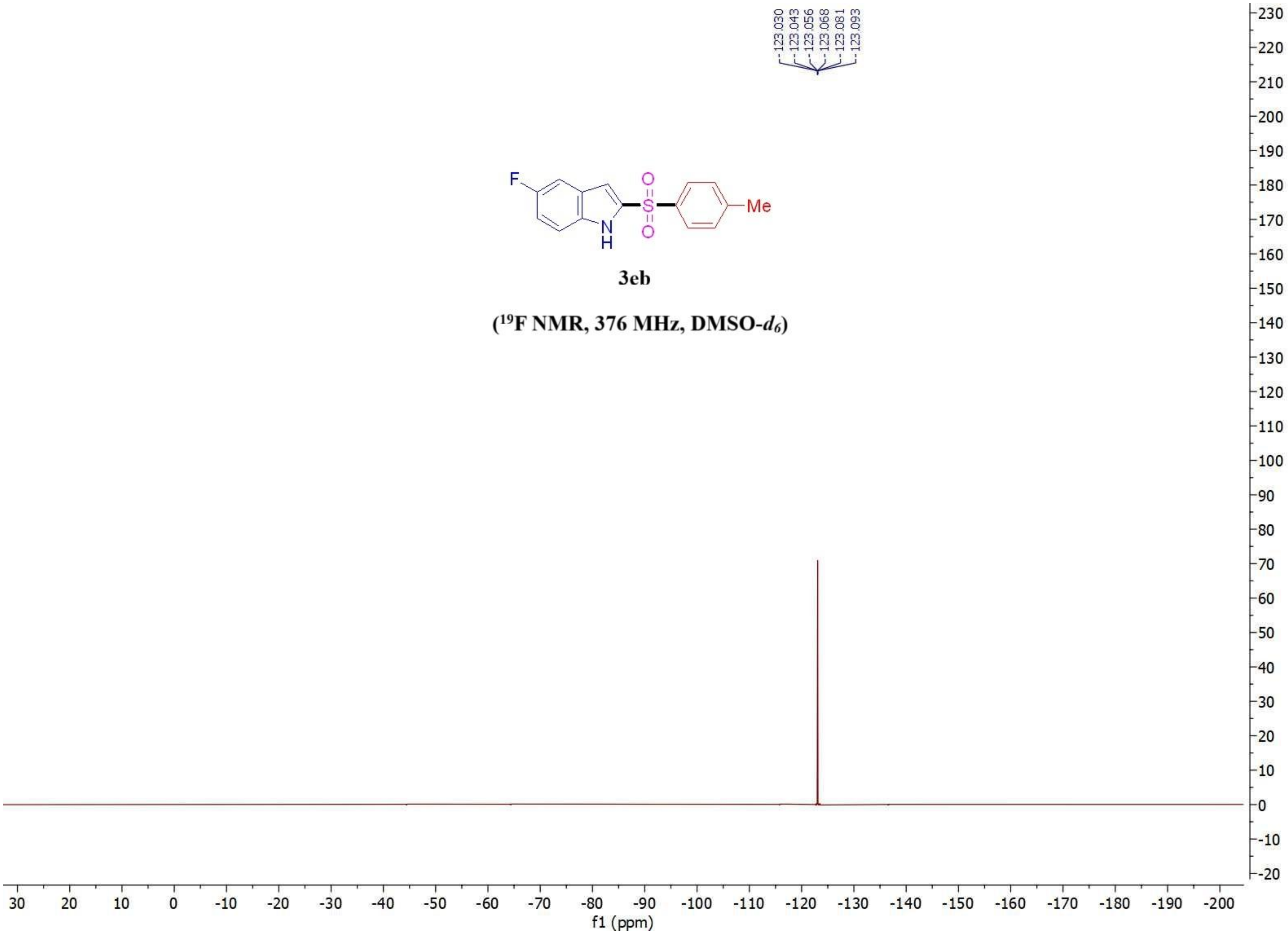


**3eb**

**($^{19}$F NMR, 376 MHz, DMSO-$d_6$)**

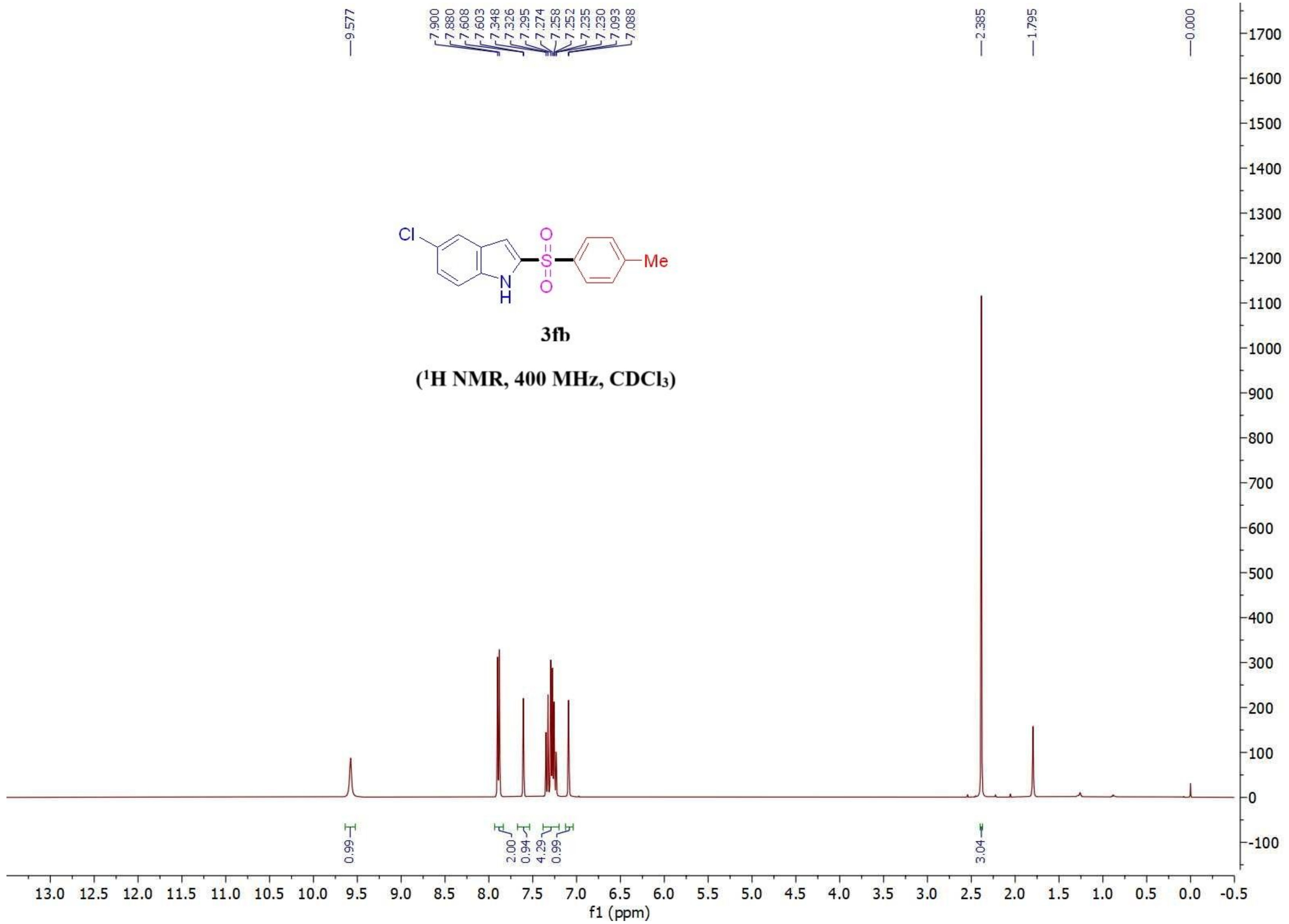

9.577
7.900
7.880
7.608
7.603
7.348
7.326
7.295
7.274
7.258
7.252
7.235
7.230
7.093
7.088
2.385
1.795
0.000
Cl
O
S
O
N
H
Me
3fb
($^1$H NMR, 400 MHz, CDCl$_3$)
0.99
2.00
0.94
4.29
0.99
3.04
f1 (ppm)

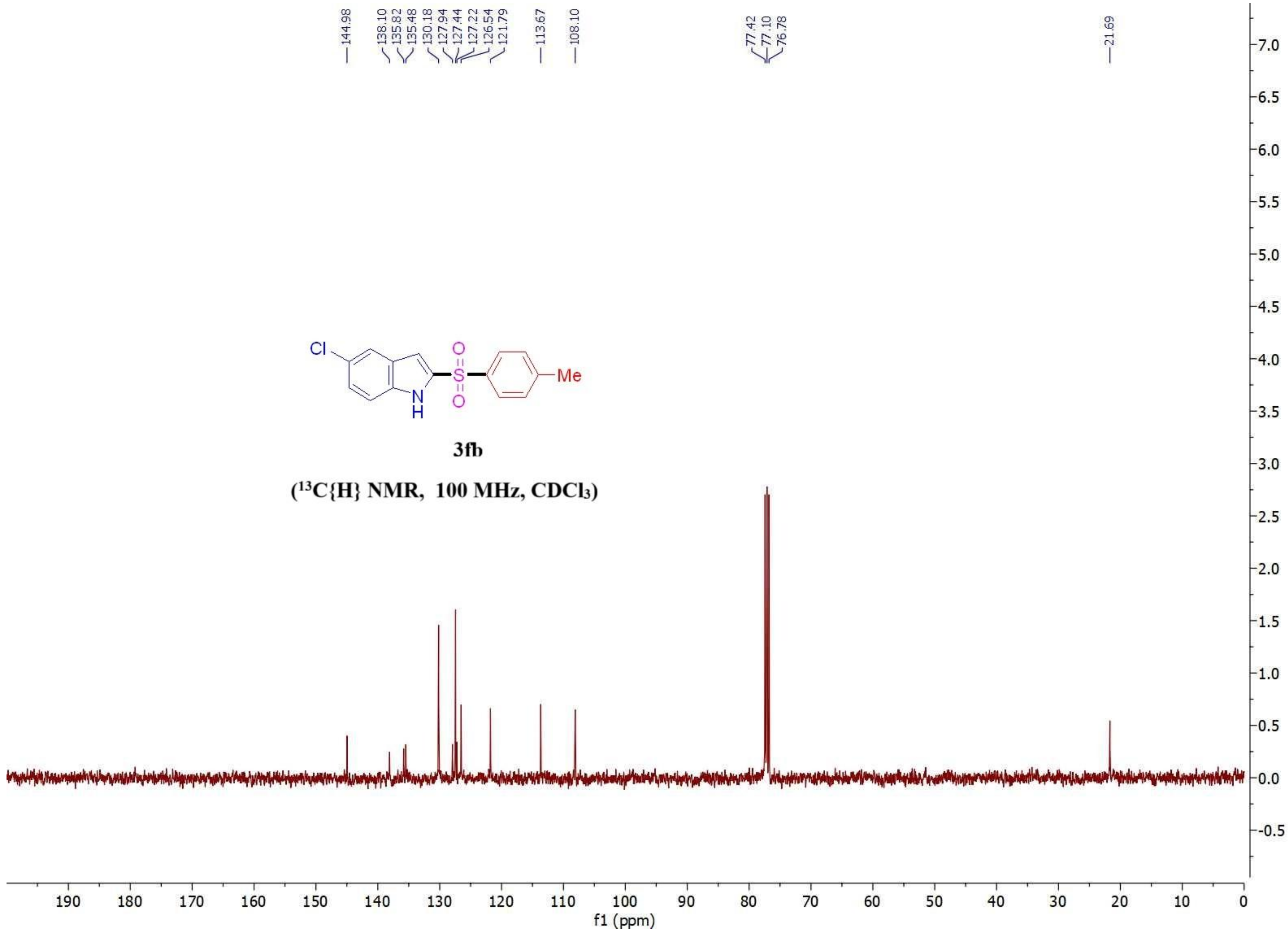


**3fb**

**($^{13}$C{H} NMR, 100 MHz, $CDCl_3$)**

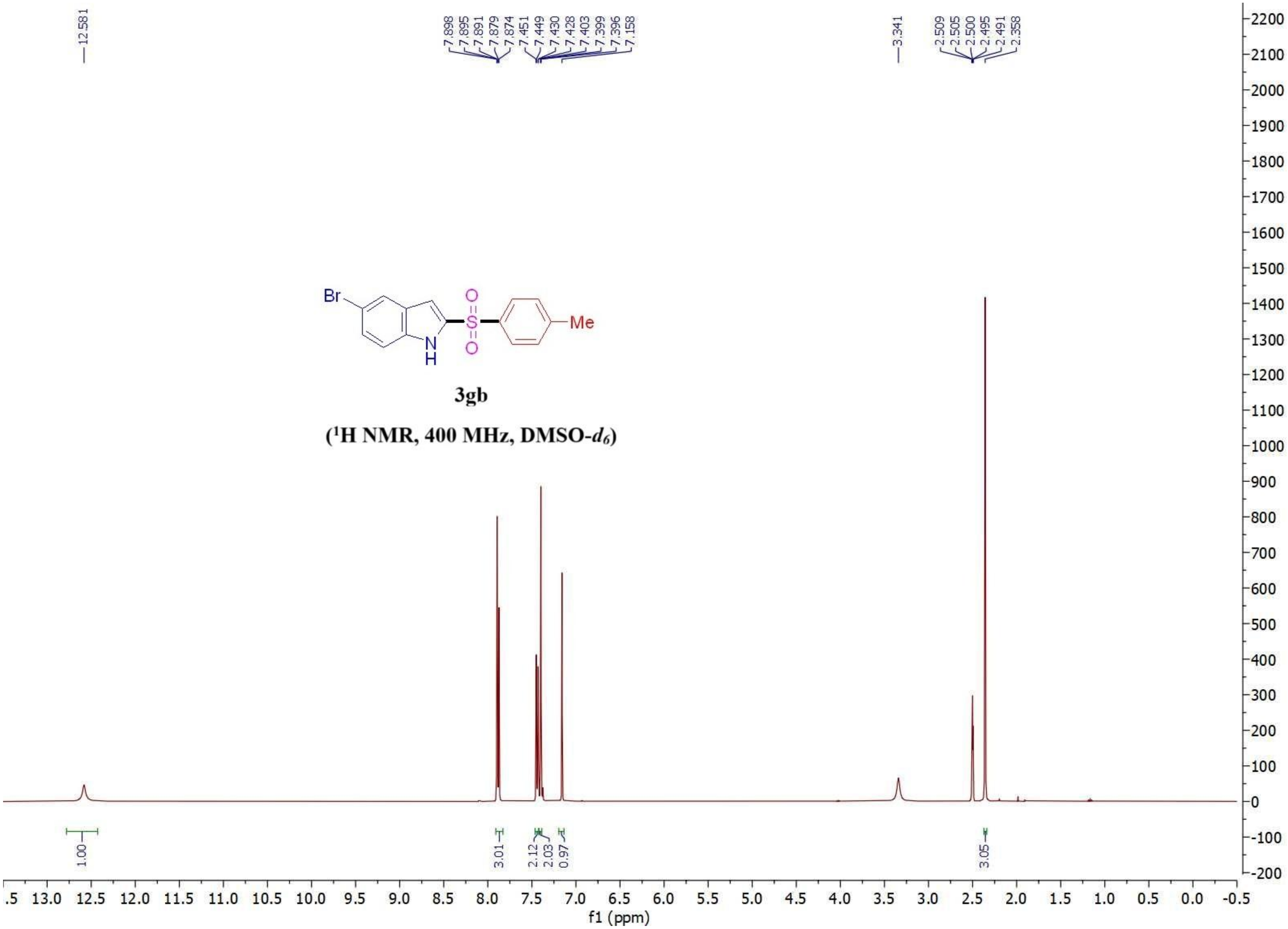


**3gb**

**($^1$H NMR, 400 MHz, DMSO-$d_6$)**

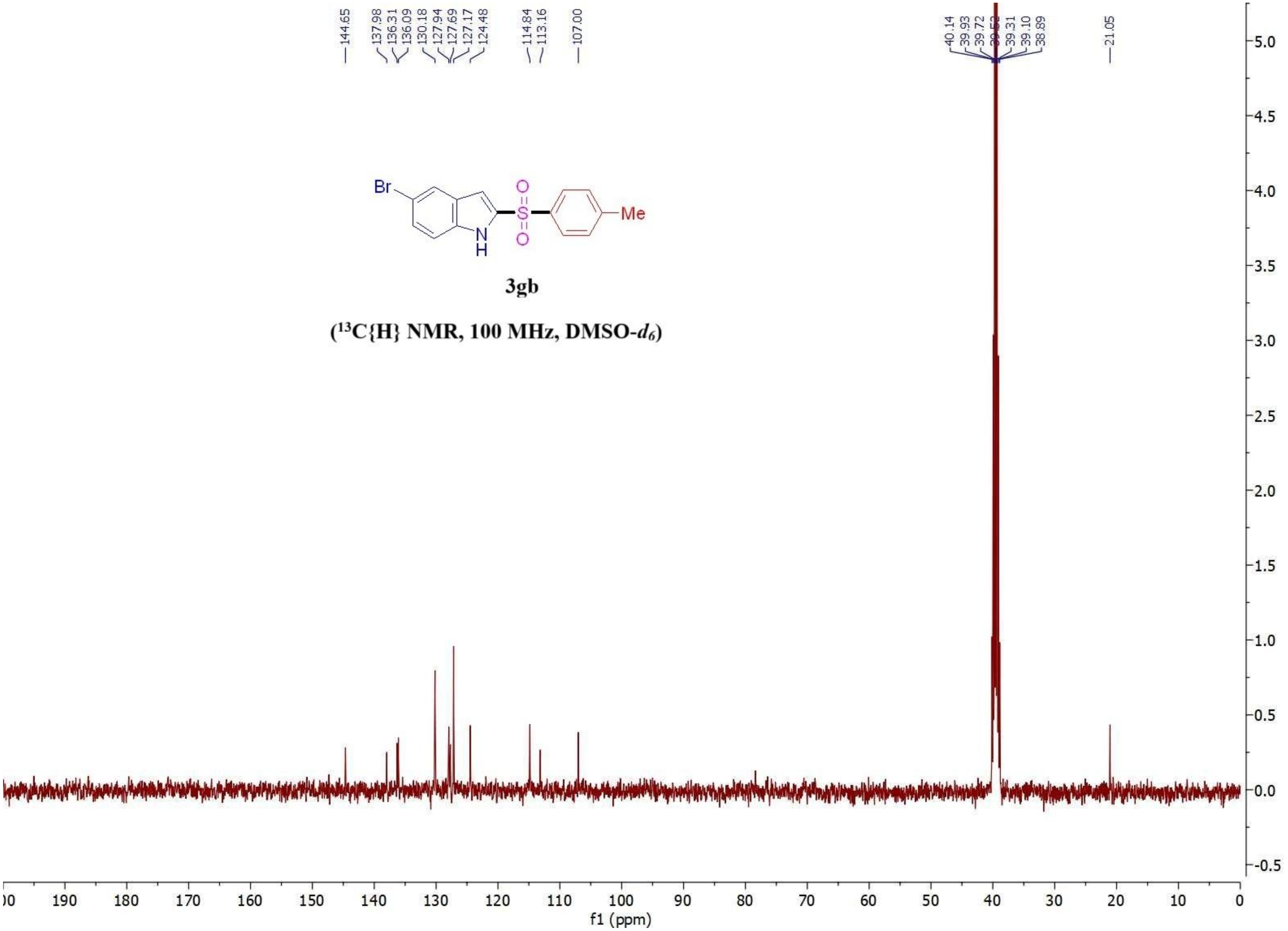
144.65
137.98
136.31
136.09
130.18
127.94
127.69
127.17
124.48
114.84
113.16
107.00
40.14
39.93
39.72
39.52
39.31
39.10
38.89
21.05
Br
N
H
S
O
O
Me
3gb
($^{13}C\{H\}$ NMR, 100 MHz, DMSO-$d_6$)
f1 (ppm)

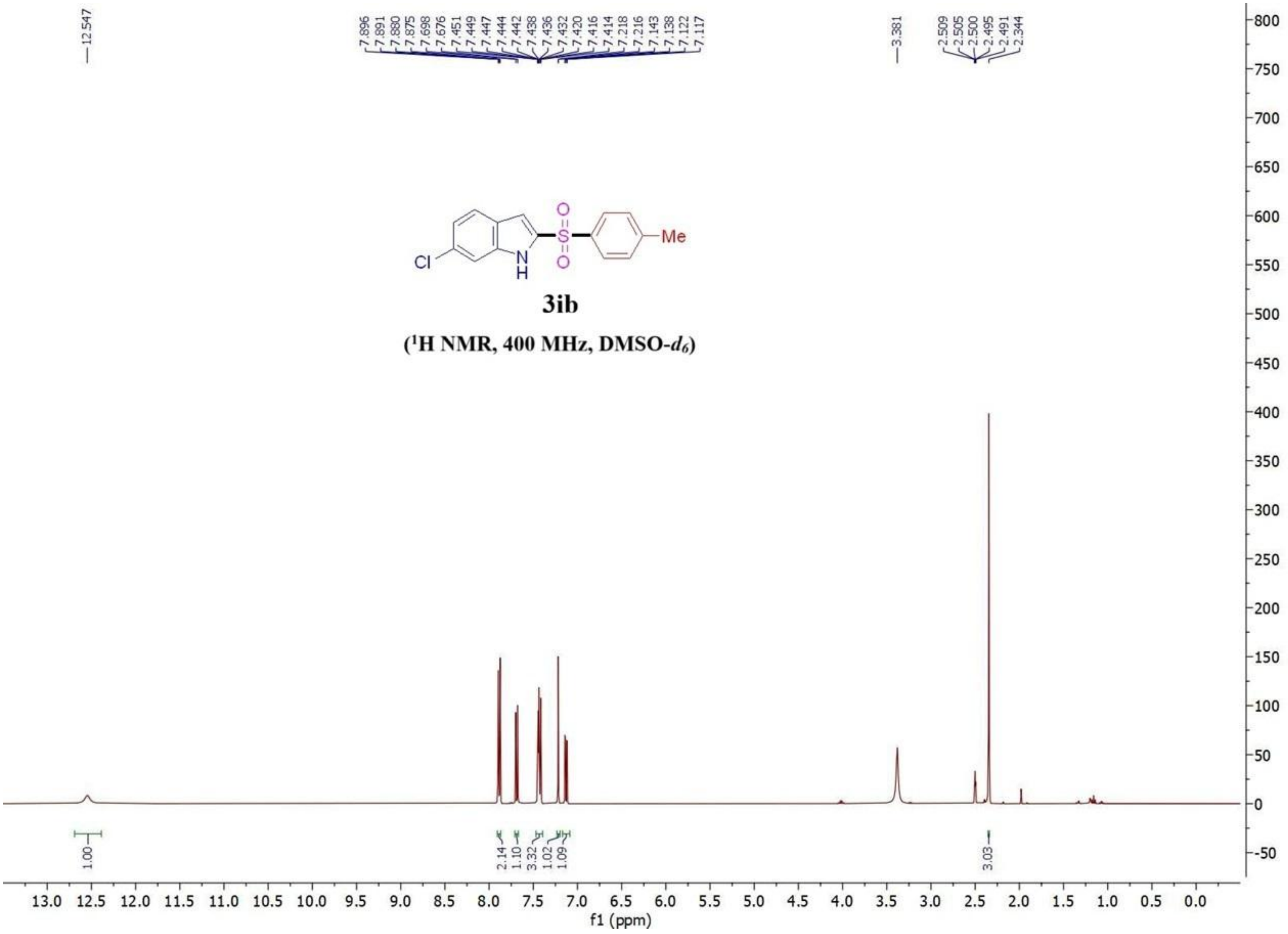

3ib
(¹H NMR, 400 MHz, DMSO-$d_6$)
f1 (ppm)

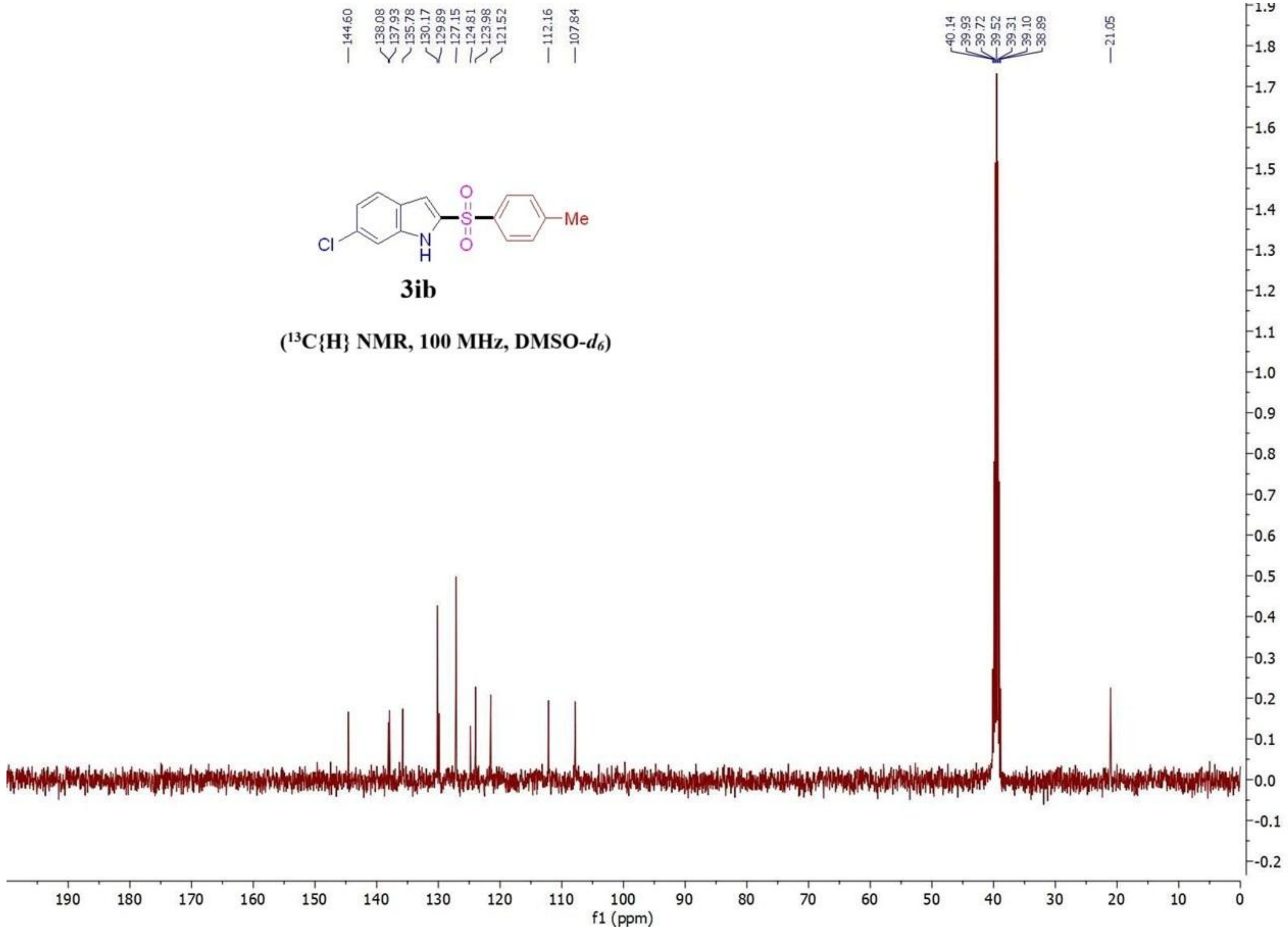

144.60
138.08
137.93
135.78
130.17
129.89
127.15
124.81
123.98
121.52
112.16
107.84
40.14
39.93
39.72
39.52
39.31
39.10
38.89
21.05
Cl
N
H
O
S
O
Me
3ib
(¹³C{H} NMR, 100 MHz, DMSO-d6)
f1 (ppm)

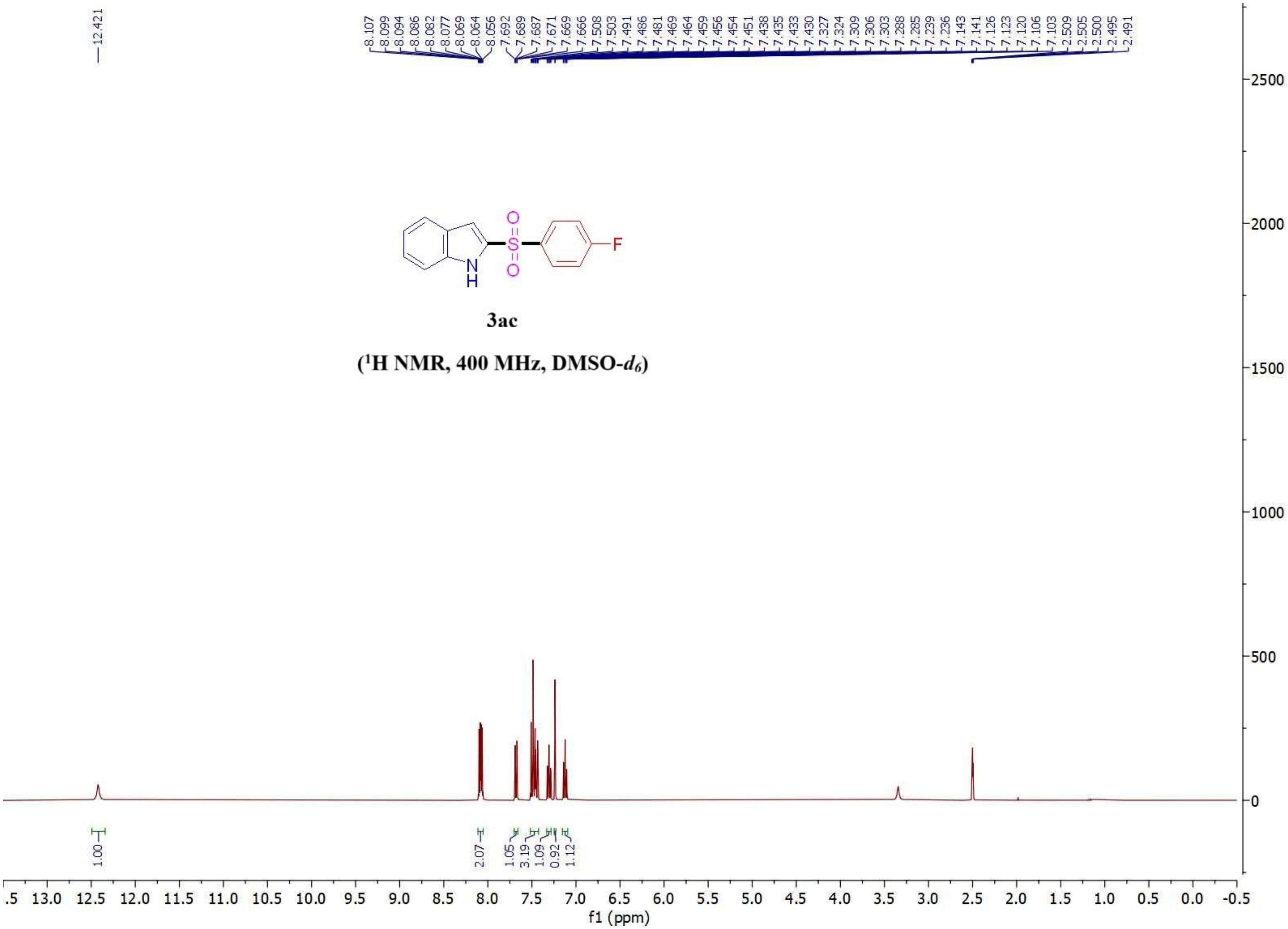

12.421
8.107 8.099 8.094 8.086 8.082 8.077 8.069 8.064 8.056 7.692 7.689 7.687 7.671 7.669 7.666 7.508 7.503 7.491 7.486 7.481 7.469 7.464 7.459 7.456 7.454 7.451 7.438 7.435 7.433 7.430 7.327 7.324 7.309 7.306 7.303 7.288 7.285 7.239 7.236 7.143 7.141 7.126 7.123 7.120 7.106 7.103 2.509 2.505 2.500 2.495 2.491
3ac
(¹H NMR, 400 MHz, DMSO-$d_6$)
1.00 2.07 1.05 3.19 1.09 0.92 1.12
f1 (ppm)

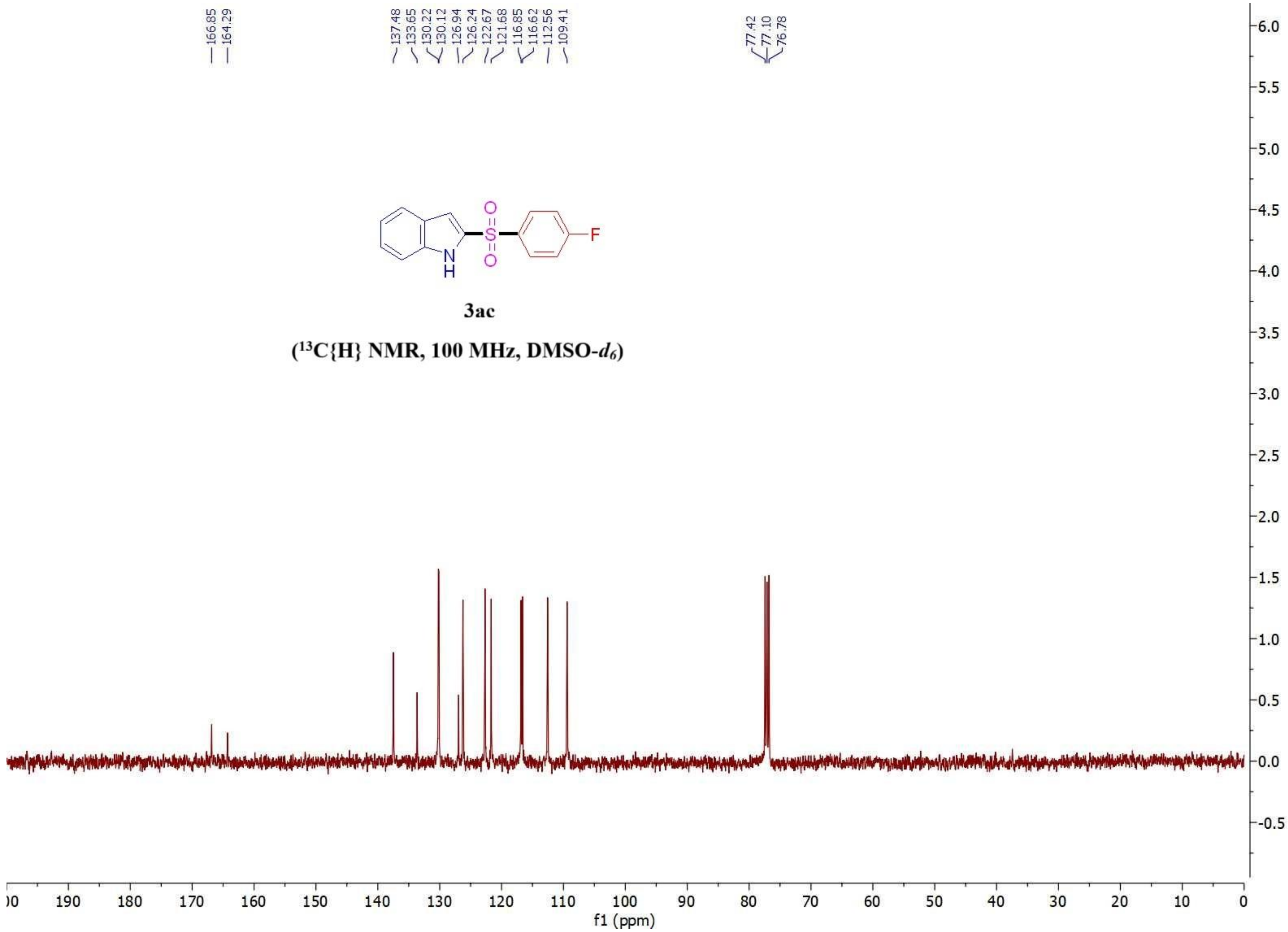


**3ac**

**($^{13}C\{H\}$ NMR, 100 MHz, DMSO-$d_6$)**

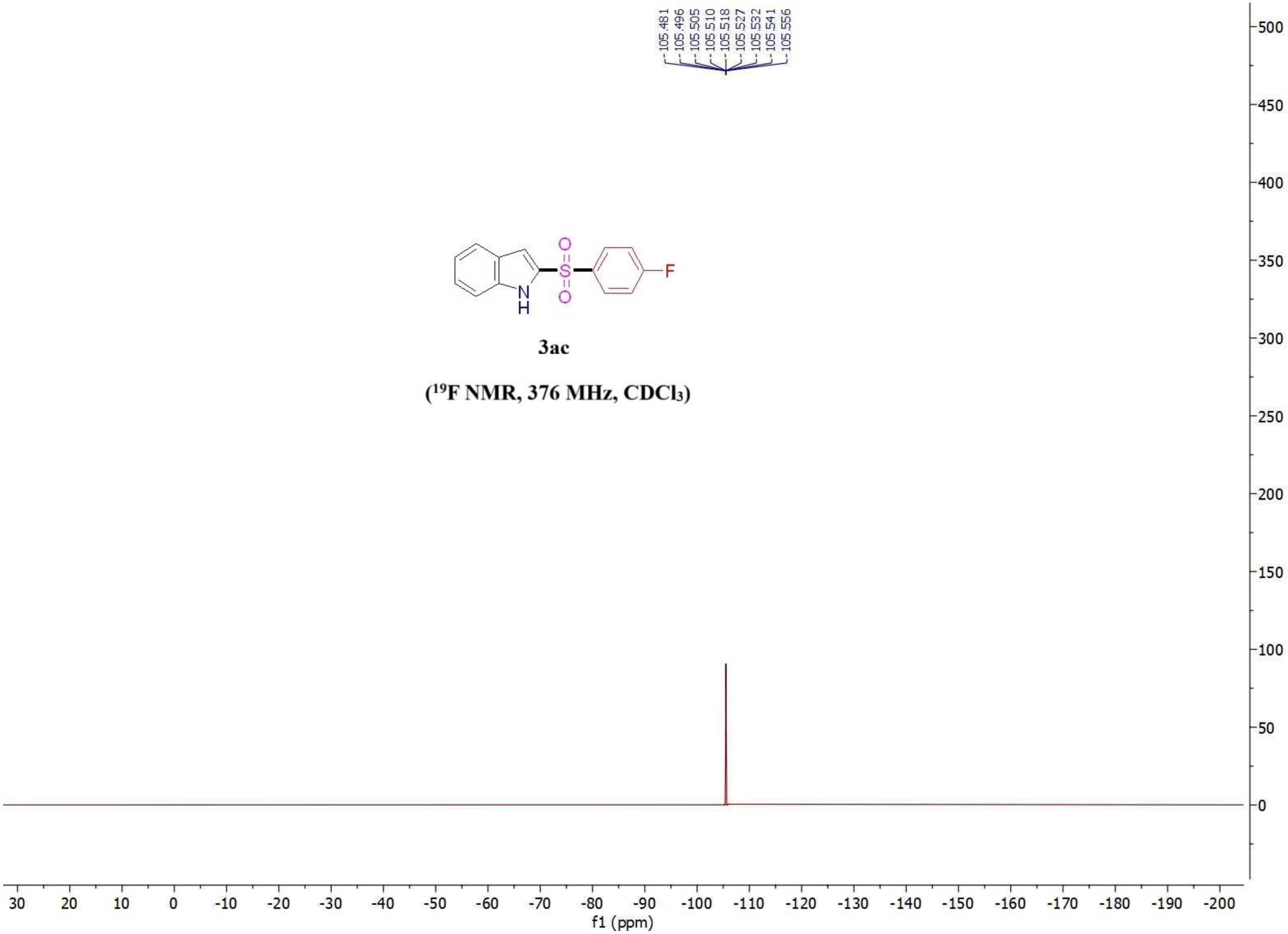


**3ac**

**($^{19}$F NMR, 376 MHz, $CDCl_3$)**

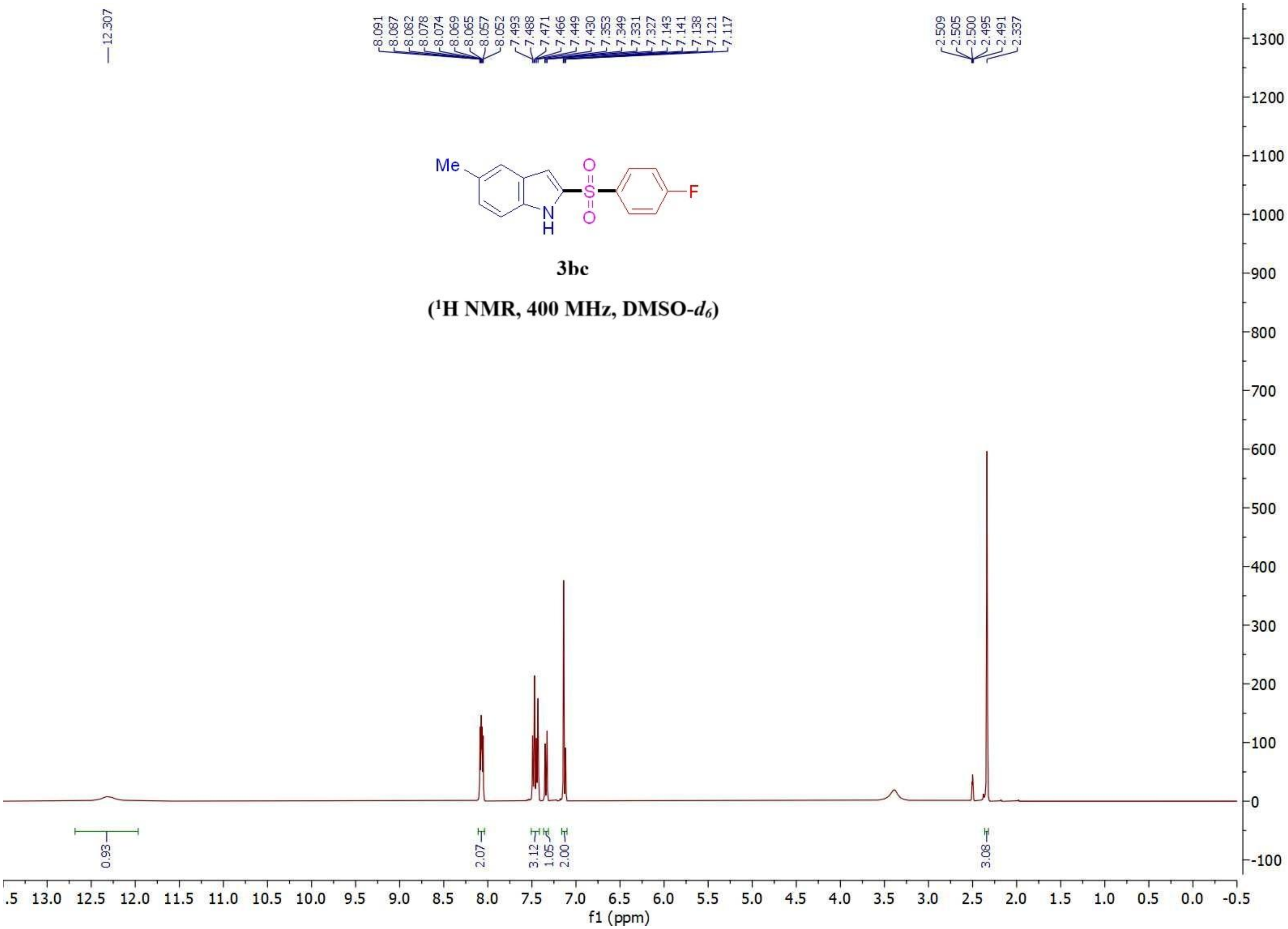
12.307
8.091 8.087 8.082 8.078 8.074 8.069 8.065 8.057 8.052 7.493 7.488 7.471 7.466 7.449 7.430 7.353 7.349 7.331 7.327 7.143 7.141 7.138 7.121 7.117
2.509 2.505 2.500 2.495 2.491 2.337
Me
O
S
O
N
H
F
3bc
(1H NMR, 400 MHz, DMSO-d6)
0.93
2.07
3.12
1.05
2.00
3.08
f1 (ppm)

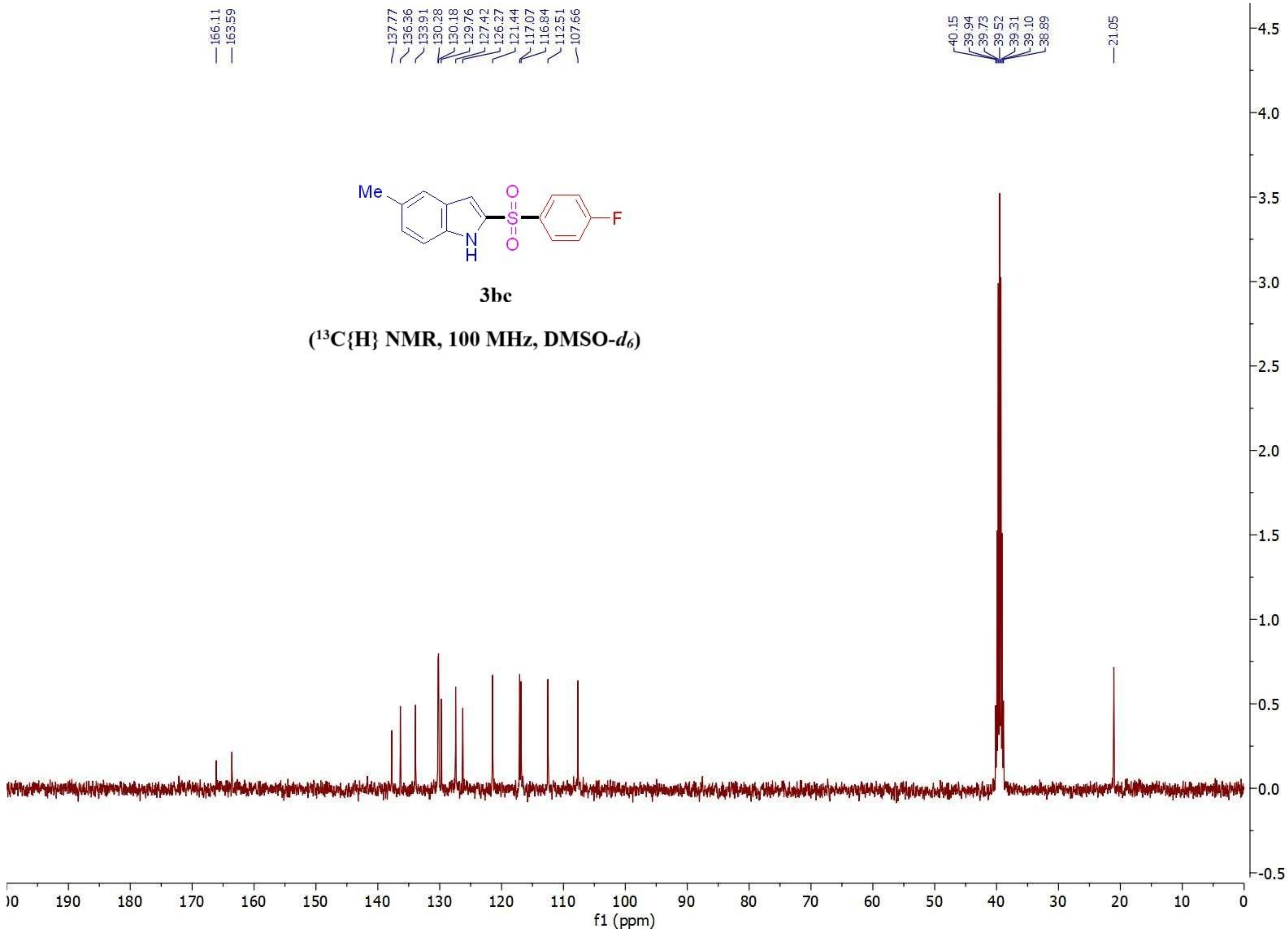


**3bc**

**($^{13}$C{H} NMR, 100 MHz, DMSO-$d_6$)**

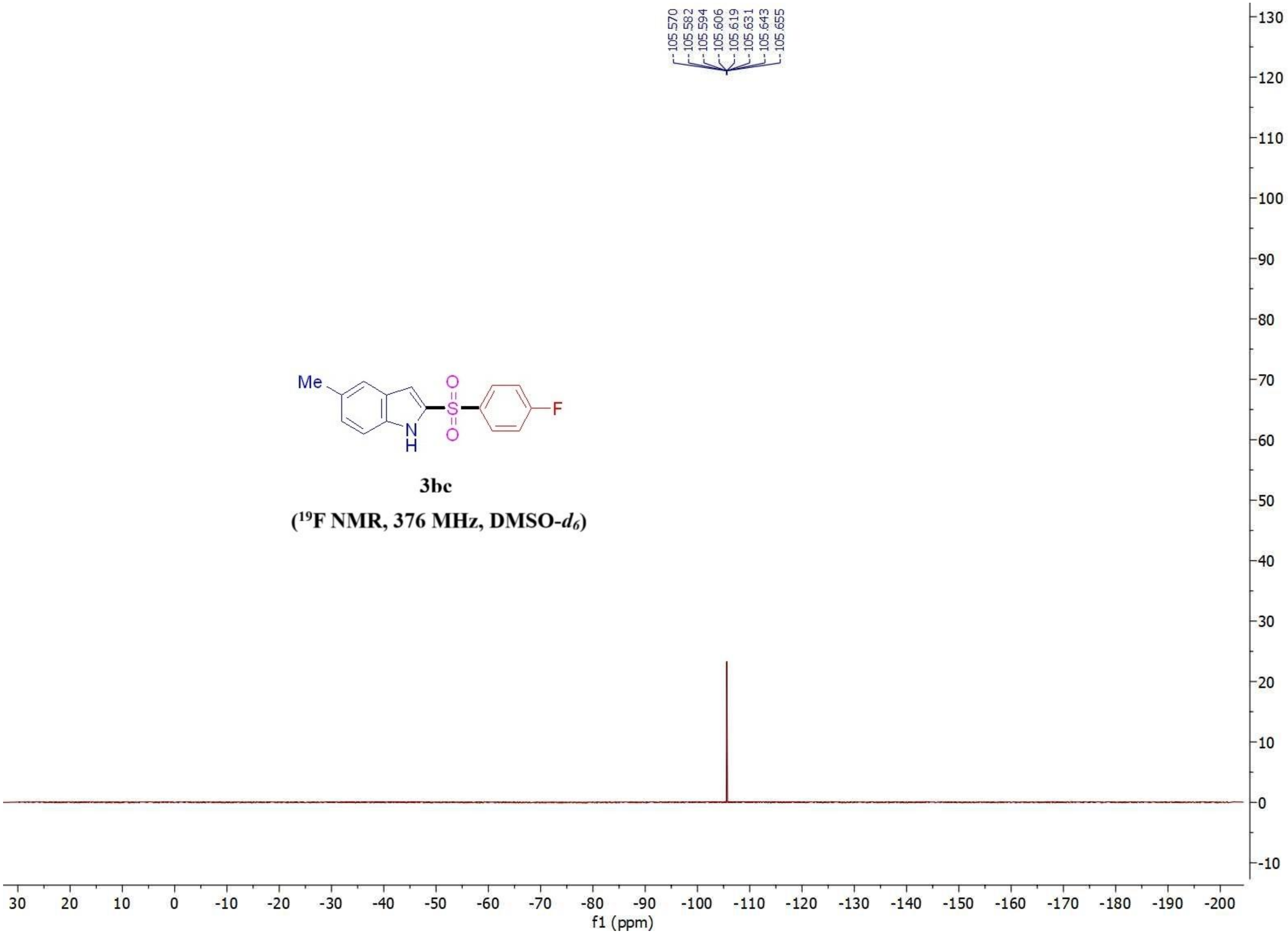


**3bc**

**($^{19}$F NMR, 376 MHz, DMSO-$d_6$)**

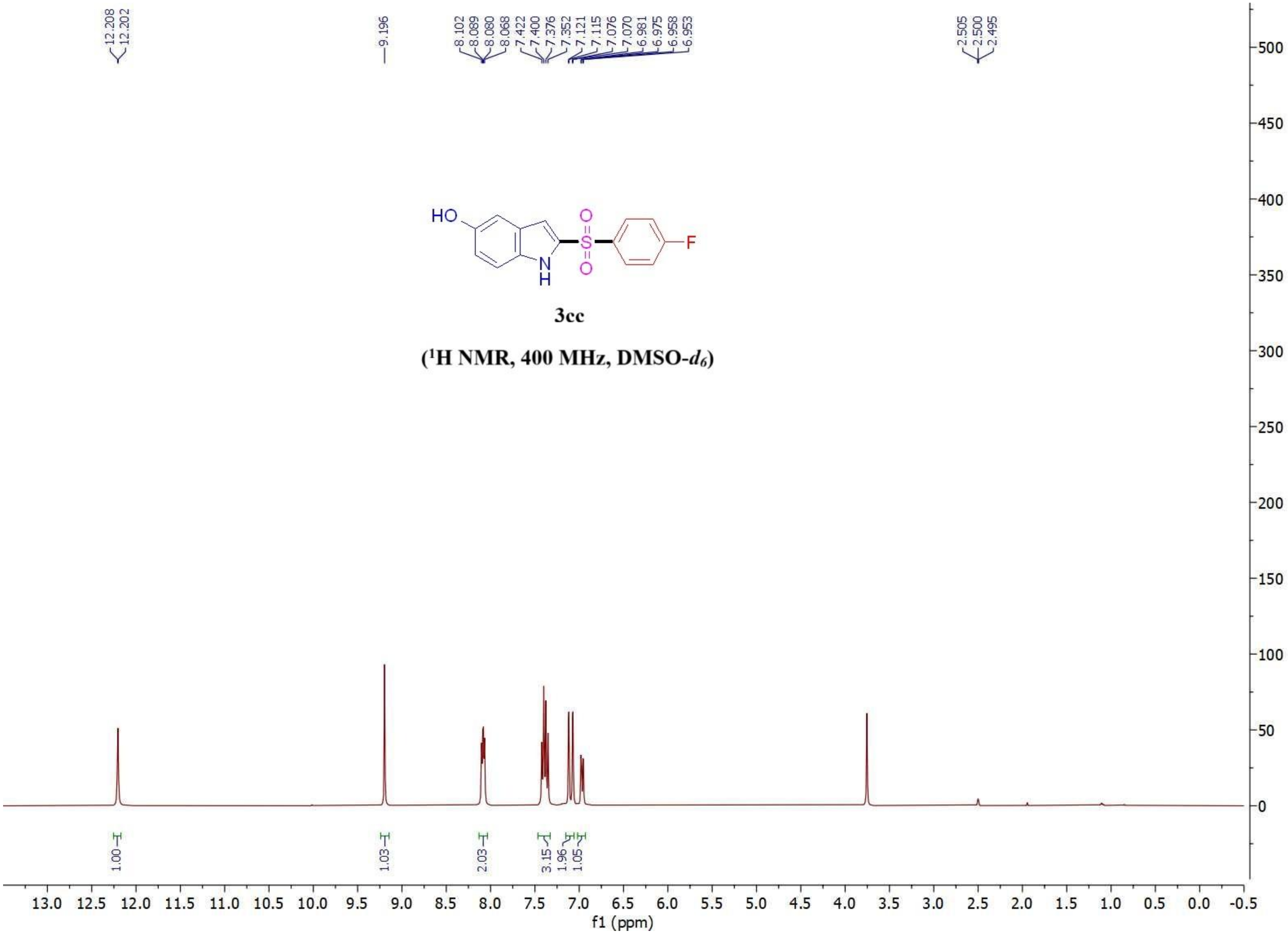


**3cc**

**($^1$H NMR, 400 MHz, DMSO-$d_6$)**

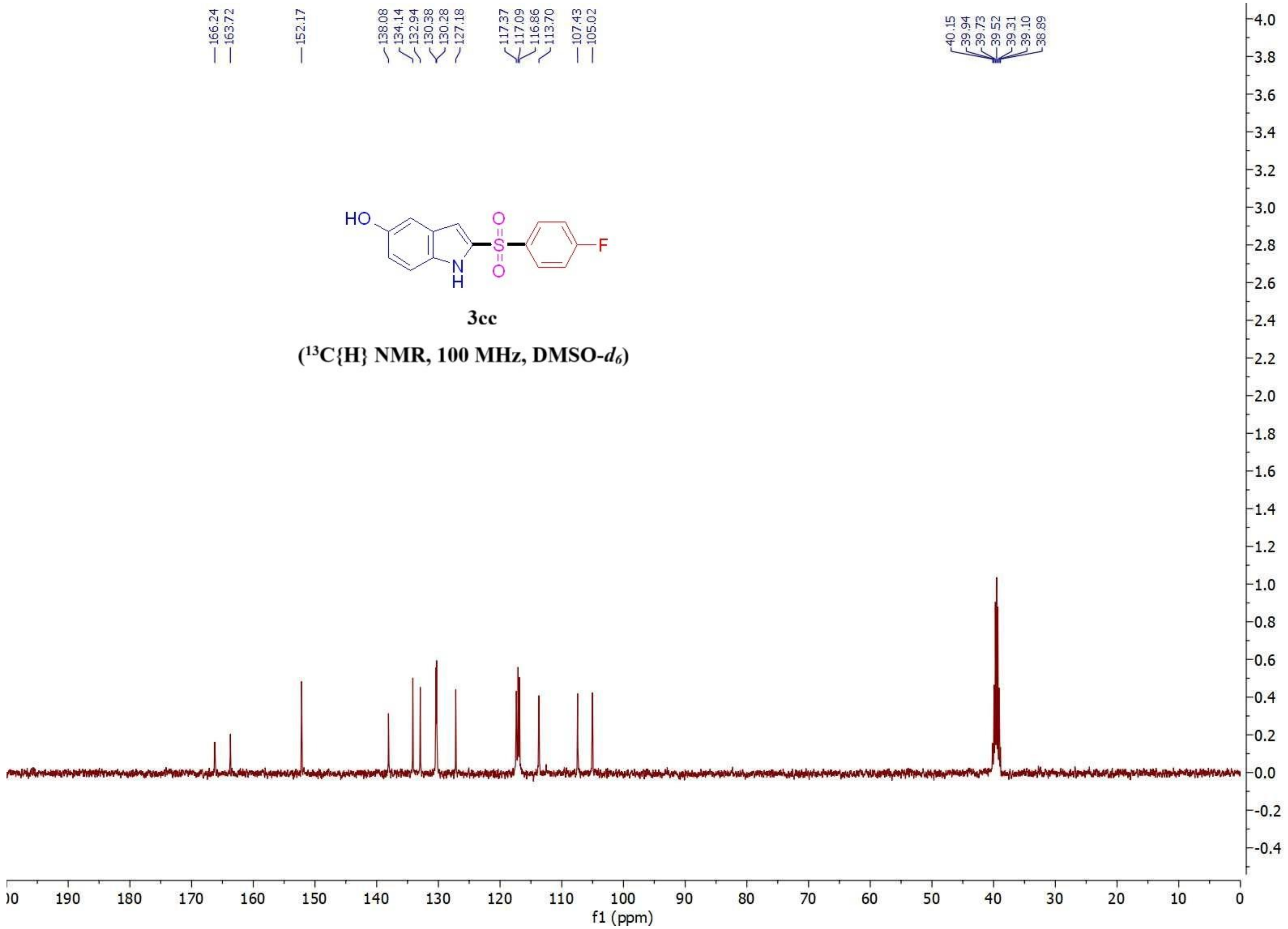


**3cc**

**($^{13}$C{H} NMR, 100 MHz, DMSO-$d_6$)**

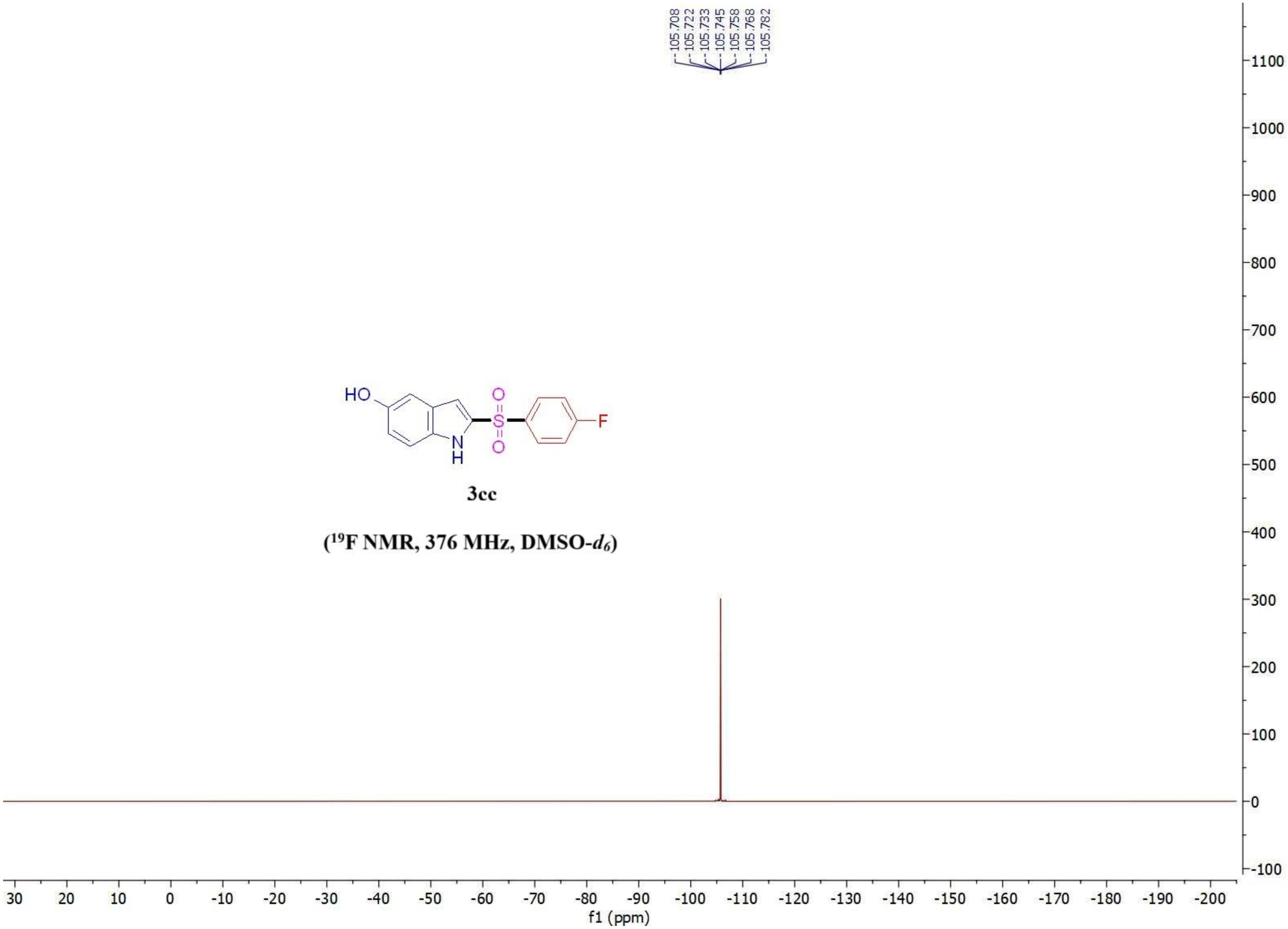


**3cc**

**($^{19}$F NMR, 376 MHz, DMSO-$d_6$)**

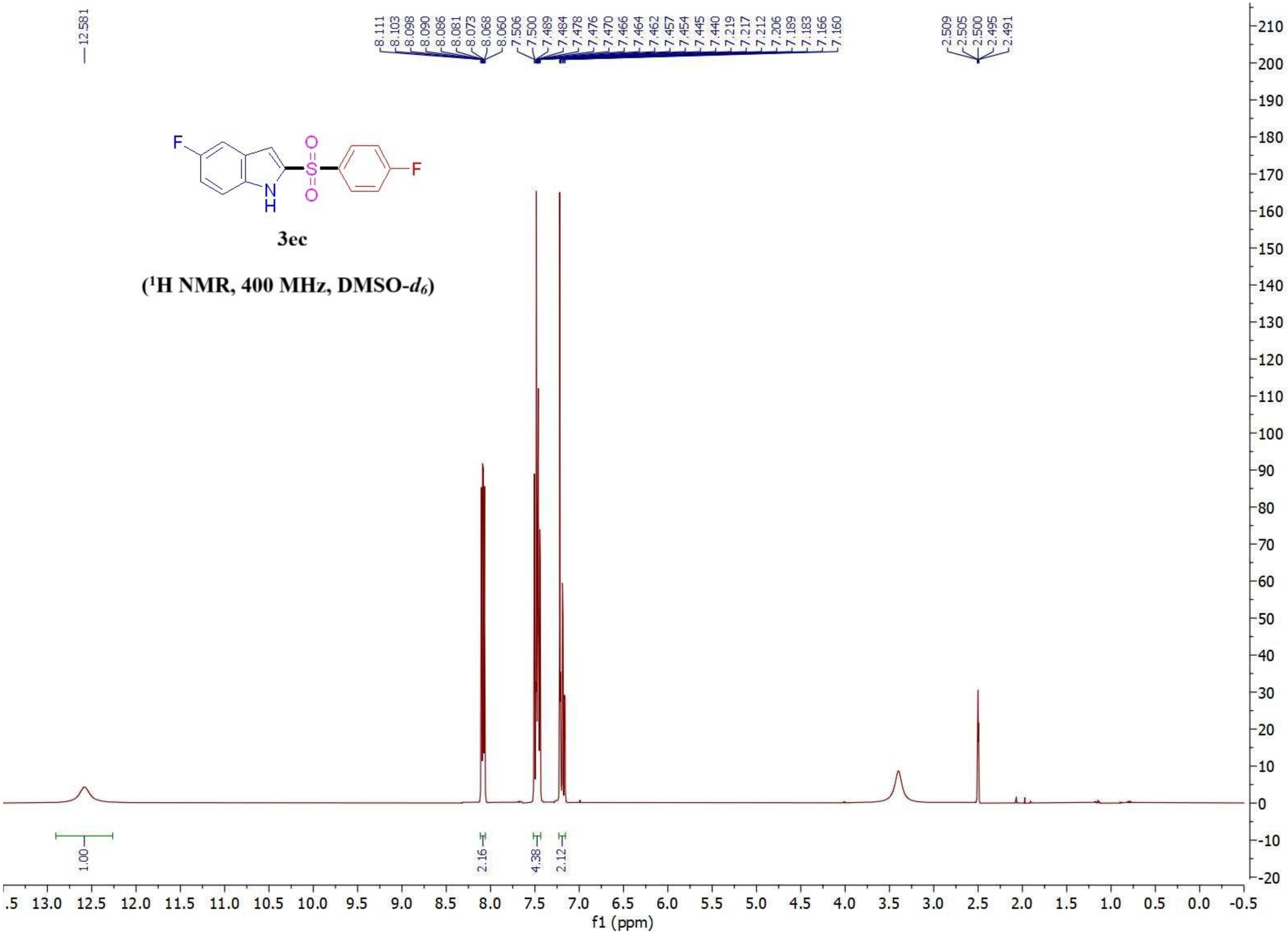
3ec
(¹H NMR, 400 MHz, DMSO-$d_6$)
f1 (ppm)

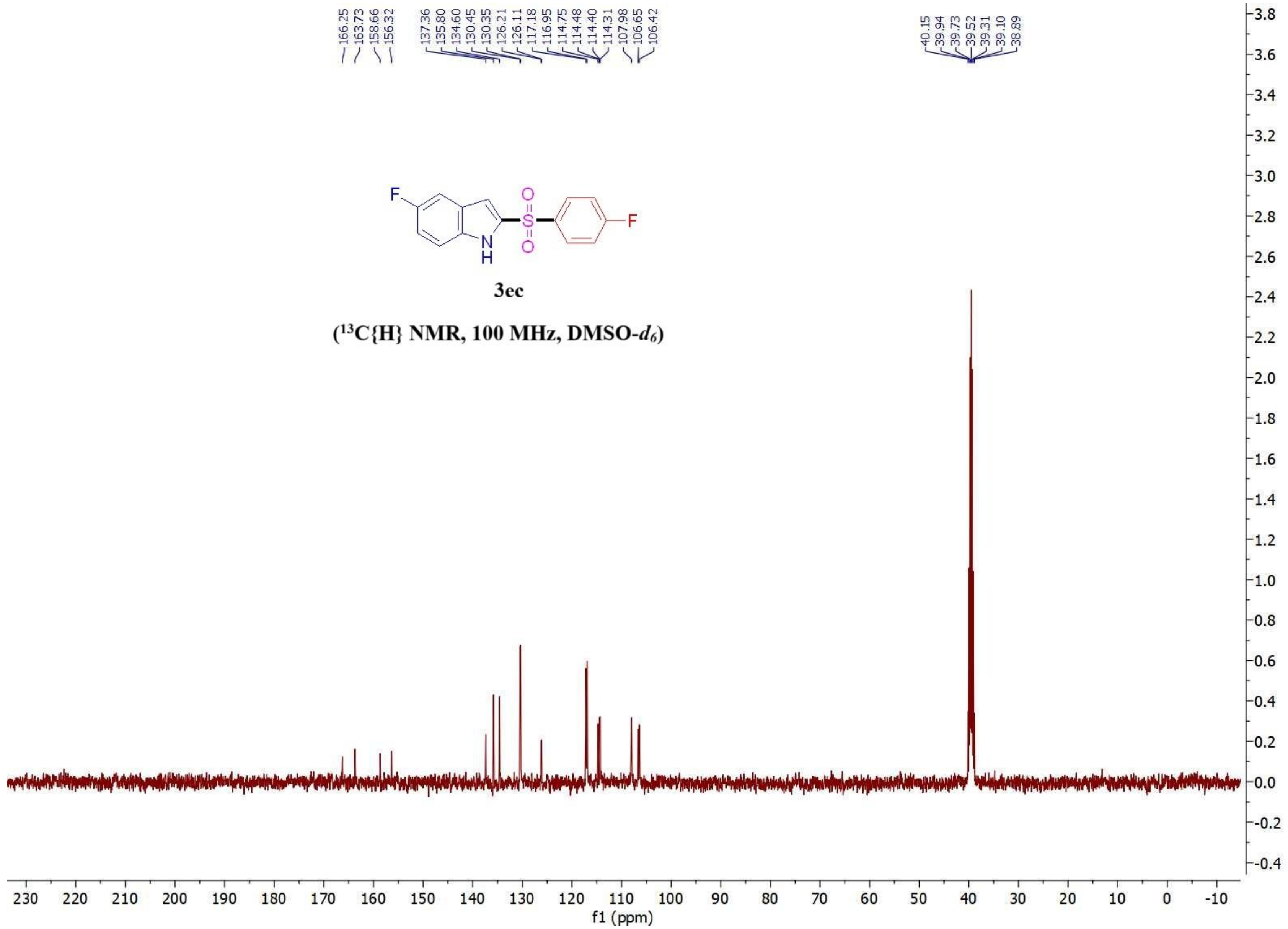


**3ec**

**($^{13}C\{H\}$ NMR, 100 MHz, DMSO-*d*$_6$)**

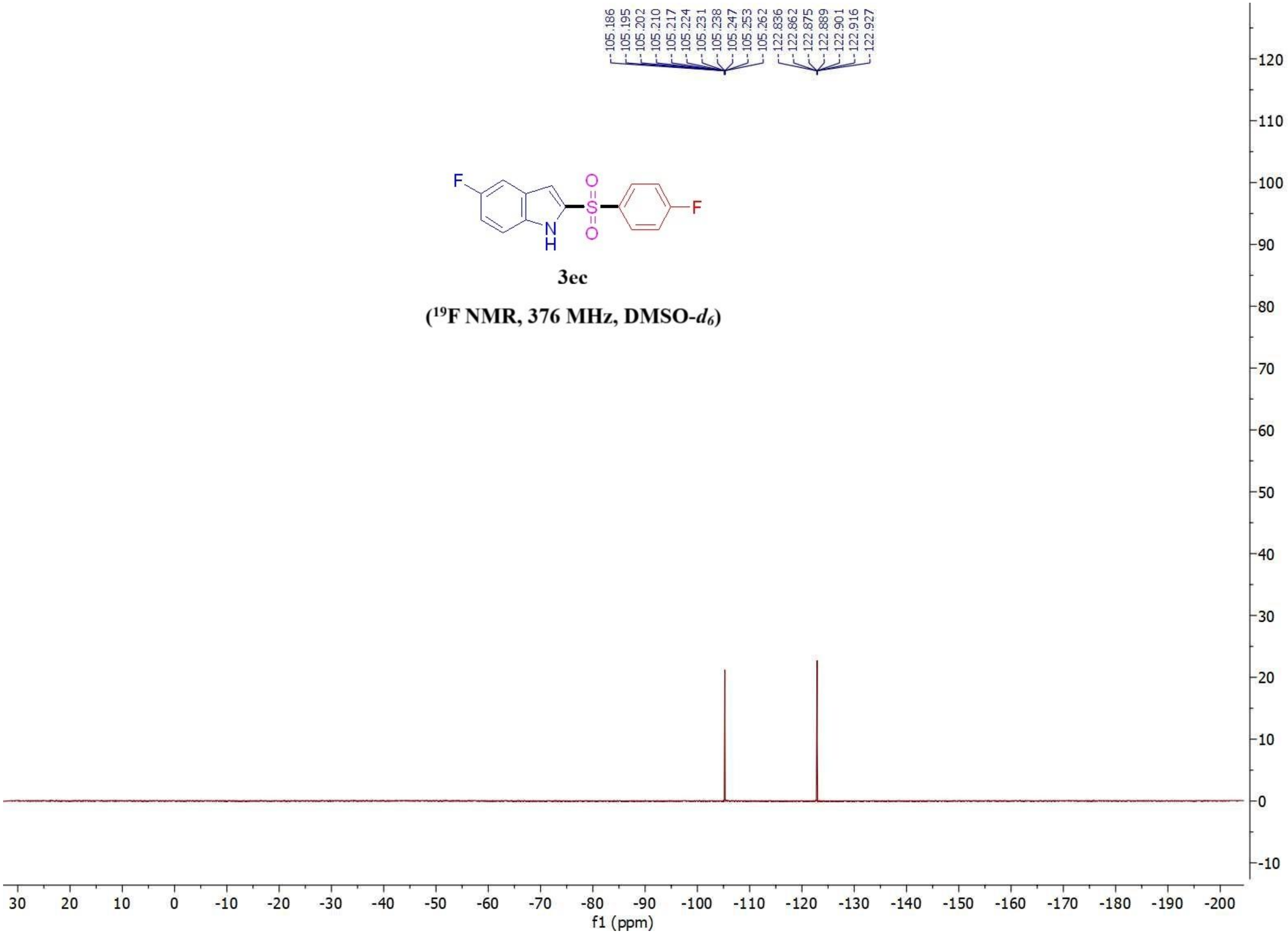
-105.186
-105.195
-105.202
-105.210
-105.217
-105.224
-105.231
-105.238
-105.247
-105.253
-105.262
-122.836
-122.862
-122.875
-122.889
-122.901
-122.916
-122.927
3ec
(19F NMR, 376 MHz, DMSO-d6)
f1 (ppm)

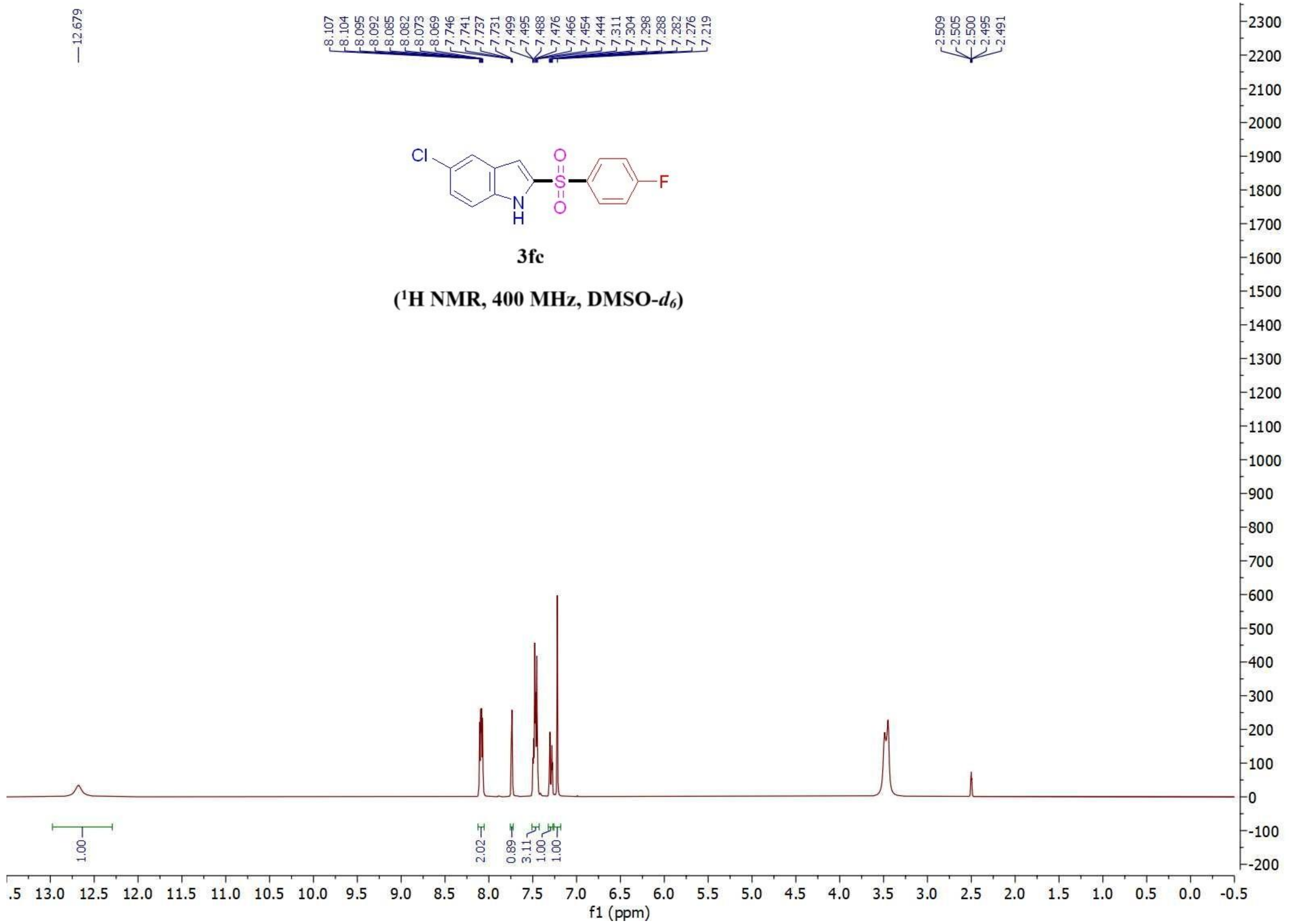
12.679
8.107
8.104
8.095
8.092
8.085
8.082
8.073
8.069
7.746
7.741
7.737
7.731
7.499
7.495
7.488
7.476
7.466
7.454
7.444
7.311
7.304
7.298
7.288
7.282
7.276
7.219
2.509
2.505
2.500
2.495
2.491
Cl
O
S
O
F
N
H
3fc
(¹H NMR, 400 MHz, DMSO-d6)
1.00
2.02
0.89
3.11
1.00
1.00
f1 (ppm)

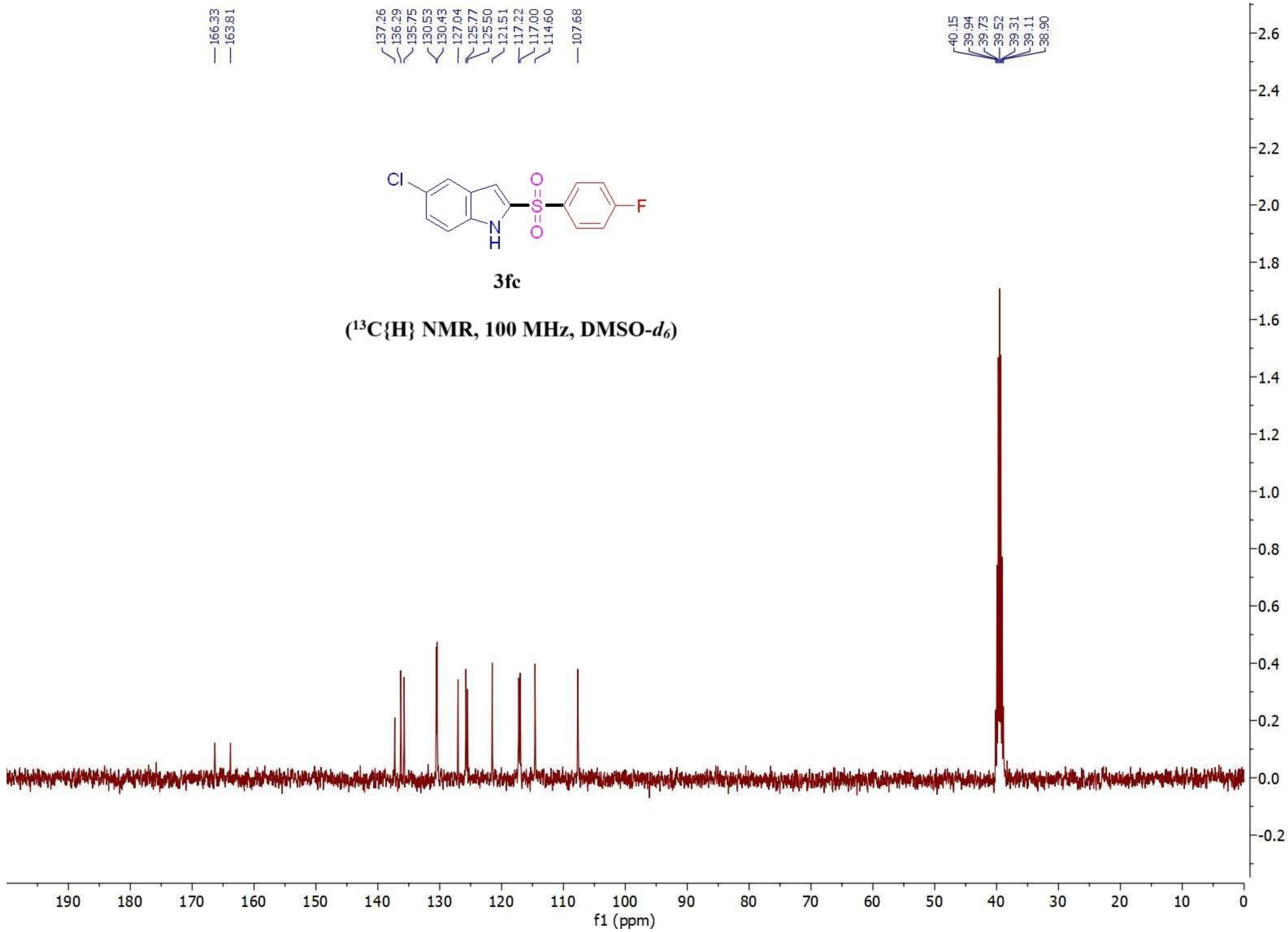
166.33
163.81
137.26
136.29
135.75
130.53
130.43
127.04
125.77
125.50
121.51
117.22
117.00
114.60
107.68
40.15
39.94
39.73
39.52
39.31
39.11
38.90
Cl
O
S
O
N
H
F
3fc
($^{13}$C{H} NMR, 100 MHz, DMSO-$d_6$)
f1 (ppm)

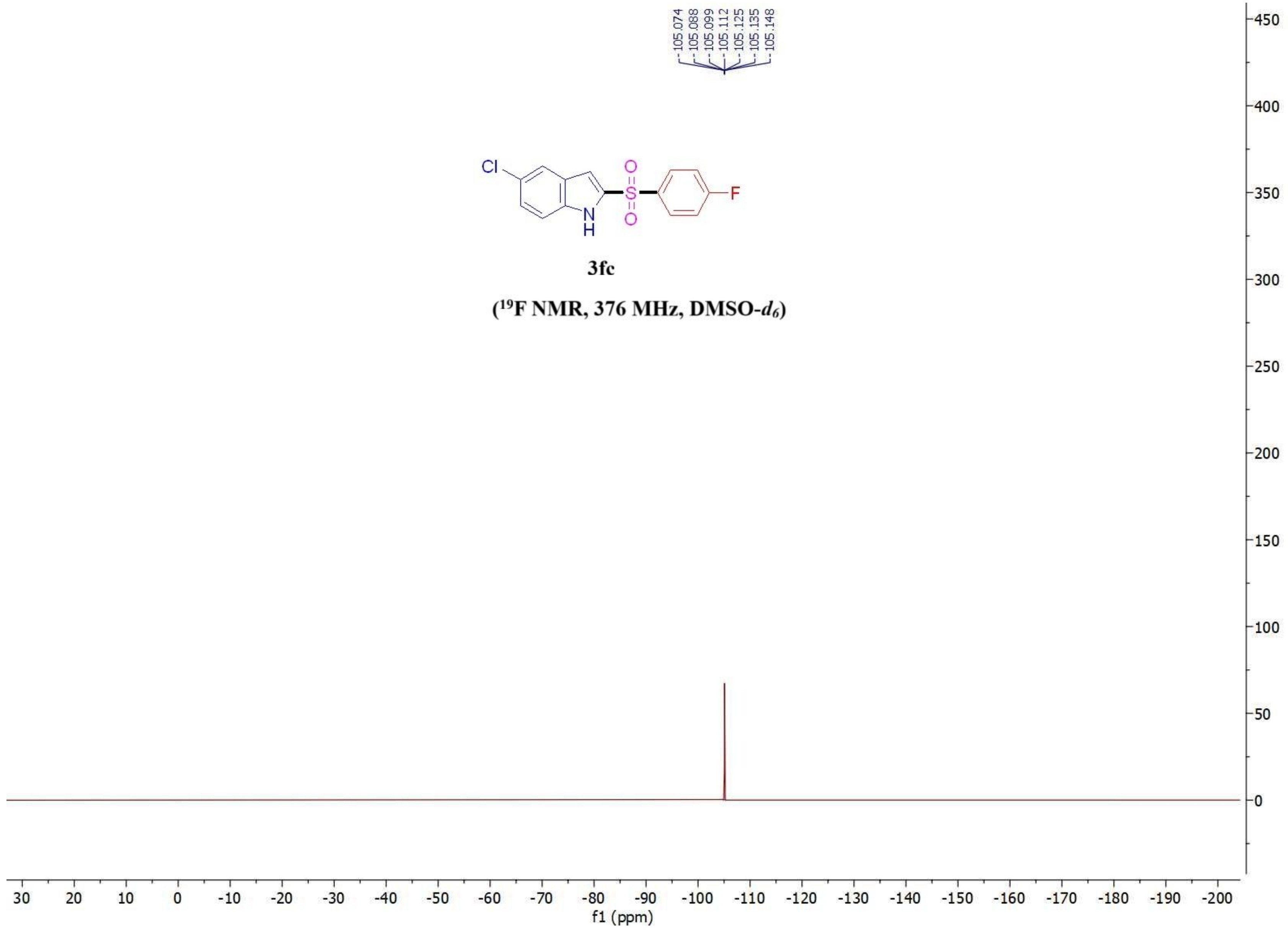


**3fc**

**($^{19}$F NMR, 376 MHz, DMSO-$d_6$)**

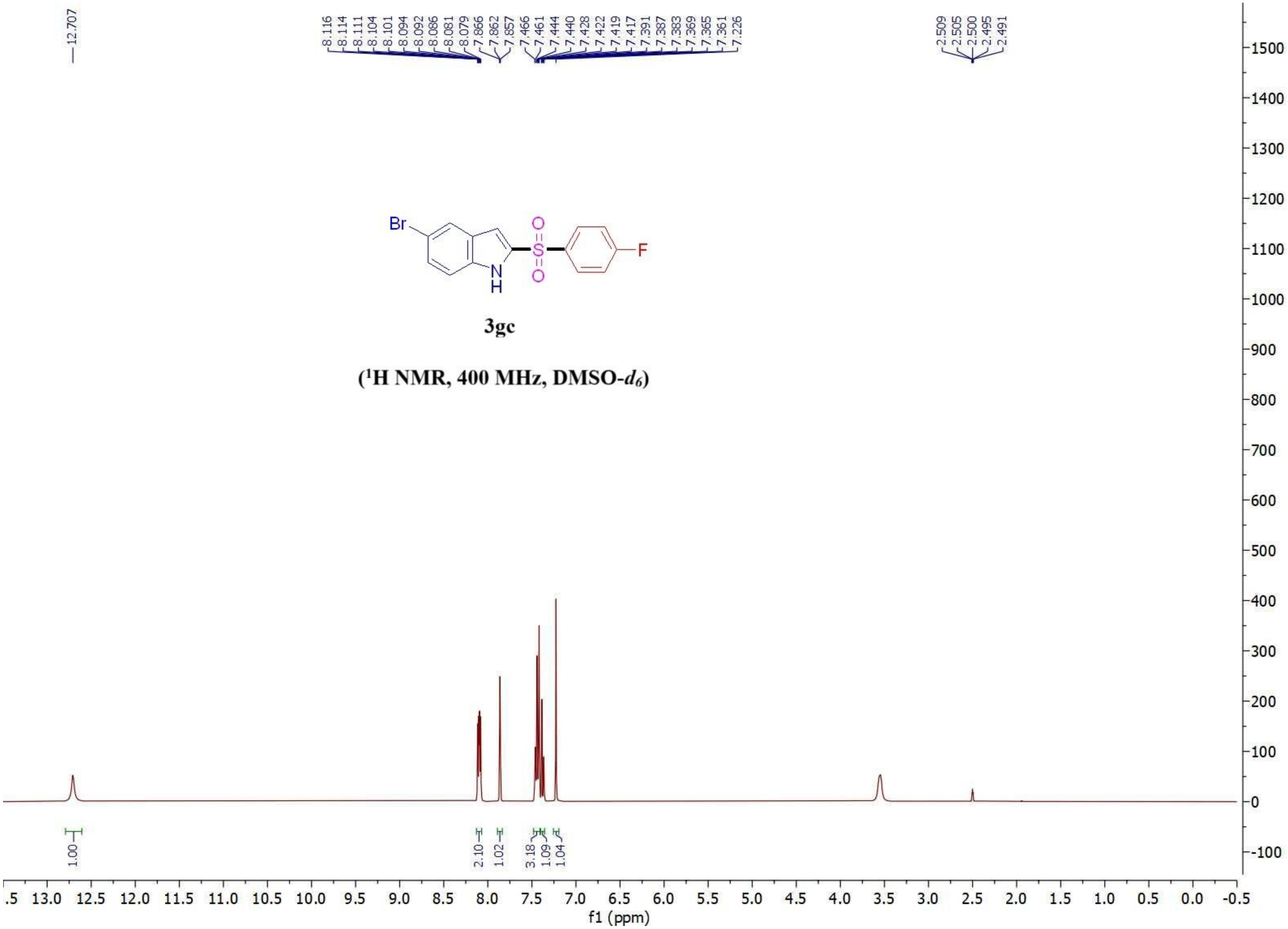


**3gc**

**($^1$H NMR, 400 MHz, DMSO-$d_6$)**

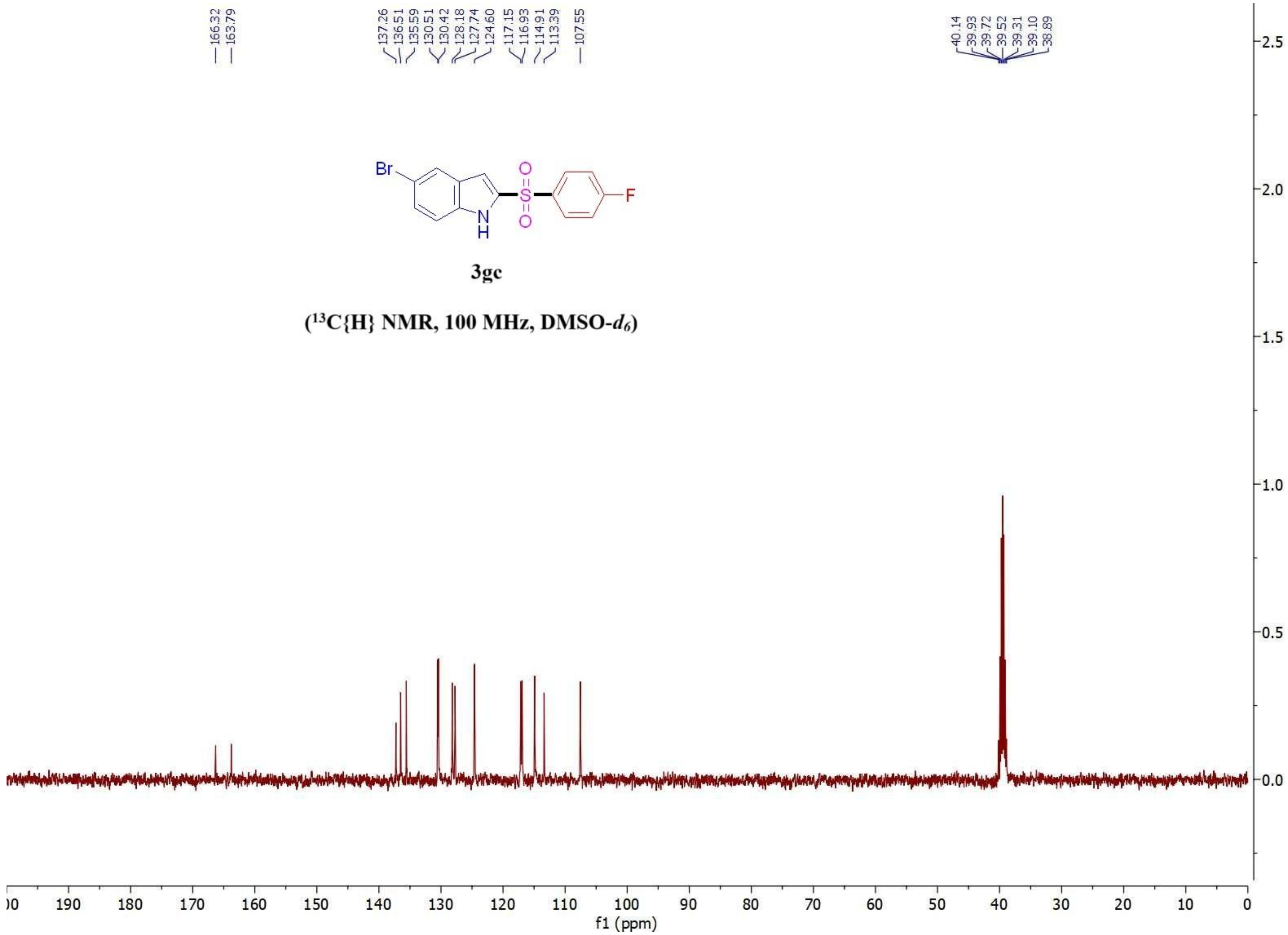


**3gc**

**($^{13}$C{H} NMR, 100 MHz, DMSO-$d_6$)**

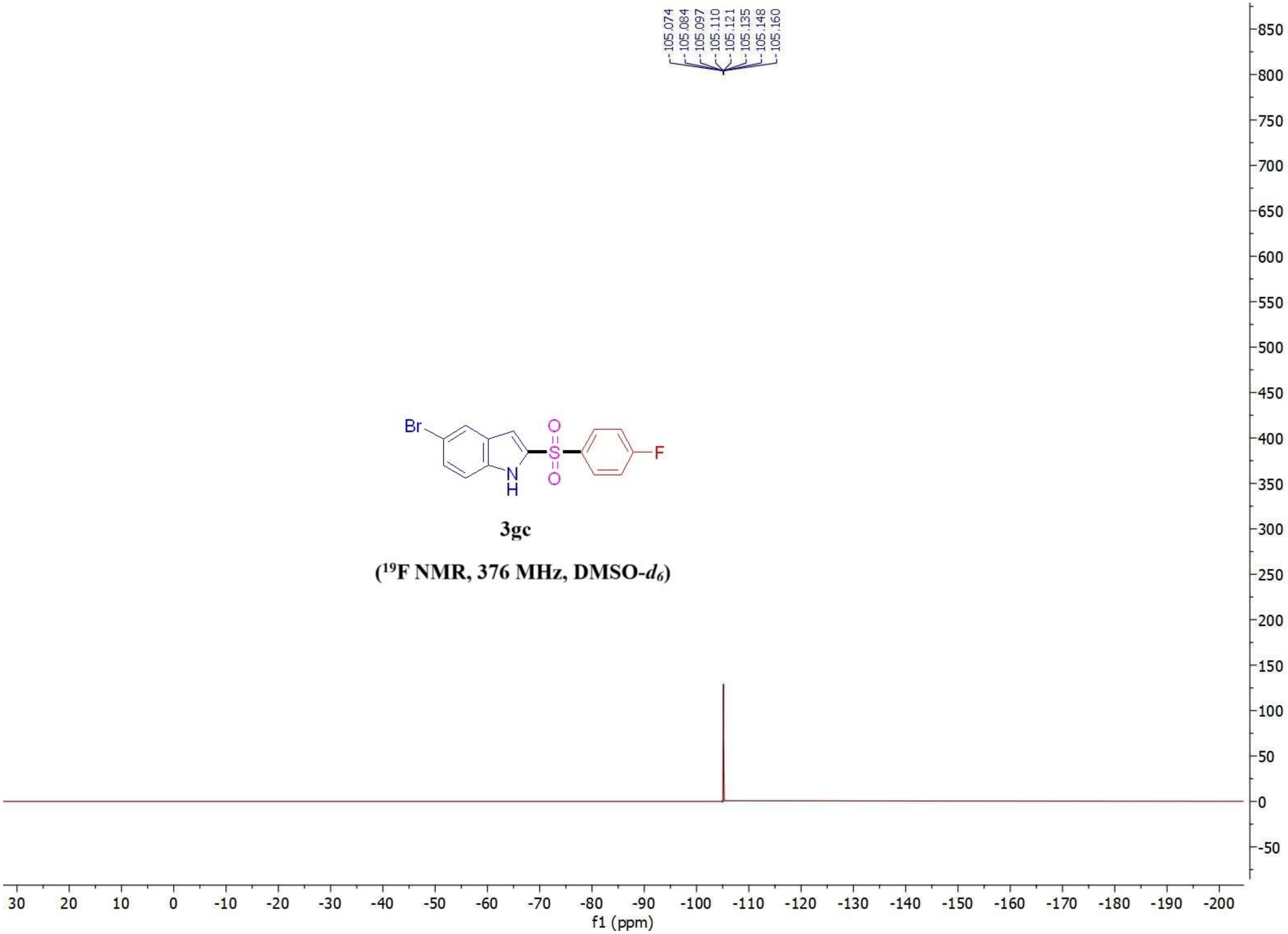
-105.074
-105.084
-105.097
-105.110
-105.121
-105.135
-105.148
-105.160
Br
O
S
O
N
H
F
3gc
(19F NMR, 376 MHz, DMSO-d6)
f1 (ppm)

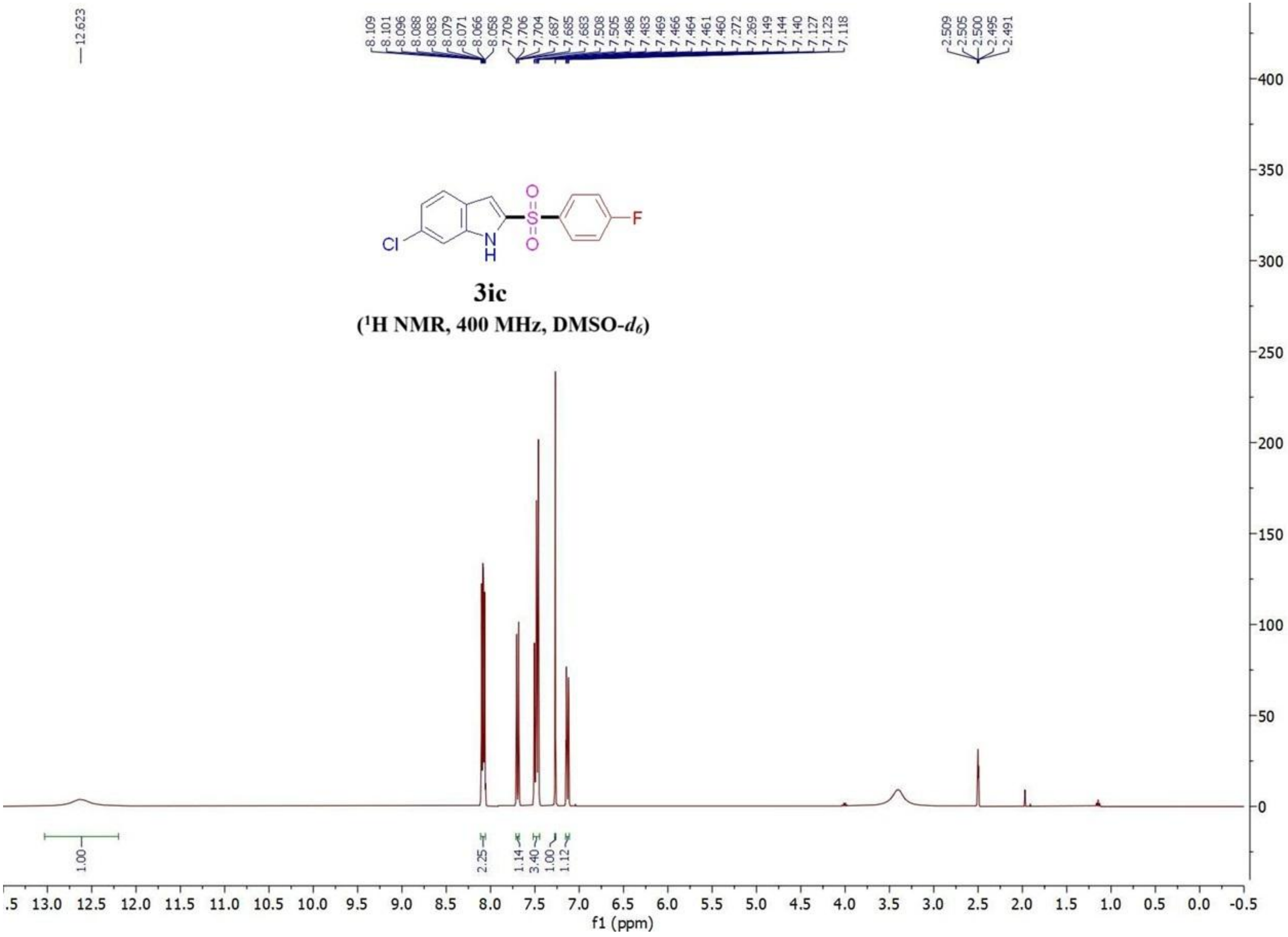

12.623
8.109
8.101
8.096
8.088
8.083
8.079
8.071
8.066
8.058
7.709
7.706
7.704
7.687
7.685
7.683
7.508
7.505
7.486
7.483
7.469
7.466
7.464
7.461
7.460
7.272
7.269
7.149
7.144
7.140
7.127
7.123
7.118
2.509
2.505
2.500
2.495
2.491
Cl
N
H
S
O
O
F
3ic
(¹H NMR, 400 MHz, DMSO-d6)
1.00
2.25
1.14
3.40
1.00
1.12
f1 (ppm)

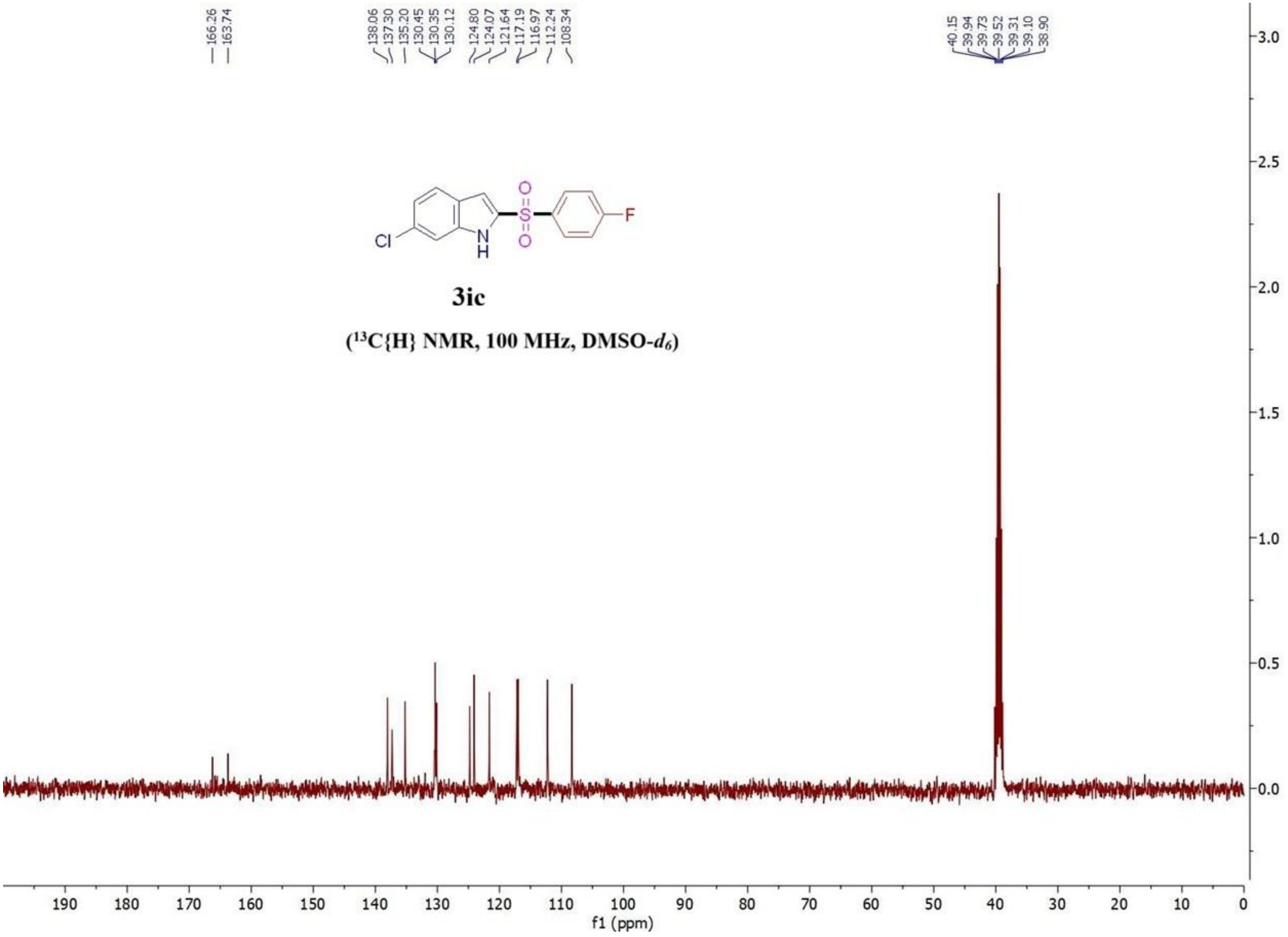


**3ic**

**($^{13}$C{H} NMR, 100 MHz, DMSO-$d_6$)**

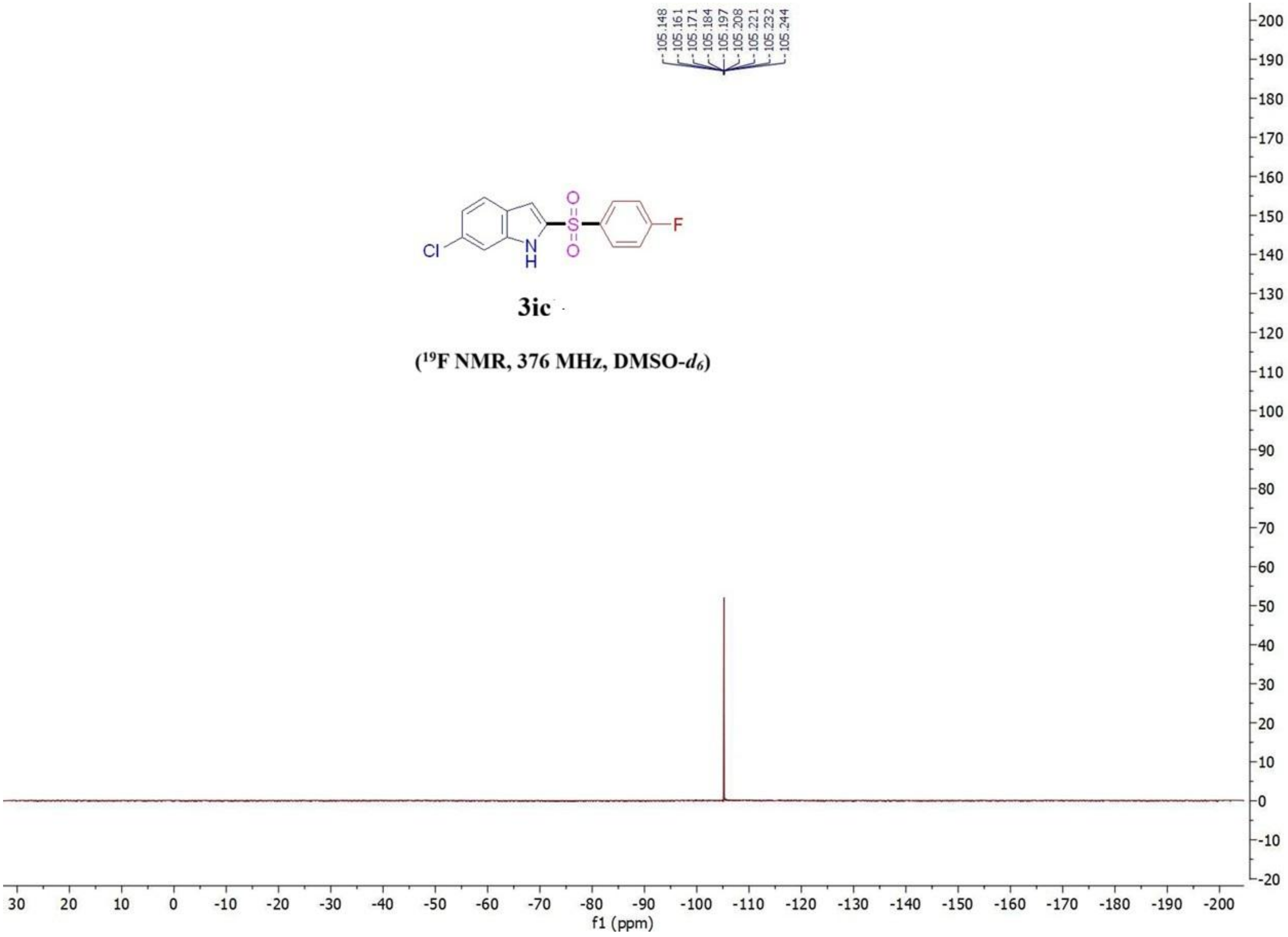


**3ic**

**($^{19}$F NMR, 376 MHz, DMSO-*d*$_6$)**

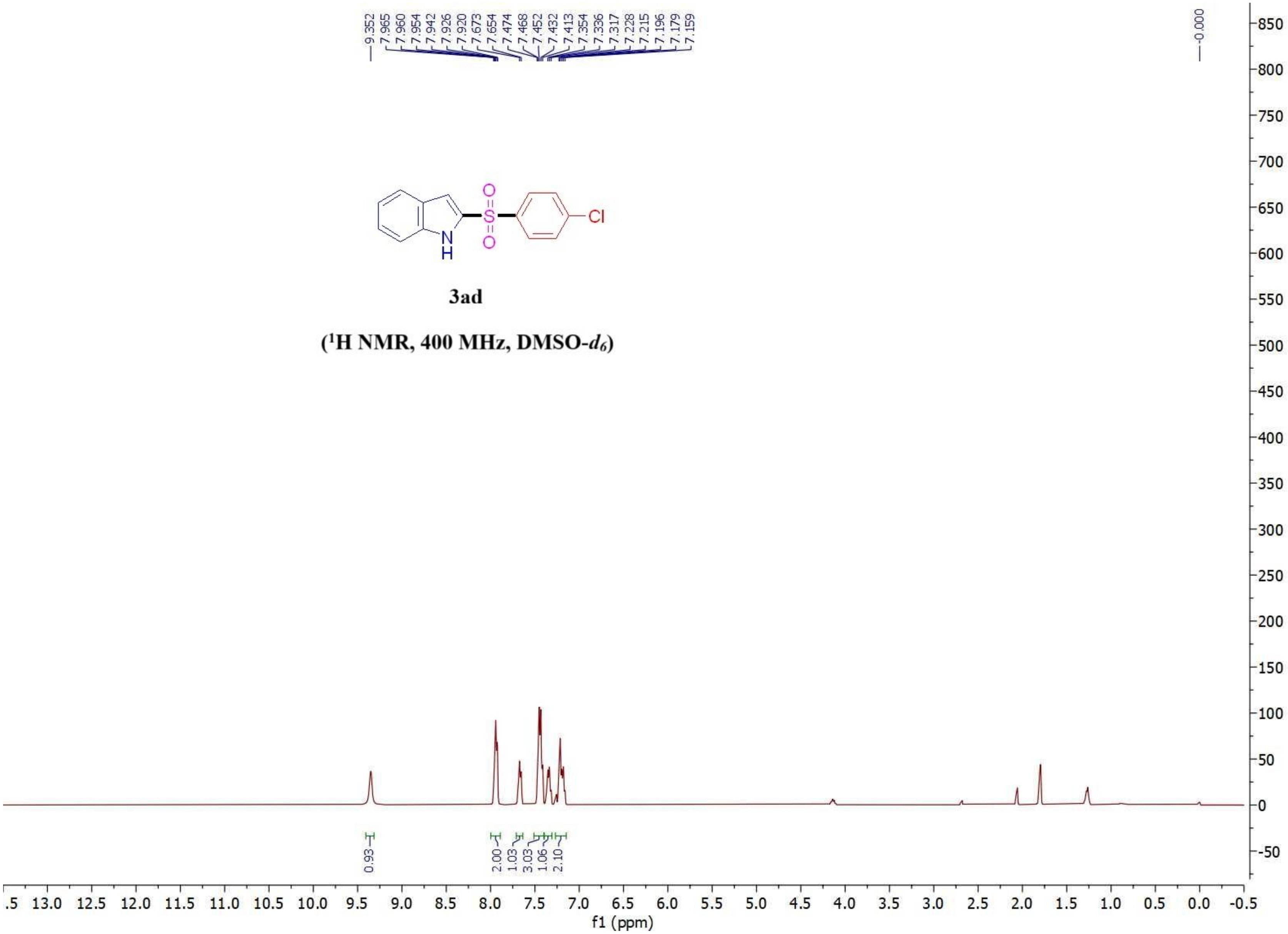
3ad
(1H NMR, 400 MHz, DMSO-d6)
9.352
7.965
7.960
7.954
7.942
7.926
7.920
7.673
7.654
7.474
7.468
7.452
7.432
7.413
7.354
7.336
7.317
7.228
7.215
7.196
7.179
7.159
0.000
0.93
2.00
1.03
3.03
1.06
2.10
f1 (ppm)

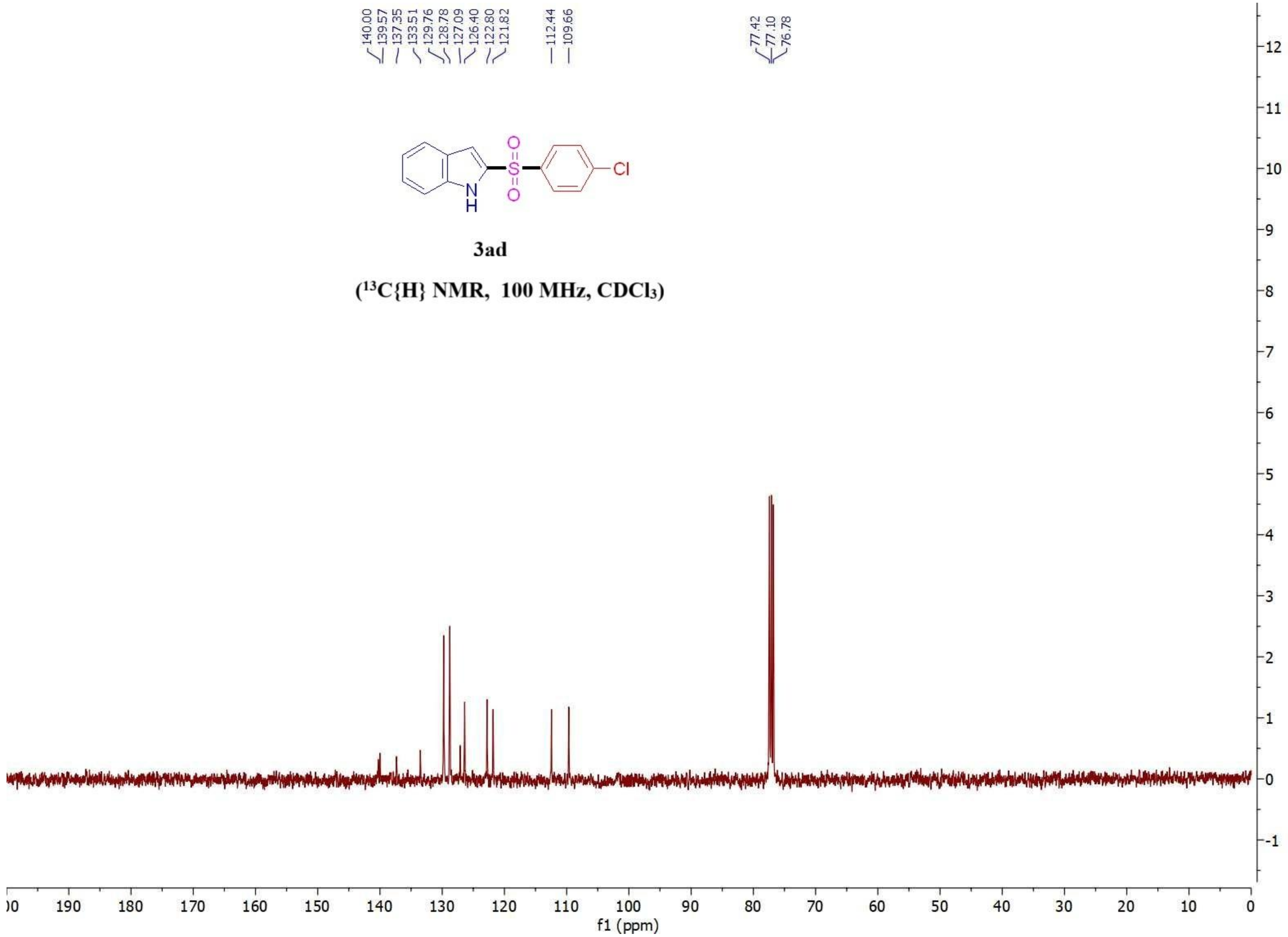
140.00
139.57
137.35
133.51
129.76
128.78
127.09
126.40
122.80
121.82
112.44
109.66
77.42
77.10
76.78
3ad
($^{13}$C{H} NMR, 100 MHz, CDCl$_3$)
f1 (ppm)

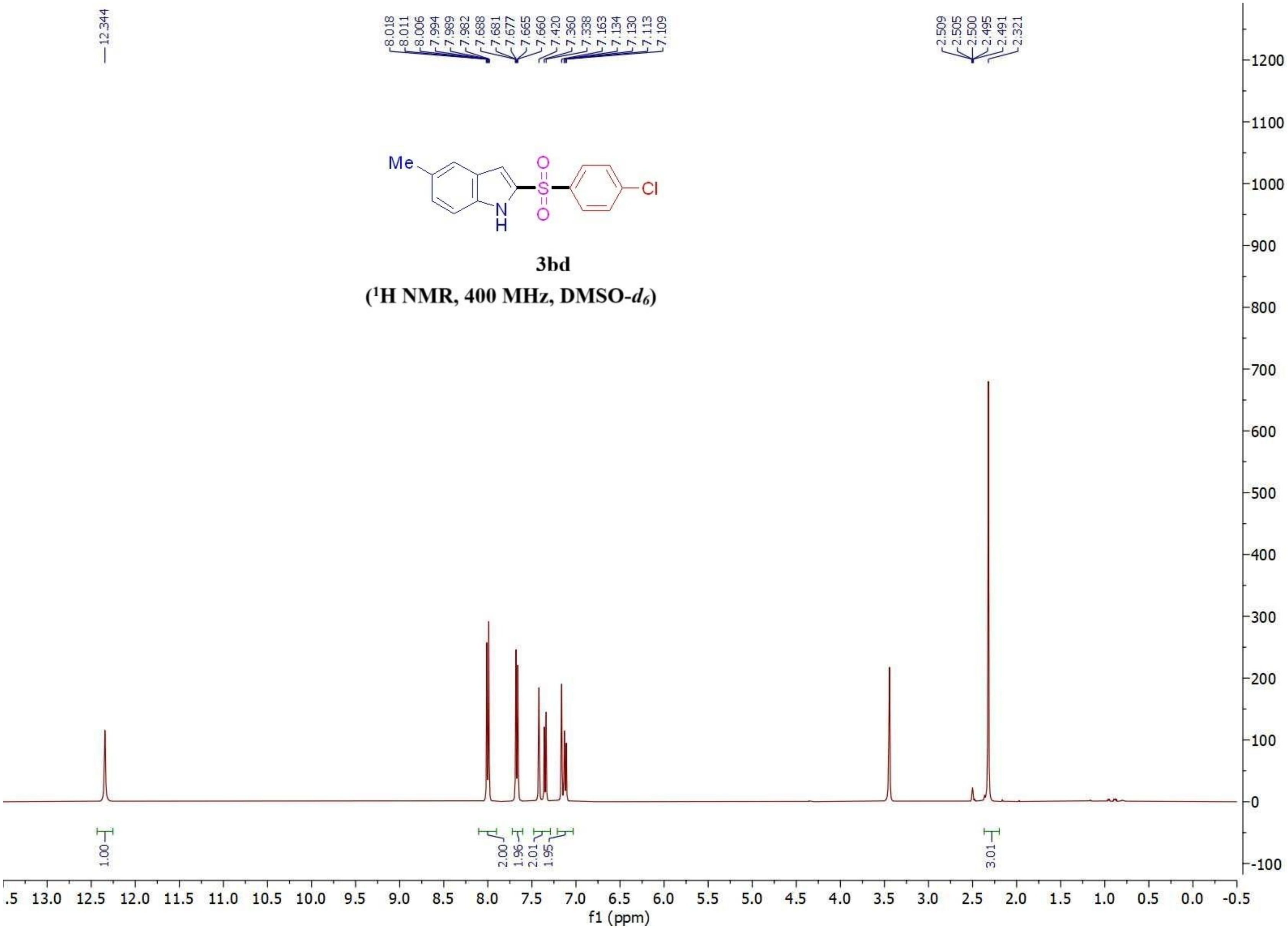


**3bd**

**($^1$H NMR, 400 MHz, DMSO-$d_6$)**

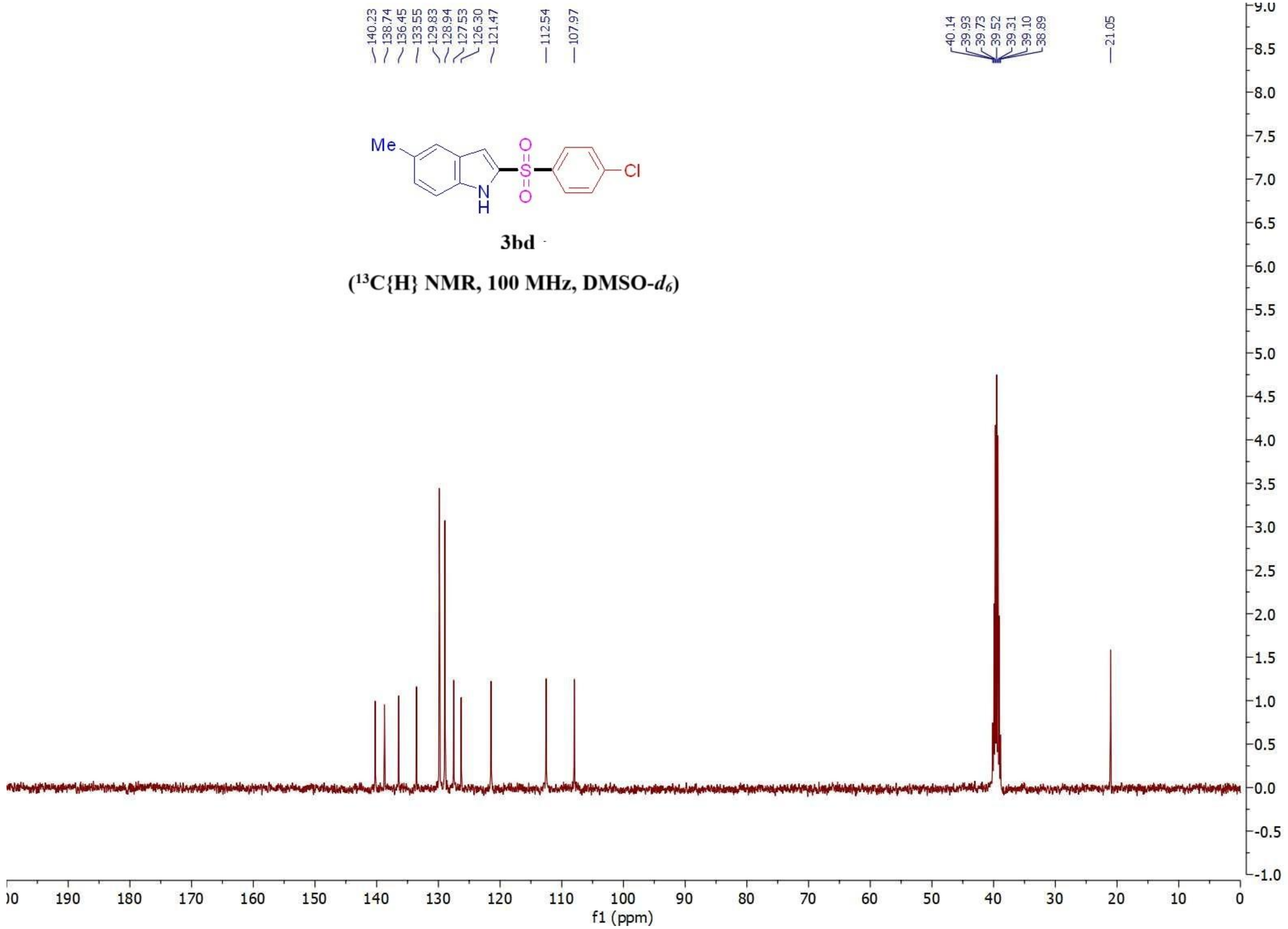


**3bd**

($^{13}C\{H\}$ NMR, 100 MHz, DMSO-$d_6$)

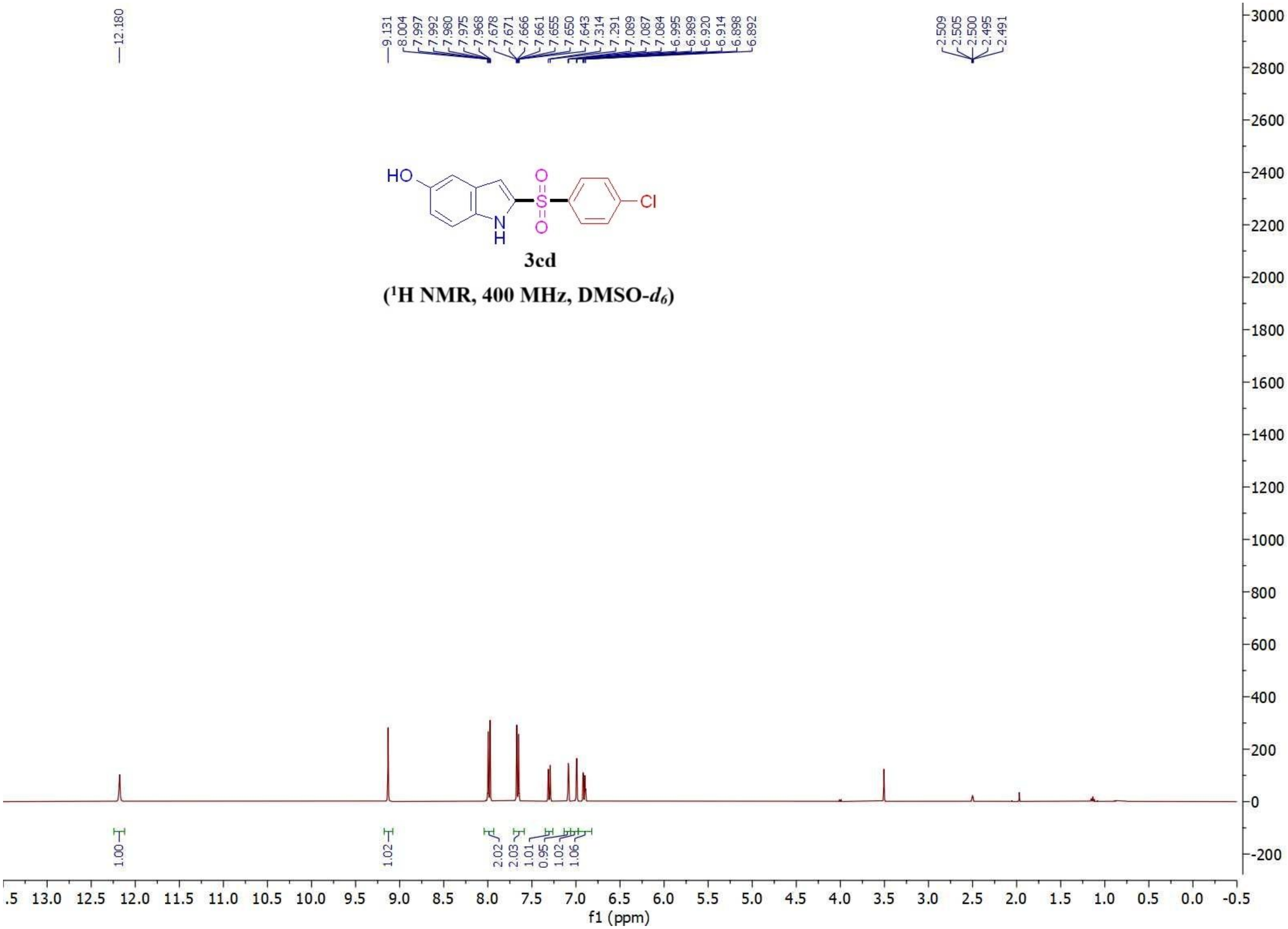

**3cd**

**($^1$H NMR, 400 MHz, DMSO-$d_6$)**

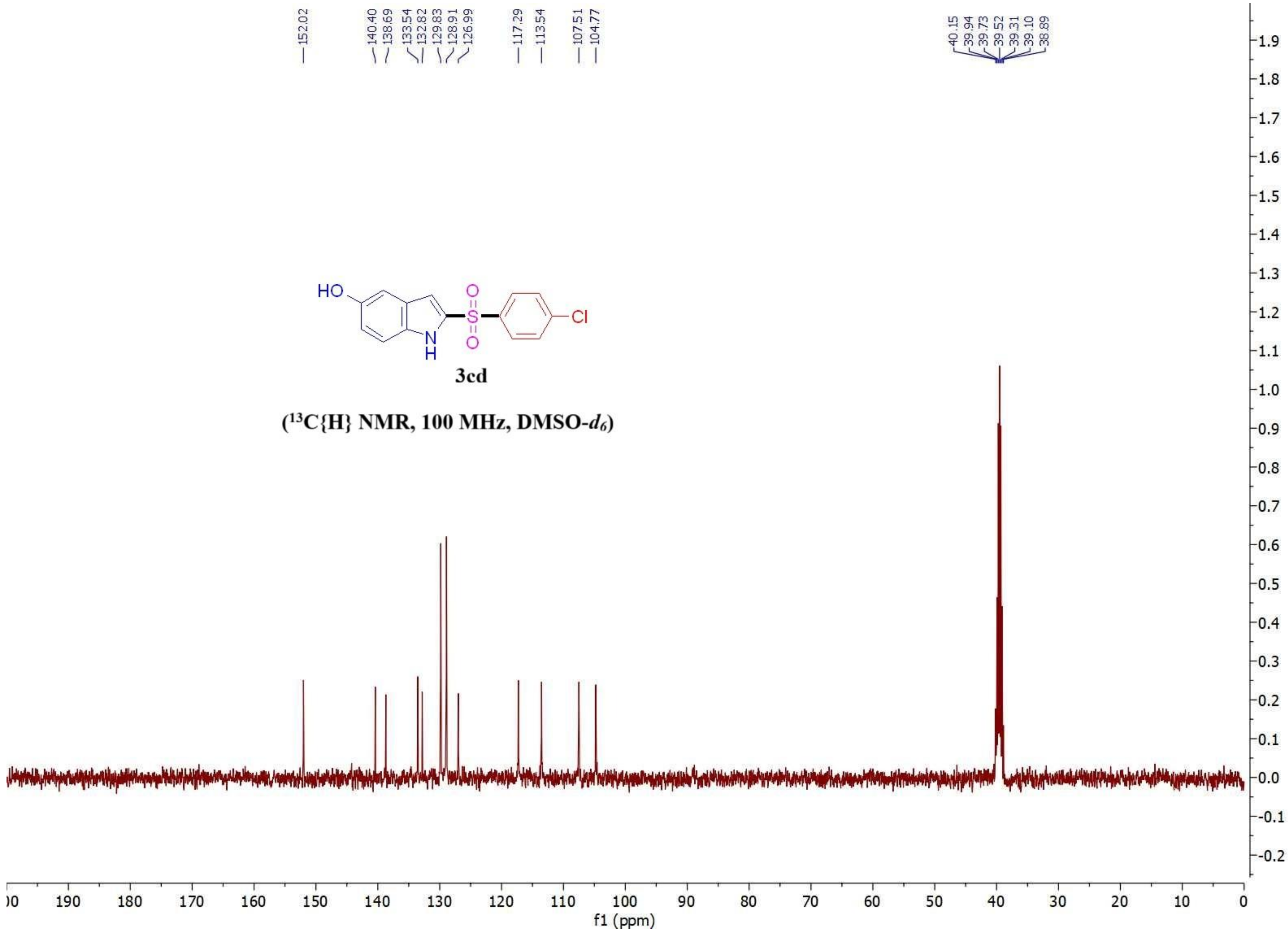

152.02
140.40
138.69
133.54
132.82
129.83
128.91
126.99
117.29
113.54
107.51
104.77
40.15
39.94
39.73
39.52
39.31
39.10
38.89
HO
S
O
O
N
H
Cl
3cd
(13C{H} NMR, 100 MHz, DMSO-d6)
f1 (ppm)

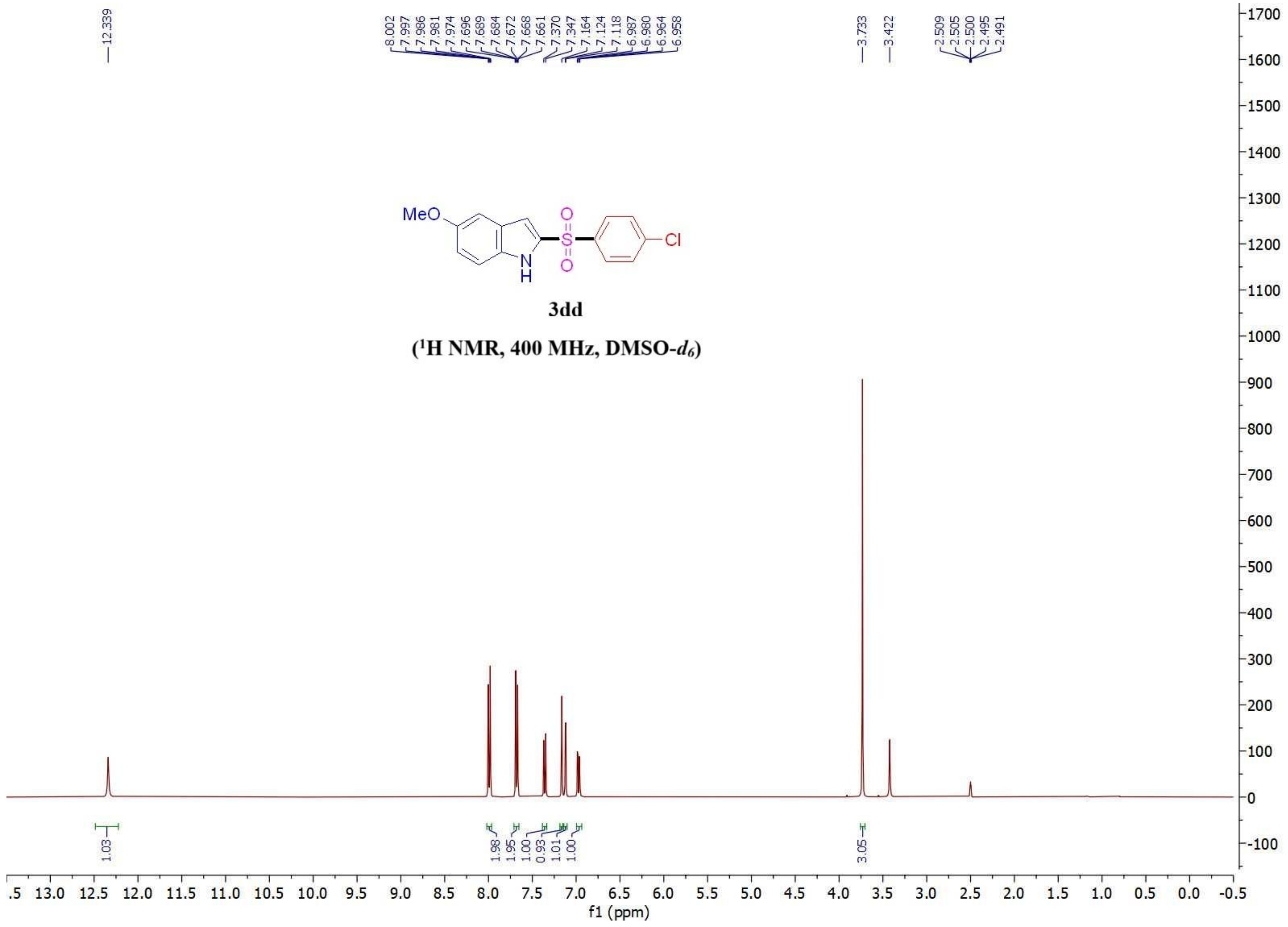
3dd
($^1$H NMR, 400 MHz, DMSO-$d_6$)
MeO
N
H
O
S
O
Cl
f1 (ppm)

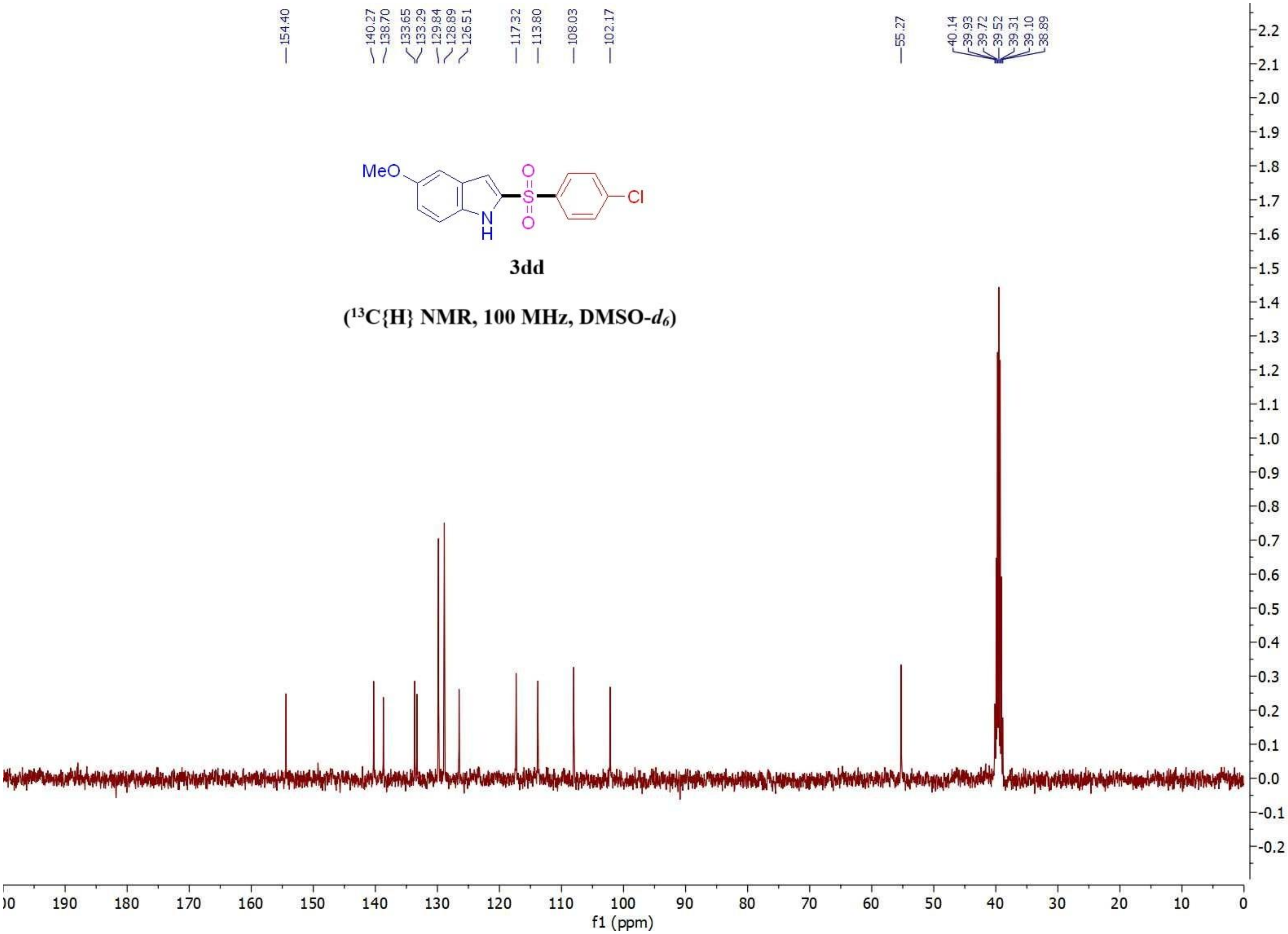


**3dd**

**($^{13}$C{H} NMR, 100 MHz, DMSO-$d_6$)**

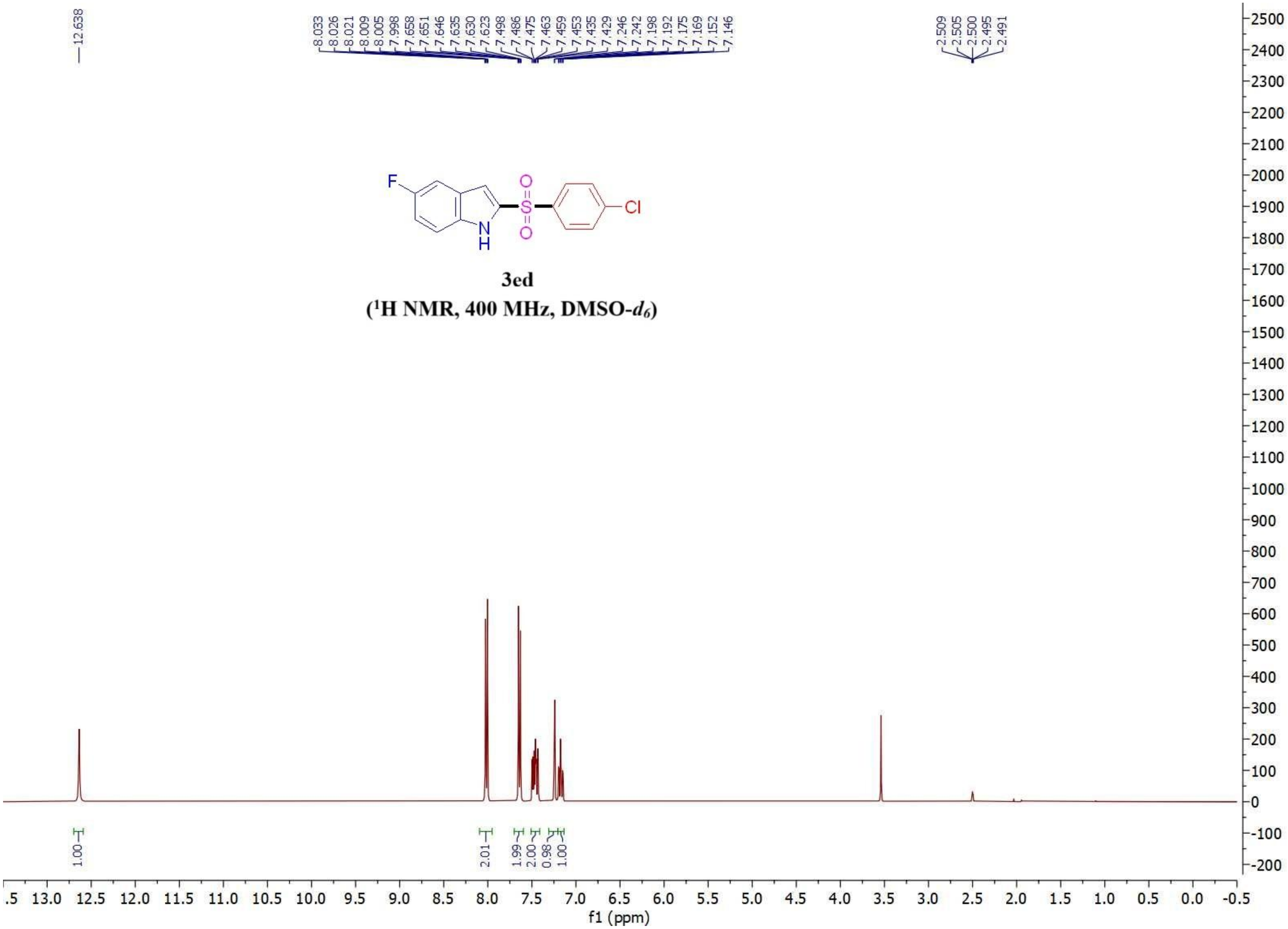

**3ed**
**($^{1}$H NMR, 400 MHz, DMSO-$d_6$)**

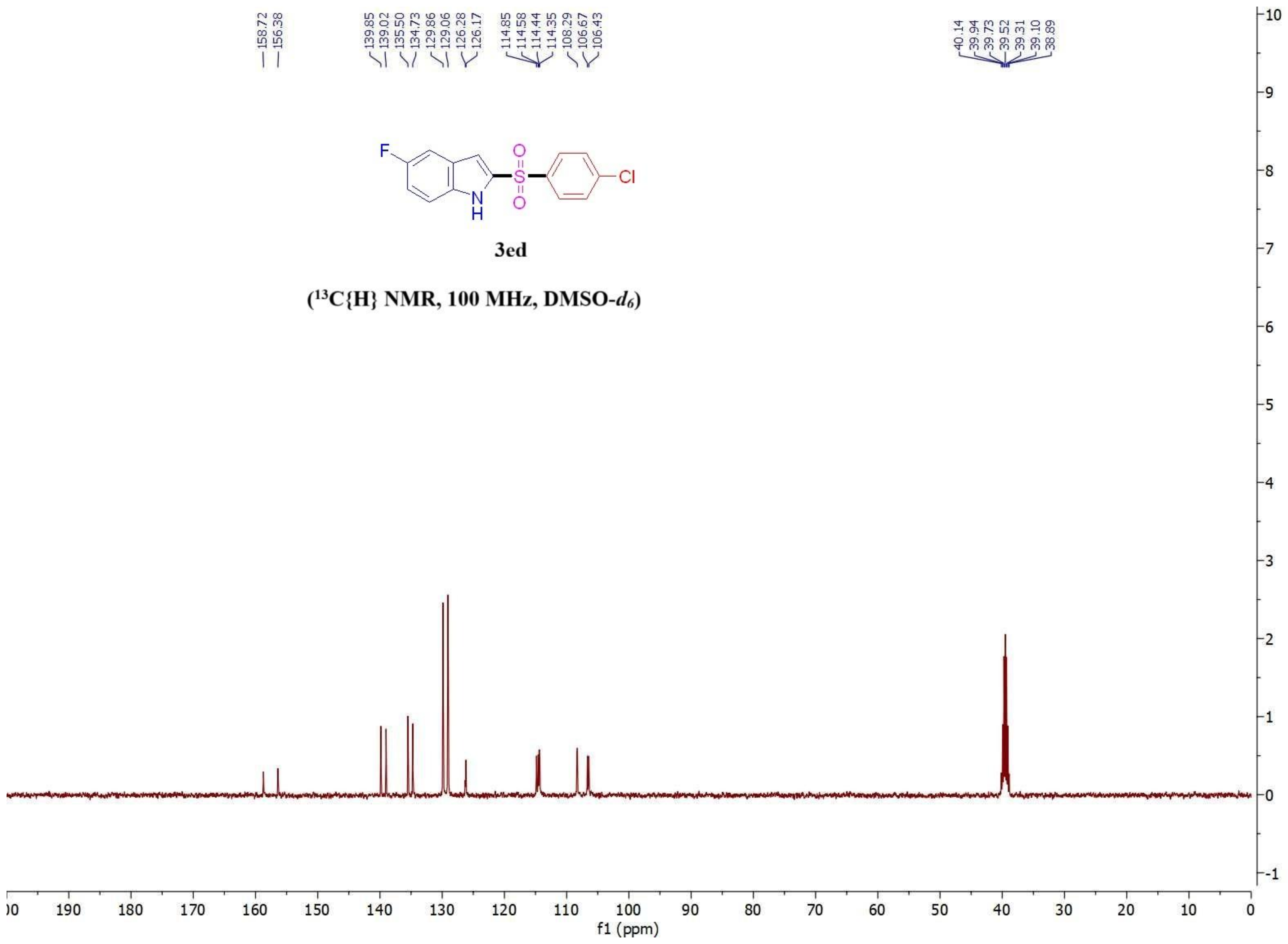


**3ed**

**($^{13}$C{H} NMR, 100 MHz, DMSO-$d_6$)**

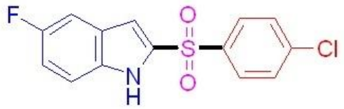

**3ed**

**($^{19}$F NMR, 376 MHz, DMSO-$d_6$)**

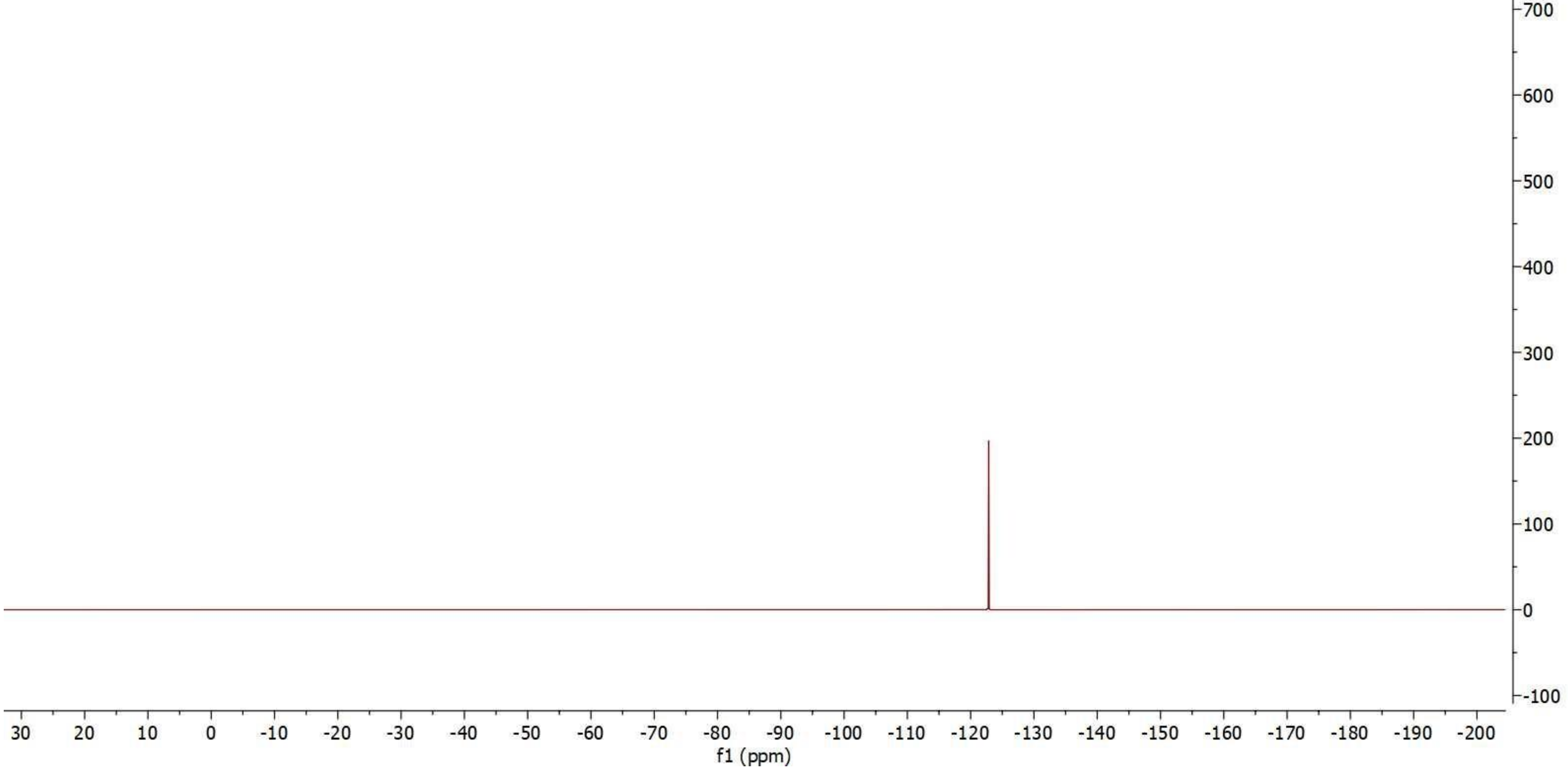

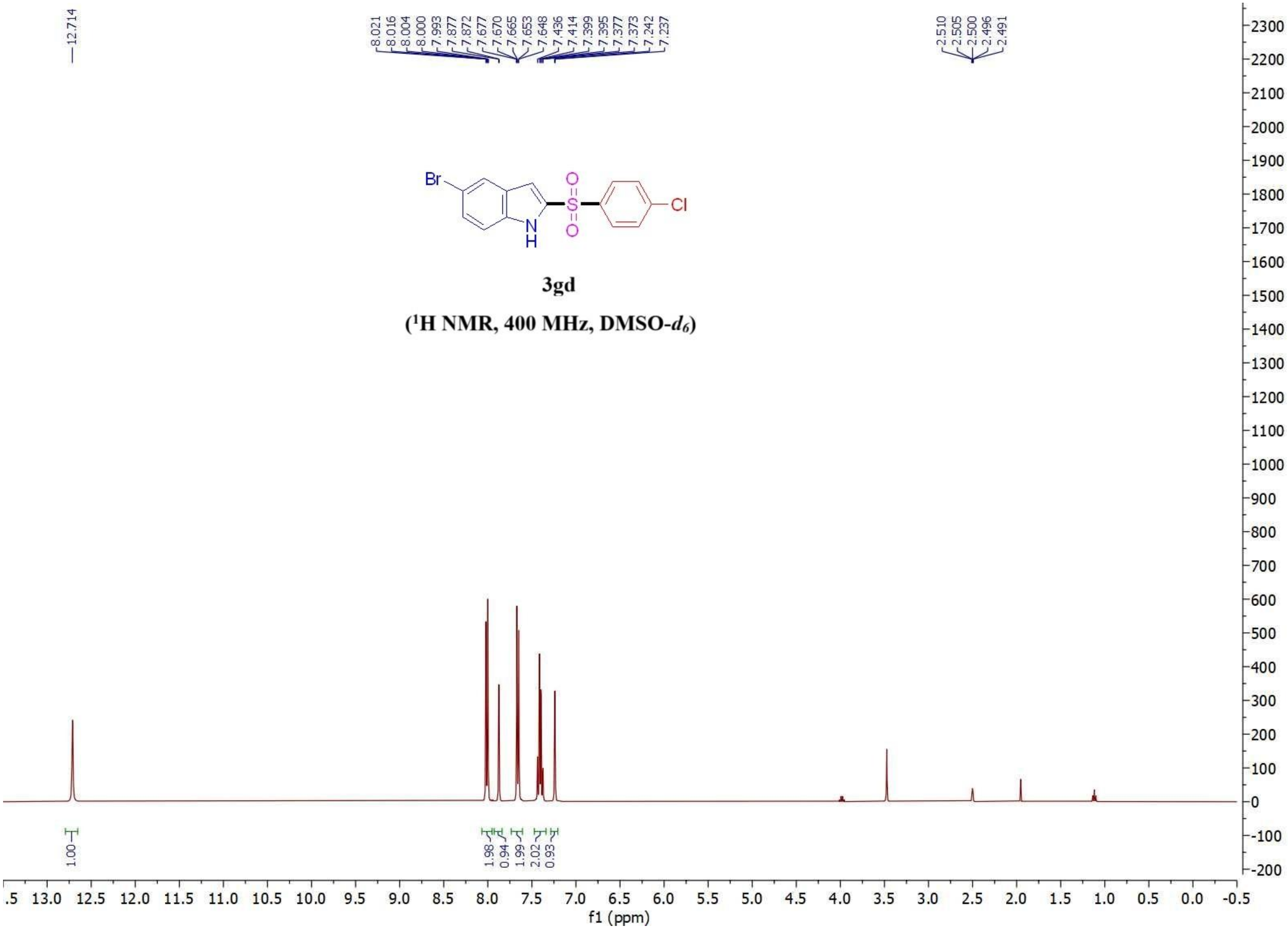


**3gd**

**($^{1}$H NMR, 400 MHz, DMSO-$d_6$)**

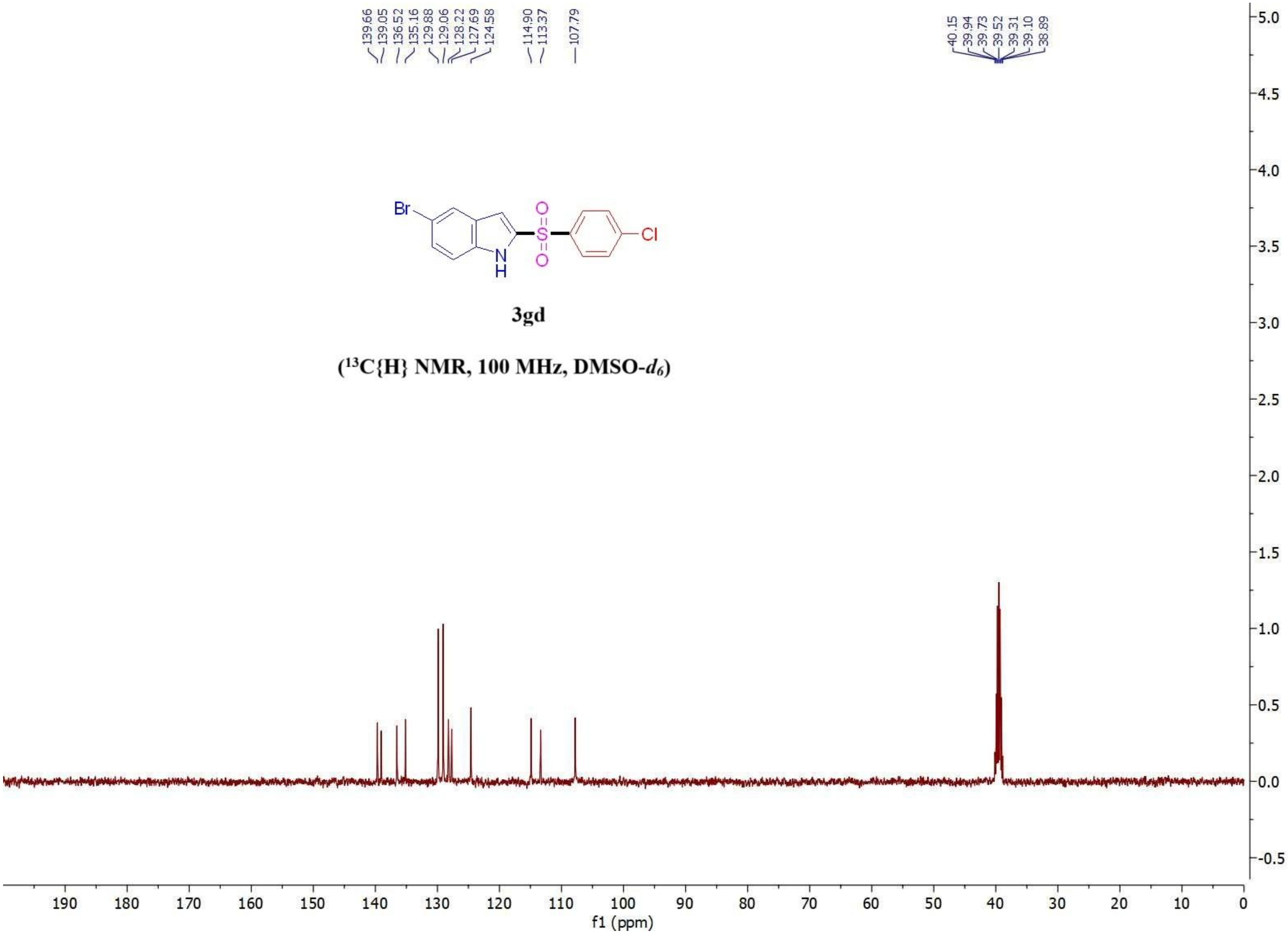


**3gd**

**($^{13}$C{H} NMR, 100 MHz, DMSO-$d_6$)**

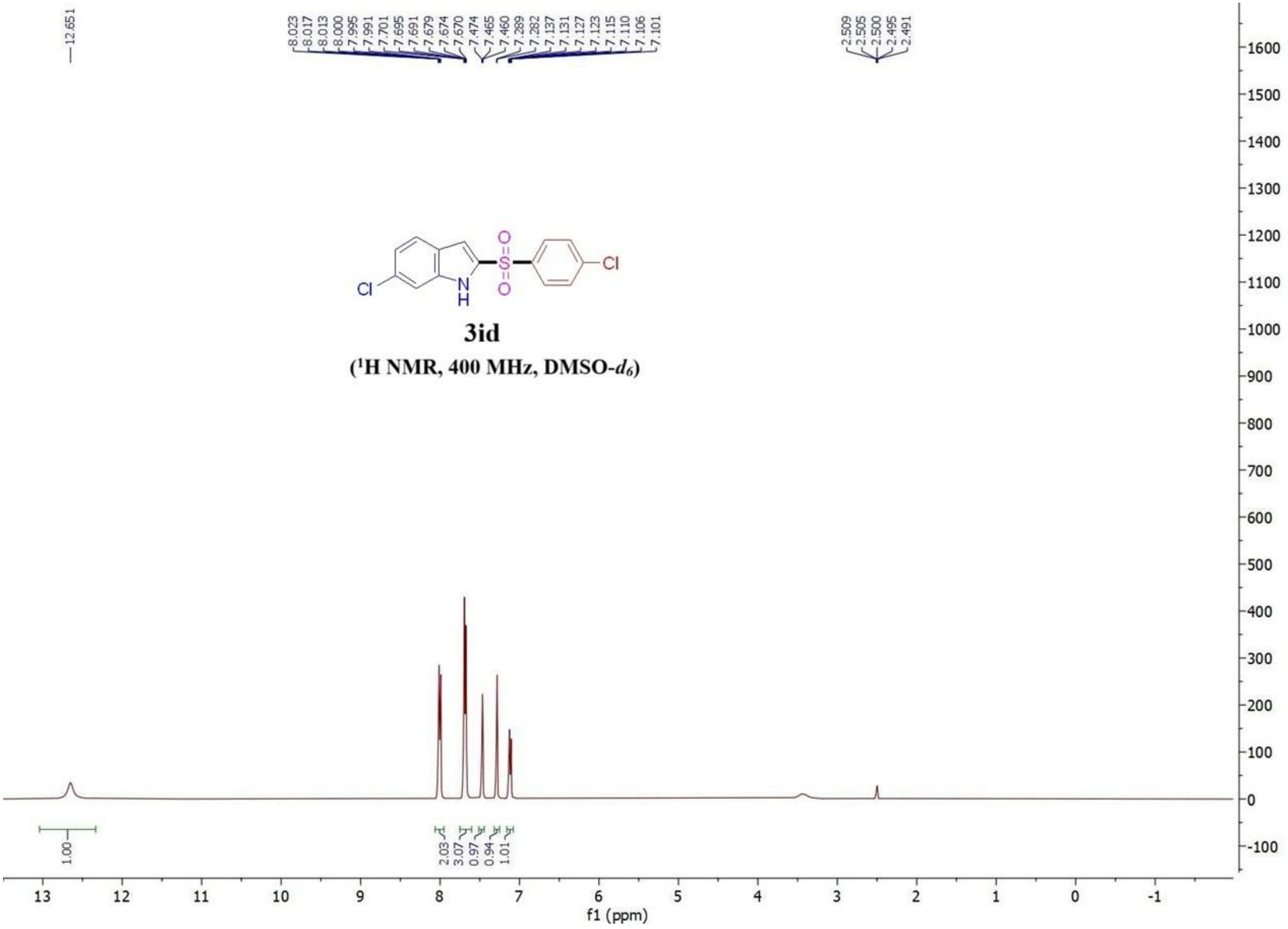

12.651
8.023
8.017
8.013
8.000
7.995
7.991
7.701
7.695
7.691
7.679
7.674
7.670
7.474
7.465
7.460
7.289
7.282
7.137
7.131
7.127
7.123
7.115
7.110
7.106
7.101
2.509
2.505
2.500
2.495
2.491
3id
(¹H NMR, 400 MHz, DMSO-d6)
1.00
2.03
3.07
0.97
0.94
1.01
f1 (ppm)

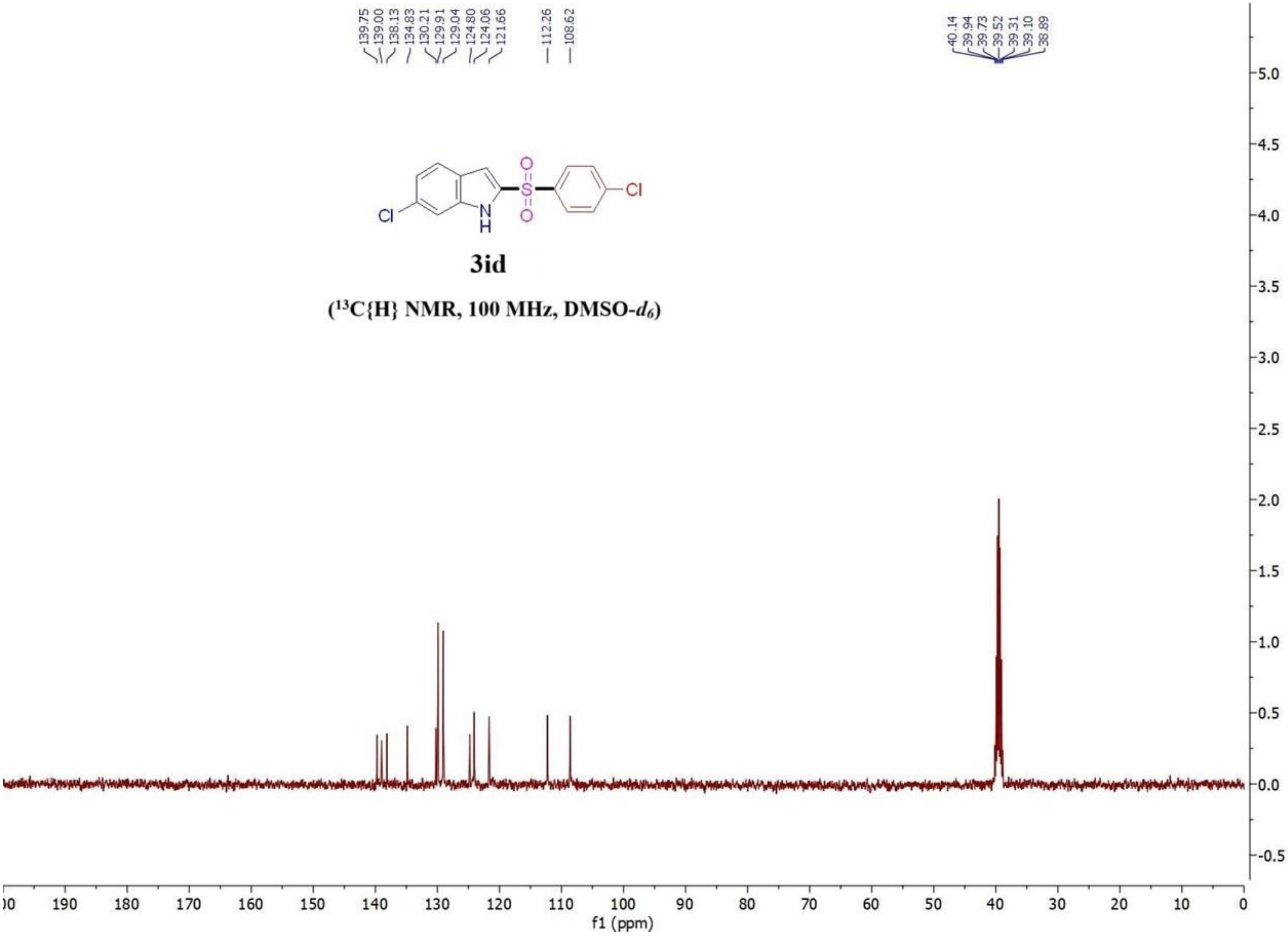


**3id**

**($^{13}$C{H} NMR, 100 MHz, DMSO-$d_6$)**

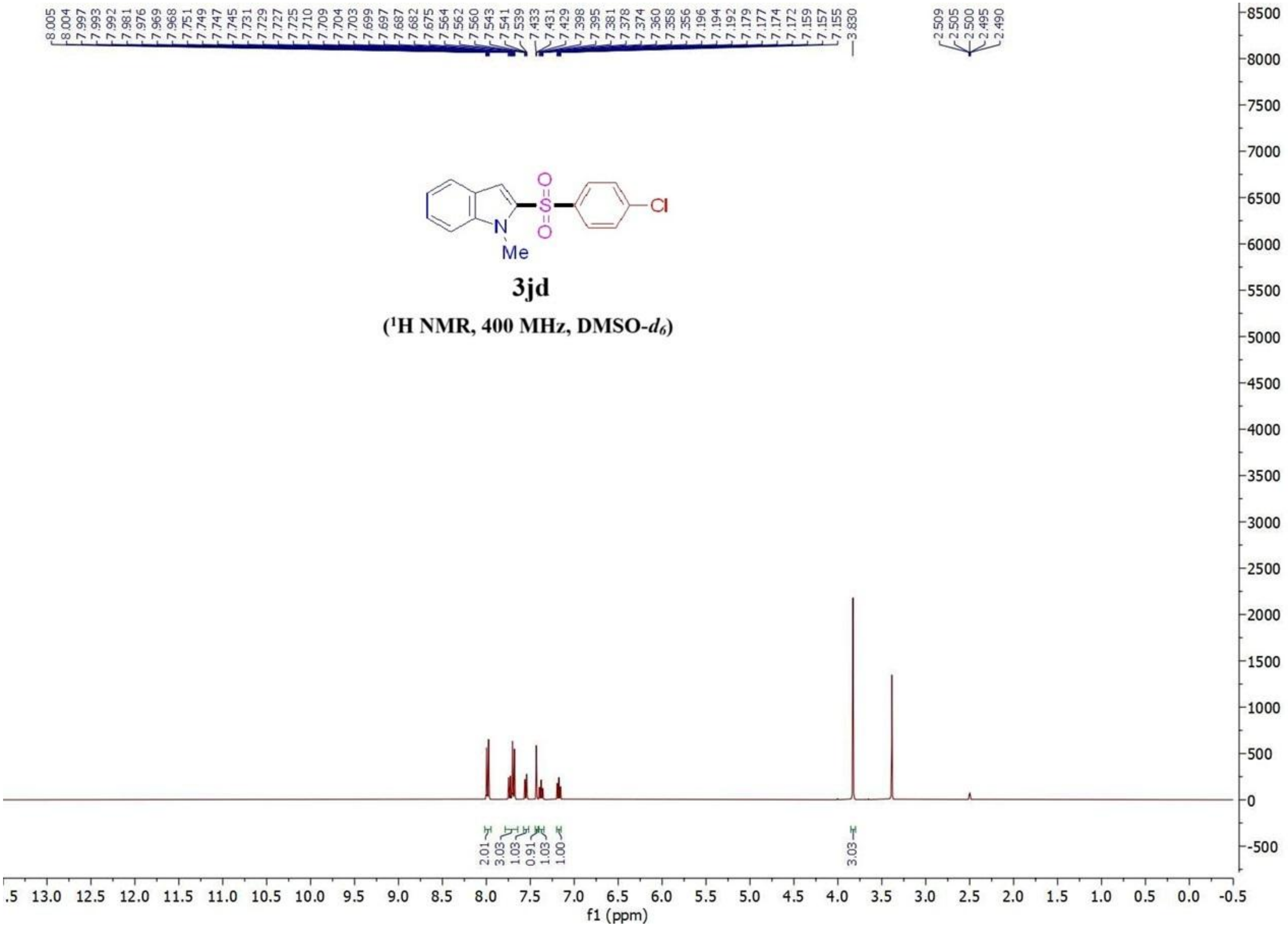

**3jd**

**($^{1}$H NMR, 400 MHz, DMSO-$d_6$)**

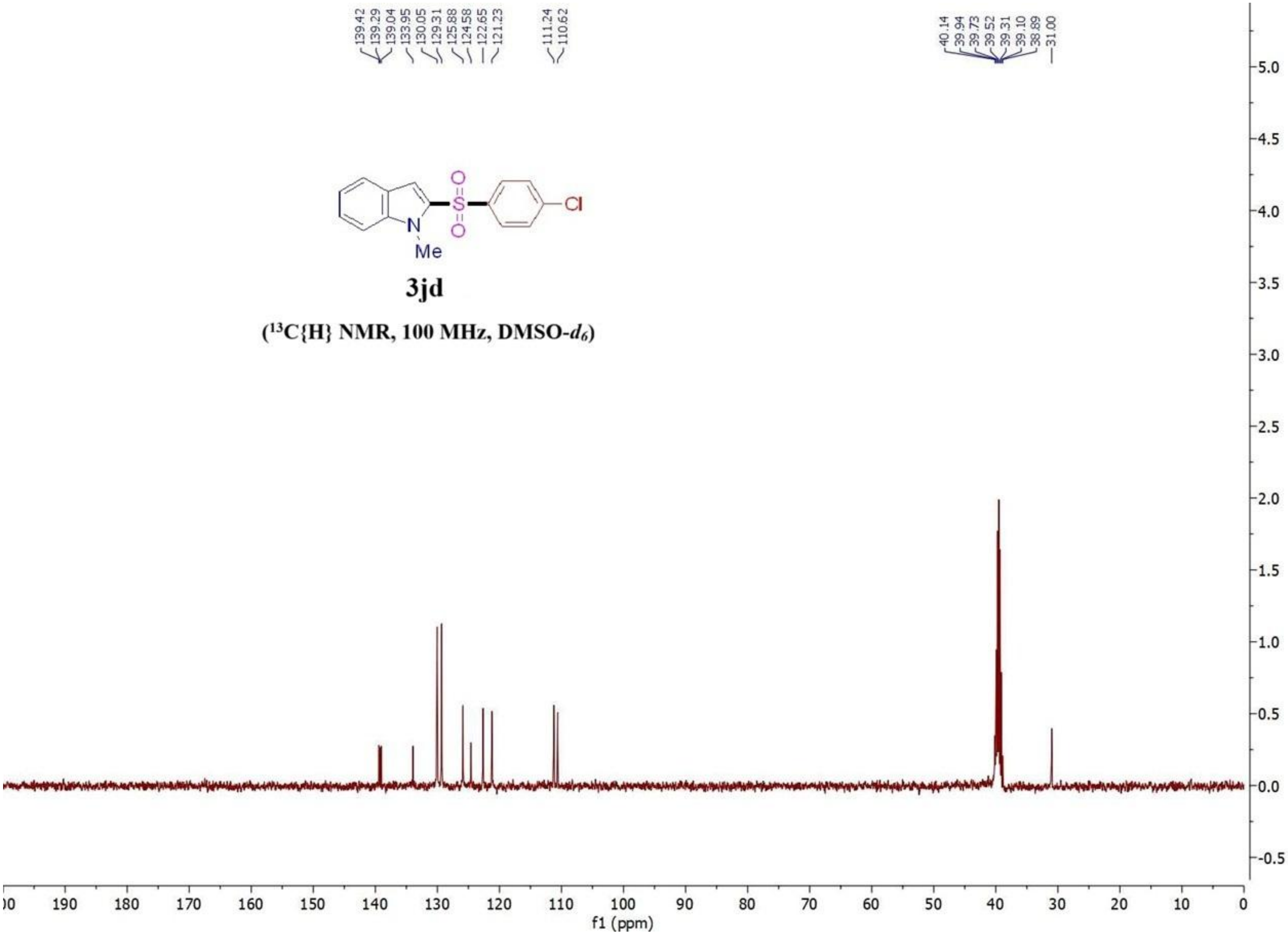


**3jd**

($^{13}C\{H\}$ NMR, 100 MHz, DMSO-$d_6$)

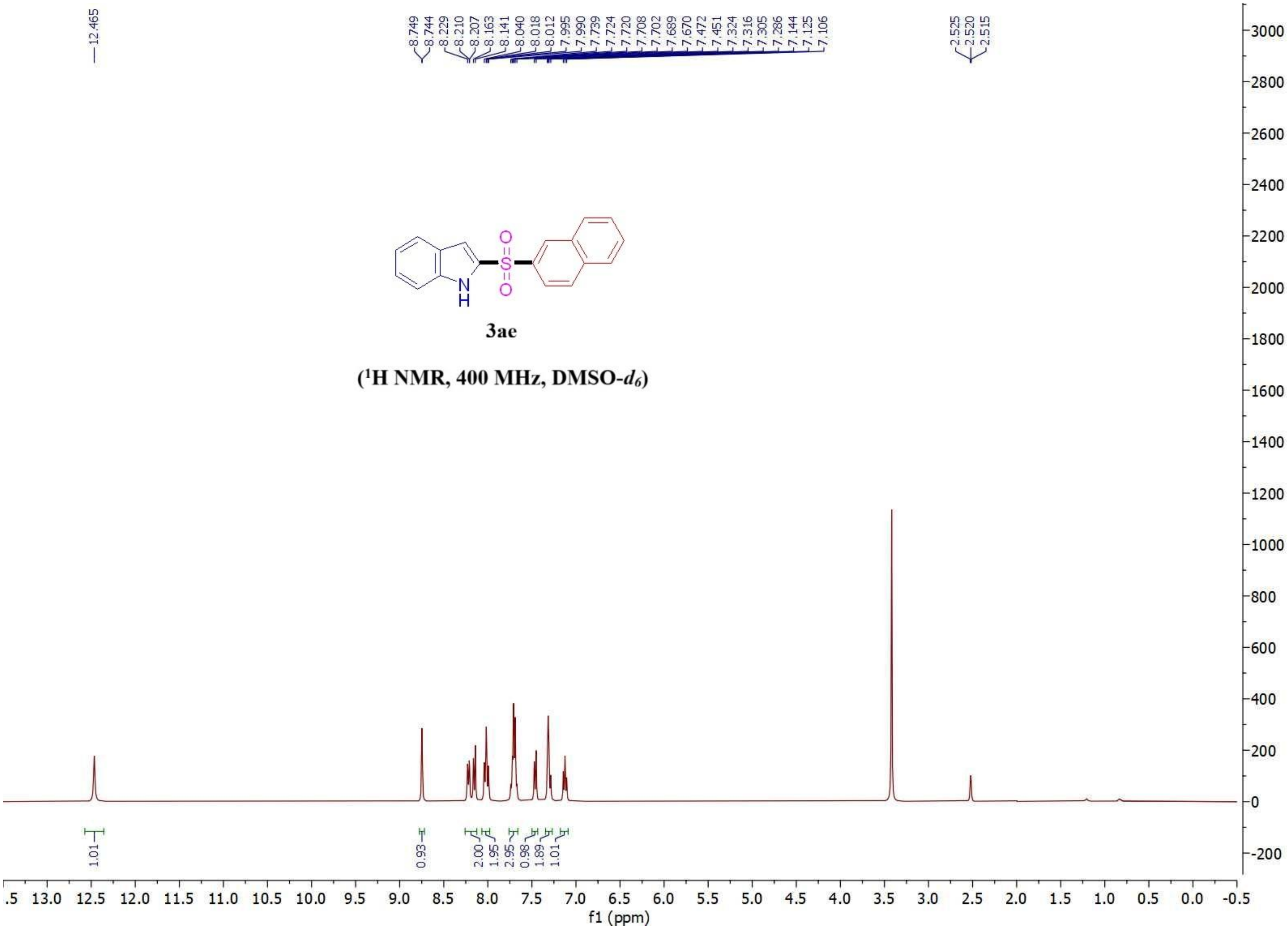
3ae
(¹H NMR, 400 MHz, DMSO-$d_6$)
f1 (ppm)

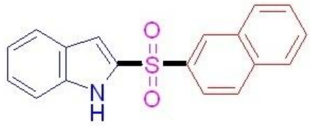

**3ae**

**($^{13}$C{H} NMR, 100 MHz, DMSO-$d_6$)**

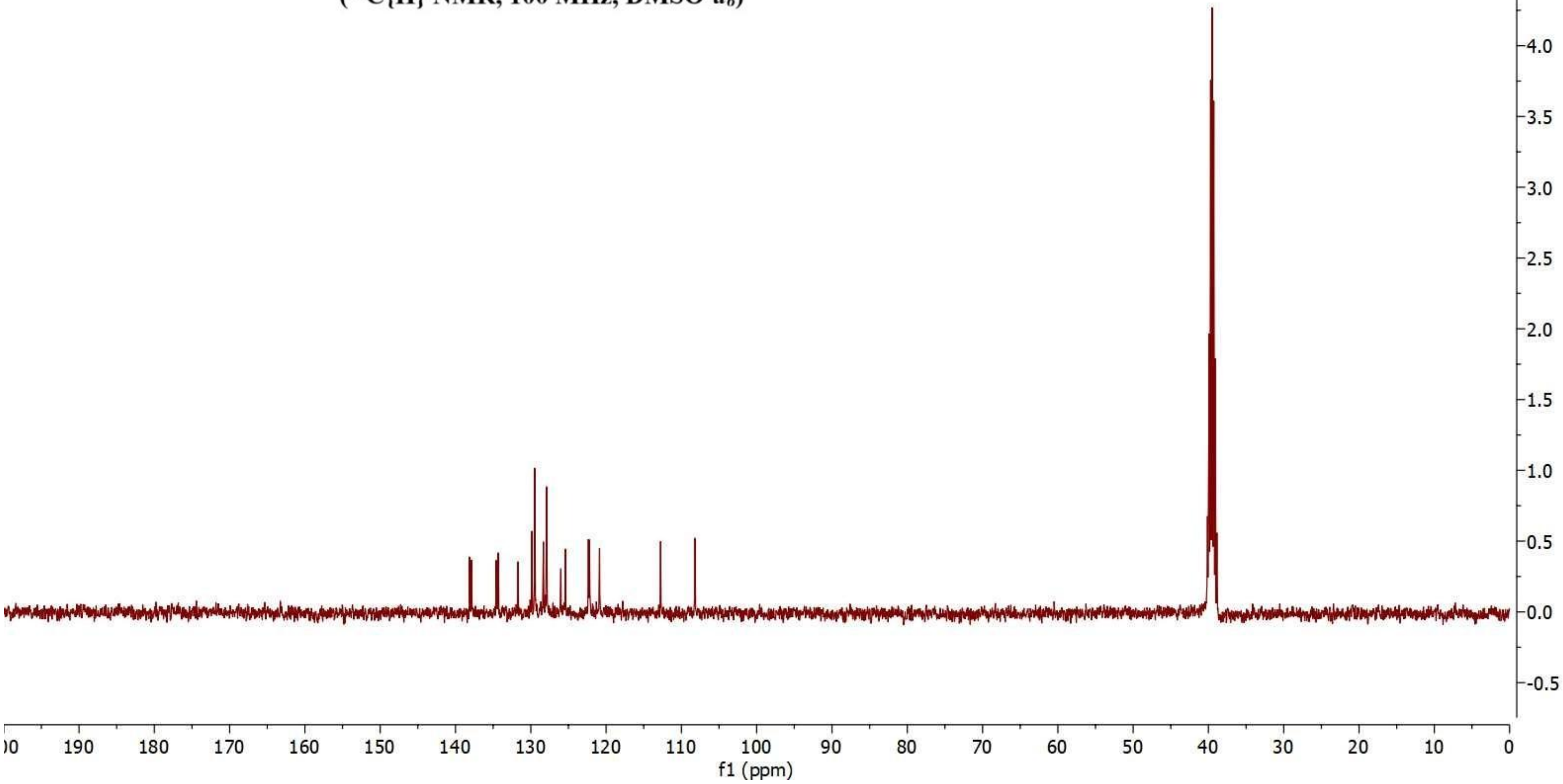

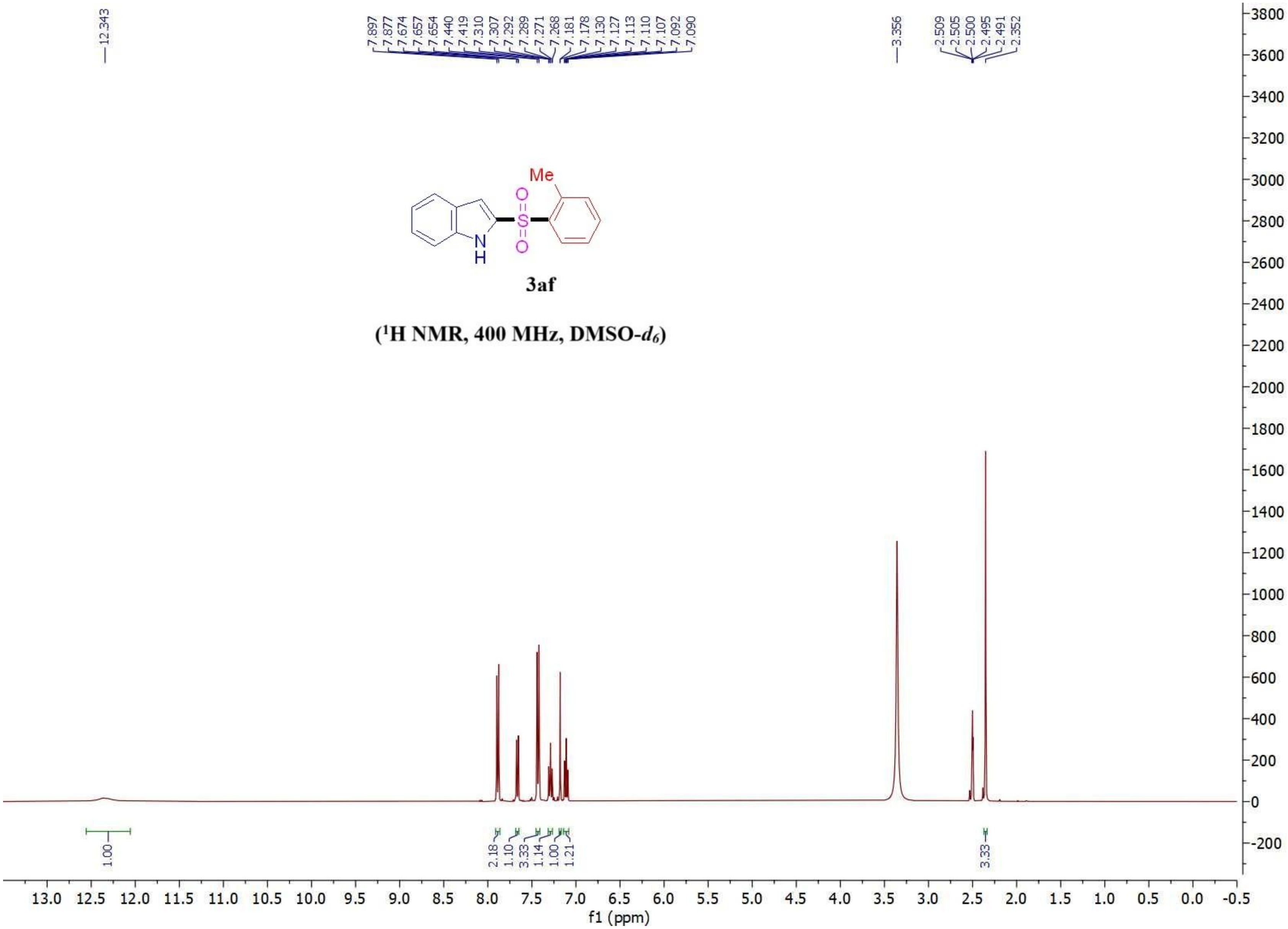

3af
(1H NMR, 400 MHz, DMSO-d6)

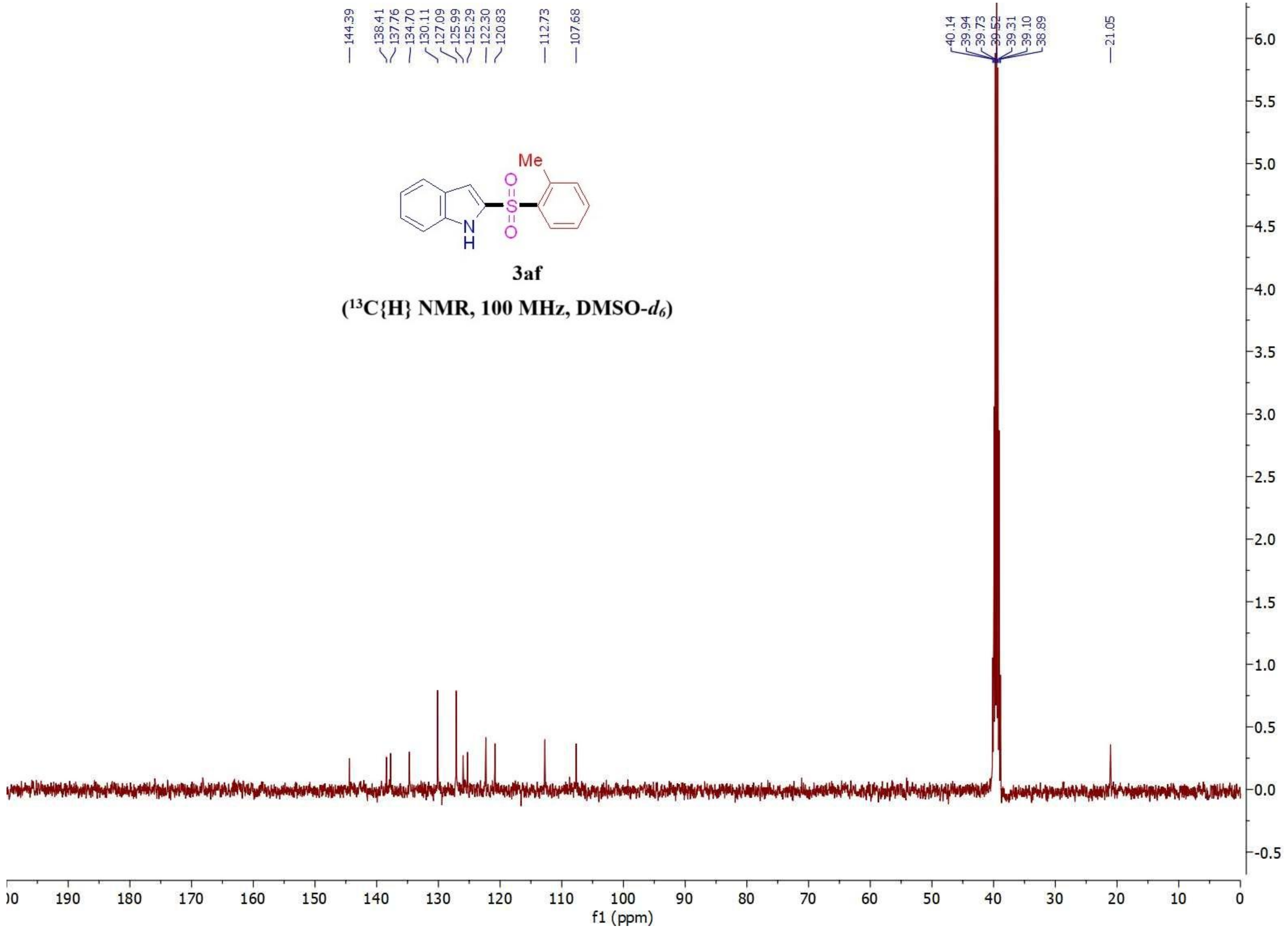


**3af**

**($^{13}$C{H} NMR, 100 MHz, DMSO-$d_6$)**

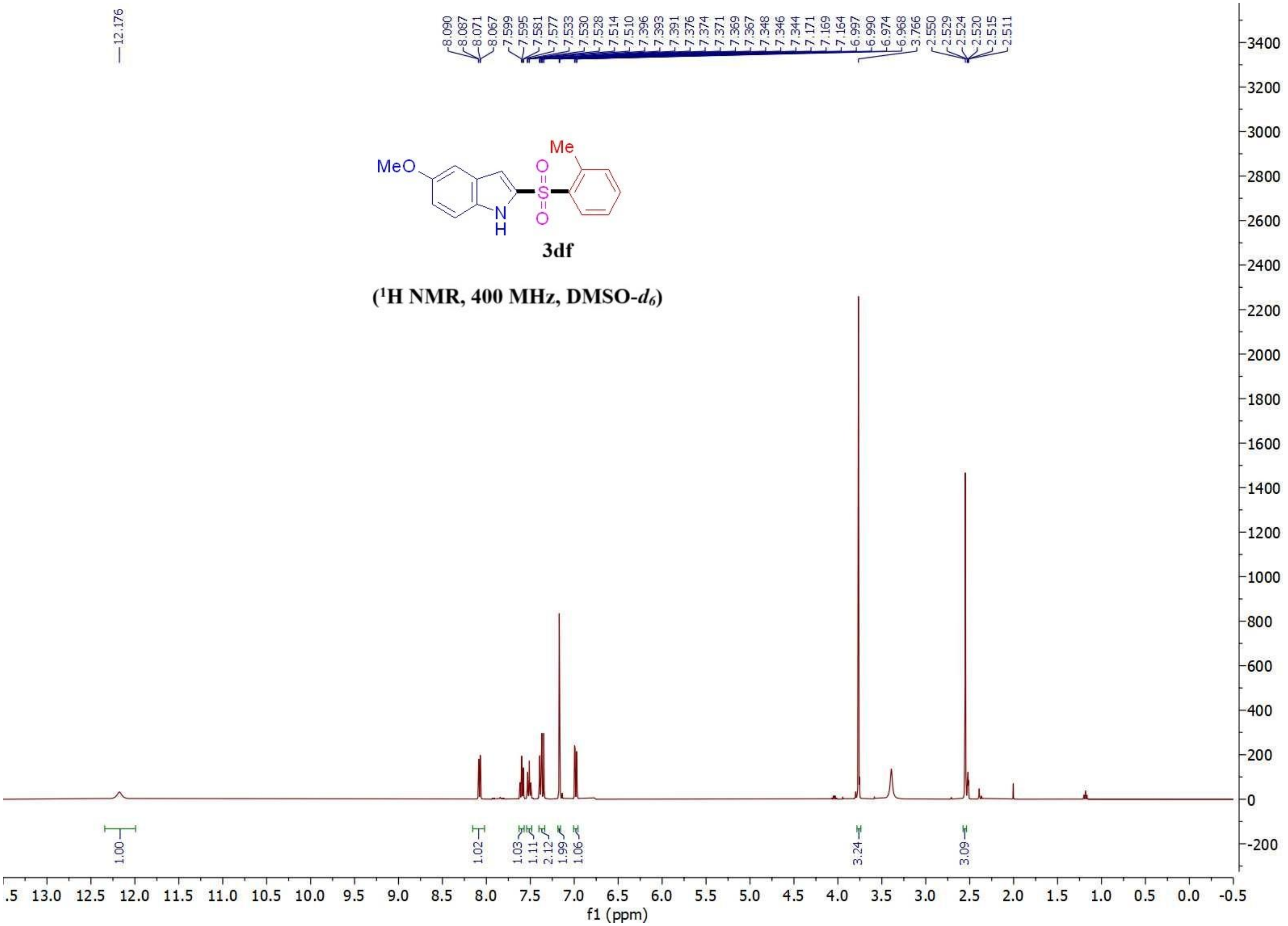


**3df**

**($^1$H NMR, 400 MHz, DMSO-$d_6$)**

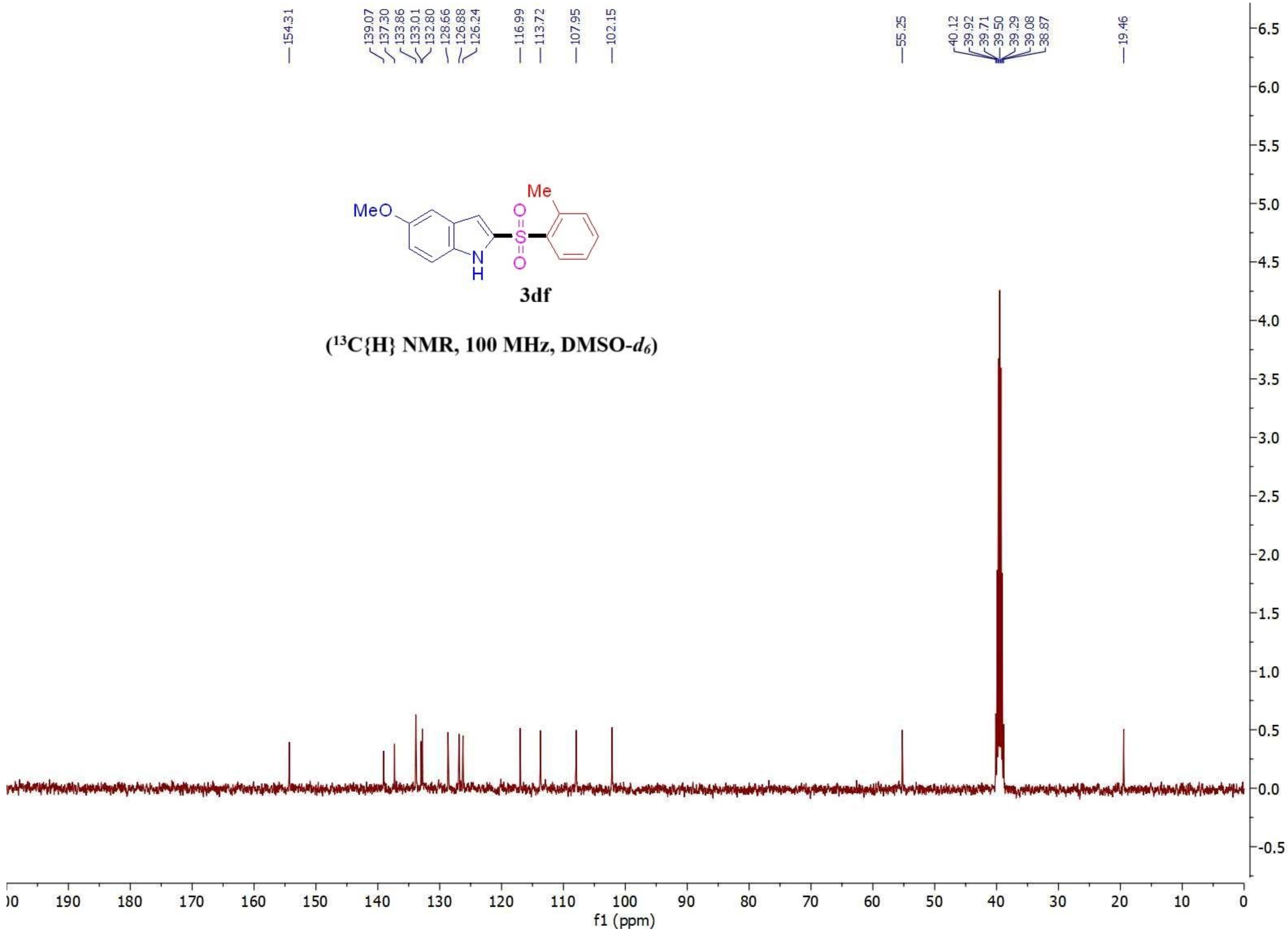


**3df**

($^{13}$C{H} NMR, 100 MHz, DMSO-$d_6$)

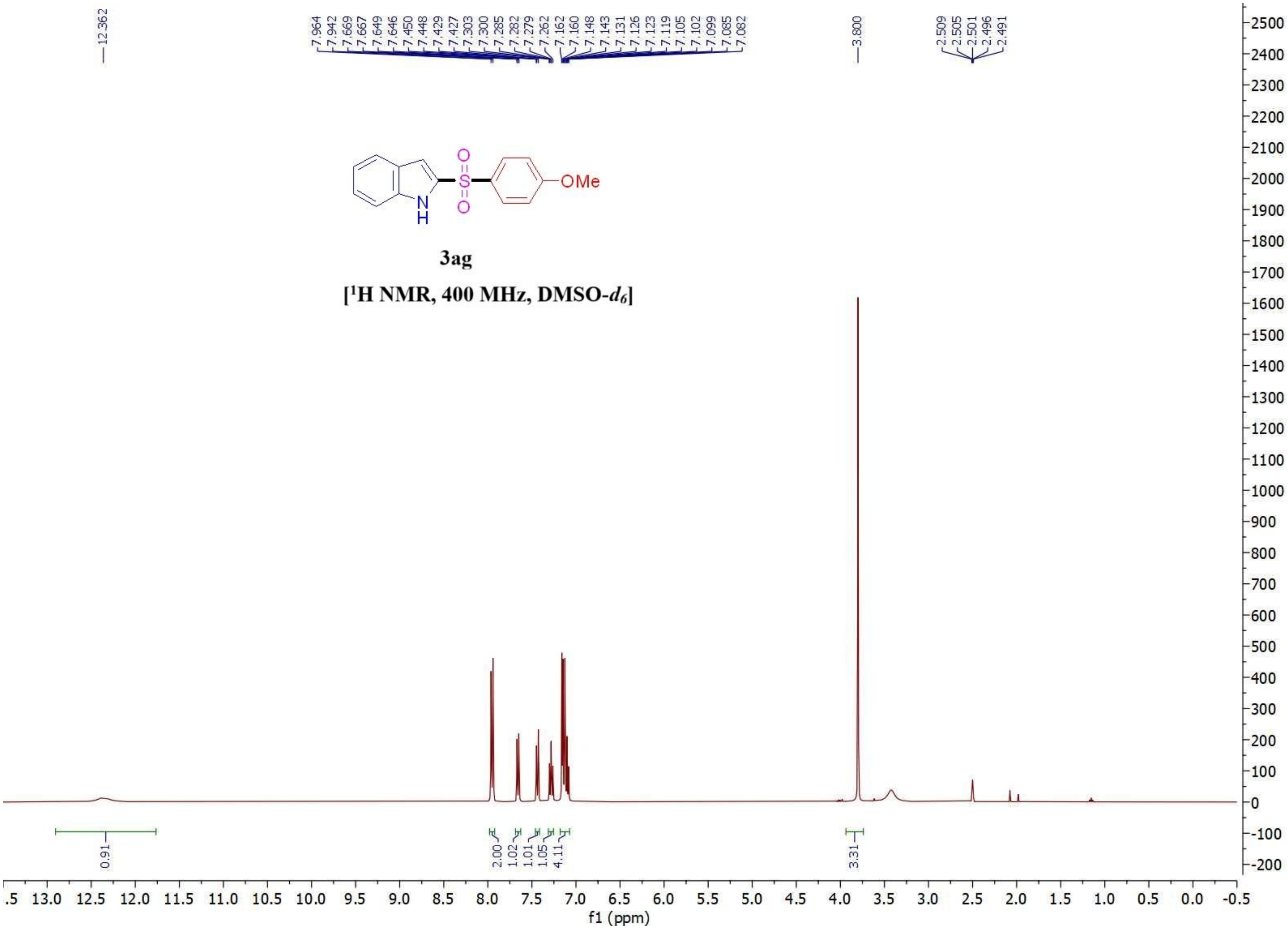
12.362
7.964 7.942 7.669 7.667 7.649 7.646 7.450 7.448 7.429 7.427 7.303 7.300 7.285 7.282 7.279 7.262 7.162 7.160 7.148 7.143 7.131 7.126 7.123 7.119 7.105 7.102 7.099 7.085 7.082
3.800
2.509 2.505 2.501 2.496 2.491
O
S
O
N
H
OMe
3ag
[$^{1}$H NMR, 400 MHz, DMSO-$d_6$]
0.91
2.00
1.02
1.01
1.05
4.11
3.31
f1 (ppm)

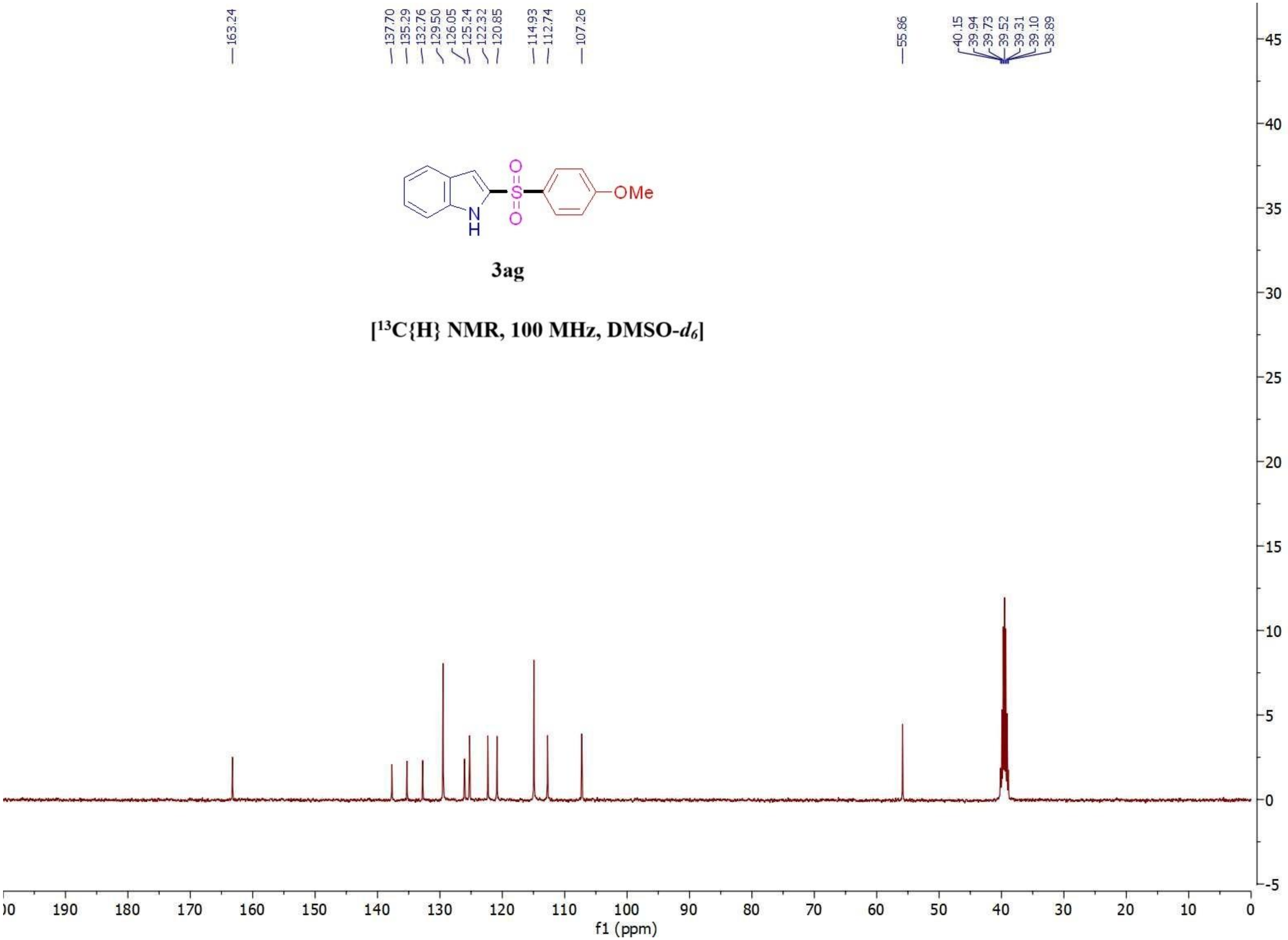


**3ag**

**[$^{13}$C{H} NMR, 100 MHz, DMSO-*$d_6$*]**

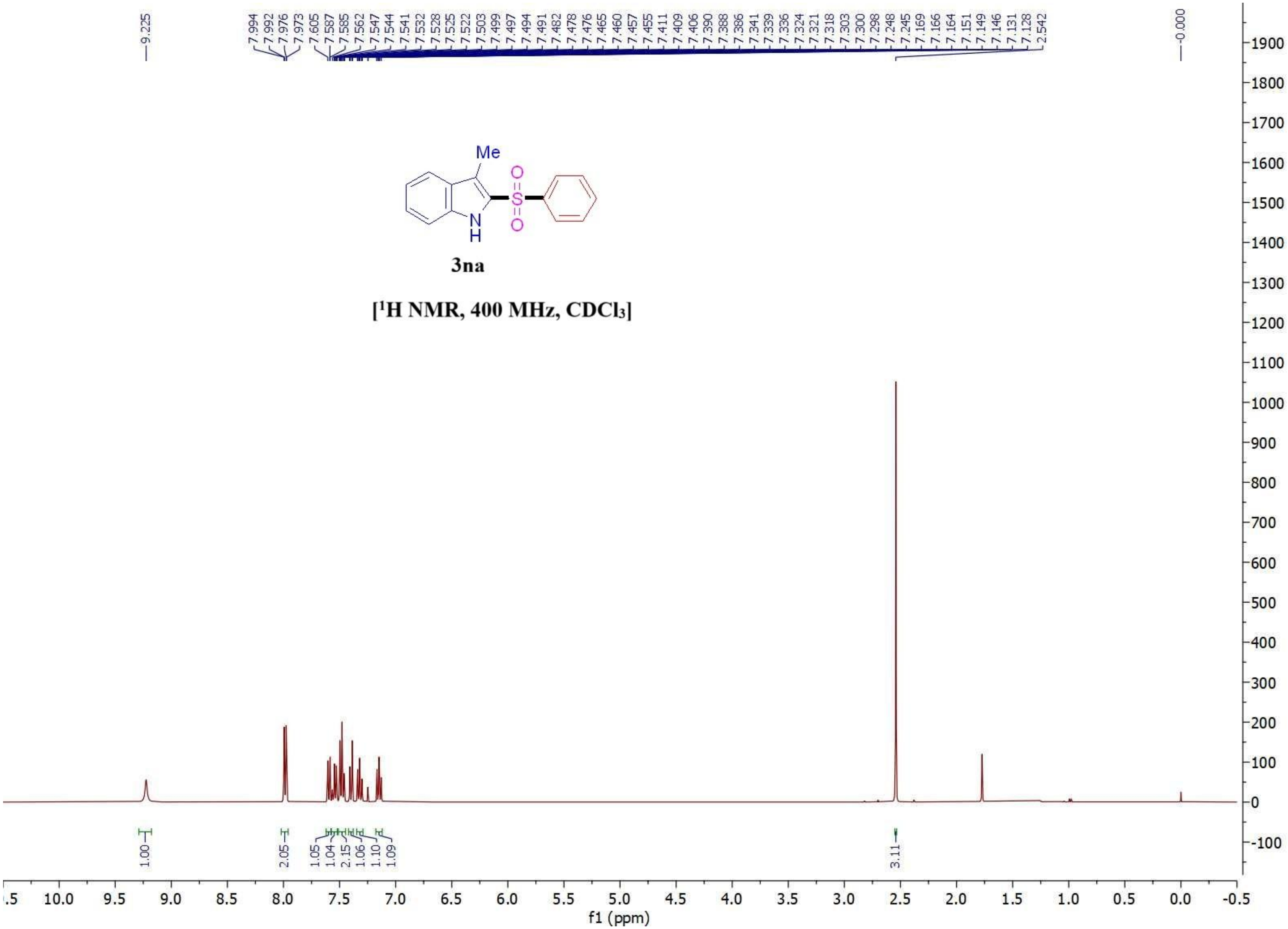


**3na**

**[$^{1}$H NMR, 400 MHz, CDCl$_3$]**

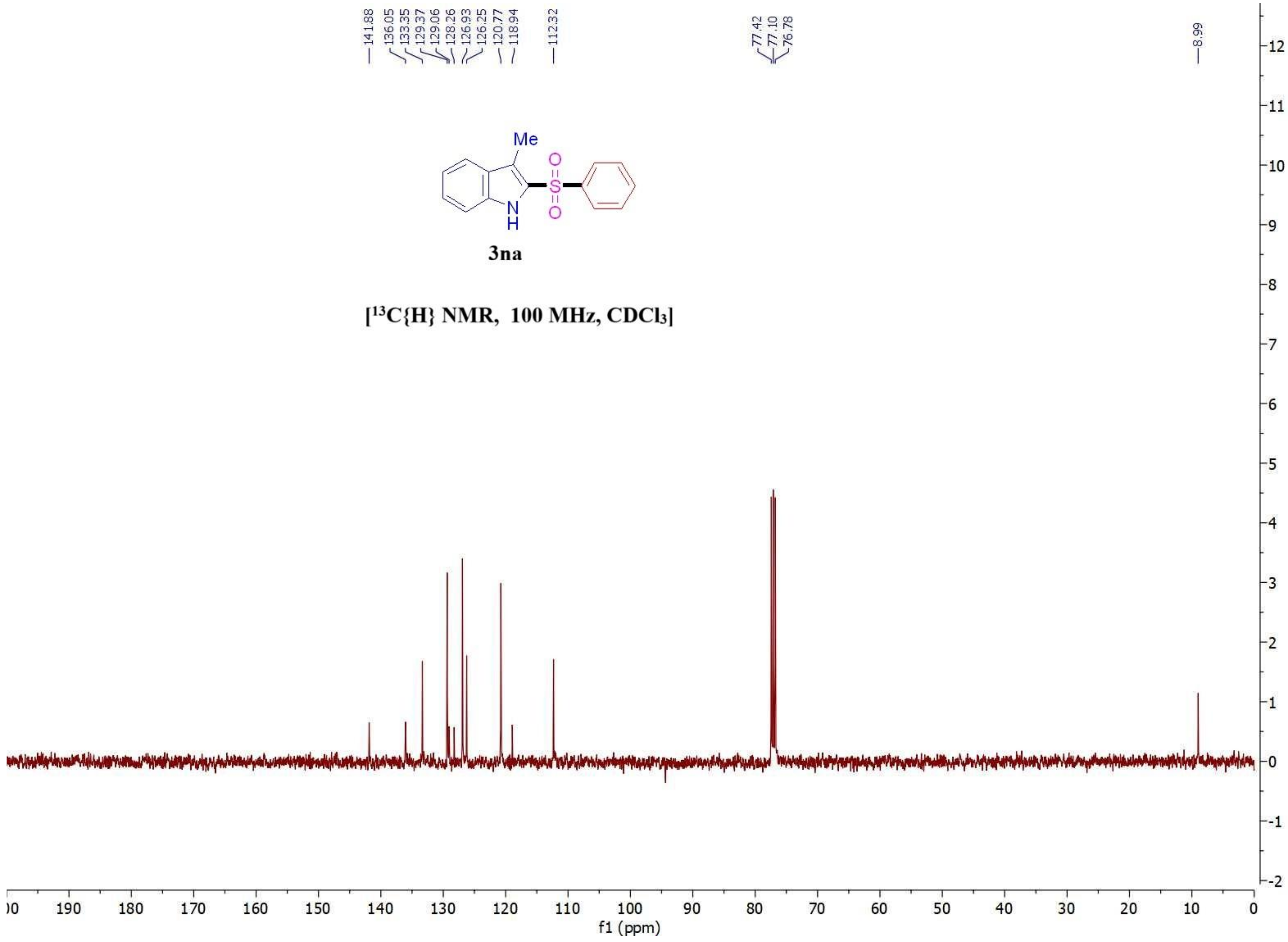


**3na**

[$^{13}C\{H\}$ NMR, 100 MHz, $CDCl_3$]

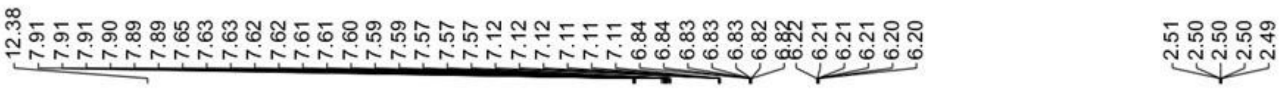


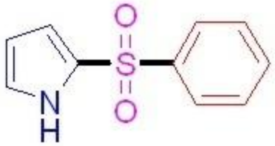


**5aa**

**($^1$H NMR, 400 MHz, DMSO-$d_6$)**

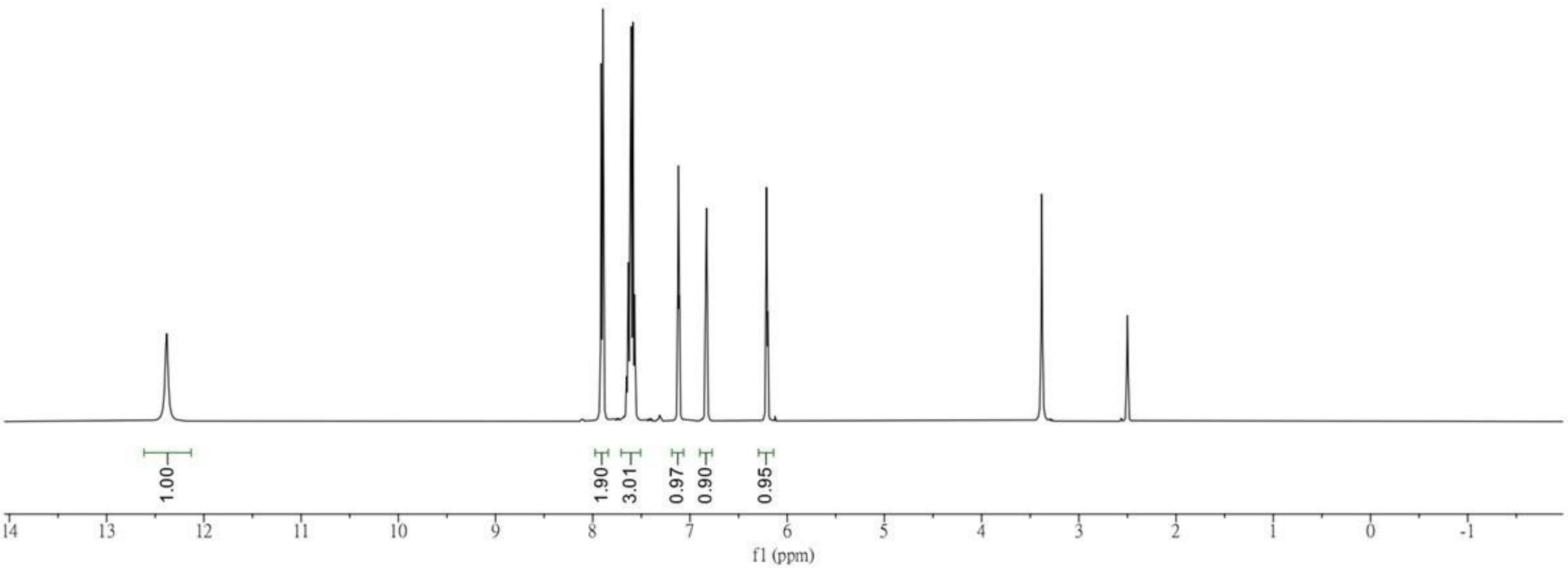

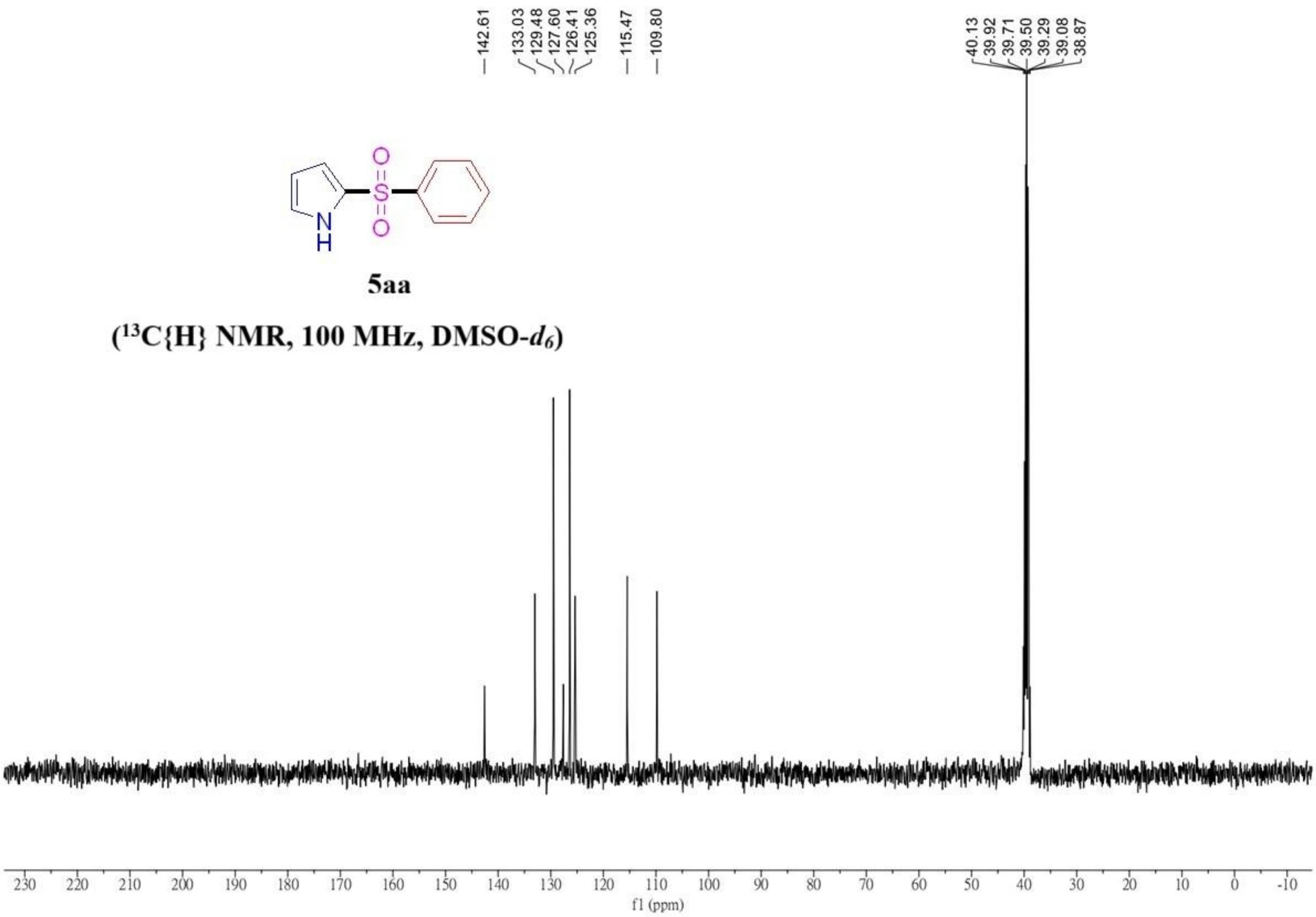
142.61
133.03
129.48
127.60
126.41
125.36
115.47
109.80
40.13
39.92
39.71
39.50
39.29
39.08
38.87
N
H
O
S
O
5aa
(13C{H} NMR, 100 MHz, DMSO-d6)
230 220 210 200 190 180 170 160 150 140 130 120 110 100 90 80 70 60 50 40 30 20 10 0 -10
f1 (ppm)

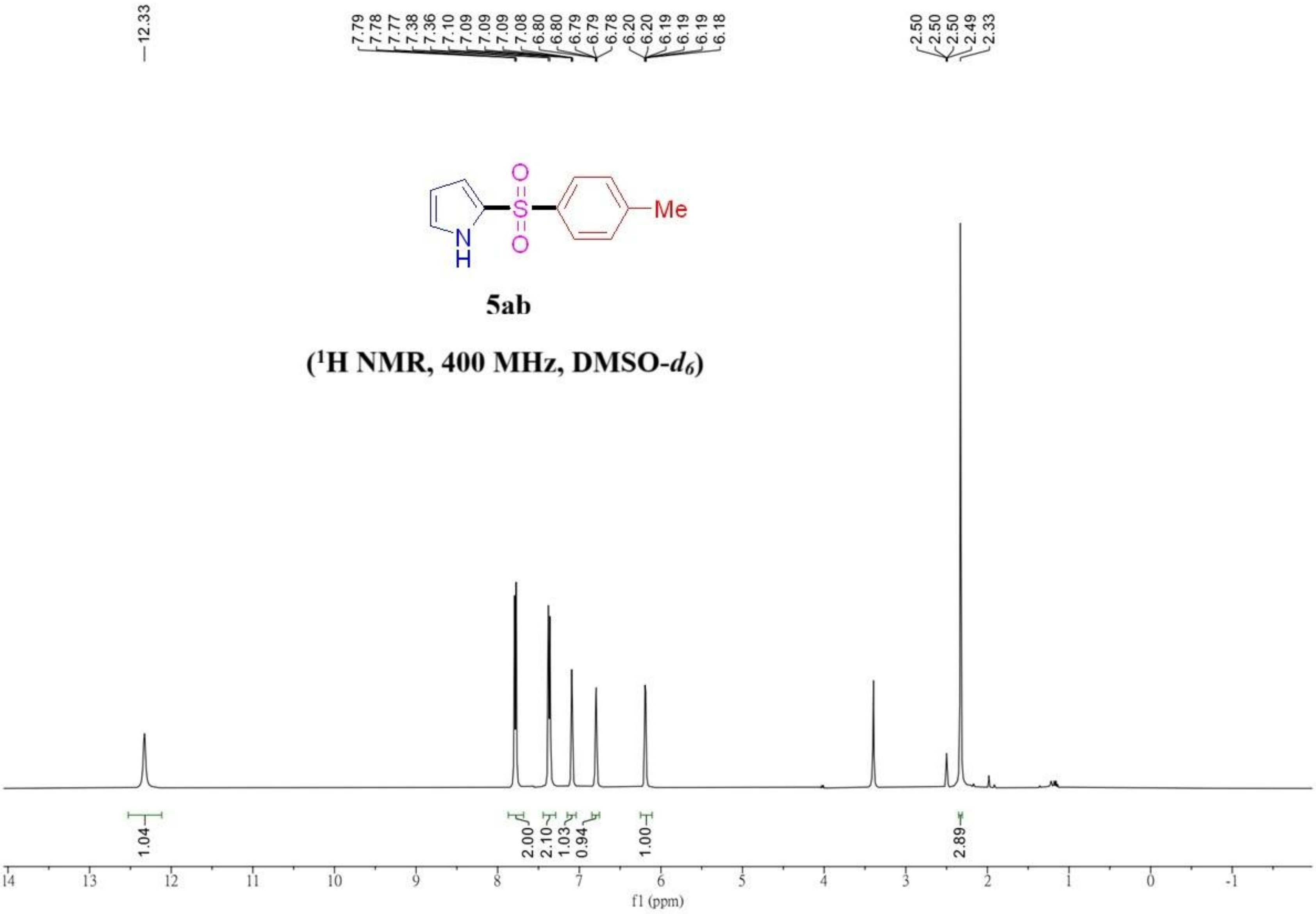

12.33
7.79
7.78
7.77
7.38
7.36
7.10
7.09
7.09
7.09
7.08
6.80
6.80
6.79
6.79
6.78
6.20
6.20
6.19
6.19
6.19
6.18
2.50
2.50
2.50
2.49
2.33
Me
5ab
(¹H NMR, 400 MHz, DMSO-d6)
1.04
2.00
2.10
1.03
0.94
1.00
2.89
f1 (ppm)

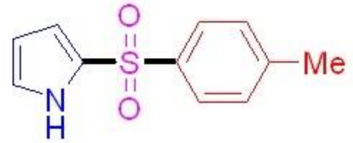

**5ab**

**($^{13}$C{H} NMR, 100 MHz, DMSO-$d_6$)**

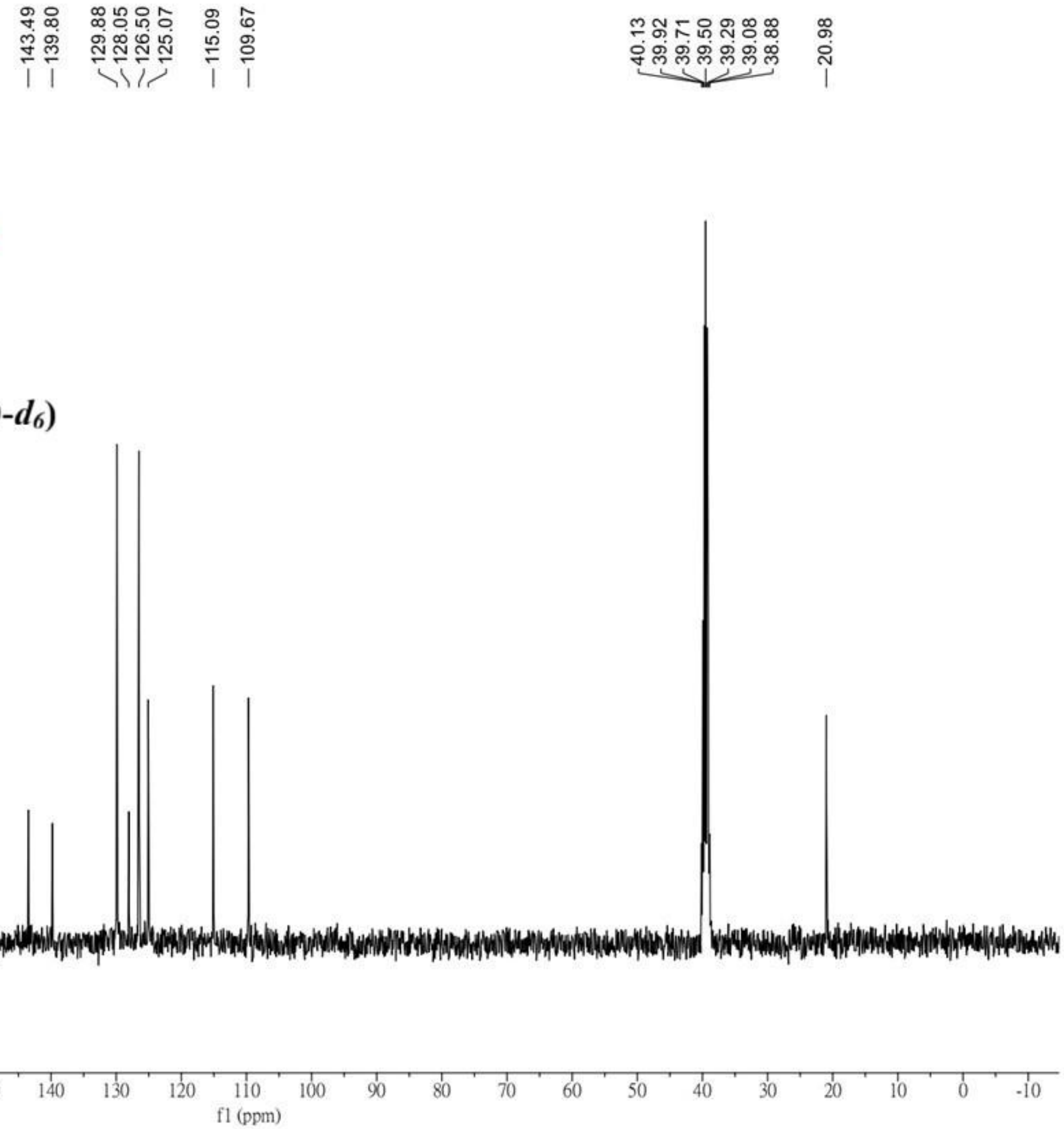

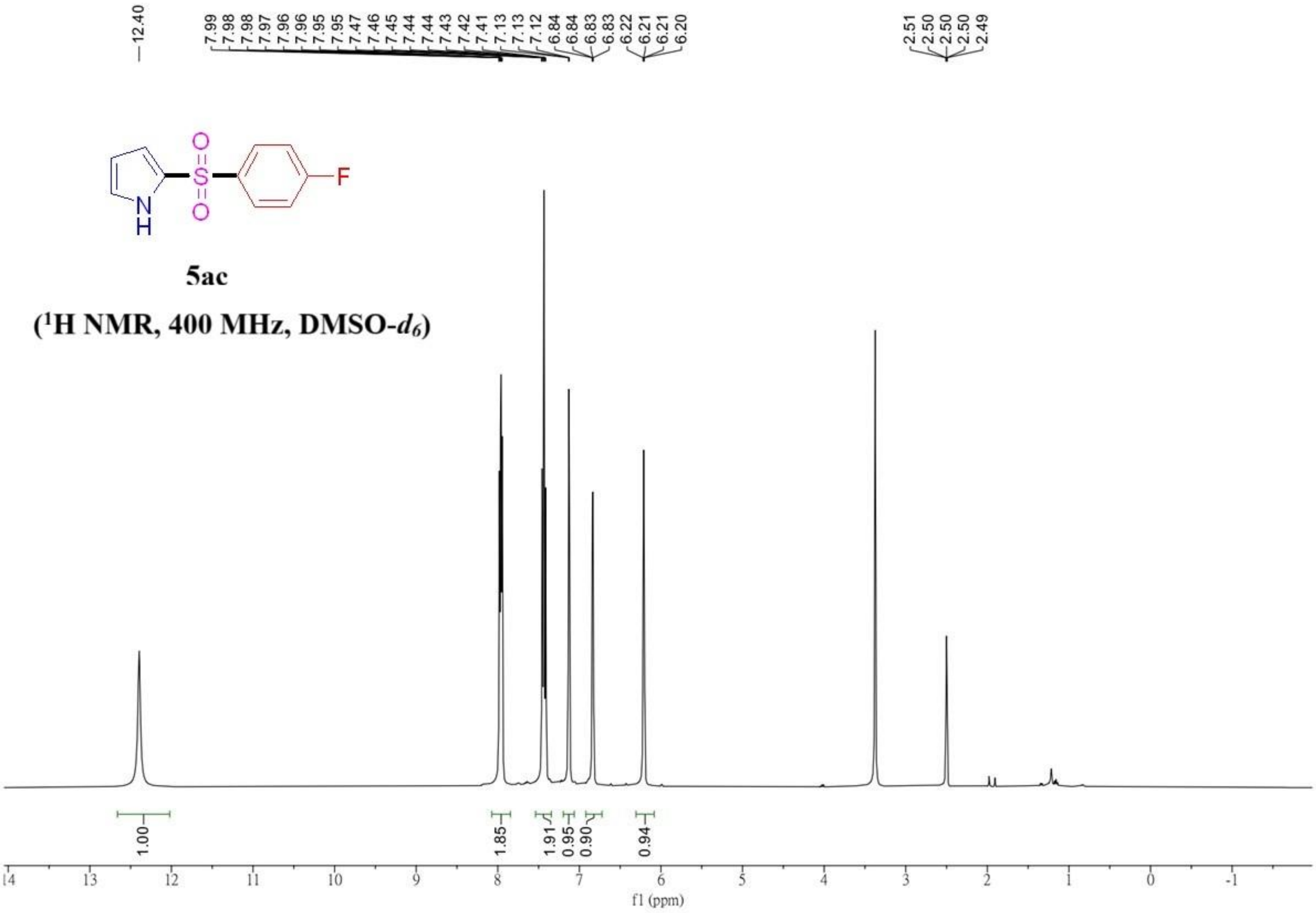


**5ac**

**($^{1}$H NMR, 400 MHz, DMSO-$d_6$)**

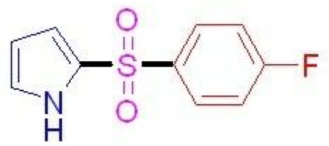


**5ac**

**($^{13}C\{H\}$ NMR, 100 MHz, DMSO-$d_6$)**

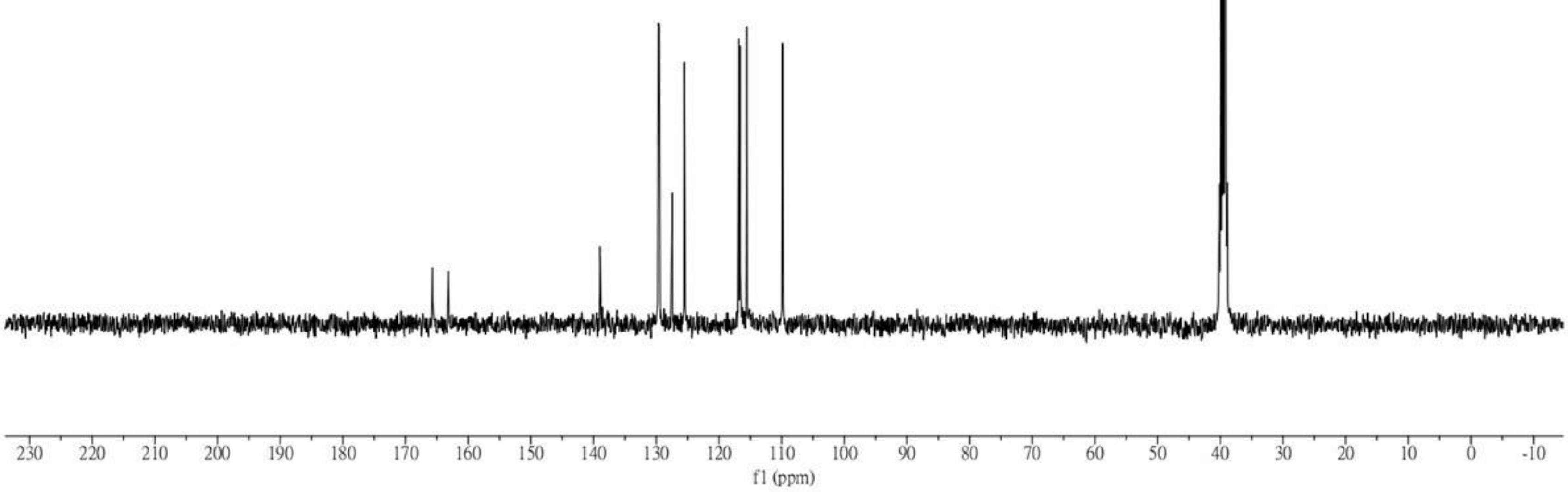

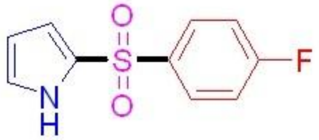

**5ac**

**($^{19}$F NMR, 376 MHz, DMSO-$d_6$)**

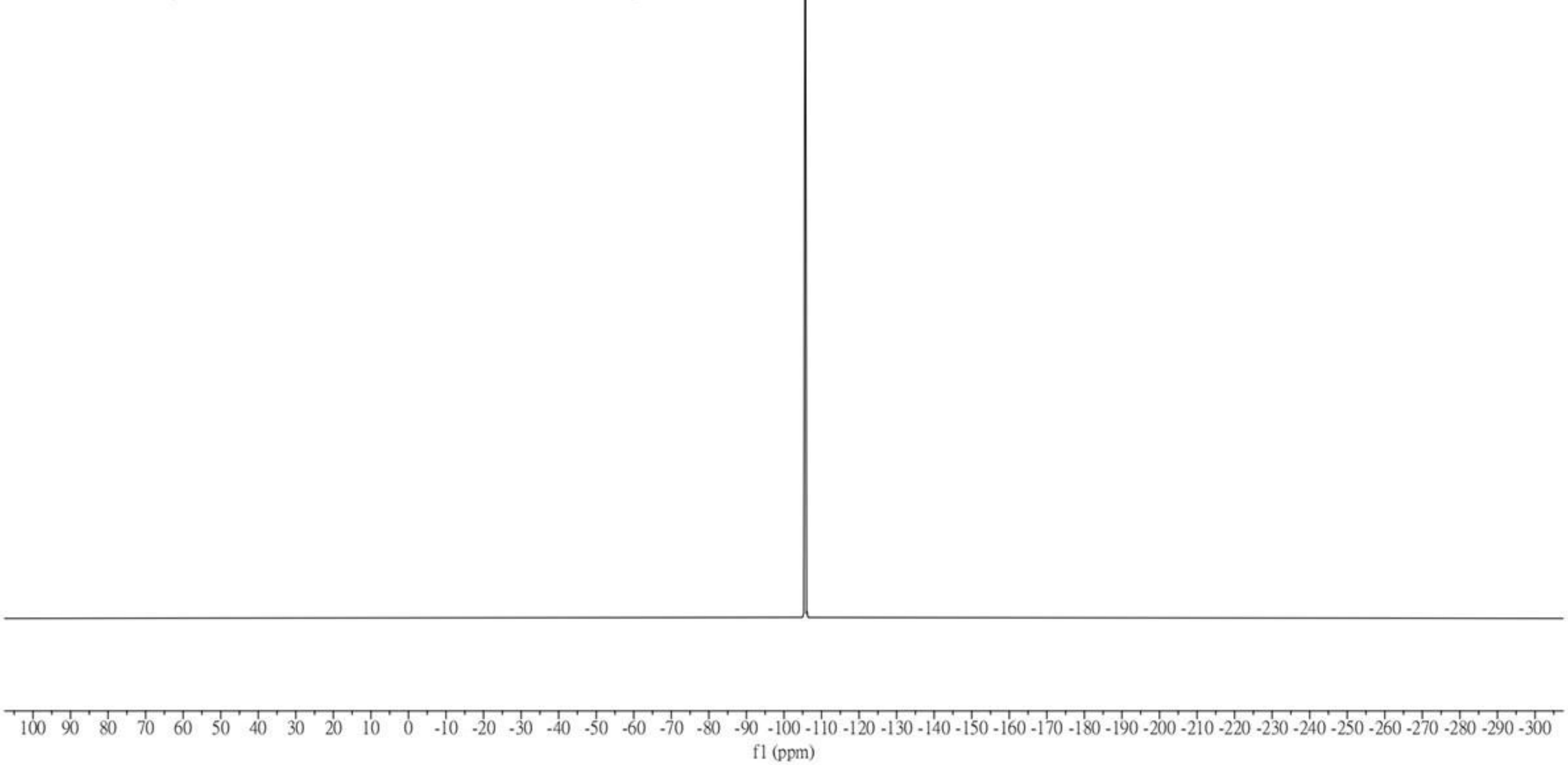

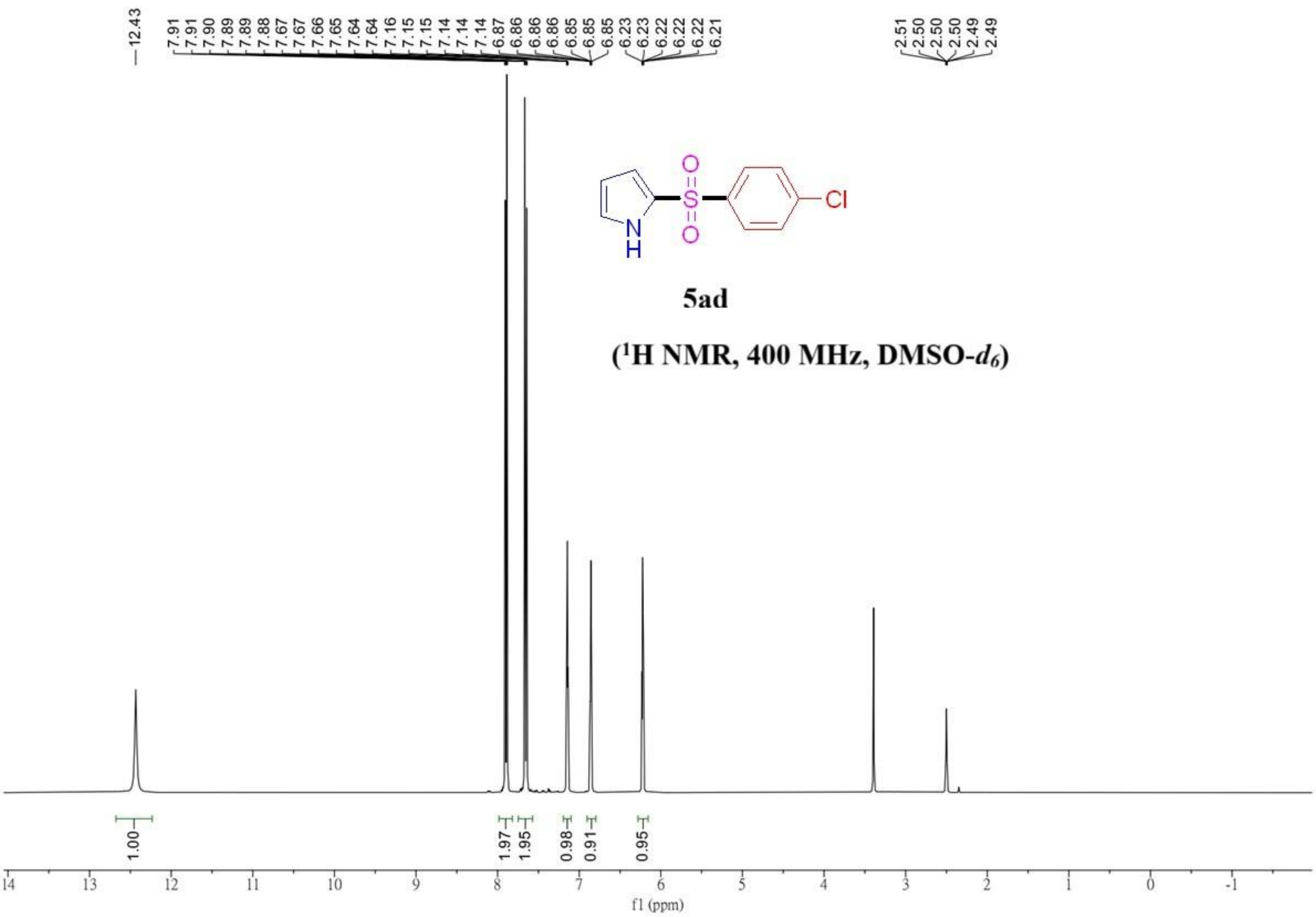
12.43
7.91 7.91 7.90 7.89 7.89 7.88 7.67 7.67 7.66 7.65 7.64 7.64 7.16 7.15 7.15 7.14 7.14 7.14 6.87 6.86 6.86 6.86 6.85 6.85 6.85 6.23 6.23 6.22 6.22 6.22 6.21
2.51 2.50 2.50 2.50 2.49 2.49
O
S
O
Cl
N
H
5ad
(¹H NMR, 400 MHz, DMSO-d6)
1.00
1.97
1.95
0.98
0.91
0.95
14 13 12 11 10 9 8 7 6 5 4 3 2 1 0 -1
f1 (ppm)

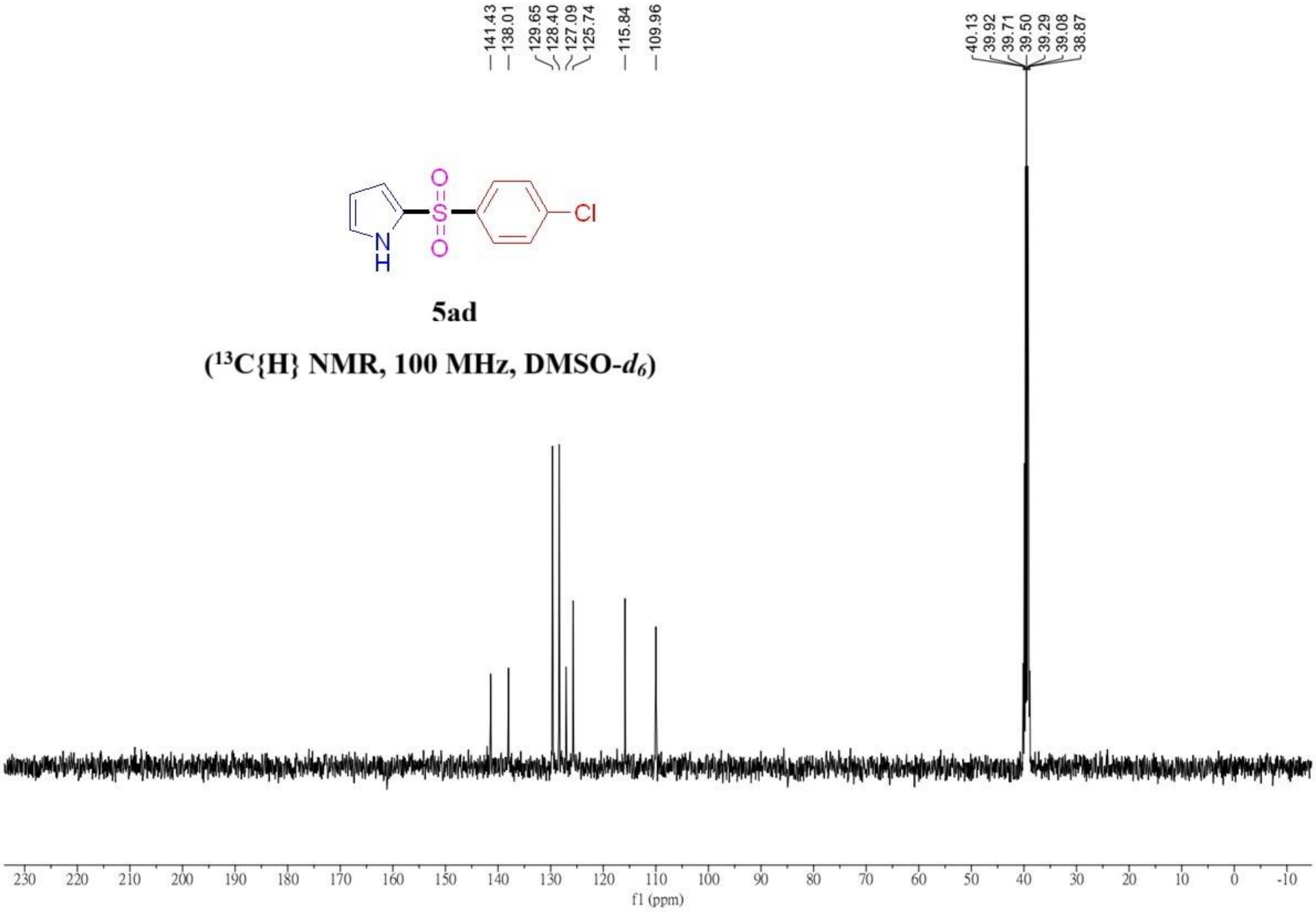


**5ad**

**($^{13}$C{H} NMR, 100 MHz, DMSO-$d_6$)**

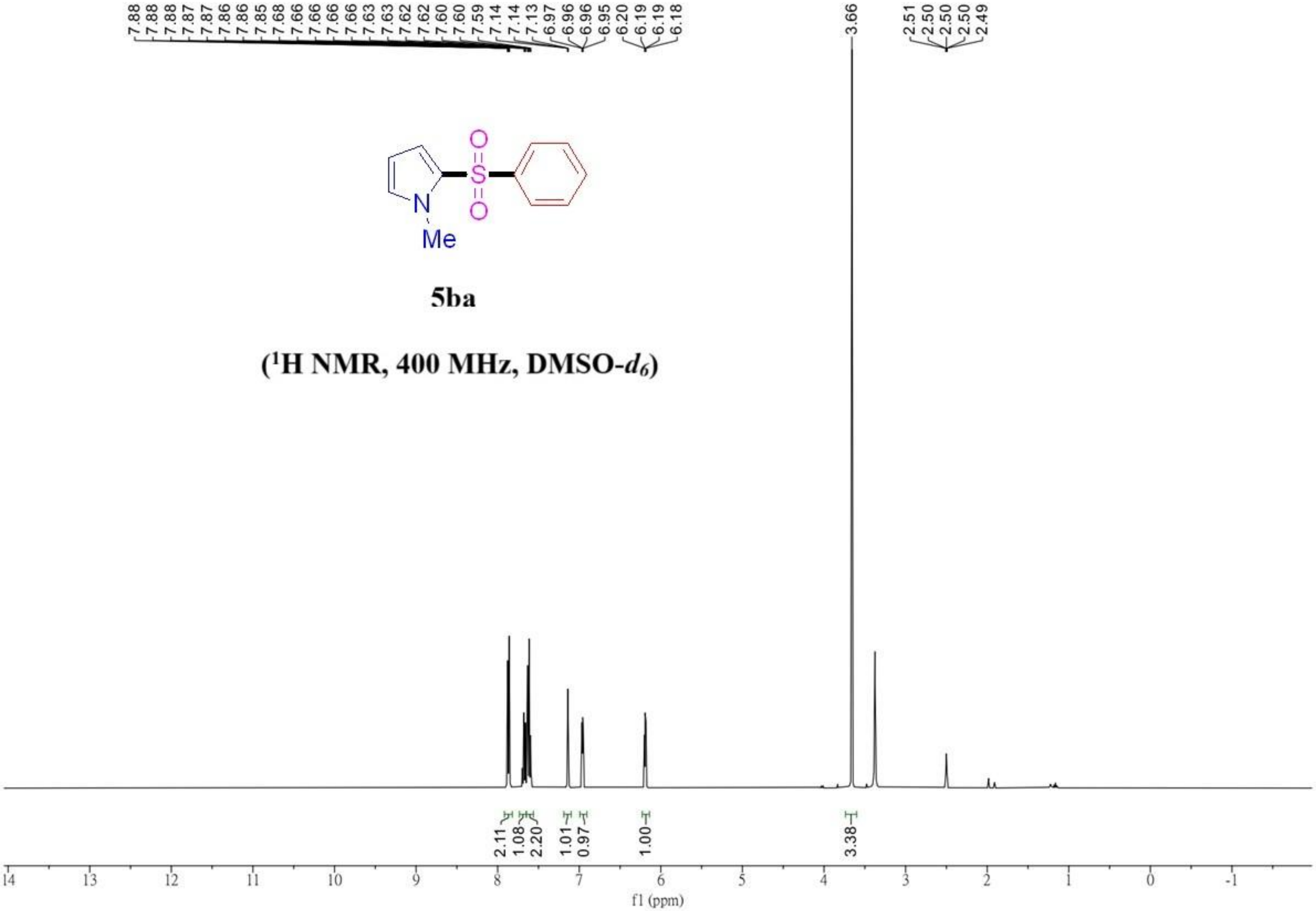


**5ba**

**($^1$H NMR, 400 MHz, DMSO-$d_6$)**

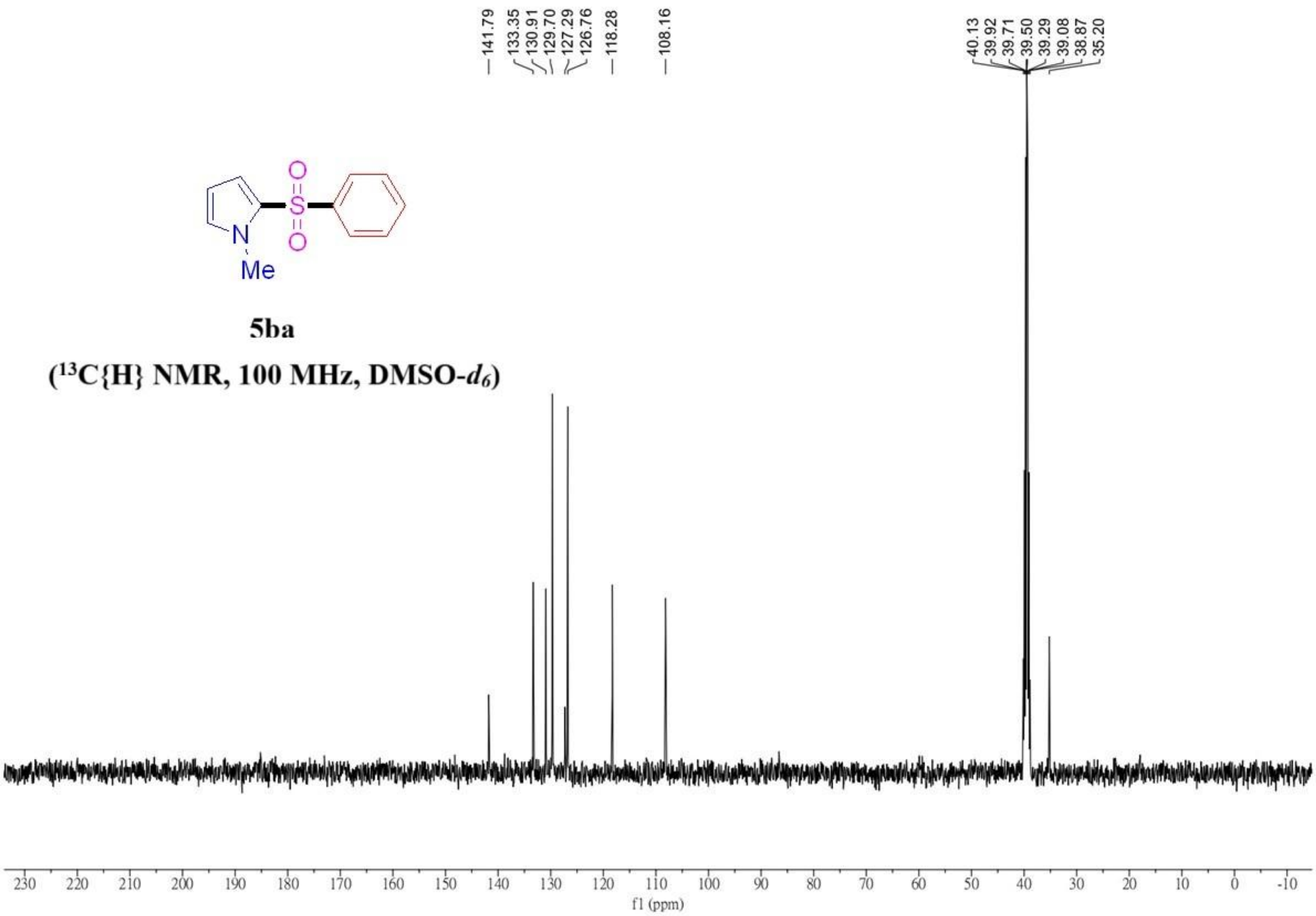


**5ba**

**($^{13}$C{H} NMR, 100 MHz, DMSO-$d_6$)**

**5bb**

**($^{1}$H NMR, 400 MHz, DMSO-$d_6$)**

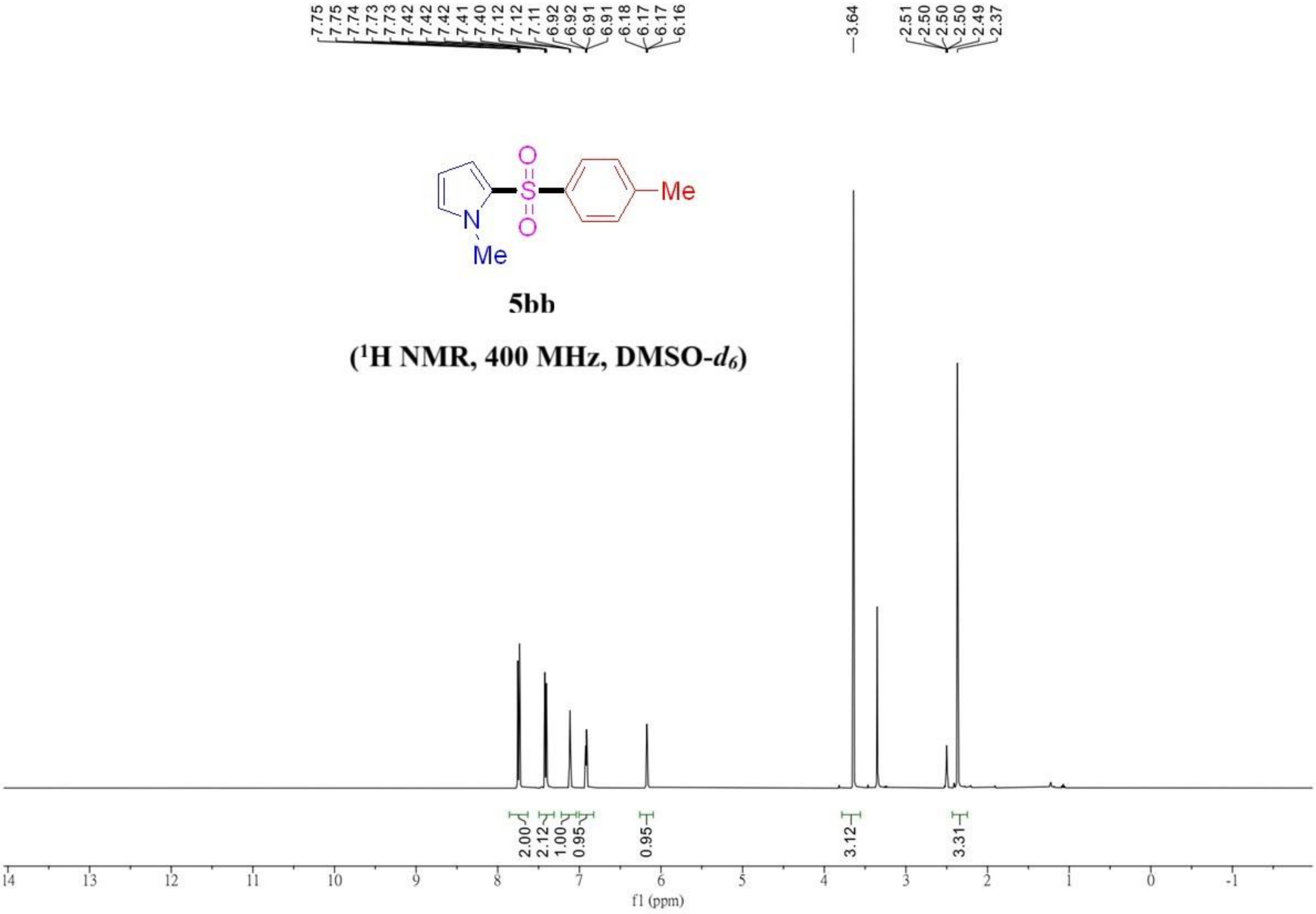

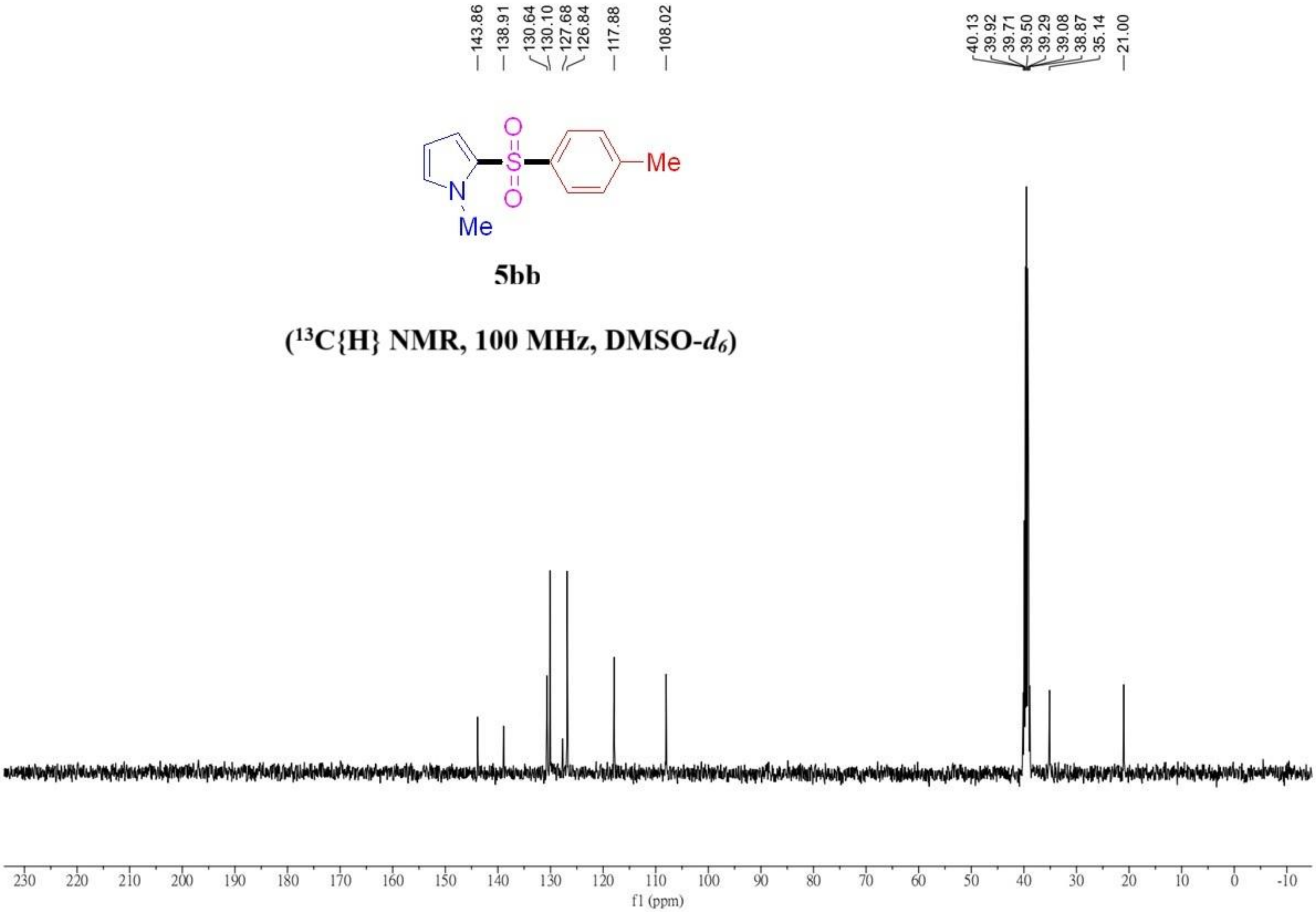


**5bb**

**($^{13}$C{H} NMR, 100 MHz, DMSO-$d_6$)**

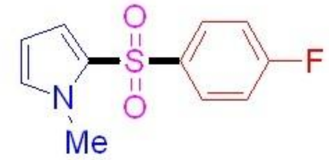

**5bc**

**($^{1}$H NMR, 400 MHz, DMSO-$d_6$)**

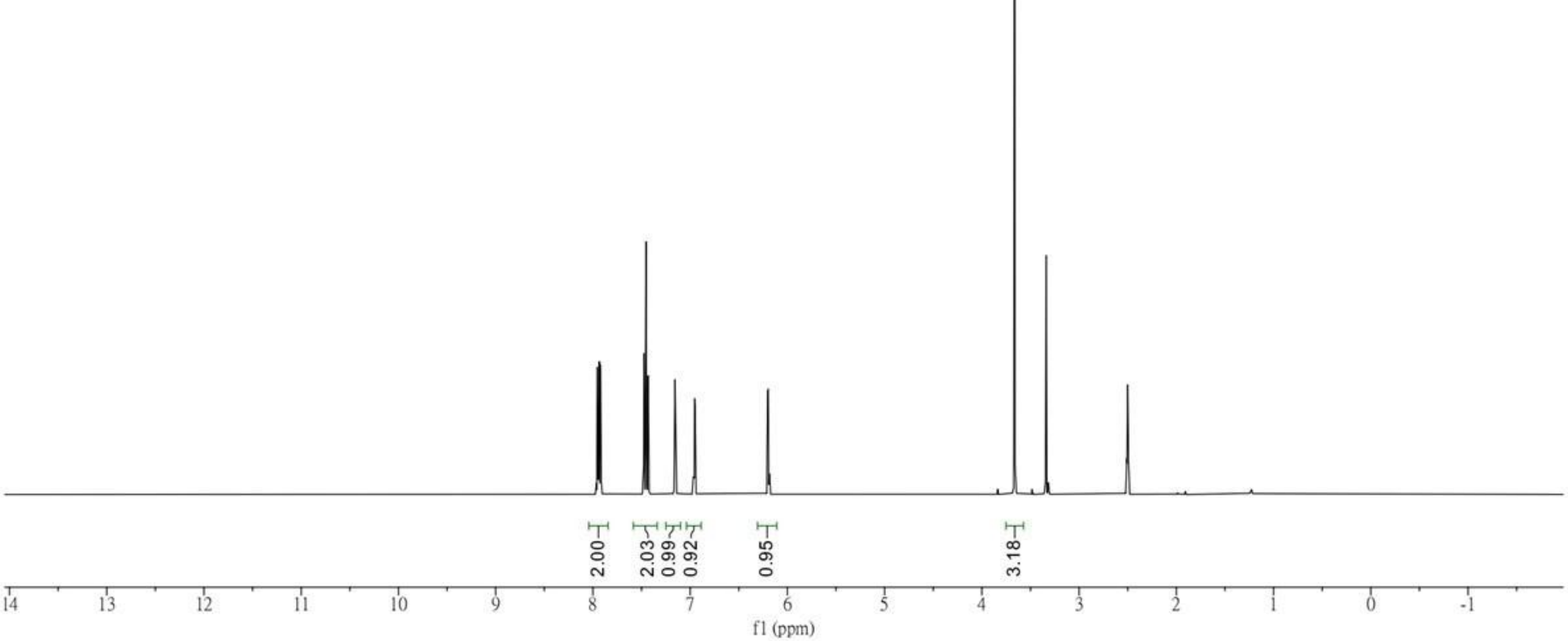

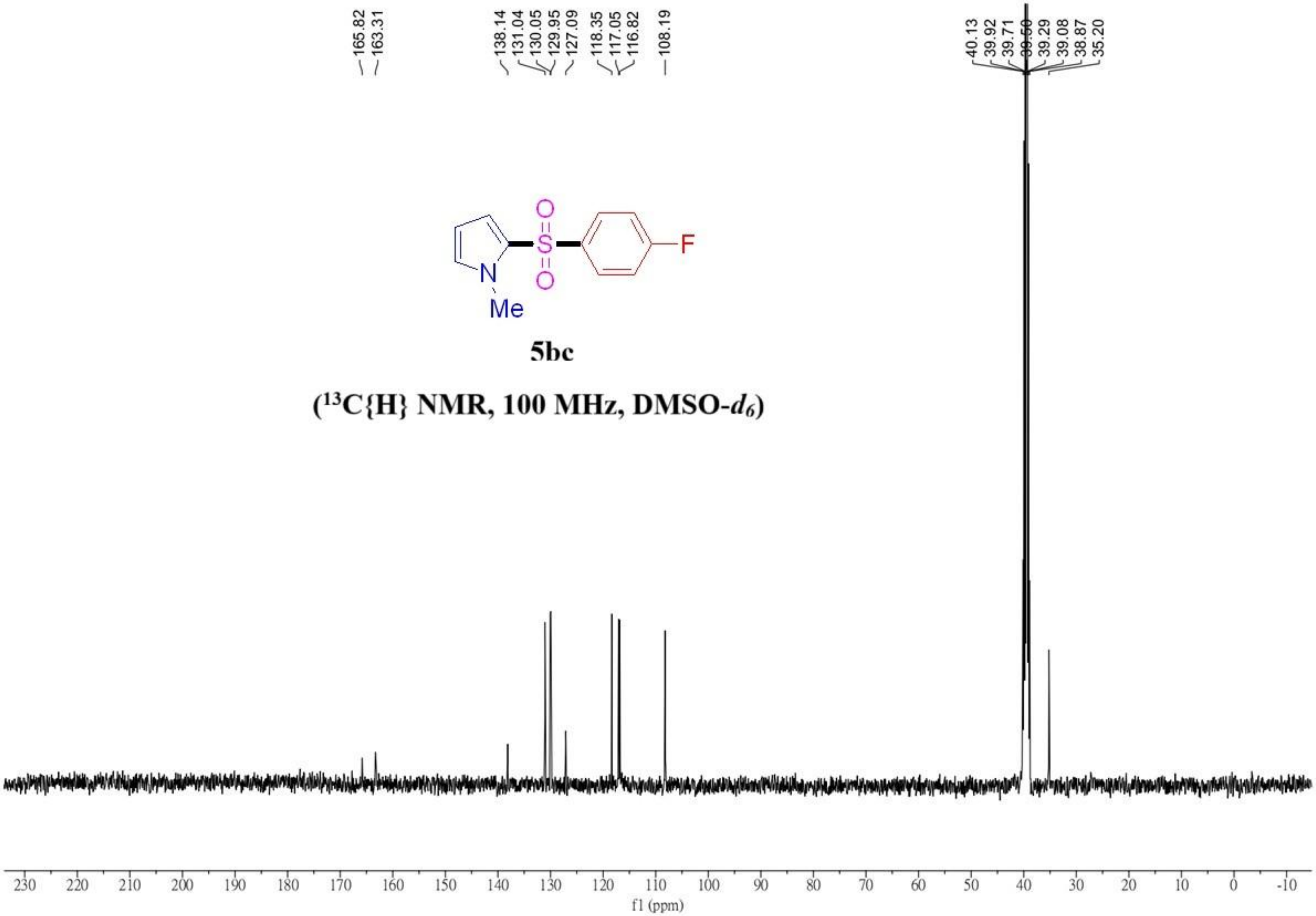
165.82
163.31
138.14
131.04
130.05
129.95
127.09
118.35
117.05
116.82
108.19
40.13
39.92
39.71
39.50
39.29
39.08
38.87
35.20
O
S
O
N
Me
F
5bc
($^{13}$C{H} NMR, 100 MHz, DMSO-$d_6$)
230 220 210 200 190 180 170 160 150 140 130 120 110 100 90 80 70 60 50 40 30 20 10 0 -10
f1 (ppm)

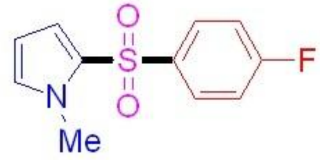

**5bc**

**($^{19}$F NMR, 376 MHz, DMSO-$d_6$)**

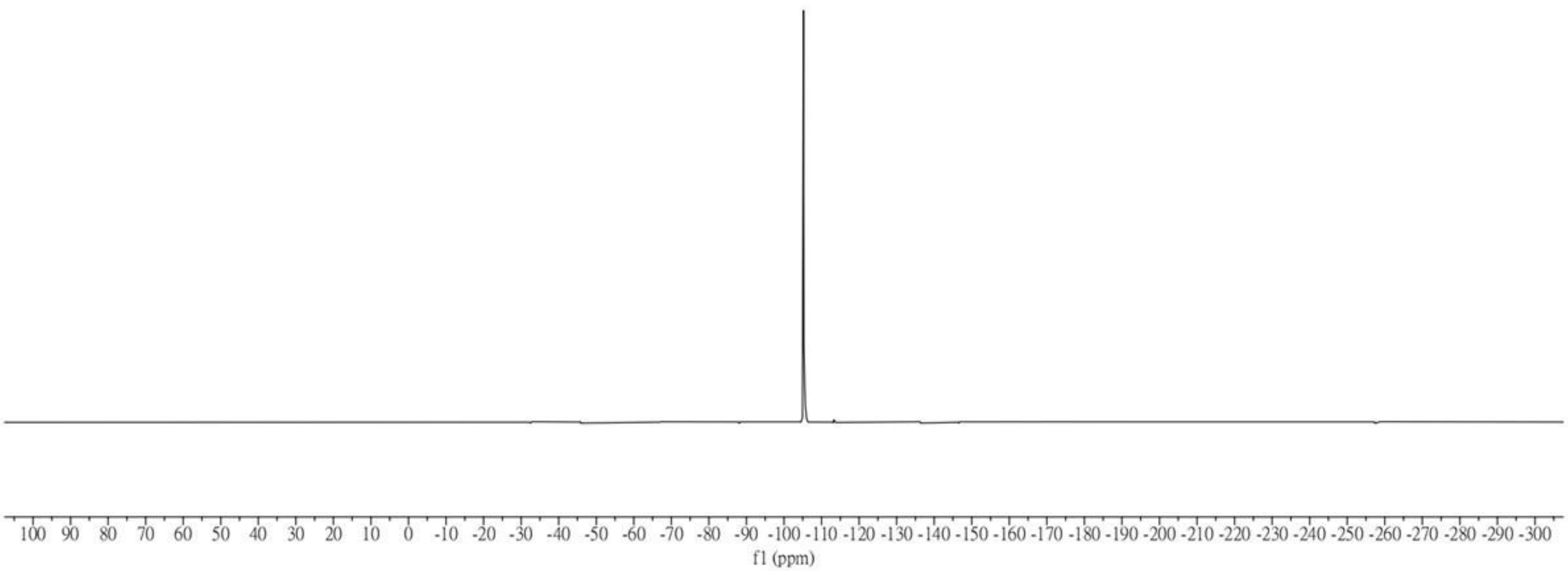

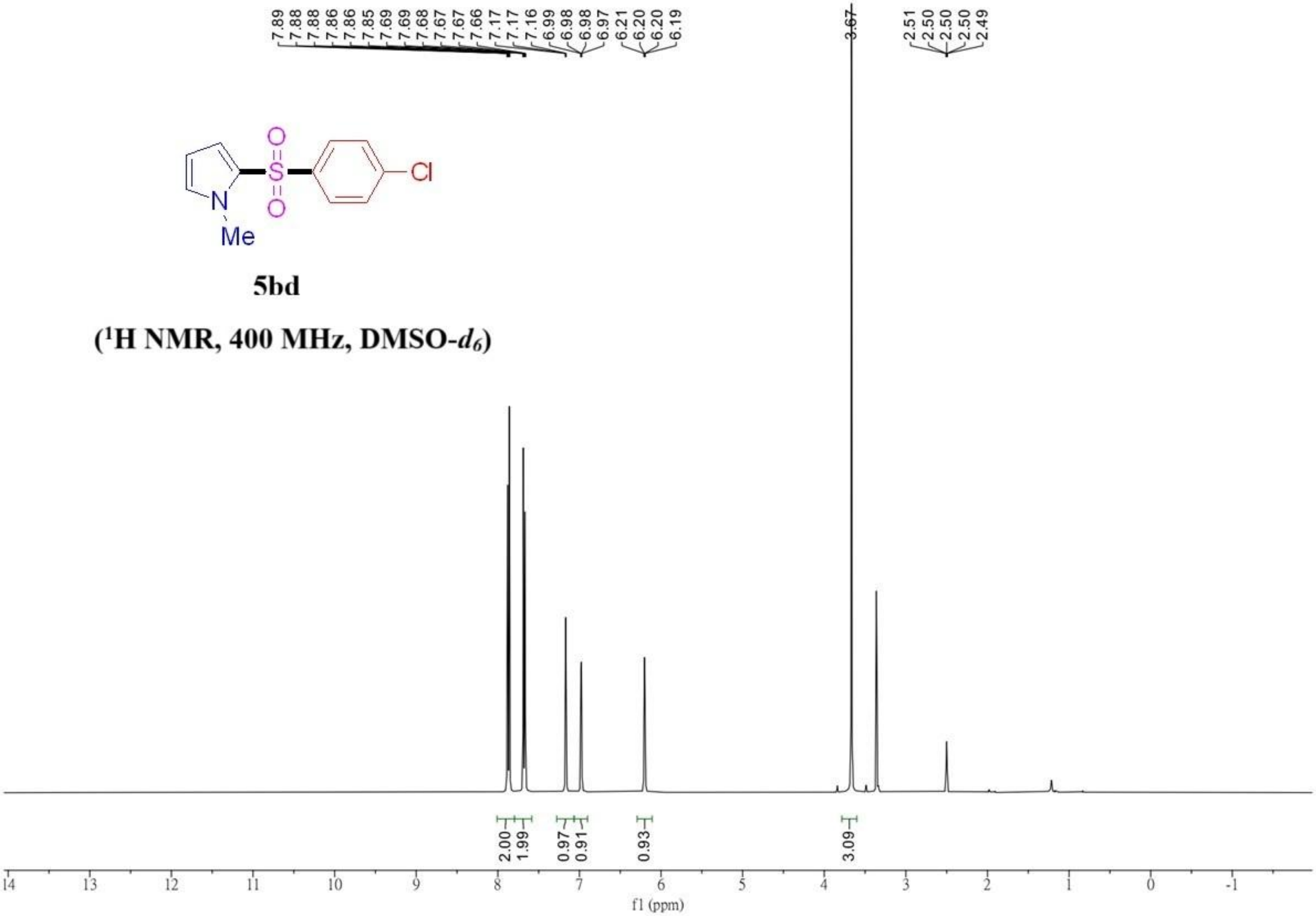


**5bd**

**($^1$H NMR, 400 MHz, DMSO-$d_6$)**

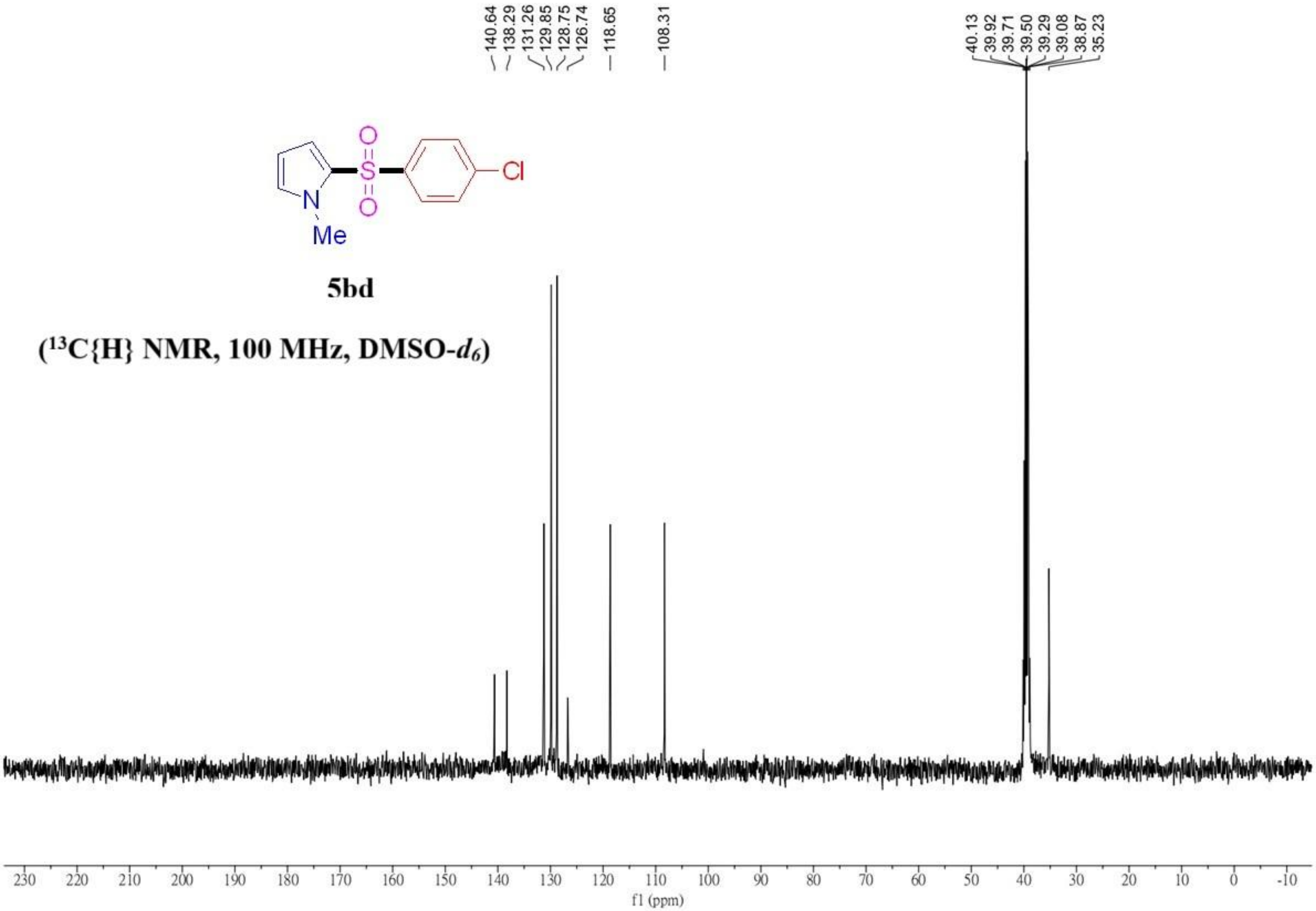


**5bd**

**($^{13}$C{H} NMR, 100 MHz, DMSO-$d_6$)**

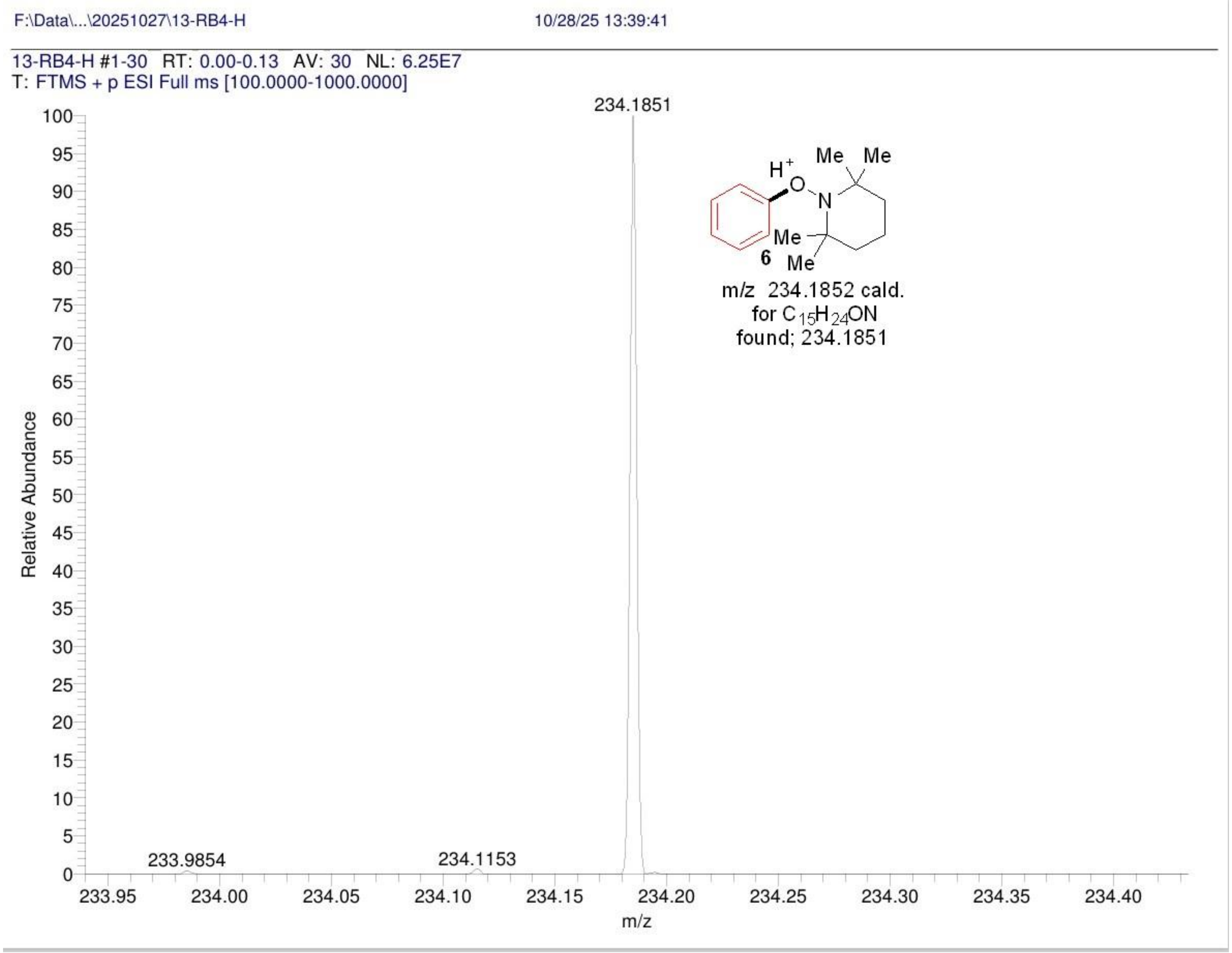

F:\Data\...\20251027\13-RB4-H
10/28/25 13:39:41
13-RB4-H #1-30 RT: 0.00-0.13 AV: 30 NL: 6.25E7
T: FTMS + p ESI Full ms [100.0000-1000.0000]
234.1851
233.9854
234.1153
Relative Abundance
m/z
H+
Me
Me
Me
Me
O
N
6
m/z 234.1852 cald.
for C15H24ON
found; 234.1851

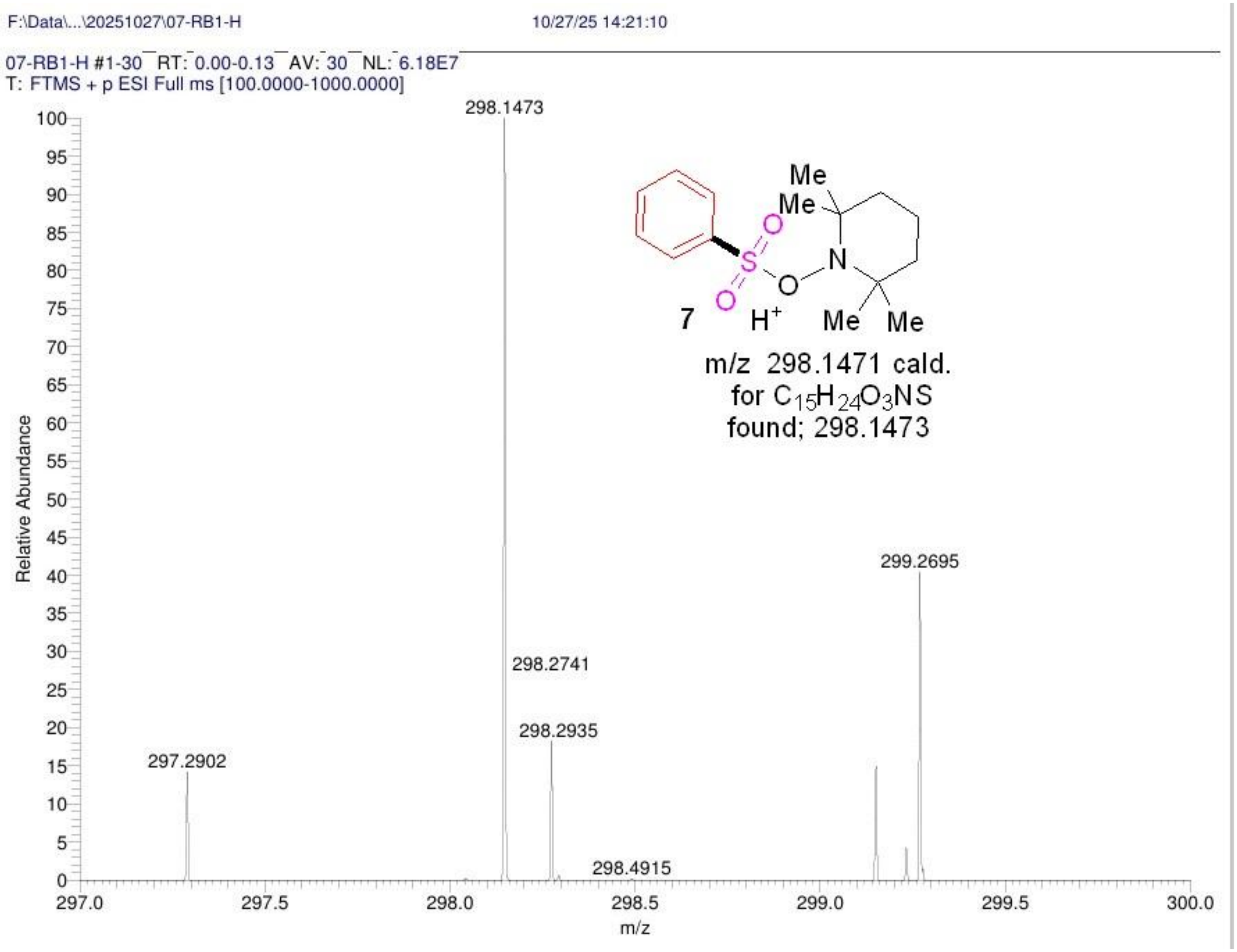
F:\Data\...\20251027\07-RB1-H
10/27/25 14:21:10
07-RB1-H #1-30 RT: 0.00-0.13 AV: 30 NL: 6.18E7
T: FTMS + p ESI Full ms [100.0000-1000.0000]
Relative Abundance
m/z
298.1473
298.2741
298.2935
298.4915
297.2902
299.2695
Me
Me
N
O
S
O
O
Me
Me
7
H+
m/z 298.1471 cald.
for C15H24O3NS
found; 298.1473

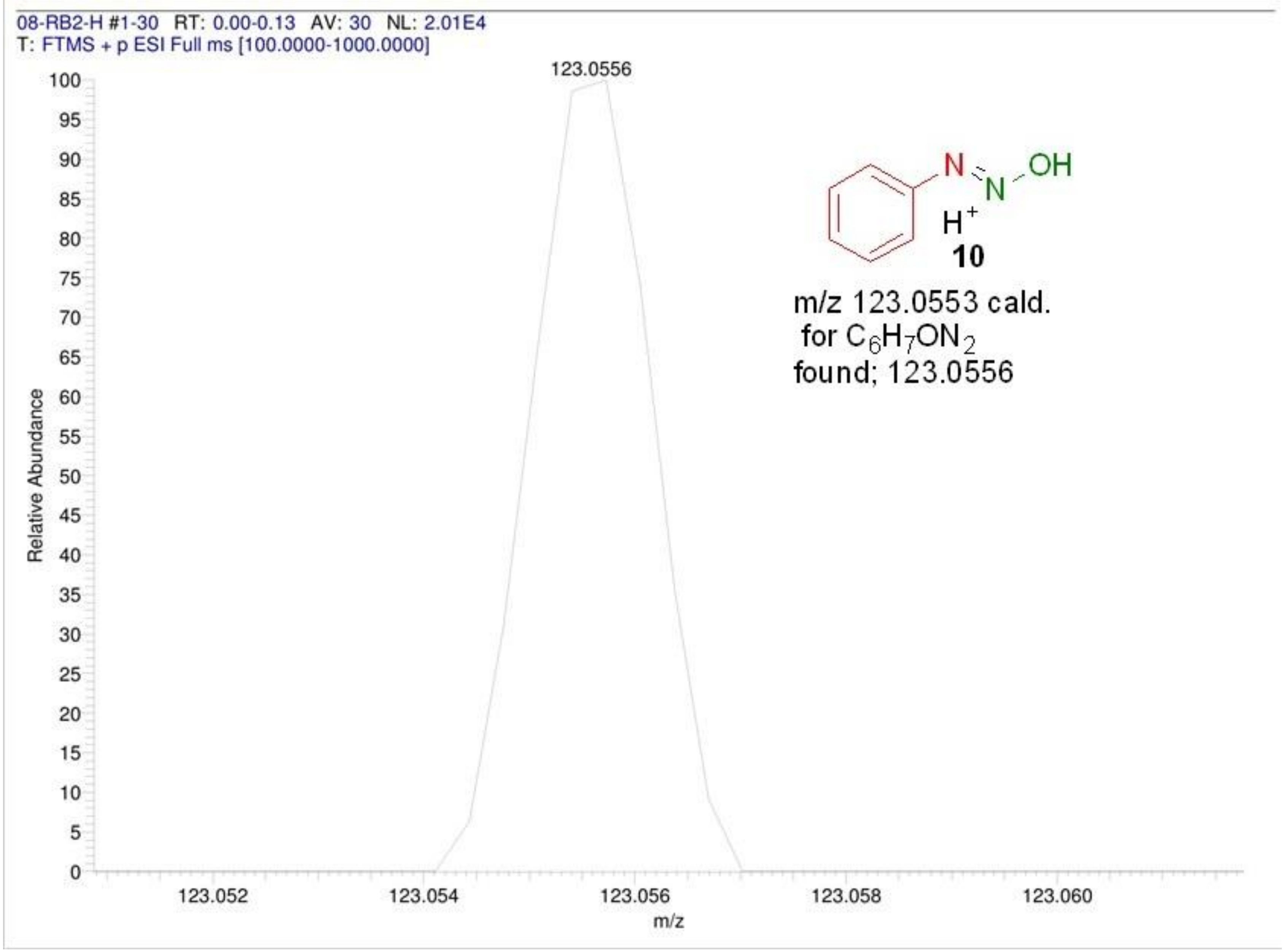
08-RB2-H #1-30 RT: 0.00-0.13 AV: 30 NL: 2.01E4
T: FTMS + p ESI Full ms [100.0000-1000.0000]
123.0556
Relative Abundance
m/z
N
N
OH
H+
10
m/z 123.0553 cald.
for C6H7ON2
found; 123.0556
